# Evolution of neuropeptides in non-pterygote hexapods

**DOI:** 10.1186/s12862-016-0621-4

**Published:** 2016-02-29

**Authors:** Christian Derst, Heinrich Dircksen, Karen Meusemann, Xin Zhou, Shanlin Liu, Reinhard Predel

**Affiliations:** Institute for Zoology, Functional Peptidomics Group, University of Cologne, D-50674 Cologne, Germany; Department of Zoology, Stockholm University, S-10691 Stockholm, Sweden; Center for Molecular Biodiversity Research, Zoological Research Museum A. Koenig, D-53113 Bonn, Germany; Australian National Insect Collection, CSIRO National Research Collections Australia, Acton, ACT 2601 Canberra, Australia; China National GeneBank, BGI-Shenzhen, Shenzhen, Guangdong Province 518083 China

**Keywords:** Neuropeptides, Transcriptome, Archaeognatha, Collembola, Crustacea, Diplura, Myriapoda, Protura, Remipedia, Zygentoma

## Abstract

**Background:**

Neuropeptides are key players in information transfer and act as important regulators of development, growth, metabolism, and reproduction within multi-cellular animal organisms (Metazoa). These short protein-like substances show a high degree of structural variability and are recognized as the most diverse group of messenger molecules. We used transcriptome sequences from the 1KITE (1K Insect Transcriptome Evolution) project to search for neuropeptide coding sequences in 24 species from the non-pterygote hexapod lineages Protura (coneheads), Collembola (springtails), Diplura (two-pronged bristletails), Archaeognatha (jumping bristletails), and Zygentoma (silverfish and firebrats), which are often referred to as “basal” hexapods. Phylogenetically, Protura, Collembola, Diplura, and Archaeognatha are currently placed between Remipedia and Pterygota (winged insects); Zygentoma is the sistergroup of Pterygota. The Remipedia are assumed to be among the closest relatives of all hexapods and belong to the crustaceans.

**Results:**

We identified neuropeptide precursor sequences within whole-body transcriptome data from these five hexapod groups and complemented this dataset with homologous sequences from three crustaceans (including *Daphnia pulex*)*,* three myriapods, and the fruit fly *Drosophila melanogaster*. Our results indicate that the reported loss of several neuropeptide genes in a number of winged insects, particularly holometabolous insects, is a trend that has occurred within Pterygota. The neuropeptide precursor sequences of the non-pterygote hexapods show numerous amino acid substitutions, gene duplications, variants following alternative splicing, and numbers of paracopies. Nevertheless, most of these features fall within the range of variation known from pterygote insects. However, the *capa/pyrokinin* genes of non-pterygote hexapods provide an interesting example of rapid evolution, including duplication of a neuropeptide gene encoding different ligands.

**Conclusions:**

Our findings delineate a basic pattern of neuropeptide sequences that existed before lineage-specific developments occurred during the evolution of pterygote insects.

**Electronic supplementary material:**

The online version of this article (doi:10.1186/s12862-016-0621-4) contains supplementary material, which is available to authorized users.

## Background

Insects diverged more than 440 mya [1] and are currently the most speciose animal group, with numerous ecologically and economically important lineages. Knowledge about insect diversity, including particular physiological adaptations and life histories, is essential for the development of novel strategies to control pest species as well as medically important vectors without destabilizing or destroying complete ecosystems. One of the key players in information transfer, acting as important regulators of development, growth and reproduction within Metazoa, are the neuropeptides. They represent the most diverse group of messenger molecules with regard to numbers and primary structures. Ongoing genome and transcriptome projects and an increasing number of studies identifying processed insect neuropeptides through mass spectrometry are providing comprehensive data to elucidate trends in the evolution of neuropeptides. Thus, ancient and conserved sequences can be discriminated from derived sequence substitutions that mostly occur only in single insect lineages.

Thorough peptidomic analyses of mature neuropeptides are mainly limited to species for which complete genome data are available. In most cases, these species serve as model organisms, often represented by holometabolous insects (e.g., Diptera: *Drosophila melanogaster* [2–4], Coleoptera: *Tribolium castaneum* [5], Hymenoptera: *Apis mellifera* [6]). Among larger polyneopterans in particular, such as locusts and cockroaches, and medically important heteropterans (e.g., *Rhodnius prolixus*), neuropeptides have been identified and analyzed via mass spectrometry prior to genome sequencing [7–10]. However, nearly nothing is known about the neuropeptides of the non-pterygote hexapods, which comprise the entognathous Protura (coneheads), Collembola (springtails), and Diplura (two-pronged bristletails) as well as the ectognathous Archaeognatha (bristletails) and Zygentoma (silverfishs and firebrats). Only a first compilation of the neuropeptide precursors of *Acerentomon* sp. (Protura) using data from 1KITE has been recently published [11].

**Fig. 1:**
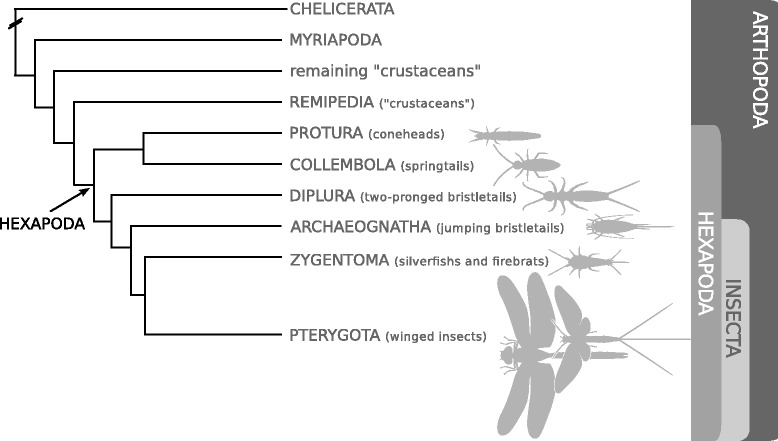
Simplified topology of the phylogenetic relationships among non-pterygote hexapods and arthropod outgroup taxa; modified from [1]

We employed sequences from transcriptome shotgun assemblies from 1KITE (www.1kite.org) to search for neuropeptide-containing precursor sequences in 24 species from all major groups of non-pterygote hexapods (Protura, Collembola, Diplura, Archaeognatha, Zygentoma). Inferred phylogenetic relationships among these hexapod lineages and their positions within the arthropods were derived from transcript libraries ([1], see Fig. 1). The neuropeptide precursor sequences from the non-pterygote hexapods were then complemented with homologous sequences from the transcripts of two crustaceans and three myriapods (transcript libraries from 1KITE) and from the genomes of the water flea *Daphnia pulex* (Branchiopoda, crustacean) and the fruit fly *D. melanogaster* (Diptera). The genomes of *D. pulex* and *D. melanogaster* have previously been screened for neuropeptide genes and their encoded precursors. Mature neuropeptides from these species have been confirmed via mass spectrometry (e.g., [2–4, 12]) and provided reference points for the assignment of putative mature peptides from non-pterygote hexapods. Since several database entries, particularly those for *D. pulex*, contained doubtful submissions, we here provide an updated list for these species. Considering the results for all of the compared species, we assigned more than 1,300 neuropeptide/protein hormone precursors to 39 neuropeptide or protein hormone genes. Specific features, such as distinctive sequence motifs, numbers of paracopies, gene duplications and the occurrence of splice variants, clustered within the well-described systematic groups Protura, Collembola, Diplura, Archaeognatha, and Zygentoma. Our data provide the first comprehensive overview of the neuropeptide complement of the non-pterygote hexapods and therefore allow a reasonable estimation of the basic pattern that existed before lineage-specific developments occurred in the pterygote insects.

## Results and discussion

The transcriptome data analyzed herein were generally of high quality, with a sequencing depth of 2.5 Gbases of raw sequence reads per species, as illustrated by comparison of the number of neuropeptide precursors deduced from recently published Remipedia EST data (11 precursors, [13, 14]) and those deduced in this study from the 1KITE transcriptome assemblies (24 precursors). Ongoing peptidomic analyses of neuropeptides found in the American cockroach (*Periplaneta americana*) and firebrat (*Thermobia domestica*) (S Neupert, M Bläser, R Predel; unpublished results), also using data from 1KITE, did not reveal any obvious sequence errors within mature peptide sequences. We corrected few obvious errors in the dataset analyzed in this study (frameshifts in sequences or in-frame stop codons) if sequencing errors were considered to be more likely than the true occurrence of non-functional genes. These corrections are indicated in our datasets and the original GenBank data remained unchanged.

We screened the assembled transcript libraries for the following neuropeptide-containing precursors: adipokinetic hormone/corazonin-related peptide (ACP), adipokinetic hormone (AKH), FGLamide allatostatin (AST-A), allatostatin C and CC (AST-C, AST-CC), allatotropin (AT), CAPA, crustacean cardioactive peptide (CCAP), CCHamide1 (CCHa1), CCHamide2 (CCHa2), corazonin, CNMamide (CNMa), corticotropin-releasing factor-related diuretic hormone (CRF-DH), calcitonin-related diuretic hormone (CT-DH), elevenin, ecdysis-triggering hormone (ETH), extended FMRFamide (FMRFa), inotocin, insect kinin, ion transport peptide (ITP), myoinhibitory peptide (MIP/AST-B), myosuppressin (MS), natalisin, neuropeptide F (NPF), neuropeptide-like precursor1 (NPLP1), orcokinin and orcomyotropin (orcokinin A, B), pigment-dispersing factor (PDF), proctolin, pyrokinin/pheromone biosynthesis activating neuropeptide (PK/PBAN), RYamide (RYa), SIFamide (SIFa), EFLamide (EFLa), sulfakinin (SK), short neuropeptide F (sNPF), tachykinin-related peptide (TKRP), and trissin. For most of the neuropeptides that we searched for in this study, the corresponding G-protein-coupled receptors are known from insects [15–17]. For the NPLP1 peptides, a membrane-bound guanylate cyclase has been described as a receptor in *D. melanogaster* [18]. Receptors for the mature products of the orcokinin, elevenin, and EFLa precursors are hitherto unknown. In addition to the aforementioned neuropeptide-containing precursors, we searched the transcript assemblies for the presence of cysteine-rich hormone-encoding precursors of bursicon-α, bursicon-β, and eclosion hormone (EH). The biological functions of neuropeptides and protein hormones, where available, have been explained in detail in recent publications [19, 20].

### Neuropeptide precursors of *Lepidocampa weberi* (Diplura)

**Fig. 2:**
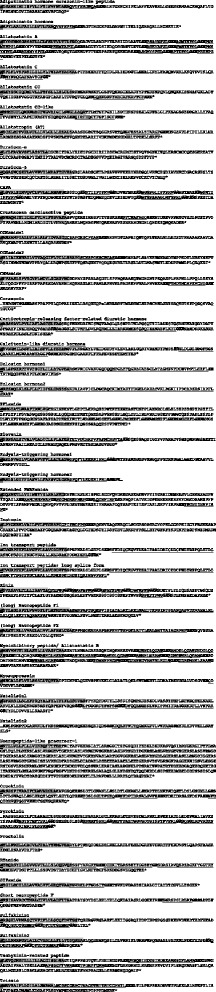
*Lepidocampa weberi* (Diplura) precursor sequences of putative neuropeptides and selected protein hormones (bursicon, EH)*.* In *L. weberi*, the transcript sequences cover the full-length sequences of most precursors; note the distinct novel CCAP sequence. We did not find pigment-dispersing factor (PDF)-containing precursors in any proturan and dipluran, but they were identified in all other non-pterygote hexapods (i.e., Collembola, Archaeognatha, and Zygentoma). Predicted signal peptides (highlighted in grey), amidation signals (bold), cleavage signals (italics, bold), splice variants (a, b), and supposedly bioactive mature peptides (underscored) are indicated. Incomplete sequences are indicated with “…”

The neuropeptide and protein hormone precursors of a single species, the dipluran *Lepidocampa weberi*, are shown in Fig. 2. This species was selected since we identified nearly all of the above-mentioned neuropeptide/protein hormone precursors, and most of the precursor sequences were full-length. To our surprise, we could not detect a PDF precursor, a finding that holds true for all of the investigated diplurans. The sequences of most of the predicted mature peptides showed typical features known for the neuropeptides of pterygote insects. However, two of the single-copy peptides, CCAP and SIFa, exhibited substitutions within highly conserved sequence motifs (PFCNAF**A**GCa, NNVRKLPFNGSI**Y**a). Among the non-pterygote hexapods analyzed in this study, these amino acid substitutions are only present in *L. weberi* and the closely related *Campodea augens* (Diplura). Interestingly, the derived dipluran CCAP is also known from *D. pulex* [12]. In addition to the two commonly occurring CCHa precursors, we found two precursors of EH, ETH, natalisin, NPF and SK in *L. weberi*. This indicates the presence of two genes for each of the six precursors. We discovered splice variants only for ITP.

### Neuropeptide precursor sequences from 24 species of non-pterygote hexapods, 3 myriapods, 3 crustaceans, and the fruit fly

The complete sets of neuropeptide precursors for three proturan species, nine collembolan species, four dipluran species, four species of Archaeognatha, and four species of Zygentoma are listed in Additional file 1. Our main reason for using the transcriptome data of species from the 1KITE project was the exceptional coverage of major lineages of non-pterygote hexapods in this project. This enabled us to obtain a reasonable overview of the evolution of neuropeptides within these lineages as well as sufficient information regarding highly conserved sequences. In most cases, the sequence motifs of predicted mature neuropeptides, the numbers of paracopies in multiple-copy precursors, gene duplications and the occurrence of splice variants were observed to cluster within the different non-pterygote hexapod lineages, with significant leaps in the manifestation of such features being observed between these taxa. We found only a few indications of duplicated genes encoding single-copy peptides, such as ACP, AKH, AST-C, AST-CC, CCAP, CT-DH, CRF-DH, CCHa1, CCHa2, CNMa, corazonin, elevenin, inotocin, ITP, MS, NPF, PDF, proctolin, sNPF, SIFa, and trissin. The respective genes showing a scattered occurrence within Protura, Diplura, Collembola, Archaeognatha and Zygentoma comprise ACP, AKH, AST-C, AST-CC, corazonin, ITP, MS, NPF, PDF, proctolin, SIFa, sNPF, and trissin. Notably, in *Nipponentomon nippon* (Protura), two precursors of each of the closely related ACP, AKH, and corazonin genes are present, which is not the case in any other of the examined lineages. Only NPF was found to exhibit two (occasionally three) precursors in most species, whereas we identified 2–3 SIFa precursors and 2–5 ITP precursors (*Tricholepidion* 2, *Jordanathrix* 4, *Sminthurus* 5) in at least a few species. Two splice variants of ITP (ITP/ITPL) are common in non-pterygote hexapods, as is typical of many arthropods [21]. In addition, we found splice variants of orcokinins in all collembolan species, in 3 out of 4 jumping bristletails (Archaeognatha) and in all zygentoman species, but not in any of the examined species of Protura and Diplura.

**Fig. 3:**
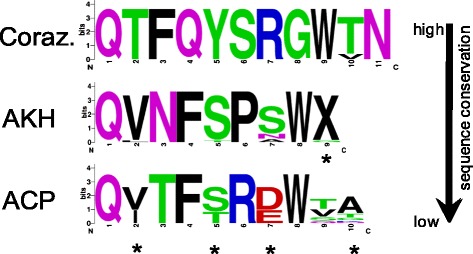
Sequence logos illustrating the different degrees of conserved sequence motifs in the neuropeptides of non-pterygote hexapods. The genes encoding corazonin, AKH, and ACP likely developed independently from an ancient metazoan gene following gene duplications. Substitutions at amino acid positions indicated with asterisks were found only in collembolan species. X, no amino acid occurring at this position

Comparison of the sequence conservation of single-copy peptides in non-pterygote hexapods clearly showed that these peptides present very different degrees of conservation (see Additional file 2). Proctolin and MS are identical in all species, and the substitutions present in corazonin and inotocin are restricted to one or two amino acids, respectively. The sequences of AKH, PDF and CCAP are slightly more variable, although the observed amino acid substitutions are restricted to a few positions within the respective peptides. CCAP is generally highly conserved in arthropods but shows distinct amino acid substitutions in Collembola (e.g., **T**FCNAFTGCa); in a few cases, we even found very unusual C-terminal extensions and deletions (**T**FCNAFTGC**A**a, **T**FCNAFTGC**Q**a, **T**FCNAF**-**GC**Q**a). Figure 3 illustrates the conserved sequence motifs present in corazonin, AKH and ACP. The corresponding genes likely developed independently from an ancient precursor gene, followed by gene duplications [22]. For corazonin, AKH, and ACP, the conservation of amino acid sequences differs remarkably among the non-pterygote hexapods. In fact, the number of amino acid substitutions within ACPs is comparable with the substitutions observed in multiple-copy peptides such as periviscerokinins (see Additional file 2). These different ACP sequences are, however, not evenly distributed within the non-pterygote hexapods. Protura, Diplura, and Archaeognatha + Zygentoma each exhibit characteristic ACPs that differ only in their penultimate amino acids. In contrast, the eight collembolan species show a basic pattern of ACP in which five amino acids, including the penultimate amino acids, differ from those observed in Protura, Diplura, Archaeognatha, and Zygentoma. Longer single-copy peptides, such as NPF, CRF-DH, trissin, ITP, and CT-DH, display even greater sequence variability. This is also the case for the large protein hormones bursicon and EH, which in a strict sense are single-copy peptides as well. Bursicon and EH show conservation mainly with regard to the positions of the cysteines. Two *eh* genes are common in non-pterygote hexapods. However, there are only single genes encoding the protein hormone subunits bursicon-α and bursicon-β. Similarly, a single *itp* gene is present in most species, but *itp* expression generally results in at least two splice variants.

In addition to the single-copy peptide precursors, there are a number of precursors that contain at least two paracopies of putative bioactive peptides, comprising the precursors for AST-A, CAPA/PK, FMRFa, EFLa, ETH, kinin, MIP, RYa, SK, TKRP, and natalisin. As observed for the sequences of many predicted neuropeptides, the number of paracopies in precursor sequences separates the springtails (Collembola) from the other non-pterygote hexapods (Table 1). More precisely, the Collembola contain the lowest number of paracopies of AST-A, EFLa, ETH, kinin, MIP, RYa, TKRP, and natalisin.

**Table 1:** Average number of paracopies with the uncorrected sample standard deviation (S_N_) in precursors with multiple-copy peptides. Only full-length precursor sequences are considered; data without S_N_ refer to a single complete precursor sequence. For hexapod orders lacking full-length precursor sequences, the maximum number in a partial sequence is given in parentheses. Note, that Collembola show the lowest number of paracopies by far

	Protura	Collembola	Diplura	Archaeognatha	Zygentoma
AST A	14 ± 0.8	4.2 ± 0.9	12.5 ± 0.5	15.3 ± 1.7	18 ± 2.9
MIP	9 ± 1	5.3 ± 0.9	11 ± 0	9.5 ± 0.5	12.2 ± 1.1
CAPA/PK:					
PVK	4 ± 0	3 ± 0	3.5 ± 0.5	4 ± 0	3 ± 1
trypto-PK	1 ± 0	1 ± 0	1 ± 0	1 ± 0	1.75 ± 0.4^a^
PK	3 ± 0	3.6 ± 1.1	4.5 ± 0.5	3.7 ± 0.4	1.7 ± 0.2
FMRFa	2 ± 0	3 ± 0	4 ± 0	7 ± 0.8	11.6 ± 2.9
TKRP	5 ± 0	3.6 ± 0.47	4.3 ± 0.9	8	9 ± 0
Natalisin	6 ± 0	2	3	(≥10)	(≥9)
EFLa	(≥18)	4.5 ± 0.5	9 ± 2.2	12	17.5 ± 0.5
Kinin	6.5 ± 0.5	4 ± 2.5	5	(≥20)	(≥19)
RYa	1.3 ± 0.5	2 ± 0	3 ± 0	3 ± 0	3 ± 0
ETH	2 ± 0	1 ± 0	2 ± 0	2 ± 0	2
SK	2 ± 0	2.1 ± 0.3	2 ± 0	2 ± 0	2 ± 0

^a^separate CAPA and PK precursors, usually with a single trypto-PK each

We complemented the list of precursor sequences from non-pterygote hexapods with homologous sequences from three myriapod and two crustacean species (transcripts from 1KITE), and we newly revised all of the available genome data for *D. pulex* [23] and *D. melanogaster* [24] (see Additional file 1). The genomes of the last two species have been thoroughly analyzed for neuropeptide genes and mature peptides (e.g., [2–4, 12]). In general, 1KITE data provide comprehensive coverage with respect to both the presence and length of precursor sequences. In a few cases, available EST data from *Xilbalbanus tulumensis* (Remipedia, [13, 14]) and *Folsomia candida* (Collembola, [25]) were successfully applied to complete the precursor sequences in these species.

Our datasets provide a first comprehensive survey of the development of neuropeptide precursor sequences in non-pterygote hexapods. We identified nearly all of the examined neuropeptide precursors in Protura, Collembola, Diplura, Archaeognatha, and Zygentoma. The complete absence of a specific precursor was the exception rather than the rule, as observed for PDF in Protura and Diplura, elevenin in Collembola, and CNMa in Protura and Collembola. In contrast, the peptidomes of *D. melanogaster* and *D. pulex* lack a number of neuropeptides, such as kinin, trissin, PK and NPLP1 peptides, in *D. pulex*, and ACP, AT, EFLa, elevenin and inotocin, in *D. melanogaster*. Therefore, the compiled sequences of the non-pterygote hexapods indicate that the neuropeptidomes of *D. melanogaster* and *D. pulex* each represent a rather derived condition.

### The *capa/pk* genes as an example of the rapid evolution of a three-ligand gene

**Fig. 4:**
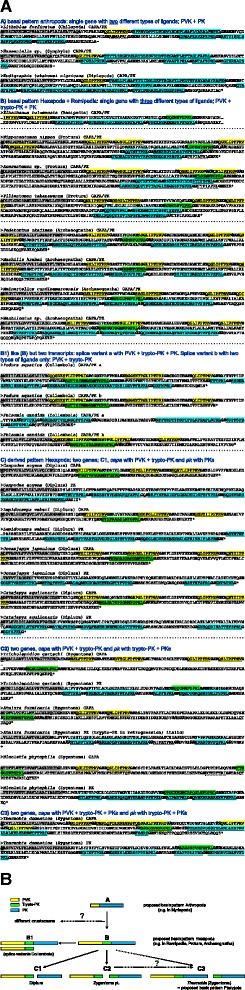
Evolution of *capa/pk* genes. Transcript sequence data indicate rapid evolution of the *capa*/*pk* gene(s) with the divergence of Remipedia and the hexapods. This rapid evolution includes the appearance of novel ligands, gene duplications, and subsequent sorting of the three putative ligands in the resulting *capa* and *pk* genes. The most derived pattern (pattern C3, Zygentoma) appears to be typical of many insect taxa. For *D. melanogaster*, it has been verified that PVK (yellow), PK (blue) and trypto-PK (green) each exhibit specific receptors (see [19]). **a** Sequences of CAPA/PK precursors assigned to different types with respect to the evolution of *capa/pk* genes. Predicted signal peptides (highlighted in grey), amidation signals (bold), cleavage signals (italics, bold), splice variants (a, b), and supposed bioactive mature peptides (underscored) are indicated. Incomplete sequences are indicated with “…”. Peptidomic studies have not been performed for any of these species. **b** Overview of the putative evolution of *capa/pk* genes, as indicated by analyses of transcript sequences. According to the phylogenetic relationships of the non-pterygote hexapods (Fig. 1), gene duplication and subsequent evolutionary development of discrete *capa* and *pk* genes must have occurred at least twice, in Diplura and in Zygentoma. The evolution of the type C3 pattern (*Thermobia domestica*, Zygentoma), which likely also represents the basic pattern in winged insects (Pterygota), took place either via the type C2 pattern or directly from the type B pattern

The presumed evolution of the CAPA/PK precursors of non-pterygote hexapods provides particularly interesting insights into the rapid evolution of a neuropeptide gene/precursor (Fig. 4). Current knowledge about *capa/pk* genes and their products suggests an ancient condition in arthropods in which a single gene encodes two types of neuropeptides (ligands with specific receptors each), the periviscerokinins (PVKs) and pyrokinins (PKs). This basic pattern (pattern A in Fig. 4) is typical of various taxa of Myriapoda (this study) and occurs similarly in Chelicerata [26]. However, the basic pattern found in hexapods (pattern B in Fig. 4) consists of a single gene showing a third putative ligand, the novel trypto-PK (designation adopted from [20]). This type of PK apparently co-evolved in hexapods with an emerging trypto-PK receptor [27]. As we also identified such a trypto-PK in the Remipedia, which are close, or the perhaps closest, relatives of hexapods [13, 28], the evolutionary origin of this ligand likely occurred in a common ancestor of Remipedia + Hexapoda. We found the pattern involving a single gene encoding three putative ligands (whose respective receptors have been verified at least in *D. melanogaster*, see [19, 29]) in all proturans and collembolans and in some species of Archaeognatha (Fig. 4). We identified splice variants of *capa/pk* only in a number of collembolan species. One transcript includes the complete set of PVKs/trypto-PK/PKs, whereas a second transcript encodes only PVKs and trypto-PK (pattern B1). Two separate genes, considered typical of pterygote insects, seem to have “suddenly” appeared in Diplura and Zygentoma, likely as a result of gene duplications of the original *capa/pk* genes (derived pattern C). Hence, according to the phylogenetic relationships of non-pterygote hexapods published by Misof et al. ([1]; Fig. 1), gene duplication and subsequent development of discrete *capa* and *pk* genes occurred at least twice: in Diplura and in Zygentoma. The CAPA precursors of all dipluran species consistently include PVKs and trypto-PK, whereas their PK precursors only comprise PKs (pattern C1). We identified a slightly modified pattern (pattern C2) in three zygentoman species. In these species, the *capa* gene encodes PVK and trypto-PK, as found in Diplura, but the *pk* gene encodes a trypto-PK in addition to the PKs. Thus, both genes have unique ligands (PVKs and PKs, respectively) but share a gene-specific trypto-PK as the third potential ligand. Finally, in a single species of Zygentoma (*T. domestica*), additional PKs are encoded within the *capa* gene (pattern C3), a situation that is typical of many pterygote insects and closely resembles the original *capa/pk* gene of Hexapoda (pattern B). Whether pattern C3 found in *T. domestica* is derived from pattern C2 or directly from pattern B remains unclear. However, peptidome analyses of *capa* products in the American cockroach *P. americana* (Blattodea) and in the red flour beetle *T. castaneum* indicate that the PKs derived from CAPA precursors are not necessarily processed as bioactive peptides, at least not in the neuroendocrine systems [30]. Following gene duplication of the *capa/pk* gene, both novel genes underwent extensive differentiation in different pterygote insect lineages. This differentiation has resulted in divergent expression patterns in different neurons [31], differential processing of CAPA precursors [32], loss of ligands such as PKs in *capa* genes [33], and trypto-PK in *pk* genes [34], and additional genes encoding only trypto-PKs (in locusts, Orthoptera; [20]). Thus, the basis for this differentiation, which for example, encompasses the insect-specific regulation of water balance by CAPA-PVKs (see [29]) , most likely evolved within the non-pterygote hexapods. This indicates that there was obviously considerable evolutionary pressure for the PVKs and PKs to be located in different genes or transcripts. Recent data suggest that two genes also evolved within particular decapod crustaceans, likely independently from the hexapod lineage. In at least the freshwater crayfish *Procambarus clarkii* (Astacoidea), an unusual single-copy PVK-encoding gene is accompanied by a second gene encoding many PKs [35]. On the other hand, a derived pattern is also present in the *D. pulex* genome in the form of a single gene encoding only PVKs [12].

## Conclusions

Our results from analyses of the transcriptome data of a total of 29 species including Protura, Collembola, Diplura, Archaeognatha, and Zygentoma as well as crustaceans and myriapods, reveal the presence of approximately 1,300 neuropeptide/protein hormone precursors. Some of these precursors represent splice variants of a single gene, as is typical of ITP/ITPL and orcokinin A/B precursors. The identified precursor sequences assigned to 39 neuropeptide and protein hormone genes include ACP, AKH, AST-A, AST-C, AST-CC, AT, bursicon-α, bursicon-β, CAPA, CCAP, CCHa1, CCHa2, corazonin, CNMa, CRF-DH, CT-DH, elevenin, EH, ETH, FMRFa, inotocin, kinin, ITP, MIP, MS, natalisin, NPF, NPLP1, orcokinin, PDF, proctolin, PK/PBAN, RYa, SIFa, EFLa, SK, sNPF, TKRP, and trissin. Very few precursors (PDF, elevenin, CNMa) were found to be completely missing in Protura, Collembola or Diplura. For Archaeognatha and Zygentoma (the latter group is the closest relative of all winged insects, the Pterygota), we identified the complete set of neuropeptide precursors. These data confirm that the previously reported absence of particular neuropeptides in some insect lineages, the majority of which are holometabolous insects [36], is an evolutionary trend that must have occurred after the divergence of pterygote insects. The neuropeptide precursor sequences depicted here clearly illustrate evolutionary trends, including numerous modifications of sequences, gene duplications, splice variants, and numbers of paracopies. Specific features cluster within well-described higher systematic groups (Protura, Collembola, Diplura, Archaeognatha, Zygentoma), but, in general, most of these features remain within the limits of variation hitherto known from insects [37]. Some of the predicted mature neuropeptides of collembolans show unusual and characteristic features that place this hexapod lineage in a separate position from the other non-pterygote hexapods. Interestingly, the crustacean *X. tulumensis* (Remipedia), and even *D. pulex* (Branchiopoda), consistently show a more insect-like peptidome.

Many of the predicted mature peptides likely share conserved functions, or at least share conserved ligand/receptor interactions. However, several precursors showed doubtful signal peptides. Cleavage sites are also often not clearly predictable, which is apparently always the case when differential processing of transcripts occurs within different tissues of the same organisms. Therefore, the identification of mature peptides, including their possible posttranslational modifications, in non-pterygote hexapods is the next, and a necessary step to improve our knowledge about the basic pattern of neuropeptides and protein hormones to understand the evolution of such molecules in hexapods.

## Methods

### Ethics and legal statement

Data were obtained from a dataset originally created within the framework of the 1KITE project. All research completed during that study did not involve endangered or protected species and conforms to the provisions of the CITES guidelines. Specimens have been collected and sequenced before October 2014.

### RNA isolation, transcriptome sequencing and assembly

Identified specimens from different arthropod taxa were collected and initially preserved in RNAlater. RNA isolation, cDNA preparation, and transcriptome sequencing were carried out as described in [1]. The assembly of raw RNA-Seq reads was conducted with the program SOAPdenovo-Trans-31 kmer, version 1.01 [38] to achieve a *de novo* assembly of the transcripts. Low-quality reads were removed from the raw data, including 1) reads containing adapter contaminants (≥15 bp aligning with adapter sequences with ≤ 3 mismatches); 2) reads with >10 Ns (unreadable nucleotides); 3) reads with >50 bp of low quality (Phred quality score = 2, ASCII 66 “B”, Illumina 1.5+ Phred + 64). Next, all reads were broken into 31-mers to construct de Bruijn graphs, from which kmers containing Ns were excluded. In the case of particular kmers exhibiting more than 1 out-degree, the out-degrees presenting an abundance of < 10 % of the most abundant one were removed. Thereafter, linear kmers (i.e., kmers with a single out-degree) were merged to form the edge, and different edges were linked with arcs. Arcs showing an abundance of < 5 % of the total out-degrees or < 2 % of the total in-degrees were excluded. Edges with an average abundance ≥ 3 and ≥ 1 were printed out as contigs for assembly version 2 and assembly version 1, respectively. Thereafter, all reads were anchored to contigs of ≥ 100 bp to construct scaffolds using the paired-end information. Finally, all gaps in the scaffolds were filled using Gapcloser in the SOAPdenovo package [39].

### Search algorithms

We analyzed assembled transcript sequences from non-pterygote hexapod species and from *Xilbalbanus* (*Speleonectes*) *tulumensis* (Remipedia), *Anaspides tasmaniae* (Malacostraca), *Lithobius forficatus* (Chilopoda), *Hanseniella* sp. (Symphyla*),* and *Eudigraphis takakuwai nigricans* (Diplopoda) using the tblastn algorithms implemented in the program BioEdit [40]. Our tblastn search was performed using assembly version e1. For all species whose assembly version e1 had been released in the NCBI database (*Acerentomon* sp., *Anurida maritima*, *Tetrodontophora bielanensis*, *Folsomia candida*,* Pogonognathellus* sp.,* Sminthurus viridis*, *Campodea augens*, *Occasjapyx japonicus*, *Machilis hrabei*, *Meinertellus cundinamarcensis*, *Tricholepidion gertschi*, *Thermobia domestica*, *Atelura formicaria*), we updated the corresponding sequences and accession numbers. Additionally, we used the tblastn algorithm implemented in the NCBI database to search for partially missing neuropeptide precursor sequences of *X. tulumensis* (Remipedia; JL) and *F. candida* (Collembola; GAMN). Note that the assembly version e1 was the source for all species; assignments of sequences not yet been released are listed in Additional file 3.

We used sequences of known insect neuropeptides and neuropeptide precursors as queries. Subsequently, we translated all of the hits to the translational level with the ExPASy translate tool ([41], http://web.expasy.org/translate/). Signal peptides were predicted using the SignalP 4.1 server ([42]; www.cbs.dtu.dk/services/SignalP/). Putative cleavage sites of mature peptides were manually assigned based on the criteria of Veenstra [43] and our knowledge of the peptidomes of several insect species. Data from the genome of the fruit fly *D. melanogaster* were acquired from FlyBase (http://flybase.org/), either via direct access using gene names or CG numbers, or indirectly via the use of inbuilt BLAST routines. Annotated *D. melanogaster* polypeptides and their variants were downloaded and compared with the provided GenBank protein accession numbers. For the crustacean branchiopod *D. pulex,* we compared and updated previously published precursor and transcript data [12] using inbuilt BLAST search routines with the most recent gene models in wFleaBase (http://wfleabase.org/) and the JGI-Dappu1-Genome portal (http://genome.jgi.doe.gov/pages/search-for-genes.jsf?organism=Dappu1). The JGI-Dappu1-genome portal provided the more comprehensive and reliable data source. Hence, we updated several *D. pulex* genes (e.g., for the novel *natalisin *gene and several others) in this JGI portal. In Additional file 1, we therefore primarily provide the Dappu1_xxx accession numbers: the corresponding gene models are now essentially free of annotation errors and have carefully been checked for the expressed peptides, as previously identified in part through mass spectrometry [12]. In addition, if correct corresponding sequences were found in the non-redundant GenBank database (NCBI), the respective GenBank accession numbers are provided as well.

### Sequence logo generation

Sequence logos of manually aligned homologous neuropeptide sequences were generated using the tool WebLogo version 2.8.2 ([44]; http://weblogo.berkeley.edu/logo.cgi). Each stack represents one position in the multiple sequence alignment. The overall height of a stack indicates the sequence conservation at this amino acid position: the height of letters within the stack indicates the relative frequency of each amino acid at that particular position. For the color scheme of amino acid residues, the default settings were selected. In addition, the amino acid Cys is colored in orange.

## Availability of data and materials

The complete list of neuropeptide precursor sequences is included as Additional file 1. The respective genomic sequence records which were submitted to NCBI can be found using the GenBank accession numbers as given in Additional file 1.

## Additional files

Additional file 1: List of prepropeptides. List of prepropeptides (precursor sequences) from 24 non-pterygote hexapod species (Protura, Diplura, Collembola, Archaeognatha, Zygentoma), 2 crustacean species and 3 myriapod species; these data are deduced from transcriptome sequence assemblies obtained from the 1KITE project. The majority of prepropeptides contain neuropeptides with known receptors in insects; receptors are not known for the products of the *elevenin*, *efl-amide*, and *orcokinin* genes. In addition, currently available and critically revised sequences from the well-studied water flea *Daphnia pulex* (Branchiopoda) and fruit fly *Drosophila melanogaster* (Diptera) are listed. The genomes of these species were repeatedly screened for neuropeptide genes. Predicted signal peptides (highlighted in grey), amidation signals (bold), cleavage signals (italics, bold), splice variants (a, b), and supposed bioactive mature peptides (underscored) are indicated. Incomplete sequences are indicated with “…”. In some cases, sequences were reconstructed through the fusion of different database entries, or by including sequences from 3'-UTR regions encoding putative coding exons of a different splice form (see ITP). For a few *Thermobia* sequences, PCR and RACE experiments were conducted (Derst C, Bläser M, Predel R; unpublished results) to obtain full-length sequences; this information is given subsequent to accession or JGI numbers. (DOC 1182 kb) Additional file 2: Sequence logos of single-copy peptides. The logos show the sequence variability in the single-copy peptides (<30 amino acids) of non-pterygote hexapod lineages determined using WebLogo version 2.8.2 (http://weblogo.berkeley.edu/). For species with two described genes containing different mature neuropeptides, the more conserved sequence was selected. CNMa was not found in Protura and Collembola, while elevenin was not found in Collembola. Pigment-dispersing factor (PDF) was not found in Protura and Diplura. For comparative purposes, the sequence conservation of CAPA-PVK paracopies was added as an example of multiple-copy peptides. A distinct core sequence is obvious in all sequence logos. The N-terminal sequence motifs are variable in a number of peptides, including SIFa, and this variability is similar to that of the N-termini in the different PVK paracopies. (DOC 460 kb) Additional file 3: Assignment of precursor sequences listed in Additional file 1 (only sequences not released in NCBI yet)*.* The original sequences from assembly version 1 will be available upon request. (XLS 62 kb)
