# Supplementary material for: Evolution of neuropeptides in non-pterygote hexapods
Source: BMC Evol Biol. 2016 Feb 29;16:51. doi: 10.1186/s12862-016-0621-4 (PMC4770511; doi:10.1186/s12862-016-0621-4)
Supplement: Additional file 1: — List of prepropeptides. List of prepropeptides (precursor sequences) from 24 non-pterygote hexapod species (Protura, Diplura, Collembola, Archaeognatha, Zygentoma), 2 crustacean species and 3 myriapod species; these data are deduced from transcriptome sequence assemblies obtained from the 1KITE project. The majority of prepropeptides contain neuropeptides with known receptors in insects; receptors are not known for the products of the elevenin, efl-amide, and orcokinin genes. In addition, currently available and critically revised sequences from the well-studied water flea Daphnia pulex (Branchiopoda) and fruit fly Drosophila melanogaster (Diptera) are listed. The genomes of these species were repeatedly screened for neuropeptide genes. Predicted signal peptides (highlighted in grey), amidation signals (bold), cleavage signals (italics, bold), splice variants (a, b), and supposed bioactive mature peptides (underscored) are indicated. Incomplete sequences are indicated with “…”. In some cases, sequences were reconstructed through the fusion of different database entries, or by including sequences from 3'-UTR regions encoding putative coding exons of a different splice form (see ITP). For a few Thermobia sequences, PCR and RACE experiments were conducted (Derst C, Bläser M, Predel R; unpublished results) to obtain full-length sequences; this information is given subsequent to accession or JGI numbers. (DOC 1182 kb) [file 12862_2016_621_MOESM1_ESM.doc]

**Additional file 1** (additional file 1.doc) *List of prepropeptides*. List of prepropeptides from 24 apterygote hexapod species (Protura, Diplura, Collembola, Archaeognatha, Zygentoma), 2 crustacean species and 3 myriapod species; these data are deduced from transcriptome sequence assemblies obtained from the 1KITE project. The majority of prepropeptides contain neuropeptides with known receptors in insects; receptors are not known for the products of the *elevenin*, *efl-amide*, and *orcokinin* genes. In addition, currently available and critically revised sequences from the well-studied water flea *Daphnia pulex* (Branchiopoda) and fruit fly *Drosophila melanogaster* (Diptera) are listed. The genomes of these species were repeatedly screened for neuropeptide genes. Predicted signal peptides (highlighted in grey), amidation signals (bold), cleavage signals (italics, bold), splice variants (a, b), and supposed bioactive mature peptides (underscored) are indicated. Incomplete sequences are indicated with "…". In some cases, sequences were reconstructed through the fusion of different database entries, or by including sequences from 3'-UTR regions encoding putative coding exons of a different splice form (see ITP). For a few *Thermobia* sequences, PCR and RACE experiments were conducted (Derst C, Bläser M, Predel R; unpublished results) to obtain full-length sequences; this information is given subsequent to accession or JGI numbers.

Adipokinetic hormone corazonin-like peptide (ACP)

>*Nipponentomon nippon* (Protura) ACP1

…SLSSSSQIVLVSFLTASLFISCWCFNSAAGQVTFSRDWSA**G*KR***STYPSHRCQPGRDLLSAVRQLIMHEARKMRNCDMITSMTLEPEEAVGRKTQPW***RR***GADDKTEIPGEDGNSGFVVRNEDQHGPSTP*

>*Nipponentomon nippon* (Protura) ACP2

***M***SQVKNCHWTIILVSLGGIGLFLIHQASSQVTFSRDWSA**G*KR***SSSIVSPPKCHGDQGLLAAVKQLILHEA***RR***MVDCDRLASLRGQQEYEEV***RR***GDDRMDAGPPEIPTTIDDALSSGFVIRQPKQDRNAP*

>*Acerentomon* sp. (Protura) ACP1 [GenBank:GAXE01015046.1]

…STYSARGVILVTGATVCVIICSCFNLCSAQVTFSRDWSA**G*KR***SSYLPHRCQPSADLVTAVRQLIMHEARKMSNCNTISLNLEEEAIAR***KR***DLP***RR***QPDDKQDSEIPTLDEVVNSGFVIHRGNDENGQRGTAR*

>*Acerentomon* sp. (Protura) ACP2 [GenBank:GAXE01015047.1]

…STYSARGVILVTGATVCVIICSCFNLCAAQVTFSRDWSA**G*KR***SSYLPHRCQPSADLVTAVRQLIMHEARKMSNCNTISLNLEEEAIAR***KR***DLP***RR***QPDDKQDSEIPTLDEVVNSGFVIHRGNDENGQRGTAR*

>*Filientomon takanawanum* (Protura) ACP

***M***MPVRSTLSYLLQVGLVSLLTASLLMSCWCVHSAAGQVTFSRDWSA**G*KR***SASPSHRCQPGKDLVNAVRQLIMHEARKMRNCDMLTSMTLEPEDPSGRKTQPW***RR***GADDKTEVAIEDGNSGFIVGTLSDDNHQGPSLP*

>*Anurida maritima* (Collembola) ACP [GenBank:GAUE01039625.1]

***M***FRLLSVFLIIQLSLIMTFAQITFTREWTG**G*KR***SAPTSPAV***RR***GNCVSFMPTINFLMQ…

>*Tetrodontophora bielanensis* (Collembola)ACP [GenBank:GAXI01057634.1]

***M***KQFFFIFLIIQTTFLITLAQITFTREWVT**G*KR***SSGVPSRLIIKENTRGQKLLPAIEYLMKNENLKKVGYGYTD…

>*Podura aquatica* (Collembola) ACP

…FSNAQITFSREWVG**G*KR***SAPISAFNSNLAHHAAEGCMSFMHTIQFLLKIE*

>*Folsomia candida* (Collembola) ACP [GenBank:GASX01085389.1]

***M***KYIFSIFLVVQLSLFAASMAQITFTREWVT**G*KR***SAAPKLRVLMHDDNICKSLLPAIDYLLEVEKIHQKLCGPPPKSYSVPVMLKKLTPVSDVLSSSSSGDQGYDDGYDGVASSHMQKST*

>*Orchesella cincta* (Collembola) ACP

***M***KAVVCFLLFFYFASSIAQITFTREWVK**G*KR***STTANTAAAAKDGHSVCQNLVTAVEYLIELEKL***RR***AECLECCHSAPPAFPDLPDIQRSPSDFMQFFRKLPPVEQEQMVRVVPSQESRNQ*

>*Pogonognathellus sp.* (Collembola) ACP [GenBank:GATD01025898.1]

…ITFTREWVT**G*KR***SLPPTRKVIVKEEHAWCSRSFVPVIEYLVEEERVRKHECGSHLNVNIPKSVSWIRPAIIDELP…

>*Sminthurus viridis* (Collembola) ACP [GenBank:GATZ01092399.1]

***M***RSLICILLAIQLSISLVVAQITFTREWVN**G*KR***NSPSSIFTPQNSQDYLPCKSLWPAIEILMETERLQQAHCGIRLRPAKYMAHFKDPLTGAINKLQDEESVQFEED…

>*Campodea augens* (Diplura) ACP [GenBank:GAYN01117863.1]

…FFCVLCLRSYTADAQVTFSRDWQA**G*KR***SSDFSDSCEVPHMAFFKSI***RR***LIGTESSRIASCKMQLFINGFPSLSNDVIEAAHALMKTNDGAQS*

>*Lepidocampa weberi* (Diplura) ACP

***M***ISSHHIMIISFVLIFTISVFHTANAQVTFSRDWQA**G*KR***SLETGENCEIPKLAFFKAV***RR***LLGSESSRMAACKMQAFLTDIPSLNNDDVIDSAHSLMKVHPDRQP*

>*Pedetontus okajimae* (Archaeognatha) ACP

***M***ALYRINTFLVTVCIAATLLGLAFQETASQVTFSRDWTA**G*KR***SPITSSELTSSCNLLLREVQKLAKAEI***RR***ITSCEMKSFLKGDKKLESVSDLVEDDLDTIYSART***RR****

>*Machilis hrabei* (Archaeognatha) ACP [GenBank:GAUM01120457.1]

…HETSSQVTFSRDWTA**G*KR***ASLSPNQLTSSCNMLLHEVQRLAKAEI***RR***MTSCEMKTLLKGDKKFDGITDLVEDDLETIYSTRTR…

>*Meinertellus cundinamarcensis* (Archaeognatha) ACP [GenBank:GAUG01190983.1]

***M***SLSYRLNSALVTICVFVTILSLVCHQTSAQVTFSRDWTA**G*KR***SSTGISALPSSCKLLLREVHKLAIAEI***RR***QS…

>*Machilontus* sp. (Archaeognatha) ACP

…***R***LAACESKLTFKGDKIVDAADLSEDDLESAAAYQSHA***RR****

>*Tricholepidion gertschi* (Zygentoma) ACP [GenBank:GASO01199980.1]

…FCVVAAFLVLSVIYKVDAQVTFSRDWTA**G*KR***SASDLDCNVLVKSALVKLQKLVTNELRLVSACEMGSLIHAVKGGDRLNMLEYRDESLDTPGYIPANQN…

>*Thermobia domestica* (Zygentoma) ACP [GenBank:GASN01384281.1]

…SPIVLLVIICIYGSADGQVTFSRDWTA**G*KR***SALNERGECFADARAVLHRVEKILTNEFREMAGCEVRSLLRASEGGPKINPAEFPPPVEELENTGLFSQHS***RR****

>*Atelura formicaria* (Zygentoma) ACP1 [GenBank:GAYJ01000651.1]

***M***SMTPRLSLSIFFLGLLLVVMSCMSHSADAQVTFSRDWTA**G*KR***SLGGQEECASDLRAVLVKIQRLITNELRQMYACVLKSPLNKAGDRVKLEFAEGGVGDLEGSAFFPAPS***RR****

>*Atelura formicaria* (Zygentoma) ACP2 [GenBank:GAYJ01000652.1]

***M***SMTPRLSLSIFFLGLLLVVMSCMSHSADAQVTFSRDWTA**G*KR***SLGGQEECASDLRAVLIKIQRLVTNELRQMYACVLKSPLNKGGDRVKLEFAEGGVGDLEGSAFFPAPS***RR****

>*Nicoletia phytophila* (Zygentoma) ACP

***M***VTRFSLRMPVSCVLVAILLPCTFLCSDAQVTFSRDWTA**G*KR***SEGGEVSGCLSVLRPVLGKIQRIVTDELRHIAACEVKSLLHKEGDKLKVEYHDTGVEDLDNSAFYPVAT***RR****

..............................................................................................

>*Xibalbanus tulumensis* (Remipedia)ACP [GenBank:JL122470.1](Christie 2014)

***M***NQRLDRVYPAVMGSFLISLVLLSAFTQMTVAQVTFSRDWNA*G****KR***SGAAALYPDCAMPERALLSEVSKLIHNEAQRMVSCQAWTVLR**G***

>*Hanseniella* sp. (Symphyla) ACP

…SRGQVTFSKDWKA**G*KR***AVTEGCSINTIYLV***RR***LHRIVKTELDHLEDCGYGSMIEDLPRFE*

>*Eudigraphis takakuwai nigricans* (Diplopoda) ACP1

***M***VAIRSIPTLVMLVALCSLPLLAHSQVTFSRDWSAA**G*KR***ASVVTDCHAAVTRLLPQMAYVGEVCSPHQAVEPAFATRK*

>*Eudigraphis takakuwai nigricans* (Diplopoda) ACP2

***M***AKLHCPVLAVALLFVTLLLLLAPSTGQLTFSRGWLPS**G*KR***SGDCHAALARLMPDLSYLSEVCNLRPYDDM*

..............................................................................................

no ACP known from *D. melanogaster* and *D. pulex*

Adipokinetic hormone (AKH)

>*Nipponentomon nippon* (Protura) AKH1

***M***NFASHRSLLVLLVVVLGHLCLIHGQVNFSPSW**G*KR***GPPANPAQEQPGPNVCKTSMDALMNLYRIIQAEAQRLRDCQQFER*

>*Nipponentomon nippon* (Protura) AKH2

***M***SPSMSRCLMVLFAIVLVHLCVIQGQVNFSPSW**G*KR***GPPSNAVQDGNPNACKTSMDTLMNLYRIIQAEAQRLRECQQFER*

>*Acerentomon* sp. (Protura) AKH [GenBank:GAXE01132055.1]

***M***SPSTPRSLLVVLALVLAHICLIHAQVNFSPSW**G*KR***GPPSNPAQDNGPNACKTSMDALMNLYRIIQTEAQRLRDCQQFER*

>*Filientomon takanawanum* (Protura) AKH

***M***NFASHRSLLVLFAVVLGHLCLIHGQVNFSPSW**G*KR***GPPSNPAQEQPGPNTCKTSMDALMNLYRIIQVEAQRLRDCQQFER*

>*Podura aquatica* (Collembola) AKH

***M***ISQKALITLMLVIFTSGEILGQVNFSPSW**G*KR***GANFLASNSNPNSPINIKEAQNEFINPLNSPNDNCKP…

>*Folsomia candida* (Collembola) AKH [GenBank:GASX01059173.1]

***M***KSNNVTTTLLWGAFLFLAVVALTVQAQVNFSPAW**G*KR***GHSIPVSVQSRNGGDATLGSDHLQ…

>*Bourletiella hortensis* (Collembola) AKH

***M***ALPKTAISIALLVIIAISSIHYTYAQVNFSPSW**G*KR***TAAIMSNPNTEEMNTITENCKVPLDSILRIQKLIQLEAHKLIRCESMAGEMQ***RR***K*

>*Orchesella cincta* (Collembola) AKH

***M***RSVFIMVALLYTFFTFTLIQADAQVNFSPAW**G*KR***STTDWLGESQDPTDYRKVVGIDGCQTPLECLLRMQRYPYVTPERAARLTNAKMLKTLARGDEKFD*

>*Pogonognathellus sp.* (Collembola) AKH [GenBank:GATD01008212.1]

***M***RSLSLATLFVGVVVFFIVADAQVNFSPSW**G*KR***TSGILSDSSSSDTELPQFSDNCKIPLDSIVRIQRMIQLEARHILKCEAMNVSKK*

>*Sminthurus viridis* (Collembola) AKH [GenBank:GATZ01006795.1]

***M***SQIRAGFYITLVVAIAFLAATVVEAQVNFSPSW**G*KR***ASVAMSQGGAEEFSQFPENCKIPLDSVVRIQKLIQVNSTGLEKSRVFTFTMFSLLLILLKDHICIYLSATLVGGPQTHAL*

>*Jordanathrix leptothrix* (Collembola) AKH

***M***TQMRAAMYFTLIILGLALVSVTIVEAQVNFSPSWG**G*KR***AISVLANNGAPDDLAQFSENCKIPIDSIIRIQKLIQVEANKLIRCETISGDVQKIK*

>*Campodea augens* (Diplura) AKH [GenBank:GAYN01018463.1]

***M***NRLIAALAAILVLMTLTSAQVNFSPSW**G*KR***ADPTEGCKVSLDTVMHVYKLIQNEAQKLLECEKFIK*

>*Lepidocampa weberi* (Diplura) AKH

***M***RPYLAALTAILLLVTLTSAQVNFSPSW**G*KR***ADTTEGCKPSLDAVMYIYKLIQSEAQRLLECEKYIK*

>*Occasjapyx japonicus* (Diplura) AKH [GenBank:GAXJ01103901.1]

***M***SRMGASSRHHQLSLALCCSVLLLALMVLQQAPTTSAQVNFSPSW**G*KR***TAAEVGGSTGGGGGGGGVSAAAAAGSIASCRTSMDNILYVYKLIQNEAQRLVECDKYLK*

>*Catajapyx aquilonaris* (Diplura) AKH

***M***AVLAPLVCSLLLLSSLASAQVNFSPSW**G*KR***SSESSSSNGVSADAACRNSMDNILYIYKLVQNEAQRFLECQKYLK*

>*Pedetontus okajimae* (Archaeognatha) AKH

***M***TQRLTLAVFVAIALIFACLLFDVTSGQVNFSPNW**G*KR***APASADGCKGSVDAVMYIYKLIQGEAQKLLECEKFSK*

>*Machilis hrabei* (Archaeognatha) AKH [GenBank:GAUM01163553.1]

***M***ALR***M***TLAVFVVISAILVCLLVDVTSGQVNFSPNW**G*KR***ASTGVDGCKGSVDTVMYIYKLIQGEAQKLLECEKFSK*

>*Meinertellus cundinamarcensis* (Archaeognatha) AKH [GenBank:GAUG01236146.1]

***M***SQRQTFTLLFVATILVCFVVHFTSGQVNFSPSW**G*KR***QVGEVDSCKISIDYVMNIYKLIQNEAERLMECRKLSN*

>*Machilontus* sp. (Archaeognatha) AKH

***M***SQRSALALFLVATIFVCFLVNVTSGQVNFSPSW**G*KR***GAGNGNAAEGCKFSVDSVMNIYKLVQSEAQRILDCEKFSN*

>*Tricholepidion gertschi* (Zygentoma) AKH [GenBank:GASO01224229.1]

***M***SRLMLVVLALSTLMMMLMIDSTSAQVNFTPSW**G*KR***GASPQDGCKASVDSLMYIYKLIQSEAQKIVDCEKFVN*

>*Thermobia domestica* (Zygentoma) AKH [GenBank:GASN01024159.1]

***M***SRTLLAALFLATILLALFVDTTTAQVNFSPNW**G*KR***GGPASDGCKASMDSVMYIYKLIQNEAQKIVDCEKFGN*

>*Nicoletia phytophila* (Zygentoma) AKH

***M***SRHVMIMLVVAAVLLVFLDLASAQLNFSPSW**G*KR***GGPTQEGCKASMDSLMYIYKLIQTEAQKIAECEKFGN*

..............................................................................................

>*Lithobius forficatus* (Chilopoda) AKH

***M***KQGTFLSLLVVAVIVLVAVDFTSAQVNFSPGWGQ**G*KR***SSNSDTCAVCAQTMLQVYRLLQSLEERLGGSRNLSR*

>*Hanseniella* sp. (Symphyla) AKH

***M***RAWVSLLSLTLMVLLLMGTAHAQVNFSPNW**G*KR***GDPPCAVSKDTVLTIYSLIEAEARRLNECGSNFN…

>*Eudigraphis takakuwai nigricans* (Diplopoda) AKH

***M***RPILLSVLLLSGLVVLLLTPDTSAQVNFSPGW**G*KR***FDIGEAGANPNCKPSVESIVEVYRLIQMEAQRVRQCEQ***KR****

..............................................................................................

>*Drosophila melanogaster* AKH [[FBpp0073069](http://flybase.org/reports/FBpp0073069.html)]

***M***NPKSEVLIAAVLFMLLACVQCQLTFSPDW**G*KR***SVGGAGPGTFFETQQGN**C**KTSNEMLLEIFRFVQSQAQLFLDCKHRE*

>*Daphnia pulex* AKH [[Dappu1_114396](http://genome.jgi.doe.gov/cgi-bin/dispGeneModel?db=Dappu1&tid=114396), GenBank:[EFX68649.1](http://www.ncbi.nlm.nih.gov/protein/321457565?report=genbank&log$=prottop&blast_rank=1&RID=XHCC8MHF014)]

***M***ANHRILILTLLMIGLASAQVNFSTSW**G*KR***SPSTSTKAAEPPSAPSYRQNFHSKKVEPGTLETLPNNQHLPESFDTVSSTIYDDAEEQRISISLPSPCLSILKSLLLVNQIVEFKNSPLDGRMHRFKIENLFPLPNRTCRLYI***RR****

Allatostatin A (AST A)

>*Nipponentomon nippon* (Protura) AST A

**M**SGAVKAAFSLIPCYILLALMLQCSSKALADQTDDNDIDQSIDNL**KR**QYSFGL**GKR**PASSYDD**KR**VPSYNFGL**GKR**SISDEDIDYSNPEYS**KR**ANPNMYAFGL**GKR**LSSSRYAIGL**GKR**PNELYAFGL**GKR**SGGASASDGGNELDWDDETNEDAWIEAETGGPP**GKR**RSYEFGL**GKR**QSPNRMYDFGI**GKR**RQYDFGI**GKR**RSYEFSL**GKR**RQYEFGL**GKR**RQYEFGL**GKR**RSYEFGL**GKR**PQQAYSFGL**GKR**GQQSYSFGL**GKR**ISADDDSTSGLDARDDLDQEPNLDAVSNMAALFHNPSSMMQRHPGFSESDSGSQQHSDD**KR**RDHRFSMGL**GKR**EVNIDEMPTVGATNLNLKKTTEEPQQQQQHDEGKSNSKENSQSKPDQGI***RR***MI*

>*Acerentomon* sp. (Protura) AST A [GenBank:GAXE01139630.1]

***M***KLGTNRLIRLVSGSAVAILVLHCVCLTKADDYSETSEDELDQSIDNL***KR***QYNFGL**G*KR***SPVFPAPNY***KR***VQSYNFGL**G*KR***SNEEDLSSDNS***KR***ANTNQYAFGL**G*KR***LSSLYSIGL**G*KR***PNELYAFGL**G*KR***SSGFDPDISEGLDLEYLEEEEPKPRDSRPAE***KR***RMYEFRI**G*KR***ATPEKMYGFGI**G*KR***RSYEFGL**G*KR***KPYEFGL**G*KR***RSYEFGL**G*KR***RTYEFGL**G*KR***RPYEFGL**G*KR***PAGRQAYEFGL**G*KR***LPQSYSFGL**G*KR***ENQQGYAFGL**G*KR***ISDLLDDSEPNFNTKTFFEDSSPSLNPVLSSSSSIESEKSDD***KR***KDHRFSMGL**G*KR***EVDMNELKAEDDTNQTKYQSETKSAAENATQSLRNGGIRKMI*

>*Filientomon takanawanum* (Protura) AST A

***M***GTKSSTKTVFQFVSKCVVAILVLHCVSLCTADETDESDDELDQSIDNL***KR***QYNFGL**G*KR***EQDTSYEGPTF***KR***AQSYNFGL**G*KR***SDEEDIGSDNS***KR***GGHNQYAFGL**G*KR***LSHMYSIGL**G*KR***PNEMYAFGL**G*KR***SGQVDHESEETDFNDVDDDETSMDNYYNGKPAE***KR***RMYEFGL**G*KR***AAPDRMYGFGI**G*KR***RQYEFGL**G*KR***RSYEFGL**G*KR***RSYEFGL**G*KR***PQSYQFGL**G*KR***LPQSYSFGL**G*KR***DNTRSYAFGL**G*KR***NTDLDAEAESNLNLERIYYQDNIMPLLQPGLALSGSDATQSEAD***KR***RDHRFSMGL**G*KR***EANIDELVSADDNTRKYQSDPNSTANPMKTQSQSDKNLGIRKMI*

>*Anurida maritima* (Collembola) AST A [GenBank:GAUE01053897.1]

***M***QRILSSPTFLLMFLCSYAWTSQFPSADEIDSV***RR***LVLYGSSP***RR***FQLFEY**G*RR***PFEEDRESLEDFSSIPSTNIDYYNDLD***KR***VVGNGNKYTFGL**G*KR***SKSYGFGL**G*KR***AKAYGFGL**G*KR***LQKDWKNSPALLSPNYRQE***RR***SKVYSFGL**G*KR***SAGNSLDLDKVQEGYYEGQEGPEKSARLFSVGPGKMEVNAGTS*

>*Tetrodontophora bielanensis* (Collembola) AST A [GenBank:GAXI01020132.1]

***M***QRIGSLLMSHMLVILFVGTSYAWSRPESLGDDYDSM***KR***LMYSYGTY***KR***LPSSYDFGL***KK***SMEEPSSYEDMSYSPEGYSNSMD***KR***APANMYSFGL**G*KR***NRAYGFGL**G*KR***LEQAWKIPSTSLHPSYRQE***RR***SKIYSFGLGKRQSPDLLEDDDDQLTD***KR***ASGGRQFNFGL**G*KR***EVDSIPLVKNTTS*

>*Podura aquatica* (Collembola) AST A

***M***MHRIGLVSPLVLLLVVGSSAWSSSGDSPYFSLLRTTGRGVDDLMRKSYEEDEAPDYYYSYGD***KR***AAQPLYSFGL**G*KR***NRAYGFGL**G*KR***LEEQWKNSATLHPTYRQE***RR***SKVYSFGL**G*KR***GFETIPESGDDYVEDIKEAE***KK***SRQFSFGL**G*KR***ESGDVNSKGLSSS*

>*Folsomia candida* (Collembola) AST A [GenBank:GASX01008840.1 + GASX01012811.1]

***M***TTAAPQVILLVLASCACVWAAQMSDNYEKMRLLYNYPSGF***KR***LPSYDFGL**G*KR***SFG…

…RQE***RR***SKIYSFGL**G*KR***QSHDGMEDYDGNASPPTAE***RR***ASSQRQFAFGL**G*KR***DLNAAKE*

>*Bourletiella hortensis* (Collembola) AST A

***M***QKLAIVLTAHVLIVLCVSSSAWSRQISDDSDLM***KR***MYSYASQLQL***KR***PMYDFGL**G*KR***SLEEAASYEDLSNLPLDAYNVED***KR***GAGPNSMYSFGL**G*KR***NRAYGFGL**G*KR***LEAQWKYPSARQE***RR***SKIYSFGL**G*KR***SGAGPMYNGLEDYYDMSAIQAPAAAAAAD***KR***AAGGRQFSFGL**G*KR***DVADASHNTTS*

>*Orchesella cincta* (Collembola) AST A

…***KR***SLEEPASYEDTGYPYPLDAYNNYAVE***KR***ASPSGMYSFGL**G*KR***NQRVYGFGL**G*KR***LEQAWRGVNAPSASIFPSSRQE***RR***SKIYSFGL**G*KR***QSPDLQEDDYEFPAAASANSD***KR***SSQARQFSFGL**G*KR***DTEASDHVKTTS*

>*Pogonognathellus sp.* (Collembola) AST A [GenBank:GATD01099731.1]

***M***QRLGNILAYQLLMLLLVESYAWPREMHEDYDTKGLFPAELQSNRFPNYELYP**G*KR***SGSAKDEVVSYEDFTSPSLVDTLNGID***KR***VSPNMYSFGL**G*KK***NRAYGFGL**G*KR***FEHNMWKTYPASSPSFRQERSSKIYSFGL**G*KR***GVNYDDQGRSSVSGQRQFSFGL**G*KR***ETQQSTSAAN*

>*Sminthurus viridis* (Collembola) AST A [GenBank:GATZ01018538.1]

***M***QRISSLLASQVLVILFVGSYAWSRQADESDLM***KR***MYSIASQY***KR***PMYDFGL**G*KR***SWDEPASDEDLASALDTYNVED***KR***GAPNSMYSFGL**G*KR***NRAYGFGL**G*KR***LENPWKYSPVSANRQE***RR***SKIYSFGL**G*KR***SSLPISSGSDDYYDNAGAD***KR***STANRQFSFGL**G*KR***DVSEASHVNSTS*

>Jordanathrix leptothrix (Collembola) AST A

…ASQVLVLLFVGSYAWPRQVPDDADLI***KR***MYSYASQF***KR***PMYDFGL**G*KR***SLEDQESFESSDLSAPALDAYNI…

>*Campodea augens* (Diplura) AST A[GenBank:GAYN01140363.1]

…FGL**G*KR***PQSMYSFGL**G*KR***YTPSVYAM**G*KR***PKEMYAFGL**G*KR***SADDYYDNSIEDNGLYDGDMD***KR***KYAFGL**G*KR***PMNYNFGL**G*KR***PMSYNFGL**G*KR***PMNYNFGL**G*KR***SKNMYSFGL**G*KR***EVDNDQFVKSDEGGNVEATEKSSVVEEAGKPNELQHERT***KR***AAPSGLYSFGL**G*KR***RPFDFGL**G*KR***NRMYSFGV**G*KR***DYEEVEDENLYE*

>*Lepidocampa weberi* (Diplura) AST A

***M***HYGSWMSGSSSFLLCLFSLVVLVTLTRCQDADVDLSADPYYEPSIDDLAAVD***KR***PSAYNFGL**G*KR***AGNYNFGL**G*KR***PNSMYSFGL**G*KR***YNPYYEM**G*KR***PKEMYAFGL**G*KR***SADDYYDSIEDQDGGAWVDEVD***KR***AGKYAFGL**G*KR***PMSYNFGL**G*KR***PMSYNFGL**G*KR***SRNMYSFGL**G*KR***EVSQEQYGSKNDVTTTEKPSGETQEKHDRK***KR***ASGMYSFGL**G*KR***RPYDFGL**G*KR***PRYSFGV**G*KR***DYEEPELEGTYE*

>*Occasjapyx japonicus* (Diplura) AST A1 [GenBank:GAXJ01108915.1 + GAXJ01107315)

***M***LPGGCRSLLMLAVWLYWSSVLLVTGAPSSSSSASSSSSSPASPSSAAALVHVPLELVEDDPEDDLDLD***KR***AYSYVSSY***KR***LPVYNFGL**G*KR***GKMAYSFGL**G*KR***PQQMYSFGL**G*KR***INPM**G*KR***PKEMYSFGL**G*KR***DASEYDDGRDWELVDLPEQEEGEEQEDGEGEAPEGGEEEEEEEGEVDEPE**G*KR***AQGMYSFGL**G*KR***RPYSFGL**G*KR***TQGMYSFGL**G*KR***APMYGFGL**G*KR***ERMYGFGL**G*KR***ERMYGFGL**G*KR***PDGNSRQRFAFGL**G*KR***TATEDESPEEEV***KR***EV***KR***SELEAVRKEGAAAAAGSLEKPAPSANQQPNADAESKV***KR***ALYSFGL**G*KR***AYDFGL**G*KR***PKQYSFGI**G*KK***SLEEEEEADEDRVGMSQ*

>*Occasjapyx japonicus* (Diplura) AST A2 [GenBank:GAXJ01074849.1 + GAXJ01075325.1]

…GPYSFGL**G*KR***APLYSFGL**G*KR***ASGGRQYSFGL**G*KR***LNNNHRQYSFGL**G*KR***EVSASEAAAVAAEAAAAEEHVV***KK***RSANPEQLEWADAETSRSDEEQMSSVPGPAEFGRA**G*RR***TYSFGL**G*KR***LPLYSFGV**G*KR***LSA*

>*Catajapyx aquilonaris* (Diplura) AST A

***M***PGAMIPAGCRSLLLLSVWLYWSSVVVSSVPSPTSSASSVLPVELMDDPDDDLQQLPYPSDLD***KR***AYSYVSSY***KR***LPVYNFGL**G*KR***GNKMAYSFGL**G*KR***PQMYSFGL**G*KR***VNQDYGYPAM**G*KR***PKEMYSFGL**G*KR***DTSEYEDWDDNNSQEED***KR***APKYAFGL**G*KR***RAYSFGL**G*KR***SPQMYSFGL**G*KR***APMYGFGL**G*KR***ERMYGFGL**G*KR***PDYNSQRFSFGL**G*KR***SMEEEDEEDAED***KR***EVNPSEMEEVRQEADNQAEV***KR***RSPYSFGL**G*KK***AYDFGL**G*KR***PKQYSFGM**G*KK***SEGMSQ*

>*Pedetontus okajimae* (Archaeognatha) AST A

***M***GRTNRHLTQFQSTAVIVFLIFGVSIATTLDEWQADEFSESSLEDEDSTLDAY***KR***LYDFGV**G*KK***SYLYED***KR***MPAYNFGL**G*KR***APSYNFGL**G*KR***LKNGKIVYPYGPGNPTNRYNLGL**G*KR***PSHLYSFGL**G*KR***SGSDMGFSDDEYEYDMPVEDGKQEDE***KR***SKMYNFGL**G*KR***DRLYSFGL**G*KR***TPNMYSFGL**G*KR***APKYSFGL**G*KK***SDSRLYSFGL**G*KR***SAPYERFNFGL**G*KR***EYDTSYEDYGDEE***KR***SGHRYSFGL**G*KR***EVDSSEQRYIQEQEEERNHDNSNELSAD***KK***SRAYNFGL**G*KK***NFILQNAKNESKHSETKQAAVPHHGNRE***KR***STPQYSFGL**G*KR***DGFENSIEAMQYAEDPEYNSVEAPSTYRVA***KK***NKMYSFGL**G*RR***SGGGQQIPLYGFGI**G*KR***STDDASEVH*

>*Machilis hrabei* (Archaeognatha) AST A [GenBank:GAUM01012727.1 + GAUM01078271.1 + GAUM01025621.1]

***M***GWSNRHLSQCQTTFAVLFLIFGVCIAAASDEWQSDEFPESSLAGEDDSAMDAY***KR***LYDFGV**G*K***…

…NFGL**G*KR***VPSYNFGL**G*KR***LRNGKVIYPFGPANPSDRYGQGL**G*KR***PSQLYSFGL**G*KR***SSNDMDLSDDEYEFDMPSEESDQ…

…SFGL**G*KR***TPNMYSFGL**G*KR***TPMYSFGL**G*KK***SDGRLYSFGL**G*KR***SAPYQKFNFGL**G*KR***EYDNGEEDYEDEE***KR***S**G*RR***YSFGL**G*KR***EVDSSEQRYILEEEQRNHDNAKEFADE***KR***SREYNFGL***GKK***DTIPEHVKNQSTNSQSKEKDTRLHGARE***KR***STSRYSFGL**G*KR***DDMDNAIEAMQYEEDPEFSSFEGPSTYRLD***KK***NRLYSFGL**G*RR***SGGGRQIPLYGFGI**G*KR***STDVNEANLEVH*

>*Meinertellus cundinamarcensis* (Archaeognatha) AST A [GenBank:GAUG01035062.1]

***M***GLLSKHHIQIRASLSVLLVLLCLSTTAAVDDWESEIVPESSIEDDDNSIEEY***KR***LYNFGL**G*KR***SYSNEY***KR***LPVYNFGL**G*KR***GHAYSFGL**G*KR***AKSGSSSMYSFGL**G*KR***PNSNKYAFGL**G*KR***PSQLYSFGL**G*KR***SSDDSEFSDDDYDIEASLDYGTPED***KR***GKAYSFGL**G*KR***DRSYNFGL**G*KR***APSMYSFNFGL**G*KR***DYDEELDEIADEV***KR***SNGHRFAFGL**G*KR***EVDASEMKDLNAELSQNVAADIKHETE***KR***AAYNFGL**G*KK***SVEAHHEKNHTTDEEVQSSQTSHHHGHLV***KR***SPSPYNFGL**G*KR***DDVDDDVEFDEPEVFEKPSTYLMD***KR***SRASAFGFGRSSDGRQMPLYGFGI**G*KR***STSNEDSLEVVH*

>*Machilontus* sp. (Archaeognatha) AST A

***M***GNYRHHIAFPASLTALLMIFLVSATTATDDWPNEEIPETSMEDEDNSLEAY***KR***LYDFGL**G*KR***SSYSNEY***KR***LPIYNFGL**G*KR***APMYSFGL**G*KR***SKSGSSMYSFGL**G*KR***PNGNSKYSFGL**G*KR***PSQLYSFGL**G*KR***SSDDTMFSDEEEYDMDNDYDTSEE***KR***SRQYSFGL**G*KR***ERMYNFGL**G*KR***SPSSMYSFGL**G*KR***SRTYSFGL**G*KR***PSDRLYSFGL**G*KR***SDG***RR***YNFGL**G*KR***DYDEDVEDLDSEV***KR***SNGHRFSFGL**G*KR***EVDPSDISDIRAEAERMNAENEETHNTE***KR***AVYSFGL**G*KK***SEVSEPGKNKSSENEQGSEDTHNHKQPV***KR***SPSPYAFGL**G*KR***DDIDNLDNIDFEESDPIERPSTYLMD***KR***SRPYSFGL**G*KR***SGYGRQIPLYGFGI**G*KR***SITSSENVH*

>*Tricholepidion gertschi* (Zygentoma) AST A [GenBank:GASO01229841.1 + GASO01255089.1]

***M***HWPVFHRLLLCLVVYITFLLLGITTAEDASVALPDSDVDVDEELY***KR***MYDFGL**G*KR***AYSYVSEY***KR***LPVYNFGL**G*KR***STGMYSFGL**G*KR***SPSRESSKLYSFGL**G*KR***TRPSQMYSFPQT***KR***VKL…

…***KR***NRVYSFGL**G*KR***AKAYSFGL**G*KR***LPQKMYSFGL**G*KR***VPMYSFGL**G*KR***SGGRPYSFGL**G*KR***PMSPKQSSSRFNFGL**G*KR***DAGYPYFYDEESDDGAEDLIHEEAD***KR***GEQRFSFGL**G*KR***EVTPQEINAFQQELNHHYDDGEKSMNHED***KR***SKAYSFGL**G*KK***DSSPGNENDTRDDKSNQSQHPGGVV***KR***SPHYSFGL**G*KR***DDEEDQDVSDDWAEDISIAEDNNDD***KR***NNHRYKFIGL**G*KK***TPGYEYSRP**GRR**TYSFGL**G*KR***LPMYDFGI**G*KR***STDSSAIH*

>*Thermobia domestica* (Zygentoma) AST A [GenBank:GASN01396887.1 + GASN01407427.1]

***M***LSSFKPLDNMSSFKCWIFVFVVFILSVMAEDLETSGASREVPVDDIDEATLDLY***KR***LYNFGL**G*KR***AYTSLSEY***KR***LPVYNFGL**G*KR***SPGLYSFGL**G*KR***SPPSNLYSFGL**G*KR***VRPSEMYSFGL**G*KR***SWSYEDDYPEEENNQEEYDDDTEEE***KR***NNRLYSFGL**G*KR***ARSFNFGL**G*KR***DMQRLYGFGL**G*KK***SGMYDFGL**G*KR***GMRDYAFGL**G*KR***PVPRLSNSRFNFGL**G*KR***DPGYSDGDQFDQDEEYWQDEAD***KR***GEHRFAFGL**G*KR***EVAPQDIAALKEELDKDKSKNNDEHEHGSANKSSQTVSENSTGADKGSQNINRVDMQDKDGSAANSYHLV***KR***EEEASNYWVDDAEDIE***KR***NPRYKFIGL**G*KK***TLGYEI***RR****P***G*RR***MYNFGL**G*KR***IPMYDFGV**G*KR***ADISADH*

>*Atelura formicaria* (Zygentoma) AST A [GenBank:GAYJ01034351.1]

***M***LTSRCRDLLLFAPLWVVLLTLNVMADDIAAELSDLTDEDGLQLTDADESAADIY***KR***LYDFGL**G*KR***AYSYVSEY***KR***LPVYNFGL**G*KR***SPHMYSFGL**G*KR***LPSGSSGHLYSFGL**G*KR***TRPNEMYAFGL**G*KR***PAWGPDYDYSEEDVEEELEPELEED***KR***NKLYSFGL**G*KR***ARSYSFGL**G*KR***QKQYSFGL**G*KR***PMYDFGL**G*KR***SGKQYSFGL**G*KR***PQPSRDGSRFNFGL**G*KR***DPGFSYYDEDESSPRSEEMFHDEED***KR***GEHRFSFGL**G*KR***EVPSQEVEALKQEMSHNHQGEVKSDISED***KR***ARTYSFGL**G*KK***SDNNGTAEVAAAADNHQDDDEHLAGHI***KR***SPHYNFGL**G*KR***GDGEWSDLPLMEAED***KR***NPRYKFIGL**G*KK***TALSGSGGPGS***RR***MYSFGL**G*KR***RPMYDFGL**G*KR***SE*

>*Nicoletia phytophila* (Zygentoma) AST A

***M***FEPRLHHLLFYAPVFILLLTFEVHGDDLSTELNEAASAEDGQLLSDSDEDAVELY***KR***LYDFGL**G*KR***STSFASQF***KR***LPMYNFGL**G*KR***SPSMYSFGL**G*KR***TPGMYSFGL**G*KR***SPGMYSFGL**G*KR***TPGMYSFGL**G*KR***TPGMYSFGL**G*KR***TPSKSQGLYSFGL**G*KR***PAPGQMYSFGL**G*KR***SPFSAEYDYEEEESEEDADEPEVTE**G*KR***DAKMYSFGL**G*KR***SRMYDFGLGK**G*KR***RSRMYDFGL**G*KR***KADRLYSFGL**G*KR***LPMYDFGI**G*KR***GMQPYAFGL**G*KR***ASPSRQSGLFNFGL**G*KR***DHDFYDDDEENEELNEDMLSEE**G*KR***KDHLYAFGL**G*KR***EVPSQEIDDFKQEMDEKHLEDLKHEGHSE**G*KR***NKVYSFGL**G*KK***SQVNGSGEVAESLDNQQGNDDHLAGRI***KR***SPHYYNFGL**G*KR***SDDLEEYDSEEWNEVPVMDETE**G*KR***SPRYKFIGL**G*KK****TSGNGLSGP****GRR***LYSFGL**G*KR***LPLYDFGI**G*KR***SDH*

..............................................................................................

>*Xibalbanus tulumensis* (Remipedia) AST A

…ESSPD***KR***LATYGFGL**G*KK***AYDFGL**G*KR***AYDFGL**G*KR***AYDFGL**G*KK***ALDYGTGLE***KR***LSSLYTLEM**G*KR***LSELYKSGL**G*KR***PGEMYSFGL**G*KR***SDSSVDDLEDDDGLDEE***KR***GSAAYSFGL**G*KR***DPAQYAFGL**G*KK***DRGPYEFGL**G*KR***EQHSYAFGL**G*KR***DPQAYAFGL**G*KR***DPYGFGL**G*KR***DPYGFGL**G*KR***DPYGFGL**G*KR***DPYGFGL**G*KR***EPYAFGL**G*KK***DPYAFGL**G*KR***DPYGFGL**G*KR***FPYEFGL**G*KK***APMYEFGL**G*KR***EPMYEFGL**G*KR***DKGMYHFGL**G*KR***HEFKEDY***RR***FDFGL**G*KR***NDELDETGEDE***KR***DGNKFSFGL**G*KR***EVDPTELDDMLMRSDDEEEK***KR***SMSYGFGL**G*KK***SASDSDTEKDNFSENNEHLIPKE***KR***SPAYGFGL**G*KR***QKH***KK***AYDVSVGETIKPYNSKIEKSERKTGSVGQ*

>*Anaspides tasmaniae* (Malacostraca) AST A

…DDSLPA***KR***AYTYVSEY***KR***LPVYNFGI**G*KR***WIDNED***KR***ERYSFGL**G*KR***SRQYSFGL**G*KR***NENSDYPDRLGFDYVPASGTPDRSPG…

…GL**G*KK***AGTYSFGL**G*KR***SDPIAYRVGRSSYDFGLGNRNNQNTNEVYNYTVGEEAVNL*

>*Lithobius forficatus* (Chilopoda) AST A1

…DEEAELSAEVEE***KR***NKMYGFGL**G*KR***EDEDMESA***KR***NKGMYSFGL**G*KR***EEE***KR***NKMYGFGL**G*KR***NKGMYSFGL**G*KR***EAVDDEDVE***KR***NKMYGFGL**G*KR***EEEE***KR***NNKMYGFGL**G*KR***EEDA***KR***NSKMYGFGL**G*KR***DEDNMEE***KR***SKSYAFGL**G*KR***DSDSELEED***KR***SKSYSFGL**G*KR***DSDSELEE***KR***SKSYAFGL**G*KR***DSDSELEED***KR***SKSYSFGL**G*KR***DSDS…

>*Lithobius forficatus* (Chilopoda) AST A2

…GL**G*KR***DSDSELEED***KR***SKSYSFGL**G*KR***DQGDDELEE***KR***SKSYAFGL**G*KR***ETDSNNENNEE***KR***AKSYSFGL**G*KR***DDEDEGPEV***KR***AKSYSFGL**G*KR***EEDDEAME***KR***MKSYGFGI**G*KR***GGDAENVD***KR***NKNYSFGL**G*KR***GAEEVVNESED***KR***AKPNQYAFGL**G*KR***TASAEEHKAAAAVSTANDSATPHH*

>*Hanseniella* sp. (Symphyla) AST A

…***KR***GPREKLYSFGL**G*KR***ATIATQVEPSSRENSRTEFDL**G*KK***NDSDSSSVDA***KK***VSTARPAASS*

>*Eudigraphis takakuwai nigricans* (Diplopoda) AST A

…LV***KR***ARSFGFGLGN***R***SRSFQFGL**G*KR***SSEEGEDEVVDEDEILPDED***KR***TKSMAFGL**G*KR***SQKQFGFGIGI***R***PNQQFSFGL**G*KR***AQAYTFGL**G*KR***PDRQFSFGL**G*KR***PSSQQFAFGL**G*KR***AKNFEFGL**G*KR***AKPYTFGL**G*KR***SVDESNQLMVASNETARSIEDTRQSNGGQSKSQPDVQAVHKEKDRSGQR*

..............................................................................................

>*Drosophila melanogaster* AST A ([FBpp0084119](http://flybase.org/reports/FBpp0084119.html)]

***M***NSLHAHLLLLAVCCVGYIASSPVIGQDQRSGDSDADVLLAADEMADNGGDNID***KR***VERYAFGL*G****RR***AYMYTNGGPGM***KR***LPVYNFGL*G****KR***SRPYSFGL*G****KR***SDYDYDQDNEIDYRVPPANYLAAERAVRPGRQN***KR***TTRPQPFNFGL*G****RR****

>*Daphnia pulex* AST A [[Dappu1_312277](http://genome.jgi.doe.gov/cgi-bin/dispGeneModel?db=Dappu1&tid=312277), GenBank:[EFX87432.1](http://www.ncbi.nlm.nih.gov/protein/321476471?report=genbank&log$=prottop&blast_rank=1&RID=XHDUXZFW015)]

***M***LFLSTALFLLVALLQSSQCTDVNNDSSVIEAVGAGGGDSQAKAESLASAEDKLSALTADGMATRA***R***YFKSFGGNPTGDPNLNIYSFGL*G****KR***TSRSYSINPYSFGL*G****KR***GGNAKSYPQQIPYSFGL*G****KR***NPTKYNFGL*G****KR***PDRFGFGL*G****KR***NLKDDDLDQWLNDEEYSQFDDTEEEIEGREDDSQEIMDVN***KR***SQMAAGQQQQQQQQAFFPSHLQSAFYGGPFMSNLARANTHALGKSRTFNQQAATHDLPNL*G****KR***LPVYNFGL*G****K****

Allatostatin C + CC (AST C + AST CC)

>*Nipponentomon nippon* (Protura) AST C

***M***NTDSIRLVLLLSTMAITISLGMGNPQQQSPHRRLQSVNDLDLIEDDGELETALLNYLFAKQIA***RR***LHSQLDIQDLQR***KR***SYWKQCAFNAVSCF**G*K****

>*Nipponentomon nippon* (Protura) AST CC

***M***MFELHLWTMSSSLLFAVLAFLLRPEFSLCLQDGHPAYYLTPLQVKV***KR***GEVNLERVDTLYLPEKQPNLDSNQLVI***KR***SSFLMDKLRLALENALEEDERILELKQKQQPIQTKADDATRYEEEHQQRAAKLKATAAYQLTPAAVAQLEMQ***RR***GQQDGRVYWRCYFNAVSCF*

>*Acerentomon* sp. (Protura) AST C [GenBank:GAXE01005791.1]

***M***KSESVSLFILLALAVVIGLVQSHVTKGSSNHHQFQSTQDLDLMEDDGELETALLNYLFAKQIA***RR***LHSNLDIQDLQR***KR***SYWKQCAFNAVSCF**G*K****

>*Acerentomon* sp. (Protura) AST CC [GenBank:GAXE01125568.1]

***M***MNRMDQMNIMCCFIWVLLAFLIISVVSANPVYILTPFVSKV***KR***GEVNLEKPETLFLPEKQMESSLLDHPLMGEQS***KK***STFLMNKLRYALENAINKEERLIE…

…KNYLLTPSAVAQLEMQ***RR***GQQQDGRVYWRCYFNAVSCF* [GenBank:GAXE01135524.1]

>*Filientomon takanawanum* (Protura) AST C

***M***NIGAMCLLALSALVGLVHCHSAKASPSRHQFQSAQDLDLVDDDGELESALLNYLFAKQIA***RR***LNSNLDIQDLQR***KR***SYWKQCAFNAVSCF**G*K****

>*Filientomon takanawanum* (Protura) AST CC

***M***LAILVVVVLAAIPLHSVGGSPVYILTPIMSKV***KR***GEVNMERADPLFLPDKQTDTNSMELAIPFIGDQS***KK***STFLMNKLRHALEN…

…KLKATAAYQLTPAAVAQLEMQ***RR***GQQDGRVYWRCYFNAVSCF*

>*Anurida maritima* (Collembola) AST C [GenBank:GAUE01052945.1]

***M***PRWMIEVGLLLVFSITLATSSG***KR***LSHEYAFGGLNNQGAAGDLGQSQQPSMNGAIFPPEIESGNKGTGILNLDSPEGGWEDSAGSMENALMNYLYRKHMIQKMKQQIATDNLRQQQR***KR***SQGQYWKMCAFNAVSCF**G***

>*Anurida maritima* (Collembola) AST CC [GenBank:GAUE01008269.1, GAUE01008268.1]

***M***TLILCVFVSFGWSDPVPSPEPKKAHEVSNGSLQDRKVEKEKALEVVKRSAEESDEEETPTVYYPMIIS***KK***SATVLNKLVTALRNSIDRNPMRNANFLREKELVSLRNSANYDMFNLQ***RR***NQKERKNYLRCYFNAVSCF*

>*Anurida maritima* (Collembola) AST C-like[GenBank:GAUE01009178.1, GAUE01009177.1]

***M***AMSATLHNFLMLATFVILFATKAMGAIDPLENGVFGPIETINDEESGNRNRANGFERQEYQNVPMIGTRWPTDNNQDWLHLSQMMKLLLGGIQNRKANEQQSLDVPRTPQRQSYG***KR***QIIYRQCFFNPISCF*

>*Tetrodontophora bielanensis* (Collembola) AST C [GenBank:GAXI01143148.1]

***M***GKWIGTGILIIILVSLQLLDSTDGKTILPQQQQQQQLQMQQQQGSQDDKSGVLIPDMDVNDDEGGGNVEASLLNYLYTKQMLQRMKQQLELSDPMR***KR***SYWKQCAFNAVSCF**G***

>*Tetrodontophora bielanensis* (Collembola) AST CC [GenBank:GAXI01133916.1]

***M***MMIIKFHGIPLLLIFSFFLTICICAHIPNNPKQSSSSSTTLDVANANKIMNKVSKYT***KR***DLVEQDEDDNVIFPMVIS***KR***SATALGKLMHLLRTSMDHSGGKIISSSREKESSPMHSANYEMFNLQ***RR***NQKSNYLRCYFNAVSCF*

>*Podura aquatica* (Collembola) AST C

***M***SY***M***RLVSN***M***RNVGVKAFTSLIFLLLISCADSKNIGHSGGDVISSEISAPNLQQQFLPEEKQQNPLYNFETSDVVYDDGPLFPSPMENALFNYLYT***RR***MMKKVKEQSPIDFNAANNILK***KR***NSNGNYWKQCAFNAISCF**G*KKK****

>*Podura aquatica* (Collembola) AST CC

***M***VKIGRKSFISSTFIILIFLIIIISSAASKKHH***KR***ARVLNDTLVIDRDGEI***KR***NSTNYQNLTQVESKKAEKLPNVKKSEIEVSDSQNLDRNGGGDEQSLKNLKIG***KR***SIIEELYDVDEGQVEEEPLPPMLISKKAAKSLNKIANVIRGSYDYKPRNSYRQREMMTRSGATNEKMYNIIQ***RR***NPKERKNYLRCYFNAVSCF*

>*Folsomia candida* (Collembola) AST C [GenBank:GASX01079362.1]

***M***VTAAYKTSWTVLILPFIVVVMATLISASPSGSPSSEDLLGSRVYYRNRETLSNPEDNVVPITLDAGQQSSPMLYDPHVLSAQAPSDNALITYLLT***RR***FALANNNRLRSYVDSSSNEISR***KR***SYSKYCAFNAVSCF***GKK****

>*Folsomia candida* (Collembola) AST CC [GenBank:GASX01011215.1]

***M***MNLILILAFSAVLPSSIVCGTIARTSSDMKARPPILNQSLPPSPLLASHSKNYNSSHPASRNLDARE***KR***EIPAEANDVLFPDYRISEMMP***KR***AAAGLNKIIYVLRNSFDKAAPSSPENDVGSRENDNFSGDINRALVRGSSVDRFPAALVE***RR***GQAAQKDRKIYLRCYFNAVSCFR***KKK****

>*Folsomia candida* (Collembola) AST C-like[GenBank:GASX01004300.1]

***M***KTLSFLIVTLFVLSSDFNSCCEAQKVYKLDPETMSDDDYSPEMDLVGPSSPPQSSEEMNIKPNFPSRFTSLASSPQSYNYFTPGG***KR***YLLSNTQLSTLNGHSPKYILQPSTRQSGFIPQQKSYYGRMSGFGMGGFGGVVGGGGRHSSAFMPPVSPR***KR***QILFRQCFFNPISCF*

>*Bourletiella hortensis* (Collembola) AST C1

***M***QLAVGILVIIMIIGSSLSSPISQHVLDDPEAMAGLQLEMSPGEADMMASNNGGVEGRNERDLYVTPLLLWKLLQLSRAHQEPASPP***KR***NHYKQCAFNAVSCF**G***

>*Bourletiella hortensis* (Collembola) AST C2

***M***GPSGILKIFAILWVIAVATAMNLPVRPSEEIVDSPQDLELVQELQENSKLNPITFTPLERLVMRLRGS***KR***SWRPCTFNAVSCF**G***

>*Bourletiella hortensis* (Collembola) AST CC1

***M***DIKSFVILLYFASFQAVSSMPYEDVPRTDLS***KR***SAAALSKFLLQSLSNQMEAEGKTAAGLDPVYLAHAQRRLLDQQLASPYRSDTVVNLE***RR***EPKQNEGARKYMRCYFNAVSCF*

>*Bourletiella hortensis* (Collembola) AST CC2

***M***MMMLMGIINPFSASSASNNNGMVMARTIPASALDQLDYYYLPDRNLVMASATALNKLMLALRSYEKASPEKSNVGKAVGGDLGKAGSGSNLDTFNMQ***RR***NQKERKVYLRCYFNAVS…

>*Bourletiella hortensis* (Collembola) AST C-like

***M***KLCQLFEILLIAICFCGCCLCKPFYVDYEDVEELESPGAGGDGSAKNVPEVPMSPSMVAAATATGMATPL***KR***SSTDTESENTWNGGGSNNGMIRHTLKVPTNDRPRFVTQPENNGWYLNSPSATVARHIPVRLFPTYNFQPQSSSSGM***RR***TSPFGLR***KR***QSILYRQCFFNPISCF***KK****

>*Orchesella cincta* (Collembola) AST C

***M***AKVIVLAVPLILLAVNNSFAAPATSNEDQIGFAPARQVFRSRENPXLAEYNIVPLIPDPGIPAENTLDNQQNVEALLKYLLARNSYFTNRLRPFSSLDSAGNELAR***KR***SSYSRSRYCAFNAVSCF**G*RKK****

>*Orchesella cincta* (Collembola) AST CC

***M***VCITHFVVSLFILVLPFEISGFTI***KR***DSKAKSPPIQLNGMESPSTIAPTHHVPLNSVPSRSLSVV***KR***EVMTDD**G*R***VSNLYDPGLMIMS***KR***SAAALNKLMSALRSSFDEPASNSFENASETATQDN**G*R***FPTVGFRTSGSGFNSGTYTSNAVQ***RR***GSKDRKVFLRCYFNAVSCF*

>*Orchesella cincta* (Collembola) AST C-like

…IPGVSYPSSFEDGP***KR***YPAQPQISSESSPDINSAENPGFRIRQKILNLRNQYGDKWLSLVGPYAYR**KR**QILYRQCFFNPISCF*

>*Pogonognathellus sp.* (Collembola) AST C [GenBank:GATD01094276.1]

***M***VSKTSKSTLWTLSILLTTANLIFAAPSINNEESSGSRAFIHSAPGGLPPSEFDLMPMVEQDNAVQASLPYQYVPIPATPVRQPSFENAILNYLSAKHLMMSLLNSKA***RR***FNDWDSNEVNR***KR***SYKLCAFNAVSCF**G***

>*Pogonognathellus sp.* (Collembola) AST CC [GenBank:GATD01099903.1]

***M***NSICIVFCVFLLPAFALCKPAVTVIKSSTVSEDNYSPNEANLT***KR***TDPANLADFQPLSIV***KR***DVGNMENELYSQHPYPMIML***KK***SATALNRLMYRLKNSFDKSGPSKEIGPRTSYYKQDSDKSLGLSPGSAMQNDESYPLLQ***RR***DQKERKYLRCYFNAVSCF***KK****

>*Pogonognathellus sp.* (Collembola) AST C-like[GenBank:GATD01087920.1]

***M***AKVFHCSTIFLLSSIFFACFNISEAATFPGAFMNGIDRTMLDEYRERSRLANSEFGDLQPEENAWSPAKGLSRIPETLTWEDTDASNTVGENDMESRADEEKSGFIKVNVRAFNPPSRLRQIENVNPRLAFELLR***KK***SMKAFVPYPQK***KR***QIHYKQCFFNPISCF*

>*Sminthurus viridis* (Collembola) AST C1 [GenBank:GATZ01099087.1]

***M***YKLTIVLLGLTLISKSLCKSIYEDIEADNANVPQQQVSFQEEAPSPAIVLA***RR***LAALSRLQDQTDAEM***KR***SRWKQCAFNAVSCF**G***

>*Sminthurus viridis* (Collembola) AST C2 [GenBank:GATZ01099226.1]

***M***QFLALALVLTTIMISDSLSSPVSLSREDQEAVGFPLTLDAPSLSSDPELLQESDNRNDKEVLVSPILLWKLLQLSRLHQEAGS***KR***SHYKQCAFNAVSCF**G***

>*Sminthurus viridis* (Collembola) AST C3 [GenBank:GATZ01013412.1]

***M***TVKISLKILAVLVALEVVLAVNTLPVRPEDDTELGGDIDTMVQEIQENAKSNPITFTPLARKLLMMRLQQEV***KR***SWRQCTFNAVSCF**G***

>*Sminthurus viridis* (Collembola) AST CC1 [GenBank:GATZ01099567.1]

***M***GMNPKSPLFILFSLSLAMTILSSYTMARTLPSSSLDQLDFYRPDKTLMISKKSATALNKLMIALRSIDKSSAEKGNGLDSPDTVKYNPAFEPFNMQ***RR***NQKERKIFLRCYFNAVSCF*

>*Sminthurus viridis* (Collembola) AST CC2 [GenBank:GATZ01101435.1]

…SL***KR***SANALNRLILQS***RR***HKEEPGPTEDHGRKMHTALGIVAHEALLSEINELIEDQKDYNTNHME***RR***GPKPDQRTFSRCYFNVVSCF***K****

>*Jordanathrix leptothrix* (Collembola) AST C1

***M***HKITIFLLGVTVLHVVLSKPVYDDPDSEIGVSSVIENGQQEDLIPAVNLARKLLILSKLQEQNDREV***KR***SRWKQCAFNAVSCF**G***

>*Jordanathrix leptothrix* (Collembola) AST C2

…***KR***SHYKQCAFNAVSCF**G***

>*Jordanathrix leptothrix* (Collembola) AST CC

IPSSASSSSASFFVLLFMMVMSSTNLMVIGPSAVSAAMTFPSLSSSSTRQLDFYRPDKMFMME***KK***SATALNKLMIALRSYEKTSAEKGNNNGDTTGNGNGKYGTGVDTFNLQ***RR***NKQNGKIYLRCYFNAVSCF*

>*Campodea augens* (Diplura) AST C [GenBank:GAYN01141404.1]

***M***SPVALISPRAFLLITLLTWAAVATVNSMPSPDKERVYQTDLDLVGDDGSFDTALINYLFARQMINRLKNQVDVTDLQR***KR***SYWRQCAFNAVSCF**G*K****

>*Campodea augens* (Diplura) AST CC [GenBank:GAYN01089093.1 + GAYN01130244.1]

***M***EKFSSCILDMIIVLFAFSFFLSLCLAGPLDITAKEQIQNHHIL***KR***DAEEPDYYPTDLENVQLP…

…DKLASTSQKIIDEPVGGGSRYAEGPIAAPLTDVRQVLKKVGLQ***RR***GQHNDRVYWHCAFNAVTCF***KKK****

>*Lepidocampa weberi* (Diplura) AST C

***M***SPLFLVSPRSILILLVLTWIAVTATKALPTPEKERTPYQTDLDLIEDDGTLENALINYLFARQMVNRLKSQVDVTSLQR***KR***NYWRQCAFNAVSCF**G*K****

>*Lepidocampa weberi* (Diplura) AST CC

***M***EKYSSCIQDMIVLLFTFSLLISLCLSRPMEANNSKTLGSHVQ***KR***DIEEADYYPTEDENPQVLQNQ***KR***ISSWAFGKLADTVQKIIDEPVGGGTRYAEGPIAAPLTDVRQVGLQ***RR***GQHNDRVYWHCAFNAVTCF***RR***K*

>*Lepidocampa weberi* (Diplura) AST C-like

***M***MMMHCPLQWRWNACFWGVAIGILSWGLLAQATPLMTNYPGNILEDTDNSPSKSLGDWSLGLPKSAGSSEDRGALLHPAQFPVSWNYLTAPKD***RR***SFFSDQKQPAE***KR***QIRFHQCYFNPISCF***RRR****

>*Occasjapyx japonicus* (Diplura) AST C [GenBank:GAXJ01012337.1]

***M***HPDSACRFLLIFLVAMAILNVANSKNLPGESERDRLAAAGLTEEDNNAILDDDVMGYLLTKSMV***KR***LRNQVEASDMQR***KR***SYWKQCAFNAVSCF**G*K****

>*Occasjapyx japonicus* (Diplura) AST CC [GenBank:GAXJ01011505.1]

***M***EGRGCWRRRASTAALVLLQLFFCVGLAQQPQQQTQTQPREVSGAGNNHTAEGLPQQQVGDSNSGSREEGQGQQQMMVR***KR***EAEMAGPQLDLLPMPHND***KR***AALILDKLMFALQKALDEPEETFGAPAAPVPEALRAPQVDLQ***RR***GQIKGHYYVPCFFNAITCYR***KK****

>*Catajapyx aquilonaris* (Diplura) AST C

***M***HLSSTCRTLLLLLVAAAVFVNVVNSLTIPNSEEER***RR***YPRVLADDEDAVDNYQMNYPLLKAFL***KR***LRDQDVVTDMER***KR***SYWKQCAFNAVSCF**G*K****

>*Catajapyx aquilonaris* (Diplura) AST CC

***M***EGRTCCRQLGTAALVLLQLCFVGMAHPQQQQQAQVIQQEQQVNRSGMHLKN***KR***EAEMPVPNQQDVQMPHND***KR***AALILDKLMFALQKALDEPEEGYGPAAPVPEALRAPQVDLQ***RR***GQTKGRYYVPCFFNAITCYR***KK****

>*Pedetontus okajimae* (Archaeognatha) AST C

…EAKSIGQGEKEQFVSDLDLVEDDGTVDTALINYLFAKQIINRLRNQLDVTDLQR***KR***SYWKQCAFNAVSCF**G*K****

>*Machilis hrabei* (Archaeognatha) AST C [GenBank:GAUM01172669.1]

***M***ASCKWIVLLSLVGCVALGWSEAKSLGQGEKEQFVNDLDLVEDDGTVDTALINYLFAKQIINRLRSQLDVTDLQR***KR***SYWKQCAFNAVSCF**G*K****

>*Meinertellus cundinamarcensis* (Archaeognatha) AST C [GenBank:GAUG01190451.1]

***M***ASCKWFVMLSLVSCVALAWSDAKSLGQEEKQPYLNLDLVEDDGSVDTALMNYLYAKQVINRLRSQLDVTDLQR***KR***SYWKQCAFNA…

>*Meinertellus cundinamarcensis* (Archaeognatha) AST CC [GenBank:GAUG01241714.1]

***M***ALQRDSSIFVILCALFLVLIFNVPQGSTSKATLYYPQLSDRIAAESTTVVS***KR***AALLLDKLMYALEQVLDEDKTGKQVLAAAPAAPQEPTYPVEQMELQ***RR***GQQKGRVYWRCYFNAVSCF***KR***RK*

>*Machilontus* sp. (Archaeognatha) AST C

***M***AAYKFFVLLSLFSCVVLAWTEAKALAQGEKDDLSMVDDDGSVEAALMNYLYAKMVV***RR***LQNQLDISDLQR***KR***SYWKQCAFNAVSCF**G*K****

>*Machilontus* sp. (Archaeognatha) AST CC

***M***ALQRSLIVLTSTLLVVVFIILPQGHCAKATLYYPQLSDRFGTDSTAVVS***KR***AAILLDKLMFTLQQALDGEMGKQTDSQPPVPQAQTFPIEQMDLQ***RR***GQQKGRVYWRCYFNAVSCF***KK***RK*

>*Tricholepidion gertschi* (Zygentoma) AST C [GenBank:GASO01249200.1]

***M***VSTKLMLVMMIAMVTLSWAFGKTLGQPEKERFLNELDLVDDDGSVETALMNYLFAKQIVNRLRSQLDVTDLQR***KR***SYWKQCAFNAVSCF**G*K****

>*Tricholepidion gertschi* (Zygentoma) AST CC [GenBank:GASO01248131.1]

…TRETLVVP***KR***AALLLDKFMVALEKALDDGASSDSAKSTPVVAPEQMGLE***RR***GQQKGRIYWRCYFNAVTCF*

>*Thermobia domestica* (Zygentoma) AST C [GenBank:GASN01005788.1]

***M***MTTKCMLMLAVAMLTLSWAFGKTLGQPEKERFINDLDLVDDDGSVETALINYLFAKQIVNRLRSQLDVSDLQR***KR***SYWKQCAFNAVSCF**G*K****

>*Thermobia domestica* (Zygentoma) AST CC [GenBank:GASN01397206.1]

***M***GHFSSLGLVFGACFLTLIWLCLPSPATCRTTHLFDDSSAALETQDYTTGVRYDEYPVVVP***KR***AALLFDKIMAALQSSLDEGGGTRTEYVVPEQMDLQ***RR***GQQKGRVYWRCYFNAVTCF***RK****

>*Atelura formicaria* (Zygentoma) AST C [GenBank:GAYJ01274481.1]

***M***ARRILLVLLVAMVTLSLAFGRTLGEPEKERLLNELDLVEDDGSIENALMNYLFAKQVVNRL***RR***QLDVTDLQK***KR***NYWKQCAF…

>*Atelura formicaria* (Zygentoma) AST CC a [GenBank:GAYJ01022806.1]

***M***GRSWAAVVLVTWLCLTVESKPLTRDMAEQDTLLDTSMDYRLGVRYDDYPVVVP***KR***AALLLDKIMGALEKAWDEQPQPAATNRVMDSQIQ***RR***GQQKGRYVRCYFNVVTCF*

>*Atelura formicaria* (Zygentoma) AST CC b [GenBank:GAYJ01022807.1]

…DSQVD***RR***FGDVPSPKENGTGIRSTKEKALTDLARGKYSQLSQTDSRNRSIQ***RR***GQQKGRYVRCYFNVVTCF*

>*Nicoletia phytophila* (Zygentoma) AST C

***M***ARKLMFVLLIAMVTLSWAVGRTLGEPEKERFVNELDLVDDDGSIENALINYLFAKQIVGRL***RR***QLDVTDLQR***KR***NYWKQCAFNAVSCF**G*K****

>*Nicoletia phytophila* (Zygentoma) AST CC

***M***GQSLLLVITCLCLNAASQPISEDEDERDVLNPPEYRVGVRYDDYPVIVP***KR***AALLLDKIMGALEKAWDEEPRVAAIHDSRNSQVQ***RR***GQQKGRNSLRCYFNVVTCF***K****

..............................................................................................

>*Xibalbanus tulumensis* (Remipedia) AST C

***M***STFYRIFVYLLVIFVAVSAVIGKSLSQVEKERFGNELDLVDDDGSMENALLNYLFAKQMVSRLRSNMDVSDLQR***KR***SYWKQCAFNAVSCF**G*K****

>*Xibalbanus tulumensis* (Remipedia) AST C-like

…VNLQDRDKAMNNFWKLLGFNGFDIRDSSKSAPHQSAFVDSSAASSAGGYRNLAEDVDLPYGAKMLGS***KR***QIRYHQCYFN…

>*Anaspides tasmaniae* (Malacostraca) AST CC1

…PRSRPLHTNPMELQ**RR**GNTDGRLYWRCYFNAVSCF*

>*Anaspides tasmaniae* (Malacostraca) AST C-like

…RDFDESIGKGEARVQ***KR***QVEQNEASEERELAALRSLILQRLISEMQSGEWKDLPIFRANGLDDSVEEGVEKQ***KR***MFDALPVARGQFPAT***KR***QIRYHQCYFNPISCF***RR***R*

>*Lithobius forficatus* (Chilopoda) AST C

***M***ESSKIFALFFLLFLAVSVVVSKSIGEHENPNYGPDLSLVNDDGSLDTALINYLFARQMI***KR***LQSNMDVTDLQR***KR***SYWKQCAFNAVSCF**G*KK****

>*Lithobius forficatus* (Chilopoda) AST CC

…QPQ***KR***STLLLDRLVYALQKAIKQDAVQQQDMELQ***RR***GPD***RR***VYWRCYFNAVSCF***RR***K*

>*Lithobius forficatus* (Chilopoda) AST C-like

***M***VSISSSSLPQAFTLLVLLVIAVTCTVQALDARTNDDESGPDVSRLQLSSDDVDPLRSYMLSRIVDDLQMVSSPQYQQRQE***RR***AQEMT***KR***QIRFHQCYFNPVSCF***RR***RK*

>*Hanseniella* sp. (Symphyla) AST C1

***M***ALLLRSILALLAVILALNQVSPVSTAAENDLHSYDHSNPTYHMIDDDGSLDTALINYLFAKQMI***RR***LQASADITDLQR***KR***TYWRQCAFNAVSCF**G*KK****

>*Hanseniella* sp. (Symphyla) AST C2

***M***ALIVRSSLVVLAVIVAIHQVVGKSTSESESDVHGFGQEPGDRTTHLIDDDGSLDTALINYLFARQMI***KR***LQASADITDLQR***KR***TYWRQCAFNAVSCF**G*KK****

>*Hanseniella* sp. (Symphyla) AST CC

…KDLKDLKT***KK***KYWRQCAFNPVACFGNKSAGQGLNS*

>*Hanseniella* sp. (Symphyla) AST C-like1

***M***KSTVVQTVSPAVVCMTFAAVIAITLAGQTEAGSSPSASGAVDEILTEYPRPDAQEAEQMRTLLLHKLVNDLRTLSDQRGVGSARMSNTDHIENV***KR***QIRYHQCYFNPISCF***RR***RK*

>*Hanseniella* sp. (Symphyla) AST C-like2

***M***KTTVSNVSSLMLCVTLAITISLVQAGPSVGPAAPVSMEEILTDYPKDAQEAEQMRAMILQRLMDDL***RR***LSEQKNSASTRIGGSSDHIETV***KR***QIRYHQCYFNPISCF***RR***RK*

>*Eudigraphis takakuwai nigricans* (Diplopoda) AST C

***M***AFSNAFTLTCALSLLSAWTIASFFVSATSAKIAPRISAYPDEASVNEAxSFRSEPSGYTGDASGEQNSEEYRKLLFRKMVSMIHSLPKESAVIENGFALPRISPEYDYVANMGLA***KR***QTTYRQCYFNPVSCF**G*RR***RK*

..............................................................................................

**>***Drosophila melanogaster* AST C-PA [[FBpp0079821](http://flybase.org/reports/FBpp0079821.html)]

***M***MKFVQILLCYGLLLTLFFALSEARPSGAETGPDSDGLDGQDAEDVRGAYGGGYDMPAQAIYPNIPMDRLQMLFAQYRPTSYSAYLRSPTYGNVNELYRLPES***KR***QVRYRQCYFNPISCF***RK****

>*Drosophila melanogaster* AST C-PB [[FBpp0290975](http://flybase.org/reports/FBpp0290975.html)]

***M***MKFVQILLCYGLLLTLFFALSEARPSGAETGPDSDGLDGQDAEDVRGAYGGGYDMPAQAIYPNIPMDRLQMLFAQYRPTYSAYLRSPTYGNVNELYRLPES***KR***QVRYRQCYFNPISCF***RK****

>*Drosophila melanogaster* AST CC-PB [[FBpp0112947](http://flybase.org/reports/FBpp0112947.html)]

***M***LIILLVLIQNFELHMCRQLMVYPGADKRSPDKLLTIGGSAAGEVTLPEANTPADD***KR***AGGSRSAPSQPEEIFSAPADEGYDEYPMVVP***KR***AALLLDRLMVALHHALEQERSEQRIGEFFGDRNILSGKFGDSHNGMEHHQAREDGMYSDDDAGTLLDYDFKDLNQINRATGET***RR***AGADRSGTSTHSGSPAGS***RR***IQPSGSGGGRAYWRCYFNAVSCF*

>*Drosophila melanogaster* AST CC-PC [[FBpp0310218](http://flybase.org/reports/FBpp0310218.html)]

***M***LIILLVLIQNFELHMCRQLMVYPGADKRSPDKLLTIGGSAAGEVTLPEANTPADD***KR***AGGSRSAPSQPEEIFSAPADEGYDEYPMVVP***KR***AALLLDRLMVALHHALEQERSEQRIGEFFGDRNILSGKFGDSHNGMEHHQAREDGMYSDDDAGTLLDYDFKDLNQINRATGETLMPKSFQ***RR***AGADRSGTSTHSGSPAGS***RR***IQPSGSGGGRAYWRCYFNAVSCF*

>*Daphnia pulex* AST C [[Dappu1_290676](http://genome.jgi.doe.gov/cgi-bin/dispGeneModel?db=Dappu1&tid=290676), GenBank:[EFX85706.1](http://www.ncbi.nlm.nih.gov/protein/321474742?report=genbank&log$=prottop&blast_rank=1&RID=XHHW218R015)]

***M***MAKISAVVPVAILLYLAASGAAKSTDREETESTDFGQDIEVLGAVPDDGSVETALLNYLFAKQIVARLRTNANPQDLMR***KR***SYWKQCAFNAVSCF**G*K****

>*Daphnia pulex* AST CC [[Dappu1_98246](http://genome.jgi-psf.org/cgi-bin/dispGeneModel?db=Dappu1&tid=98246)]

***M***LISHPEFKTSACCILMLTIMVANSASGAALEGQVDDRSTEEEHQDWRQPLPTV***KR***AVALLNKLVMVANRANNNKKQRYQQANPLIKHHSQSPHLNDMQHREGEPLPNNSAENIDDHHHIHQKAIALPRMGFE***RR***GQSSQRVFWRCYFNAVSCF***R****

>*Daphnia pulex* AST C-like [[Dappu1_442998](http://genome.jgi-psf.org/cgi-bin/dispGeneModel?db=Dappu1&fTable=JAM_UserModels&fId=2260http://genome.jgi-psf.org/cgi-bin/dispGeneModel?db=Dappu1&fTable=JAM_UserModels&fId=2260)]

***M***LGKLYLVVMAIFSLAHSGPVIGDGDALVHIFEQDYDNSNVITSPEDLPSTVSSLTADVND***KR***LQYYLWM***KR***AADGARSGGMGRSKQLRYHHCYFNPISCF***KK***RAYESSKISDSYIGRW*

Allatotropin (AT)

>*Nipponentomon nippon* (Protura) AT

***M***QSLSATTIMKAVALLCLFCCFMVLMTSAQQEQRPRKI***R***GFKNAALSTARGF**G*KR***EFRFQQPAQEISYETPYGPSITKFDSIPIDLFVEHLLANPKLIRVLTQQCLDRDDDGLLNTDEVMSRFAPSSGSTNQN*

>*Acerentomon* sp. (Protura) AT [GenBank:GAXE01016877.1]

***M***SSVSLKSIVVFCLFCCFIVLIVGAQQETRPRKI***R***GFKNAALSTARGF**G*KR***DFRYPAHINQDPASFEPYLAAPYAAKLDSIPIDLFVEHLMSNPRLTRILTQQCLDKDEDGVLSSEEIVSRFGGNNN*

>*Filientomon takanawanum* (Protura) AT

***M***QSITSSATLKTIAMFCLFCCFIVLIVGAQQETRPRKI***R***GFKNAALSTARGF**G*KR***GDFIYPSQISSEAAYEPSLLATYAKFESIPVDMFVDHLLANPKLARVLTQQCLDKDDDGLLSSDEIISRFGTSNN*

>*Anurida maritima* (Collembola) AT [GenBank:GAUE01054459.1]

***M***SSYFLFVFVTLSVMSIALTFSQSETVRE***RR***GIPRSS***R***GFQIQNGKLSTARGF**G*KR***GSFGRENAFFRLTAAEGSERNIGSFPLIEPFLVRGNIFETKSISGPYVQIGDFKKPPITWLSHLKPNKNYHLLGSTSLSDFFSGISDGNIPLRGTRF*

>*Tetrodontophora bielanensis* (Collembola) AT [GenBank:GAXI01141587.1]

…GTLVCLFLAGWAISFGIGFPQDQQNNGNPRTN***R***GSFQLQNSKLSTARGF**G*KR***DDGLIQSQSFSPWNLYEDNVLHSFAGKTSYPKNMLLEKIQNNPEIVRLIVEKLIDVSGDDELTTDDLLKRNTY*

>*Podura aquatica* (Collembola) AT

***M***TRVLLDIFVLSLVLVLTLSYPQQETQLIGQRNGNPRSN***R***GFQIQNAKLSTARGF**G*KR***GGVEESMGSFGVPISRGQGRLETNLQFGSDAKPNIPNNWLVEEIQVNPETARLIIEKYLNENKDGELTADEILRKTWSN*

>*Folsomia candida* (Collembola) AT [GenBank:GASX01091537.1]

***M***SKTVQYVLVCWLLTLAVVACSTATRTGYPRSN***R***GFKSQGLSTARGF**G*KR***DPGLSLGQGVFAGLQQPGQQQQANVEGLLSPNFDLTRAYPNSWLIEELQNNPEAAKLIVDKVIDENGDGELTPDELFRRIYF*

>*Bourletiella hortensis* (Collembola) AT

***M***AKALCTVLLLGWAVAMVLATASGRSSYPRTN***R***GFKNAQLSTARGF**G*KR***DRDVPISLQQEGVFTSDARTYPTTWLIEEIQNNPEMARMVVERFVDENGDGELTPGELYRKLLY*

>*Orchesella cincta* (Collembola) AT

***M***SRFYLHFAILGLIVGMTVATGSSVNTGRSGYP*R*SN***R***GFRNSQLSTARGF**G*KR***DSGSLVSNSRSSQGLSLANYEGFLPVVFDSSRTNYPTSWLVELLQNNPDVAKFIVDRLVDENGDGELTPSELT***RR***ACY…

>*Pogonognathellus sp.* (Collembola) AT [GenBank:GATD01092319.1]

***M***STRLVQFLVLLGCAAAIVLATSNSGRSGYPRPN***R***GFKNAQLSTARGF**G*KR***DGRLLVGASQPLIPGLSPSSLDGILPLNFDSRTSYPTTWLIDEIQNNPEVARMIVERYVDENGDGELSPDELF***RR***SYY*

>*Sminthurus viridis* (Collembola) AT [GenBank:GATZ01100231.1]

***M***SKVICYMVLMSWALAVVLASASTSGGRASYPRTN***R***GFKNAQLSTARGF**G*KR***DRDAVLGNAQQQQMQQPLQMLEELFSSNARSSYPNAWLVEELQSNPEMARLIVERFIDENGDGELTPDELF***RR***VYF*

>*Jordanathrix leptothrix* (Collembola) AT

***M***SKVLCYMLLLSWAVAVVLATASGSGGRSNYPRTN***R***GFKNAQLSTARGF**G*KR***DRDATLANLQQQHFNFQQQQQQPTPQQLLQQSNLQQQNLEAILTGEPRSSYPAGWLIEELQTNPEMARLIVERFIDENGDGELTPEELF***RR***VYF*

>*Campodea augens* (Diplura) AT [GenBank:GAYN01143380.1]

***M***TAVVFLALVLSALSASSNDRGTNRAVRTQ***R***GFKSSSLSTARGF**G*KR***DGSPLPLPLLLRLNAFRDSDRSASGSGERRDSFPVSWFAEELQNNPELARIVVEKFIDENGDGELTPDELLLPFF*

>*Lepidocampa weberi* (Diplura) AT

***M***SCSLRFPVHLVPLLLVLGLLLHSALSAAASEGRGTSQGGRSVRTQ***R***GFKSSALSTARGF**G*KR***DNGAVPLPIPILLRLNALRDNEHSGSASAE***RR***DSFPVSWFAEEVQSNPELARIIVDKFMDENGDGELTPDELLSRFF*

>*Occasjapyx japonicus* (Diplura) AT [GenBank:GAXJ01110145.1]

***M***GSSSPSPLRLMVLVVLLGAVLHTATARPARSPSQQSSGGGGGGRSGVRTQ***R***GFKNASLSVARGF**G*KR***NYDPDVAAATLAAAIDRSGERGNSFPVSWLVEELQSNPELARLLVEKFVDENGDGELTAEELLSRFY*

>*Catajapyx aquilonaris* (Diplura) AT

***M***GLSIRLMALAMVLSVVVQSACARPTGSSSGSTGRGGG***RR***DGNPVRTQ***R***GFKNASLSVARGF**G*KR***NYDPDMAAAIASAAERNGVQADRMDSFPVSWLVEEMQSNPELARLIVEKFVDENGDGELTAEEILSRFY*

>*Pedetontus okajimae* (Archaeognatha) AT

***M***VRFVFCCAAVVLLAVYVSGAPYNKSDKSRS***RR***GFKNSALSTARGF**G*KR***GEVGDGGGGSDAIKYYFDRQESFPSSWLVEEIQGNPELARVVVDKFVDENQDGELTADELLGLRGY*

>*Machilis hrabei* (Archaeognatha) AT [GenBank:GAUM01018582.1]

***M***MVRFVVCCAAIILLAVYVSAAPYNKSDKSRS***RR***GFKNAALSTARGF**G*KR***AQVGDGGGGSEAMKYYFDRQESFPVSWLVEEIQENPELARIVVDKFVDENQDGELTADELVGQRGY*

>*Meinertellus cundinamarcensis* (Archaeognatha) AT [GenBank:GAUG01028875.1]

***M***VRLFACCAVVVLVAACVVAAPYDKSTDKSRA***RR***GFKNAALSTARGF**G*KR***GDVGDSGGGSVSEIPKSYFYRQESFPSNWLVDEIQGNPDLARIIIDKFVDENQDGELSADELLGPRGF*

>*Machilontus* sp. (Archaeognatha) AT

***M***VRFFACYAVVMLLTACVLAAPYDKSDKSRA***RR***GFKNSALSTARGF**G*KR***GDLGDSGGGSGTGSEVPKFYFYRQESFPSNWLVDELQTNPELARIIVDKFVDENQDGELTADELLGPRAY*

>*Tricholepidion gertschi* (Zygentoma) AT [GenBank:GASO01019342.1]

***M***MQASLCLPVVVMALVVASWVACATPTQIRTDPRAI***R***GFKNSELATARGF**G*KR***DYDMSGLYSDKSDSFPVEWFVEEIQNNPELARIVVHKFIDENQDGELTADELLGRAY*

>*Thermobia domestica* (Zygentoma) AT [GenBank:GASN01031927.1]

***M***RLAVCLTVIMFAILATCATPGPYENKP***RR***TI***R***GFKNVALSTARGF**G*KR***DYDVSGGVYGGERPDSFPIDWFVEELQNNPELARIIVHKFIDENQDGELTADELLGRVY*

>*Atelura formicaria* (Zygentoma) AT [GenBank:GAYJ01311722.1]

***M***RVAVCVTLVLWAVLATCGAPTRAPEKPRKA***R***SFREIALSTARGY**G*KR***DGGEMLLERPARASLPNFIPYGY**G*KR***SESGVSIDERPESFPVDWLVEELQTNPELARIIVRKFVDENQDGELTTDELLG*

>*Nicoletia phytophila* (Zygentoma) AT

***M***RVAACFTVIMFAVMASCATPAPFQTKQRTI***R***GFKNLALSTARGF**G*KR***GFVPKELSTALRY**G*KR***DYEGANEIFEERPDSFPVEWFVEEIQNNPELARIVVHKFVDQNQDGELTADELLDRVY*

..............................................................................................

>*Lithobius forficatus* (Chilopoda) AT

***M***TCIPCVRLVAMLLLVGLSWCSETEDPVPAARVRQA***R***GFKNSALATARGF**G*KR***TAMLQDLVDSASTGTMDSGALSSEWLAEQMAHNPELAQLVVTKFVDTDGDGRLSFHELFPEL*

>*Hanseniella* sp. (Symphyla) AT1

***M***QVKLFVAIWALLLIAECMTEVRDFTVPRNVRQA***R***GFKNQGLSTARGF**G*KR***TSFSNDVADVAVLGRPRLSNDDVNQCMSNLLCRIMLIRNAERSSDVSNPLASLVSSEER*

>*Hanseniella* sp. (Symphyla) AT2

***M***HKKLFVAVLVMLLISASVCQERDFTFPRLAIMRQA***R***GFKNHGLSTARGF**G*KR***MYADENVSDFSGTAWSRQQAIQESIIQCFSNPLCREMLFRNIERINDLSHDGQISSLEQRHSP…

>*Hanseniella* sp. (Symphyla) AT3

***M***NVKTWASIYAMILITKTTSQERDGNDARES***R***GFKNHGLSTARGF**G*KR***TGDIMTLAGRTRFHPTEDITQCLYNPLCKLIMRHGPTGERDVKEVSGVIPMTGIDA…

..............................................................................................

no AT known from *Drosophila melanogaster*

>*Daphnia pulex* AT [[Dappu1_111909](http://genome.jgi.doe.gov/cgi-bin/dispGeneModel?db=Dappu1&tid=111909), GenBank:[EFX71302.1](http://www.ncbi.nlm.nih.gov/protein/321460258?report=genbank&log$=prottop&blast_rank=1&RID=XHMD63M9014)]

***M***KGKGAFLMVLAGWGLIGLMILTTAVEAAPHPADYTSSSVNNQRDFRS***RR***GFKTVGLATARGF**G*KR***APSLSNFNSFQDAAEQMMQQQEENPNSDPDVFPVDWLVNYLQNKPDVIRYMVEHLLDHNGDGQVTSQEMMTSLQQQRED*

Bursicon

>*Nipponentomon nippon* (Protura) bursicon-α

***M***NSSKHIGWYIRRIALLTCILVPACSSSDDCHITPVIHVLQYPGCVPKPIPSFACSGRCSSYVQVSGSKLWQTERSCMCCQESGIREASVGLFCPKAKDGESKFRKIMTRAPVECMCRPCTAVEEGSVMPQEVTSFLNNDVTLTGLSSLPLFN*

>*Nipponentomon nippon* (Protura) bursicon-β

…PSTIHITKEAYASDGRLERTCESEVAVNKCEGTCNSHLQPSAASPTGFNKDCQCCRESFLREREITLTHCYDADGNRLTGDNEKMEIKLREPSECKCYKCGQDPANVAKH*

>*Acerentomon* sp. (Protura) bursicon-α [GenBank:GAXE01132693.1]

…SLLLCSLPFLILLMDLCSCSDDCHITPVIHVLQYPGCVPKPIPSFACSGRCSSYVQVSGSKLWQTERSCMCCQESGIREASVVLFCPKAKEGEAKFKKIMTRAPVECMCRPCTAVEEGTVMPQEMASFINDMSLPGVSMPLFN*

>*Acerentomon* sp. (Protura) bursicon-β [GenBank:GAXE01136091.1]

***M***SFSVVLVILLSCIGFGHCNLSHSKEAACETLPSTIHITKESYTPDGRMERTCESEVAVNKCEGTCNSQLQPSAASPTGFNKDCQCCRESHLREREITLTHCYDADGNRLLGGESEKMLVKLREPSDCKCSKCGQESTTHSAH*

>*Filientomon takanawanum* (Protura) bursicon-α

***M***TRRKGSSIWFLPLLFLANPCFGSDDCHITPVIHVLQYPGCVPKPIPSFAxCSSYVQVSGSKLWQTERSCMCCQESGIREASVGLFCPKAKEGEAKFRKILTRAPVECMCRPCTAVEEGSVMPQEMASFISDMSLTGVSMPIFN*

>*Filientomon takanawanum* (Protura) bursicon-β

***M***RRLKIFVFCLFSIGISGSWAITHSKEAACETLPSTIHITKESYGADGRLERTCESDVAVNKCEGTCNSQLQPSAVAPTGFNKDCQCCRESFLREREITLTHCYDADGNRLLGGETEKME…

>*Anurida maritima* (Collembola) bursicon-α [GenBank:GAUE01050189.1]

***M***DGLCAAMIVLVTFGLGVRCDDCKLTPVVHILQSPGCQPRKIPSFACVGKCTSYVQVSGSKMWQTERSCMCCQESGEREAVVTLNCEHGPPGVRFQKVVTKAPVDCMCRPCTILDEGAILPQDMVGFMEGPPPASFAVISRRLQ*

>*Anurida maritima* (Collembola) bursicon-β [GenBank:GAUE01047623.1]

***M***DVRKFLLVLAFTEFLYSQGCEACETLPSSIHIIKELYDPSGNLQRTCEGDVAVSKCEGGCDSKVRPSAISHSGFIKECHCCRESTLRAREIVLRKCYDADGGPLQGEMGTFTINLREPVDCKCFRCGDSPINQ*

>*Tetrodontophora bielanensis* (Collembola) bursicon-α [GenBank:GAXI01147839.1]

***M***VELFRSKLSDKFISIGLNNFINYVIFLSILSTLVVGEDCRLVPVLHVLQTPGCNSMTIPSFACIGKCTSYVQVSGSKMWQTERSCMCCQESGEREATVTLTCPNAPAGNPRYQKVITKAPVDCMCRPCTTLEEGSILPQEMAGFLNADGSSQFSAMLYP*

>*Tetrodontophora bielanensis* (Collembola) bursicon-β [GenBank:GAXI01149566.1]

…MSEIHGQSGQLERTCEGEVAVSKCEGSCSSKVRPSALSHSGFIKDCQCCRESSLRVREVLLSKCFDPDGISIHGDKENFIISLREPADCKCFSCGELIGSRH*

>*Podura aquatica* (Collembola) bursicon-α

***M***YKTGSYSLSFISIFLLFSLILVKKIESSSEDCRLTPVVHVLQSPGCSPLKIPSFACIGKCTSYVQVSGSKMWQTERSCMCCQESGEREASVTLNCGKGKFQKVLTKAPVDCMCRPCTAVEEGSILPQEMAGFIDGSPPHSFAVISRKLN*

>*Podura aquatica* (Collembola) bursicon-β

…IYSSGSNMLERSCEGEVAVSKCEGSCESKVRPSAIAHSGFIKDCQCCRETVLRLREVTLEKCYDPDGGILTGENGKFLLKLKEPADCKCYKCGETLLNT*

>*Folsomia candida* (Collembola) bursicon-α [GenBank:GASX01008569.1]

…LAVSLVLLLSSSGVVLGDDCRITPVLHVLQTPGCSPMTIPSFACVGKCTSYVQVSGSKMWQMERSCMCCQESGEREATVTLSCPKAPPGSPKVQKIVTKAPVDCMCRPCTAVDEGSIMPQEMAGYMDAMDVGPLSIVPMRMQMSL*

>*Folsomia candida* (Collembola) bursicon-β [GenBank:GASX01083934.1]

***M***SLGFIFPLLLLAVADVQVQGTSACETLPSTIHIIKEIYAANGQMERTCEGEVAVSKCEGSCASKVRPSAVSHSGFMKDCQCCRESNLRSREITLTKCYDQDGKLLTGDKETYVISLKEPADCRCYRCGESAQIR*

>*Bourletiella hortensis* (Collembola) bursicon-α

…TLSLISWICLGGFYFFQFIFFASAAGEECRITPVLHVLQSPGCNPMTIPSFACVGKCTSYVQVSGSKMWEMERSCMCCQESGEREATVTLSCPKAPAGSPKVQKVVTKAPVDCMCRPCTAVDEGSILPQEMAGFAADGALLSMYSH*

>*Bourletiella hortensis* (Collembola) bursicon-β

***M***VLIWLLVVGAMTVCLSPLKSVQACETLPSSIHIIKEIYTTGGILERTCEGEVAVSKCEGTCPSKVRPSAVSHSGFFKDCQCCRETGLRIREVTLAKCFDPDGNTLTGDKETFVIKLREPADCKCARCGESTMSS*

>*Orchesella cincta* (Collembola) bursicon-α

***M***LLVQSLRRLILVWLWFVGLNQHLLEANGEDCRITPVLHVLQSPGCSPLTIPSFACVGKCTSYVQVSGSKMWQTERSCMCCQESGEREATVTLSCPKAPPGSPKSQKVVTKAPVDCMCRPCTTIEEGSVMPQEVSGFVEDGQILSMIPPNLVQL*

>*Orchesella cincta* (Collembola) bursicon-β

***M***RETRTSWSLIGMCMILFVVGEQISRGMGCETLPSSIHIIKEIYQPSGQMERTCEGEVAVSKCEGSCSSKVRPSAVSHTGFYKDCQCCRETTLRSRSVTLNKCYDPNGKTLTGDKEKYVINLKEPVDCRCYRCGDLPNTSSIST*

>*Pogonognathellus sp.* (Collembola) bursicon-α [GenBank:GATD01092092.1]

***M***VVQYSRWLILVSFWVGLVSINVVKTEDCRITPVLHVLQSPGCNPMTIPSFACVGKCTSYVQVSGSKMWQTERSCMCCQESGEREATVTLTCPKAPPGAPKIQKVVTKAPVDCMCRPCTIMDEGSVLPQEMAGYMDDGPPLNMIRMQL*

>*Pogonognathellus sp.* (Collembola) bursicon-β [GenBank:GATD01014521.1]

***M***GIPSKFIFLGTFFVCLMVWKCEACETLPSSLHIVKEIYGQGGQLERSCEGDVAVSKCEGSCSSKVRPSAISHSGFLKDCQCCRETTLRVREATLTKCYDPDGNTLKGEKETYVVKLREPADCKCYRCGDAIAGQ*

>*Sminthurus viridis* (Collembola) bursicon-α [GenBank:GATZ01100163.1]

***M***LDPYYSKWIFLTGVILISIWIAGISAEDCKLTPVYHNLQSPGCNSITIPSFACVGKCTSYVQVSGSKMWEMERSCMCCQESGERVATVTLNCPGAPAGSKVRKVMTKAPVECMCRPCTAVEEGSVLPQEIGGFSSDMQLRSMYTH*

>*Sminthurus viridis* (Collembola) bursicon-β [GenBank:GATZ01104404.1]

***M***SGLPFPWFVFACAAFGALLNIGGSKACETLPSSIHIIKEIYSQGGMLERTCEGEVAVSKCEGTCLSKVRPSGVSYSGFFKDCQCCRETSLRSREVILTKCFDPDGNALSGDKETYVTQLREPADCKCFRCGEAPLAK*

>*Jordanathrix leptothrix* (Collembola) bursicon-α

***M***LGLRFLNPFCFGGFYYLLIFLFSRVSGDNCKITPVLHVLQSPGCNPMTIPSFACVGKCTSYVQVSGSKIWEMERSCMCCQESGEREATVTLSCPKAPSGSPRQQKVVTKAPVDCMCRPCTAVDEGSILPQEMAGFQADGALLAMYSH*

>*Jordanathrix leptothrix* (Collembola) bursicon-β

***M***PDSQFRFSVFIMAAVVFTGSMLELEACETLPSSLHIIKEIYSQGGILERTCEGEVAVSKCEGTCPSKVRPSAIAHSGFLKDCQCCRESNLRVREATLPKCFDLDGNTLSGDKETYVIKLREPADCKCYRCGESTMTT*

>*Campodea augens* (Diplura) bursicon-α [GenBank:GAYN01136411.1]

…LIPSFACSGRCTSYVQVSASKIWQMERSCMCCQESGEREAVVSLTCRSSSDGQVYVMKVPTRAPFECMCRPCTAIDEASVMPQELAAVVARGQTPPFQYP*

>*Campodea augens* (Diplura) bursicon-β [GenBank:GAYN01132102.1]

…EEYSTDGNLIRNCEEEVAVSKCDGACGSSARTSVVNPSGYERHCQCCRESSLRERVVTLNHCFDSDGNRLVGELEKMTIKLREPVDCKCFACGHHD*

>*Lepidocampa weberi* (Diplura) bursicon-α

***M***LYLFAVAVSFLASSTLADDCKITPALYKIEYPGCDIRIIPSFACRGRCTSYVQVSGNKIWQLERSCMCCQESGEREATVDLTCRASPGGAPYIMKIPTRAPVDCMCRPCTALDEGSVVPQEIAGIVARGQSPPFPYP*

>*Lepidocampa weberi* (Diplura) bursicon-β

***M***QGASWCSKFLAVVWVILNPAFYHVEAGRSLAADSCETLPSAVHVFKEEYNEDGQLVRNCEGDIAVNKCDGACASHSLTSVVNPTGFEKHCQCCRETSLRERLITLSKCYDSDGGLIVGILGTMTIKLREPVDCKCVTCGHQ*

>*Occasjapyx japonicus* (Diplura) bursicon-α [GenBank:GAXJ01110249.1]

***M***LAALALGVTWLLLTSADDCKVTPVIHVLQFPGCVPKPIPSFACIGKCTSYVQVSGSKIWQTERSCMCCQESGEREATVSLYCPKAEEGDPRFRKVVTRAPVDCMCRPCTAVEESSVLPQEIAGFANDGPVPFSFP*

>*Occasjapyx japonicus* (Diplura) bursicon-β

***M***LVWSKVILAVVIQLQMSLGNESTLGGTCETLPSTIRITKEQYNEGGQLERTCEAEIAVSKCEGTCNSQVQPSVVNPSGFLKDCHCCRETSLRERHVTLSNCFDGDGKRLTGELESLAVRLREPADCKCFRCGDHS*

>*Catajapyx aquilonaris* (Diplura) bursicon-α

…CPNAEEGEPKFRKVVTRAPVDCMCRPCTAVEESAVLPQEIAGFANDGPVPFTFT*

>*Catajapyx aquilonaris* (Diplura) bursicon-β

***M***VWIFSLAVLVVLQLQVSIGNESTLGGTCETLPSTIHITKEQYGEGGQLERTCEAEIAVSKCEGTCNSQVQPSVVNPS…

>*Pedetontus okajimae* (Archaeognatha) bursicon-α a

***M***MLVLVLTTLASLLVVPGLADDCKVTPVIHMLHYPGCVPKPIPSFACTGRCTSYVQVSGSKLWQMERSCMCCQESGEREASVVLICPRARSGEPRVRKVTTKAPLDCMCRPCTAVEESAILPQEIASFIDFGRLGK*

>*Pedetontus okajimae* (Archaeognatha) bursicon-α b

***M***MLVLVLTTLASLLVVPGLADDCKVTPVIHMLHYPGCVPKPIPSFACTGRCTSYVQVSGSKLWQMERSCMCCQESGEREASVVLICPRARSGEPRVRKVTTKAPLDCMCRPCTAVEESAILPQEIASFIDEGPLAFLGNKFQ*

>*Pedetontus okajimae* (Archaeognatha) bursicon-β

***M***LQTSAVSALFLLTSSTFMMSSLGWALQREGSCETLPSNIHITKEVYDAEGKLERTCEGDVAVTKCEGTCVSQVQPSVVTPTGFLKECHCCRESFL…

>*Machilis hrabei* (Archaeognatha) bursicon-α [GenBank:GAUM01181183.1]

***M***WFLEIAMLATLVVVPAMTDDCKVTPVIHMLQYPGCVPKPIPSFACTGRCTSYVQVSGSKLWQMERSCMCCQESGEREASVALFCPRARAGEPRFRKVITKAPLDCMCRPCTAVEESAILPQEIASFIDEGPLAFLGNKYQ*

>*Machilis hrabei* (Archaeognatha) bursicon-β [GenBank:GAUM01180579.1]

***M***MPHTPAIAALFIWTTSTFMMPSLGWALQREGSCETLPSNIHITKEVYDTEGKLERTCEGDVAVTKCEGTCVSQVQPSVVTPTGFLKECHCCRESFLRERLVTLTHCFDQDGNKMQDALETYDVKLREPAECQCYKCGEIPLH*

>*Meinertellus cundinamarcensis* (Archaeognatha) bursicon-α [GenBank:GAUG01228176.1]

…AALLASPTLADDCKVTPVIHMLQYPGCVPKPIPSFACTGRCTSYVQVSGSKLWQMERSCMCCQESGEREASVALFCPKARSGEPKFRKVITKAPLDCMCRPCTAVEESAILPQEIASFIDEGPLAFLGSRFQ*

>*Meinertellus cundinamarcensis* (Archaeognatha) bursicon-β [GenBank:GAUG01200861.1 + GAUG01219440.1]

MSPVSAALALFLCASGTLMLPSVGWALQREGTCETLPSNIHITKEVYDSNGQLERTCEGDVAVTKCEGTCLSQVQPSVVTPTGFLKECHCCRESFLRERLVTLTHCFNQDGTKLTGELETFDIKLREPAECQCYKCGEIPQQ*

>*Machilontus* sp. (Archaeognatha) bursicon-α

***M***ISAMWLLLWIVVLATLCAVPTLADDCKVTPVIHMLQYPGCNPKPIPSFACTGRCTSYVQVSGSKLWQMERSCMCCQESGEREASVALFCPRARSGEPKFRKVITKAPLDCMCRPCTAVEESAILPQEIASFIDEGPLAFLGSRFQ*

>*Machilontus* sp. (Archaeognatha) bursicon-β

***M***NHLCAVIVVFVCASGTLLLPSLGWALQREGSCETLPSNIHITKEVYDGNGQLERTCEGDIAVTKCEGTCLSQVQPSVVTPTGFLKECHCCRESFLRERLVILSHCFDQDGTKLTGSLETYEIKLREPSECQCYKCGEVPQQ*

>*Tricholepidion gertschi* (Zygentoma) bursicon-α [GenBank:GASO01237852.1]

***M***RLAQQTTYTGWK***M***YCWVCACMMMMTMMAVVAADECQVTPVIHVLQYPGCVPKPIPSFACTGRCTSYLQVSGSKIWQMERSCMCCQESGEREASVSL

FCPKAKPGERKFRKVITKAPLECMCRPCTGVEESSVIPQEIAGYADEG…

>*Tricholepidion gertschi* (Zygentoma) bursicon-β [GenBank:GASO01234912.1]

***M***SPQSPVWLVSPLVIFLFFSLMSATEAEGEEACETLPSEIHIIKEEFDELGRLQRTCNGDIAVNKCEGTCNSQVQPSVITPTGFLKECYCCRESFLRERVISLTHCYDPDGGRLHGARESLDVKLREPANCKCFKCGDFSR*

>*Thermobia domestica* (Zygentoma) bursicon-α [GenBank:GASN01375041.1]

***M***SPIHKLRQNGSEWRRKSSTYILLLSTIVHLTLADECEVTPVIHVLQYPGCVPKPIPSFACTGRCTSYIQVSGSKLWQMERSCMCCQESGEREATVSLFCPKAKPGERKFRKVITK…

>*Thermobia domestica* (Zygentoma) bursicon-β [GenBank:GASN01389553.1]

***M***PSTLYCTLYAFLVVLLLLLSVASTNAEGEEACETLPSEIHIVKEEYNELGRLQRTCNADVAVNKCEGTCNSQVQPSVITPTGFLKECYCCRESYLRERTITLPHCYDPDGARLHGDMESMEIKLREPVDCKCYKCGEFSR*

>*Atelura formicaria* (Zygentoma) bursicon-α [GenBank:GAYJ01285991.1 + GAYJ01294051.1]

***M***RHQAQGLHSGRQMLCFLLAPLLALAVADECQVTPVIHVLQYPGCVPKPIPSFACTGRCTSYLQVSGSKIWQMERSCMCCQESGEREASVSLFCPKAK…

…KFRKVTTKAPLECMCRPCTGVEESAVVPQEIAGYTDEGQFNGHFRTTSL*

>*Atelura formicaria* (Zygentoma) bursicon-β [GenBank:GAYJ01031398.1]

***M***ALLMSPLALVPLALLLSQLAATDAAGEEACETLPSEIHIVKEEYDELGRLQRTCNGDIAVNKCEGACSSQVQPSVITPTGFLKECYCCRESFLRERTITLENCFDPDGFRLRGDKDSMDVKLREPTDCKCYKCGDFSR*

>*Nicoletia phytophila* (Zygentoma) bursicon-α

***M***RFLLCAVLLVAAMTRQSTADECQLTRVMHVLQYPGCMPKPIPSYACTGRCTSYLQVSGSKIWQMERSCMCCQESGEREASVTLFCPRDKQKERYRKIITKAALECMCRPCTGIEENSVIPQEIAGYSDEGPLTDHFRRSS*

>*Nicoletia phytophila* (Zygentoma) bursicon-β

***M***SPPSVWSTVLVSHLAVLVVCHVTASDAEPQEACETLPSEIHIVKEEYDELGQLQRTCNGDIAVNKCEGACSSQVQPSVMTPTGFLKECYCCRESLLRERVISLTHCYDPDGFQIRGDKEVMEVKLREPTDCKCYKCGDFSG*

..............................................................................................

>*Xibalbanus tulumensis* (Remipedia) bursicon-β

…EYYDTAGQLERTCEGNIAVNKCEGTCTSQVQPSVVTPSGFLKECQCCREEFMKEREVSLDSCYDPDGHQLIGGLGQMVVKLQEP…

>*Anaspides tasmaniae* (Malacostraca) bursicon-α

…APVIHILSYPGCTSKPIPSFACQGRCTSYVQVSGSKIWQTERSCMCCQESGEREASVTLTCPKARPGEPRIRKVLTRAPIDCMCRPCTEVEESSVMAQEIANFMDESAMGSVPFLK*

>*Anaspides tasmaniae* (Malacostraca) bursicon-β

…TIHVTKEEFDEAGRLVRTCEEDLAVNKCEGGCVSKVQPSVNTPSGFLKDCRCCRETHLRSREVLLTHCYDGDGGRLTDADDRSKILVQLREPADCKCYKCGEAN*

>*Lithobius forficatus* (Chilopoda) bursicon-α

…PSFACQGRCMSYVQVSGSKLWQTERSCMCCQEMGIREANVTLHCPQDRAGEPRFRKITTRAPVDCMCRPCTAVEKH…

>*Hanseniella* sp. (Symphyla) bursicon-α

***M***ISSWIKCLSAVVILSCVKEVAADECHITPVIHVLQHPGCVPKPIPSFACQGRCSSYVQVSCDK…

>*Hanseniella* sp. (Symphyla) bursicon-β

…LIRTCEGDIAVTKCEGTCISQVQPSVVTASGFVKECHCCRESTLRERDIVLARCFDESGDVIKSGSRSRMSVRLREPA…

>*Eudigraphis takakuwai nigricans* (Diplopoda) bursicon-α

***M***AWVGTLLCVLIGLCLATEKSKGDECHASPVIHVLQHPGCIPKPIPSFACQGRCTSYVQVSGYKLWQTERSCMCCQEMG…

>*Eudigraphis takakuwai nigricans* (Diplopoda) bursicon-β a

***M***TKVPNEMFILVISLIAGQSGMVSAAATRSLAPTESSCETLPSTIHISKEENDEYGQTIRTCEGDVAVNKCEGTCQSQLRPSVFTTSGFIKECECCQETHLREREIVLTHCYDAHEQLLTNEMSSMLIRLREPAQCHCFKCGDN*

>*Eudigraphis takakuwai nigricans* (Diplopoda) bursicon-β b

***M***TKVPNEMFILVISLIAGQSGMVSAAATRSLAPTESSCETLPSTIHISKEENDEYGQTIRTCEGDVAVNKCEGTCQSQLRPSVFTTSGFIKVIRKFKQCIK*

..............................................................................................

>*Drosophila melanogaster* bursicon-α [[FBgn0083529](http://flybase.org/reports/FBpp0083529.html)]

***M***LRHLLRHENNKVFVLILLYCVLVSILKLCTAQPDSSVAATDNDITHLGDDCQVTPVIHVLQYPGCVPKPIPSFACVGRCASYIQVSGSKIWQMERSCMCCQESGEREAAVSLFCPKVKPGERKFKKVLTKAPLECMCRPCTSIEESGIIPQEIAGYSDEGPLNNHFRRIALQ*

*>Drosophila melanogaster* bursicon-β [[FBpp0080207](http://flybase.org/reports/FBpp0080207.html)]

***M***HVQELLFVAAILVPQCLRALRYSQGTGDENCETLKSEIHLIKEEFDELGRMQRTCNADVIVNKCEGLCNSQVQPSVITPTGFLKECYCCRESFLKEKVITLTHCYDPDGTRLTSPEMGSMDIRLREPTECKCFKCGDFTR*

>*Daphnia pulex* bursicon-α [[Dappu1_312014](http://genome.jgi.doe.gov/cgi-bin/dispGeneModel?db=Dappu1&id=312014), GenBank:[EFX87546.1](http://www.ncbi.nlm.nih.gov/protein/321476586?report=genbank&log$=prottop&blast_rank=1&RID=XHPMZ5DJ015)]

***M***FLFVGLVSVIRADECQLTPVIHVLQYPGCIPKPIPSFACTGKCTSYVQVSGSKLWQTERSCMCCQESGEREATVSLLCPKAAPGEPKLRRVVTRAPVDCMCRPCTALEESAVMPQEIARFLDDGSFPFKL*

>*Daphnia pulex* bursicon-β [[Dappu_312013](http://genome.jgi.doe.gov/cgi-bin/dispGeneModel?db=Dappu1&tid=312013), GenBank:[EFX87749.1](http://www.ncbi.nlm.nih.gov/protein/321476789?report=genbank&log$=prottop&blast_rank=1&RID=XHPY6RV6014)]

***M***FWYVLIILLGAGSSRETLASKTNLMSGTCETLPSTIHITKEEYTDGGILSRTCEGDIGVAKCEGSCSSQVQPSVVHPSGFLKECMCCRESFLRERVVTLTHCYDANGNRLTGKSSSLDVKMREPADCKCFRCGDSAE*

CAPA/Pyrokinin (CAPA/PK)

>*Nipponentomon nippon* (Protura) CAPA/PK

***M***DPRLWHTATYLKWLLFVLLLGYAKGLRET***KR***HQGLIPFPRV**G*R***GGSGLIPFPRV**G*R***TSPFRYYDYGGG**G*R***SASSYSSHLSD***KR***QGSLIPFPRV**G*KR***QNQNLIPFPRV**G*R***SSLPAVLLYSPFYSEDIAEITARNTGPTTLQSNSENPFAHLSRQSLADLLMAIN**G*K***QDSSWTGPRM**G*R***Q***RR***DASAEPDAYPDAYPEADADPARQQF**G*RR***QLAALLREMPWAPGQRNGAENTGGP***KR***QQTNFTPRL**G*R***SSEEYGYDDSSTVFSPRL**G*K***DGEGEDTDDYFDLATAFPSSSLMMERGSSA***RR***LSQRYSRSAHQNELTQERTGSNS**G*RR***TKDEADDYEVVTEPGVHGGGSPFSPRL**G*R***TTPIAPRL**G*K***EK*

>*Acerentomon* sp. (Protura) CAPA/PK [GenBank:GAXE01024165.1]

***M***DLRQWYNTATYLKWLLVLFILGFAKGLRET***KR***QNLIPFPRV**G*R***GGSGLIPFPRV**G*R***TTNLRYYDYD***KR***QGSLIPFPRV**G*KR***QNLIPFPRV**G*R***SQLPPLMIYSPYLSEEMAQLTSTSQGPSSLLQSRSIDNPFSHITSQSNSMENPFSHLSRQSIADLLLAIESVNS***KR***EDSGMWFGPRL**G*R***N***RR***DASADPDAYPDAYPEADADPANANRQF**G*RR***QLAALLREMPWAPGTRNAADNGP***KR***QQTNFTPRL**G*R***SSEETYGYDDSSTIFSPRL**G*K***DGDEPVDDDYDLVTAFPSSGLLSRASGSSD***RR***LTSRYSRSTHNDMIRDSGLANN***RR***LRNREEQDDTPVTAPGIHEGNSPFSPRL**G*R***TTPIAPRL**G*R***EKK*

>*Filientomon takanawanum* (Protura) CAPA/PK1

***M***DLTLCHCSANLKWLLFVMLLGFSKGFRDI***KR***QNLIPFPRV**G*R***GGSGLIPFPRV**G*R***TSKFRYYDYVRGHQSD***KR***QGSLIPFPRV**G*KR***QNLIPFPRV**G*R***SSTAPLLATSPHHNKAISRQISRKPTVPSDLEIQNQFTRLSELSLLDIMRAIAAVNS***KR***EDNGLWFGPRL**G*R***T***RR***DASADPDAYPEAYAEADADPANANRQF**G*RR***QLAALLREMPWAPGTRNAADTGP***KR***QQTNFTPRL**G*R***SSEESMGYDDSSTIFSPRL**G*K***DGEEDEDDFFDLHTSFPSSPLLMDKEASVAHRFQPRYSRSTNKMMRDTDLPNSERISKDEAINQSQYTTVTAPGTHDGGSPFSPRL**G*R***TTPIAPRLGAE*

>*Filientomon takanawanum* (Protura) CAPA/PK2

…PDAYPEADADPARQQF**G*RR***QLAALLREMPWAPGQRNGAENTGGP***KR***QQTNFTPRL**G*R***SSEEYGYDDSSTVFSPRL**G*K***DGEGEDTD…

>*Anurida maritima* (Collembola) CAPA/PK a [GenBank:GAUE01054696.1]

…E***RR***TFTPRL**G*R***AYSGDGESHFSSEPYTPRL**G*R***SHEAVLAGEELGMSQRH***RR***DGTNE**G*R***NRQPKGQEDTNVGGAPFSPRL**G*R***DHINPRL**G*R***SSLDEHE*

>*Anurida maritima* (Collembola) CAPA/PK b [GenBank:GAUE01051518.1]

***M***NVFLCLLTFSSVILVHGQQSDKGTSMIPFPRI**G*R***ADPRLFAD**G*KR***DGNGNGLIPFPRI**G*R***AEGFQEM***KR***QLIPFPRV**G*KR***PYWRIPLDFEEYTFPLGESSVP**G*R***VPDMVFEPSEDTEGE***RR***HLGNKNGGGVWFGSRF**G*RKKR***SIESQTSHERDSIDSRLLLKILHNFNWAIVPIKGKTEIV*

>*Tetrodontophora bielanensis* (Collembola) CAPA/PK [GenBank:GAXI01154264.1]

***M***NLILSLATLTSVAIVHGQSGDKGIIPYPRV**G*R***PDRLYMD**G*KR***DGNTMSPGLIPFPRI**G*R***SDGNSIHE**G*KR***QLIPFPRV**G*KR***PFWHIPMDPYMVDYEEFSFPVTDGGSIDQNMLRNGLRGMSTDPNAIEENDTE***KR***SSNNNGGGGMWFGPRL**G*KR***R***KR***SIETDSSTEHSRDSLDTRTLLKILHNFNWAIVPIKE***RR***ILTPHI**G*R***SSDEIGIH***RR***STPPFIPRL**G*R***SKEVASSEVDMEEEVHV**G*R***FSKNNNAEQHRKATQSTPSESTNIDNGGSTAPFSTRLSSDHLTPRLAHSVED*

>*Podura aquatica* (Collembola) CAPA/PK a

***M***MHIIISLIALSSLIIVHGQQSDKSTLIPFPRI**G*R***SGSEPRFFMEA***KK***SGGSNTPGLIPFPRI**G*R***SEGIHE**G*KR***QLIPYPRV**G*KR***AYFRVPIDPYMVEYDENIELSPDPSTDDFEGD***KR***SGTTSSNNNGMWFGPRL**G*KRKR***RSVEDTEKTISTSDTLLKILHNFNWAIVPIKE***RR***TFTPRL**G*R***SSDEASSTRIHHRSQPYTPRL**G*R***SKERASNSEDDEDENRSKNDKNSRSTDETIGGVPFSPRL**G*R***DHFNPRL**G*RR***SSEDGEQNLN*

>*Podura aquatica* (Collembola) CAPA/PK b

***M***MHIIISLIALSSLIIVHGQQSDKSTLIPFPRI**G*R***SGSEPRFFMEA***KK***SGGSNTPGLIPFPRI**G*R***SEGIHE**G*KR***QLIPYPRV**G*KR***AYFRVPIDPYMVEYDENIELSPDPSTDDFEGD***KR***SGTTSSNNNGMWFGPRL**G*KRKR***RSVEDTEKTISTSDTLLKILHNFNWAIVPIKGNSVN*

>*Folsomia candida* (Collembola) CAPA/PK a2 [GenBank:GASX01092562.1]

…VPVKE***RR***NFTPRL**G*R***SSHEGFTVHQRGQPFSPRL**G*R***SGDMASPSIDEDEELETRSHV***KR***RPPYRDTTPDPIFDLRPRAPLSPRL**G*R***SDPFSPRL**G*R***ADPFSPRL**G*R***SGTESTQPDDSRDRK*

>*Folsomia candida* (Collembola) CAPA/PK b [GenBank:GASX01089394.1]

***M***NFFLALVTLISVAIGIANAQQGEKGNSIIPFPRV**G*R***SGNSPGLIPFPRI**G*R***DGGFHEM***KR***QLIPFPRV**G*KR***AFWHLPVESAYYTEYEDINPLMVDPNFLPPPPSFQVESSPSEDGEVD***KR***GGTPATSSAGMWFGPRL**G*RR***K***KR***SVVDTVAESSEHRGDTTIDMKPLLKMLHNSNWAIIVPVKG*

>*Bourletiella hortensis* (Collembola) CAPA/PK

***M***NLLLSLITLASVAIVHGQQGDKSTSIIPFPRI**G*R***ADRFYMSD***KR***SSGSPGLIPFPRI**G*R***DGSGIHES***KR***QLIPFPRV**G*KR***PYWHYAVDPFFVEYEDLQYPIVDSVSPVLATPESTSTGLEENDAE***KR***NGANGGGMWFGPRL**G*R***K***KR***SIETDSSAERSRDTLDTRTLLKILHNFNWAIVPVK**G*R***SSEEVTVHHRTPAYSPRL**G*R***SREGSSAEFELEEDNRPRHS***R***AGSNSTPAPSDDSSSSSSTSQASPPFSPRL**G*R***DHFSPRL**G*R***SGEDQK*

>*Orchesella cincta* (Collembola) CAPA/PK

***M***NILLAFVTLTGISCAHGQEKGNSIIPFPRI**G*R***SNGNAMSPGLIPFPRI**G*R***DGGGFHEV***KR***QLIPFPRV**G*KR***FPMYMDPYYAEYEAQYAPWYDQTGEMVASYSAADKDDNVDVD***KK***SSPSPNAGGGMWFGPRL**G*KR***K***KR***SIEESSSEHARDTLDTRTLVKILHNFNWAIVPVKE***RR***TFTPRL**G*R***SSGEISVHTRAQPLTPRL**G*R***SREMVSAELDVDEDGAS***R***YARNEEHSHIKGTQQPPIEVSGPPFSPRL**G*R***ADHYSSRLRSGDDHE*

>*Pogonognathellus sp.* (Collembola) CAPA/PK1 a [GenBank:GATD01014935.1]

***M***SFIHAFVTLASISAGIVYGQQGEKGNSIIPFPRV**G*R***SDNRYFMAE***KR***NGNSPGLIPFPRI**G*R***SDSNGPHEM***KR***QLIPFPRV**G*KR***PYWHYPVDAYSAYEYEDYPYMVDPNGSSSMMNSPMDPNVMNTDEIDND***KR***SNNAEKGMWFGPRL**G*KR***K***KR***SVSESSDSSYEHSRDSVDTKTLVKILRSFQWAIVPIRE***RR***TFTPRL**G*R***NSDEVVVHSRAQPFSPRL**G*R***SREVASVELDLEEKSQSRNSRNDSE**G*R***GHDKGTEPPPSDVLAPRLVRDHFSPRL**G*R***SGEDHE*

>*Pogonognathellus sp.* (Collembola) CAPA/PK2 a [GenBank:GATD01096836.1]

***M***SFIQIFVTLASISAGFVYGQQGLKGNSIIPFPRV**G*R***SENRFFMAE***RR***NGNSPGLIPFPRI**G*R***SDSNDLHEM***KR***QLIPFPRV**G*KR***PYWRYPVEAYNNGAFEYEDYPFMVDPNGSSPMINAPGEPNAMNADEIDND***KR***NAEKSVWFGPRI**G*KR***R***KR***SISESTDSSYE***RR***DSTIDTKTLVNILHSFKWAIVPIRE***RR***TFTPRL**G*R***SSDEVVVHSRAQHYSPRL**G*R***SREVASVELDVEDKGQS***R***ASRSDNDVPTHDKDNETPPTVVYTPRL**G*R***DQYTPRL**G*R***SGEDHQ*

>*Sminthurus viridis* (Collembola) CAPA/PK1 a [GenBank:GATZ01004120.1, GATZ01004119.1]

***M***NLLLGSLTLFSLAVVHGQGEKSTSIIPFPRI**G*R***SGVDRFYMSD***KR***SSNVSPGLIPFPRI**G*R***DGSGIHEA***KR***QLIPFPRV**G*KR***PYWHYPVDPYFFEYEDIQYPIDAVNPNSLLTPSSDSSSTGIEENDAE***KR***NGAGGGGMWFGPRL**G*KR***R***KR***SVEMESSTERTREELDTKTLVKILRNFNWAIVPIKS***RR***TFTPRL**G*R***SSEEVGTHHRNQPLTPRL**G*R***SREVSSVELDLDDDNQSRY**G*R***SGTSSSGHGNPTASPNNDDTTPAAPFSPRL**G*R***DHFSPRL**G*R***SGEDRD*

>*Sminthurus viridis* (Collembola) CAPA/PK2 b [GenBank:GATZ01005692.1]

***M***NLSLGFITLCCLALIHCQGDKSASNIHFPSI**G*R***DGTGIREVKGQLIPFPRA**G*K***QTYWHYPGDPYVFDYEDIQYPIAAVNPSSHPSDPSSPGMEERFAE***KR***DSAGSGGMWFGPRM**G*KR***R***KR***SIELESSSERTREEVDTKTLVKILRNFNWAIVPLKSKNLSSRNPTEN*

>*Jordanathrix leptothrix* (Collembola) CAPA/PK1 a

…E***KR***NGAGNTNAMWFGPRL**G*RRKR***SIEADSRDNSIDSRTILKILHNFHWAVVPIKE***RR***TSFTPRL**G*R***SSKENSHHSSPPYSPRL**G*R***SGEISSVELDTDEESQN***R***YTRSGTNGNSGTGHSTQPPSSNDDTTGSPPFSPRL**G*R***DNFSPRL**G*R***SSSPVDDHE*

>*Jordanathrix leptothrix* (Collembola) CAPA/PK2 b

***M***NLLLGFLALVSLAYVYGQQGEKSSSMIPFPRI**G*R***ADRFFMSD***KR***SQGSISPGLIPFPRI**G*R***SGIHEN***KR***QLIPFPRV**G*KR***PYWHYSLEPNFFEYEDLQYPVVDGSSPNSLLTPPESSNTGVEESEVD***KR***NGAGSNGMWFGPRL**G*K****

>*Campodea augens* (Diplura) CAPA [GenBank:GAYN01005928.1]

**M**PGTVVFHLCVLLVLVQICWGDSIQLH***KR***NNIIPYPRV**G*R***SDSTFLDE***KR***TGPGLIPFPRV**G*R*G*KR***GQNTLIPFPRV**G*R***SSLLVVPFNSDGLEDQQFWDPAHSVEDRLEEQEDVFDLHDTFQ***KR***NGGTVNGGGMWFGPRL**G*RRKR***STEGQDGEGNDDYSSGQLSHSGHEKHSRSYGEV*

>*Campodea augens* (Diplura) PK [GenBank:GAYN01016646.1]

***M***QHFNSLCLILLLLGVRSYCSALPSLSLKTVLLGFPRSSEEQAPLFPVFSAGSADDSYEDSYENPYFSLSA**G*KKKR***SPESEIESDNQPRKLEAQELADLLKDTPWALIAVKDFSTP***KR***TYNTFTPRL**G*R***SSEESFSVESRGNQFSPRLGQ***R***SRPYNPADIGFGGSADYGISPTFVPRL**G*R***SQDSVSRDDNRHKDSSNTKIAPRLSRIPMSPRL**G*R***SIY*

>*Lepidocampa weberi* (Diplura) CAPA

***M***LGVRLEIMVQVCLVVLALNMAWGESSSIQ***KR***NTIIPYPRV**G*R***ADVNFMAE***KR***GGGPGLIPFPRV**G*R***AGTMAE***KR***GSNTIIPFPRV**G*R***SAMIVFPFNQDGGEETPFWTPSNSFEDQMNEQDEVIEIHDPYQ***KR***GTGPAAAGLWFGPRL**G*R***K***KR***SPEETDAEEDN**G*R***YT*

>*Lepidocampa weberi* (Diplura) PK

…PASSSLRSILFDLARAHDLSGGDDSSAEESLESALRLGGL***KKR***SLETEVESDNLPRKLEPEEIADLLKDTPWALIAVKEYSPAP***KR***AGYNTNFTPRL**G*R***SSEESLSLES***R***SNNRFSPRL**G*R***GHFFPAETGGVGDYGISPTFVPRL**G*R***SQGGLRDVRSKNSNAKLGPRINRIPLSPRM**G*K****

>*Occasjapyx japonicus* (Diplura) CAPA [GenBank:GAXJ01109829.1]

***M***QFVRGVLFFVVLVVASSCCDGQTERIQ***KR***NSIIPYPRV**G*R***SFYMEE***KR***GGPGGLIPFPRV**G*R***SFLDEDVFHEM***KR***QLIPFPRV**G*KR***QNTIIPFPRV**G*R***SSSTLYLVPVDDDDVAAAAWESEAVDGGDEDVQG**G*KR***NGAGASGAGGMWFGPRL**G*R***K***KR***STDAEYEAAP**G*R***QVKAEVVQVLKDTPWALISMK**G*R***DDPDLMEGHSGHLTHSRPAEEDSDNWTAPSGEGGHFPSSI**G*R***VPFVPESKNH*

>*Occasjapyx japonicus* (Diplura) PK [GenBank:GAXJ01108037.1]

…VAVVTIAGSEGIYVM***KK***NSSPSHGHTLYLLSRAANNGDQLDESGLLFR***KR***RSPEYSQEEDENI***RR***LDPADLAELLKDTPWALIAIKDYNTQ***KR***GGGNTVATNFIPRL**G*R***SFHQFSPRL**G*R***ADAQMEGDDADEEWDGSTTFVPRL**G*R***SRGSGSKDDNQGNSQD**G*R***SSSTKFSPRL**G*R***GNFSPRL**G***

>*Catajapyx aquilonaris* (Diplura) CAPA

***M***GFVRGALFFALLVAVSNCCDGET***KR***IQ***KR***QSIIPYPRV**G*R***SQWYMED***KR***GAPGLIPFPRV**G*R***SHLDEDVFHEM***KR***QLIPFPRV**G*KR***QNTIIPFPRV**G*R***SSAASTLYLVPLEEEEGLWETDVDNDDDSQQ***KR***NGAGATGPGGMWFGPRL**G*R***K***KR***SVDYDDHKSDVVQILKDTPWAVISMKGHNPEEMVEGHSVHNEDSEDWPAASEVGHFPSTIGEVPFVAKSKHH*

>*Catajapyx aquilonaris* (Diplura) PK

***M***EVLHVLISLAMVMAFTGSEGLYVM***KK***SSPSQHSATMVLIPATQEEDSGLLVR***KR***RSPEYAQDENDNI***RR***LDPADLAELLKDTPWALIAIKDYNTQ***KR***GGGNSLSTNFVPRL**G*R***SYHQFSPRL**G*R***SDVQFEEEEDNEEWDGSTTFVPRL**G*R***ASRGSGGSNNNNNNKED**G*R***GNSQQDISATTKFSPRL**G*R***GGFSPRL**G***

>*Pedetontus okajimae* (Archaeognatha) CAPA/PK

***M***NEGYLIFHAAILFLGGASFFGNTVDGMAAE***RR***GSLIPFPRV**G*R***SEYFMDS***KR***SSGLIPFPRV**G*R***SDMEMGEQAMHED***KR***QGLIPFPRV**G*KR***QSLIPFPRV**G*R***SSMSPYFY***RR***PLAYFISDGNSDDDMLELDFPATEV**G*KR***GGMWFGPRL**G*RR***D***RR***SIDTLVAGE**G*KK***EESMTLAELLKNTPWALIALKAGHSED***KR***QTPNFTPRL**G*R***ESGEEEGQTFEDRSTPYTPRL**G*R***SNENKSVANAPRW**G*R***QGEEDEERGSSFEDYDREDRSTVFSPRL**G*R***SVEPPSDHDLSKQQNSSKSQAKSH*

>*Machilis hrabei* (Archaeognatha) CAPA/PK [GenBank:GAUM01023456.1]

***M***NEGYLVCHVVVLILGSATFLTNTADGLAAE***RR***GSLIPFPRV**G*R***SEYFMDS***KR***SSGLIPFPRV**G*R***SDMEMGESAMHED***KR***QGLIPFPRV**G*KR***QGIIPFPGV**G*K***SSMTPYFY***RR***PLAYYVTDGNTDDDMLELDFPTSEVA***KR***GGMWFGPRL**G*RR***D***RR***SVDTVVTGE**G**E***KK***ESMTLAELLRNTPWALIALKAHSDE***KR***HTPNFTPRL**G*R***ESGEEDGQTYEDRSTPYTPRL**G*R***ESENRGVPFSPRL**G*R***QEDLEEDD***KR***SSFEDYDREDRSTVFSPRL**G*R***SVEPVHHQDLSKQQSTSKTEAKSH*

>*Meinertellus cundinamarcensis* (Archaeognatha) CAPA/PK [GenBank:GAUG01032439.1]

***M***NEGYIVFHSLVLLLGVSYFVHTADGLAAE***KR***GSLIPFPRV**G*R***SEFFMDS***KR***SSGLIPFPRV**G*R***SDMEMGEQAFHEV***KR***QGLIPFPRV**G*KR***QDIPRF**G*K***SSMIPVHY***RR***PLVYDRSDGDDEVFEIEFDVPSTDVS***KR***NGGGGMWFGPRL**G*RR***S***RR***SAELVEGTD***KK***EDSATLVDLLKNTPWALIALKAEYPDE***KR***RTVNFTPRL**G*R***ESGEEAQNLENRSTPFTPRL**G*R***SNEDRSVPFSPRL**G*R***QEDEEERGGSFEDYDRDDRSTVFSPRL**G*R***SAEVTRQQPSSKPQAKNQ*

>*Machilontus* sp. (Archaeognatha) CAPA/PK

***M***NLFVFVLGVVYVAHIADGLTAE***RR***GSLIPFPRV**G*R***SEFFMDS***KR***SSGLIPFPRV**G*R***SDMAMGEEALHEV***KR***QGLIPFPRV**G*KR***QSLIPFPRV**G*R***SPAYY***RR***PLAYYITDGDADDDMLELDVDVTNADI**G*KR***SGNGGTGGMWFGPRL**G*RR***S***RR***STEIVESSD***KK***EDSATLADLLRNTPWALIALKAEYPEE***KR***RTVNFTPRL**G*R***ESGEEEGQSLENRSTPFTPRL**G*R***SNENRSTQFSPRL**G*R***QQDDEEASEREASFEDYDRDDRSTVFSPRL**G*R***SVGTAPTRQQQQKQEQPSTRPQPQAKTH*

>*Tricholepidion gertschi* (Zygentoma) CAPA [GenBank:GASO01203566.1 + GASO01255059.1]

***M***NQALLSAVILVLVAGITHCNGASR***KR***SSLFPFPRV**G*R***ASWE***KR***DVSGLFPFPRV**G*R***GTWSADISGLHEV***KR***QGLIPFPRV**G*KR***QSLIPFPRV**G**…

…EDVEEV**G*KR***NGNGGMWFGPRL**G*RR***N***KR***SVESKIDLDQLYREYPWAVVALKEFGDGQQMVFPGPRDQDDLHNNADAEDDNEEEEEEVED***RR***F**G*R***STSQLHTRSRPSTPSQSKPQ*

>*Tricholepidion gertschi* (Zygentoma) PK [GenBank:GASO01003080.1]

***M***NDSILLQALALLVSTVFLARSEGLRIPVSVSSSPYFHLADLDGDAEEGIELELDDGDVFAGL**G*KR***EGGGMWFGPRL**G*RR***E***KR***SSDSNRDEAQESVLELLKNSPWALIALKEYQC**G*KR***HTFTPRL**G*R***ESEESLSVEHRSPPFAPRL**G*R***TSN***RR***ALPFSPRL**G*R***DQGDDDAQSESGHIFSPRL**G*R***DESRLNKP*

>*Thermobia domestica* (Zygentoma) CAPA [GenBank:GASN01031282.1]

***M***TVTLLYHTALLVLLVGFAHADGGI***RR***GLIPFPRV**G*R***SNPLQFDLRNEGPSFQSSASLFPFPRI**G*R***SNDQQTWDSGSTGLHEV***KR***QGLIPFPRV**G*KR***QSLIPFPRV**G*R***SSYSPIPSPIYFRDVLDDADDVNIDIDGWEDDVMDEVS***KR***SSGGGVWFGPRL**G*RR***N***KR***STEEKSVGSSFKLANLLKNPWVLLALKELSEESSTFTPRI**G*R***ELEISRSDEHTGPFN***RR***NDNRMDEDSATED***RR***FSSRI**G*R***STSNQTPPQQQPSRSHVDGTQLRQQS*

>*Thermobia domestica* (Zygentoma) PK1 [GenBank:GASN01003729.1]

***M***ASSVIVLVHLMAALVVTGIFSPAVAARFPLSYFSTPYLHVSEPDFDGEEVLDIEVEDPSELFNGLS***KR***EAGSSSVWFGPRL**G*KR***D***KR***SLESKAEKNEALNLAELLKDAPWALIALKAEYGS**G*KR***HSINFTPRL**G*R***ESEESQSVEERASPPFAPRL**G*R***NTDH***RR***AVPMVPRL**G*R***EEPSHRS**G*R***ANVFDPRL**G*R***SATKH*

>*Thermobia domestica* (Zygentoma) PK2 [GenBank:GASN01003730.1]

***M***ASSVIVLVHLMAALVVTGIFSPAVAARFPLSYFSTPYLHVSEPDFDGEEVLDIEVEDPSELFNGLS***KR***EAGSSSVWFGPRL**G*KR***D***KR***SLESKAEKNEALNLAELLKDAPWALIALKAEYGS**G*KR***HSINFTPRL**G*R***ESEESLEERSSPPFAPRL**G*R***SRD***RR***ESAPPPLIPRL**G*R***EEPGHRVDRANVFDPRL**G*R***SATKH*

>*Atelura formicaria* (Zygentoma) CAPA [GenBank:GAYJ01314506.1]

***M***TDTVLTCTILMVLLCSLNQCDGMHEV***KR***QGLIPFPRV**G*KR***QNLIPFPRV**G*R***ASLPAVPAAFYLADPEDPEDAAPALELDLEGLDLDSMDQVS***KR***TSNGGMWFGPRL**G*RR***N***KR***SIRTSKDISIAKLLRESPLAVVSMKGLSNDGYTIDENSIDGFSDESY**G*R***H**G*R***HGGGEEARVARAAPHTKLAALQTDKQG*

>*Atelura formicaria* (Zygentoma) PK [GenBank:GAYJ01310549.1]

…VLLLYTSLLASTVALSCGMRLPVPVLTSSYLQPSDLSLEADDLSAGDMDTTDLFSGL**G*RR***GATCKGAKGSCGGWFVPRVGHRD***KR***ALNSHGEVEESALVNLLKDTPWAFIALKEFPAA***KR***HTLNFTPRL**G*R***ESEESQSVEERSPPFAPRL**G*R***AIPMTPRL**G*R***DVSE***RR***HDRSSNVFQPRL**G*R***SVNKP*

>*Nicoletia phytophila* (Zygentoma) CAPA

***M***TDTFLWYTIMVMFLAGLNHCDGLHEV***KR***QGLIPFPRV**G*KR***QSLIPFPRV**G*R***SSYPPLPASLYLADLDDADDVDLDLDGFDPDPLDQVS***KR***TTNGGIWFGPRL**G*RR***K***KR***SLVETKDANLSKLLKESPWALVPMKGFSDERVYQEVMDSSNLDGERGGSFSHH**G*R***NLDNEVE**G*R***DTERFSRI**G*R***SASQTRPEHTRSRSLQFKH*

>*Nicoletia phytophila* (Zygentoma) PK

…FETVVSHVLFSSLLAAIIARSDGVRLPGLVTSPYRPGDLDLDDDVMGVEIDAADLYGGVV***KR***DPGCEKGNCRPQGLWFGPRL**G*RR***D***KR***SPGHADDREDSVLLSLLKDSPWAFIALKDYPG**G*KR***HTLNFTPRL**G*R***ESEESQSQSVEERSPPFNPRL**G*R***AIPMTPRL**G*R***QVSEREARANNVFSPRL**G*R***SKQ*

..............................................................................................

>*Xibalbanus tulumensis* (Remipedia) CAPA/PK

…IPFPRV**G*KR***QSLIPYPRI**G*R***SSYATSMVPSMFYIENDDTDGVGDYEAEAEDDTTDMSYDLQ***KR***DQGMWFGPRL**G*KR***R***KR***STSDE…

…KEGPWVLVPLDGLSYSHS***KR***ARYSSAFTPRL**G*R***SSESGESLSIENRSSPFAPRL**G*R***SSEKNSRENRSSPFAPRL**G*R***SQESAEAT…

>*Lithobius forficatus* (Chilopoda) CAPA/PK

***M***NTSCRRKCWCPHAAISIAFLAIATATTAQAYPLFAED***KR***QGLIPFPRV**G*R***SPFSVDPPTDVDDVYSDDPLSAFLYARALFNPRL**G*RRKR***SVQDGDNMDTLDLEEMGELL***RR***TPWTLLGYKENID***KK***NQMAPGD***RR***ANMAPRL**G*RKR***SDGSLLLDSDMETS***KR***GPNAPSAVSFSPRL**G*R***SSYVISDLERSPPQPNFNPRL**G*R***GTTNTNFSPRL**G*R***SANNEDSKLA*

>*Hanseniella* sp. (Symphyla) CAPA/PK1

**M**ELTNYNLGAFLLVVASCQAYQLFGEE***KR***QGLIPLPRV**G*R***SYYTLSHGHPSLRYDLEDVDDDVIEGAMPLRFTRGLFAPRL**G*R***K***KR***SVDSLDETSGSSVSDIDDLRDVLLNTPWAVVALKGLD***KK***STGTAFSPRL**G*R***AMVPRL**G*KKR***SDANLDED***KR***AVAAFSPRL**G*R***ANTAFSPRL**G*R***SGFERSAFSPRL**G*R***SFDSDSQISDDSRSS*

>*Hanseniella* sp. (Symphyla) CAPA/PK2

…PVSEELDLEMLTEMLQSSPWEVVAIKGMD***KK***ALSAFSPRL**G*R***AVAPRL**G*RKR***SDPDMDLD***KR***GAAFSPRL**G*R***SRNTNTRP…

>*Eudigraphis takakuwai nigricans* (Diplopoda) CAPA/PK

***M***NALAPLLTVCYLAALHTVTCFPYLGGEA***KR***QGLIPFPRV**G*R***TSSLLPLSAFYTDIDDLDEFYSGAVYLPEDVYFSRYARSYFAPRM**G*RRKR***SISSRENEGPEETQPSLEQDRNGPYVDLSMQDLARLLRKAPWTLLTFKGGIA***KK***TAFNPRL**G*R***AMAPRL**G*KKR***SGLSDDLDDNDDTSAMQE***KR***GTASFSPRL**G*R***SSPDGAHPTVWSDDYDGNLVSDEDLHMDRSTRGGASFSPRL**G*R***DVPIHTRASLSSGSHFSPRL**G*R***SDPQSNAASIAG*

..............................................................................................

*>Drosophila melanogaster* CAPA [[FBpp0084880](http://flybase.org/reports/FBpp0084880.html)]

***M***KSMLVHIVLVIFIIAEFSTAETDHDKN***RR***GANMGLYAFPRV***GR***SDPSLANSLRDGLEAGVLDGIYGDASQEDYNEADFQ***KK***ASGLVAFPRV***GR***GDAELRKWAHLLALQQVLD***KR***TGPSASSGLWFGPRL***GKR***SVDAKSFADISKGQKELN*

*>Drosophila melanogaster* PK (hugin) [[FBpp0082108](http://flybase.org/reports/FBpp0082108.html)]

***M***CGPSYCTLLLIAASCYILVCSHAKSLQGTSKLDLGNHISAGSARGSLSPASPALSEARQ***KR***AMGDYKELTDIIDELEENSLAQKASATMQVAAMPPQGQEFDLDTMPPLTYYLLLQKLRQLQSNGEPAYRVRTPRL**G*R***SIDSWRLLDAEGATGMAGGEEAIGGQFMQRMV***KK***SVPFKPRL**G*KR***AQVCGGD*

*>Daphnia pulex* CAPA (Periviscerokinin) [[Dappu1_312704](http://genome.jgi.doe.gov/cgi-bin/dispGeneModel?db=Dappu1&tid=312704), GenBank:[EFX87169.1](http://www.ncbi.nlm.nih.gov/protein/321476208?report=genbank&log$=prottop&blast_rank=1&RID=XHRPXYG3014)]

***M***RIAIIHSLVLVVIYLAYSDAAPPQILKSQSLIPFPRV**G*R***SRSSFIANAVGSARSGGAGNPMMMGSGGNANGKSPNNWMMNNADI***KR***HLIPFPRV**G*KR***QNLIPFPRV**G*R***AGYYQPGFFPSTDDEEGQASIQQQFALSSEESQASAPSSFLMSGSDILGALNNGRSNSDERTAVFIP***RR***WMTNSQESEE*

no PK known from *D. pulex*

Crustacean cardioactive peptide (CCAP)

>*Nipponentomon nippon* (Protura) CCAP

***M***QLNSALLVSLAVVLVVPAILGYSANRQADSQDADETGTQRI***KR***PFCNAFTGC**G*R***KRAFVGFPNNYEVANNELLQLARLLAAVRRIEQAQKQDQELIPSDENSSTSSGDSAYRRR***KR***SDLPKHQNNHQRNEQH*

>*Acerentomon* sp. (Protura) CCAP [GenBank:GAXE01028197.1]

***M***YINNALLVSFAIVLVIPAIMGFSSNRQTDPQEAEEQQLDS***KR***P***KR***PFCNAFTGC**G*R***KRSLIGPTSDLELNNEILQLARLLATIRRAERIQRTNPTEIQLQDEDSMENDNIFRRRRRSPGQPIRDHELDTSTNQNTQKEV***KR***ANEH*

>*Filientomon takanawanum* (Protura) CCAP

***M***YFNNAVLVSVAIVLVVPAIMGFTANRQPDPQESEEIES***KR***L***KR***PFCNAFTGC**G*R***KRSLIGPTNDLEFNNEILQLARLLATARRVERMQQRTAQIQSQDGIGNDNVYRRR***KR***SVLPMKIVRDNSAASDSTRKVSTDEFKSQESLQ*

>*Anurida maritima* (Collembola) CCAP [GenBank:GAUE01010373.1]

***M***MSMKSSKILVWLFGVFLVGITIVTAGTAPLK***KR***SLVVLARNGDPEDPSSDQVQKSRVPT***KR***TFCNAFTGC**G*R***KRSDSSMPPKDYEYEVPAETSDEVAVDDIARDILAEARLWEALQARNSIRVPFRNQPGFFPRQ***KR***AVMEKEKTK*

>*Tetrodontophora bielanensis* (Collembola) CCAP [GenBank:GAXI01150735.1]

***M***MI***M***RSQKTFVLCFFFVFIVGITLVTCGTTPIK***KR***SLVAMGGGESEQQTQQSSDQKPRLS***KR***TFCNAFTGC**G*KK***RSDTSVPNKDYEYDHGTMDNSDDSIDDVARDILAEARLWEALQARSSIRGPFRQQGFFPRQ***KR***SVNKEEKIIN*

>*Podura aquatica* (Collembola) CCAP

***M***DGRRFWVFLGGFLILFCSLLTIVSSGSTPIK***KR***SLVTVGGGESEEVEKSKV***KK***TFCNAFTGC**G*RK***RSGGGVVVREEGESTEDNDENNIDEIARDILAEARLWEALQATNSIRSPFRRQLGFFP***KR***S*

>*Folsomia candida* (Collembola) CCAP [GenBank:GASX01086101.1, GAMN01016815.1]

…AEYAGEPSSNNNPSPPRA***KR***TFCNAFTGC**G*R***KRNGGGGGFNRDYVDYSEGGDRGSVVDSDSDSGMEDVARDILAEARLWEALQARNSLRPRQQQRNKSSGGYRGEMRRPLEEQLPN*

>*Bourletiella hortensis* (Collembola) CCAP

***M***MISGQKSFTFAILILAVGVTVVMCGIAPIQ***KR***SILGDSDASDLKLRPN***KR***TFCNAFTGCA**G*KR***SDPMVNNKPDYEYDQQTGEGSEDSIDDVIRDILAEARLYEALQLRGPTRMSQQGRQQVGFFPRQ***KR***SVAEPRAIQGKDSIKMTSKVQRH*

>*Orchesella cincta* (Collembola) CCAP

***M***VNIQKNLTFIVLVFFILALAVVFCGKVPIK***KR***SLVVMEGDGSRDVKDQKSQPI***KR***TFCNAFTGCQ**G*KR***SDASAGAMKDYDYGHESVKEGTDENFVGDVARDILAEARLWEALQARNSMRYPVMRKQGFFGRE***KR***ATKSEEGGFNRIAPHNGIENRNNE*

>*Pogonognathellus sp.* (Collembola) CCAP [GenBank:GATD01101202.1]

***M***ATIVRNQKSIALWALIVVLMGITIAACGKAPIS***KR***SLVVMGGGGGDAAGQKPRPH***KR***TFCNAFTGC**G*R***KRSDDSVPVKEFEQEERDGTAENSYDTVEDLARDILAEARLWEALEARNSMRYPVMQSQTFSGRL***KR***SMGNDGIVLNEMKKTTEKSKQTP*

>*Jordanathrix leptothrix* (Collembola) CCAP

***M***ILPNPKNSPLFFLVIFYIGLTVVLGGKVPIK***KR***SLITVEPADMKLSLS***KR***TFCNAFGCG**G*KR***GDFNISPKQDYEYEQQGIIDVGDDSADDTARDILGEARLYEALEARNTLRMLQNRRQGFFPRQ***KR***AASPANENEPSDHQPVFNPSLLGAKKSQLPR*

>*Campodea augens* (Diplura) CCAP [GenBank:GAYN01004621.1]

***M***NNSQVLLFGHLLLLVIFTSFISATPVQ***KR***EIRSNPNYLQNKS***KR***PFCNAFAGC**G*KK***RTDSDVPPLNGVDSMQGMEVNEDYGNPEPSVENLLEDVIARTIETLVEVRQAHRRMIQEGAPLYLRRRRSTVQSVKEEEKDNGNE***KR***EEKSQDKKKSER*

>*Lepidocampa weberi* (Diplura) CCAP

***M***NNRQMITIICHLFVLVIFTSVATASPVQ***KR***EIRNSPTYVESKS***KR***PFCNAFAGC**G*KK***RTDNEVPPMTGVEDLPDEYPVGPYPAVENLLEDMIARDIGTLVEVRQAHRRLMQESGPMYQRKRRSVSSNKAEDNDELQEDKSQEQKAD***KR****

>*Occasjapyx japonicus* (Diplura) CCAP [GAXJ01007501.1]

***M***RAGSQLLFLGCAISVLLLVVEASPLA***KR***DIRLGDPDLMEAKS***KR***PFCNAFTGC**G*KK***RSDEGVSALSGAEAAAAMDLMGDGGGGGMDQVEVDPAVAKLARDILAEAKLWEAMQMRRRLEEQRMPLFLRRRRAAEAEKSGVKG…

>*Catajapyx aquilonaris* (Diplura) CCAP

***M***MRTGSNLLFVGCAISILLAASASPLV***KR***DLRSGDEMMESKS***KR***PFCNAFTGC**G*KK***RSDDGVPPLSGVADVEGLQGMQMELDPSVEKLARDILAEAKLWEAMQARRRFDERLPLFLRKRRSLAQGTAWASGSHSAKGQATQR*

>*Pedetontus okajimae* (Archaeognatha) CCAP

***M***KTNCTIIFCAAFVFLLAILPLTRGASVLA***KR***DISGLDDRLLEPK**G*KR***PFCNAFTGC**G*KK***RSDASLPLMDDASSDANVDVDIERLNRQLIAEAWLSQLLRRQAEGERPYQFGARRTRSASNQKLAVEDHQPRR*

>*Machilis hrabei* (Archaeognatha) CCAP [GenBank:GAUM01177911.1]

***M***NYGIFCAAFVFLLAILPLTRTASIVA***KR***DISGLDDRRLEPK**G*KR***PFCNAFTGC**G*KK***RSDASLPRIDYASGEDNVDFDIERLNQQKLADAMFSQLLRRQAELKSPYQFGLRRRRSATNTKSAAQDQHPRR*

>*Meinertellus cundinamarcensis* (Archaeognatha) CCAP [GenBank:GAUG01030384.1]

***M***KGYQSVVGVAIVFLVVVCSLTSAASLNI***KR***DISSSDDALMQPK**G*KR***PFCNAFTGC**G*KK***RSDSSIQVLDSPNEDSIDVNNINRQLAEAWLAELLRRRAEADGPYRLGYRRRRSATVPESAEKDQHPRR*

>*Machilontus* sp. (Archaeognatha) CCAP

***M***KTYHTVVGVAVVVLLVICSFASAASVTI***KR***DVSGSDDDMPLQPK**G*KR***PFCNAFTGC**G*KK***RSDSSINLLEGSNDDNIDIDRLSRQLLTEAWLSELLRRRAEVEGPYRHGLRR***KR***SPEAPALAEKNQHPRR*

>*Tricholepidion gertschi* (Zygentoma) CCAP [GenBank:GASO01020341.1]

***M***QRCHVIFACSAIFLLLIIPLTSSAEILEKREIDDSDIQLMDPKR***KR***PFCNAFTGC**G*KK***RSDDSLGTLVEMNSEPAVEELSRQIMAEAKLWEALQEARVELLRRRQGHQQADRLFPLVGLRK***KR***SPGNKGPAKTVTTETRSQAEPWSR*

>*Thermobia domestica* (Zygentoma) CCAP [GenBank:GASN01010397.1]

***M***NLCQAALTCSLIVLLVIIEFTSSAEIIR***KR***DVGDVDYPVIDPKK***KR***PFCNAFTGC**G*KK***RSDESLGTLTEYNSEPAVRDLSRQILAEAKLWEAIQEARLELLRRRQEEAEQGVPIGLRK***KR***SFGQVKSLDSTNKQTKRKEAPSNSGR*

>*Atelura formicaria* (Zygentoma) CCAP [GenBank:GAYJ01316277.1]

…GCCVAIMLVLVSLASSAELIR***KR***SVDDVDYAIMDPKR***KR***PFCNAFTGC**G*KK***RSDDSINSLLDLNSEPAVADLSRQILAEAKLWEAIQEARMELSRRRAHQREDGERFALRK***KR***SLLERAFQRTGHSGLREQDELAER*

>*Nicoletia phytophila* (Zygentoma) CCAP

***M***QMYYSVFGCSVILLLVILPLASSAELVR***KR***SVGDVDYPLMDPKR***KR***PFCNAFTGC**G*KK***RADESIGTLVELNSEPAVAELSRQILAEAKLWEAIQEARMELSRRRQQEKEDAEELGIRR***KR***SVLERIFQRPGYNDVTSQGQMARR*

..............................................................................................

>*Xibalbanus tulumensis* (Remipedia) CCAP

***M***QTYHVFLACVVAALFAIIPFASTASIK***KR***EISGRDVPLQTKS***KR***PFCNAFTGC**G*KK***RSFDAFANNADMDTADTDLDKLGRQILSEAR…

>*Anaspides tasmaniae* (Malacostraca) CCAP

***M***QVVAGQRGGRAGVVTITTLAFLMLAARISAGPLV***KR***DLSDMLEVKD***KR***PFCNAFTGC**G*R***KRSSSPEMEVEAASGQELDTLARHVLTEAKLWEQLQNKMDLMRSLAARMEDHPLYRR***KR***SAIHAPAHHVDDDHTNHTSAVAPIQAEENKA*

>*Lithobius forficatus* (Chilopoda) CCAP

***M***QYPISAALCVVVLGVVCSLVRSSELD***KR***EISGSEDTLIPK***KR***PFCNAFTGC**G*R***KRSDGSLVDAENDLLLEKLSQQILALVHLLEALHARIEATKQQPIGEDDARIFNYLAAR***KR***RSSANRDVAHLSEK*

>*Hanseniella* sp. (Symphyla) CCAP1

…VLCAILVVRAAAEPEQTEDFGSDEPPMPR***KR***PFCNAFTGC**G*R***KRSGAGSSLDRDDDAMQQFYRRLAEPPRGWSDRQALFTPTYFPRDFSGSYYQGFAGRRKRSLTSAE***KR***HQLPAAHEDLVQ*

>*Hanseniella* sp. (Symphyla) CCAP2

***M***EILSSFGFQYLLLIFLSDIFLGNPAGGFGVGVDSSTDQHPVPGMITFCKACTGCVLKPSSDPSVRISPQGNEAPRRPTRTQVFTDQLPPP***R***SGSFCNAFTGC**G*R****KR*RSLRSSDPPIQTVDILN*

>*Eudigraphis takakuwai nigricans* (Diplopoda) CCAP

***M***QTLSSSTAVVSCWALLALVLLATSVSSSIADPRESLVPENDLPPMS***KR***PFCNAFTGC**G*KK***RSDLPSEIAAVEESNSEINVLWEQLIRRILEQMRLRDAITRLHKTEEGRSGLYNYVEDQRR***KR***SPTYFPVMDNPFYHSGR*

>*Eudigraphis takakuwai nigricans* (Diplopoda) CCAP-like

…VPNINEHT**G*KR***SNSDFSHQLSSSNDIDQSQAYEAFEIK***KR***PFCNYGGCRN**G*R***KRTYDGEESSASTNSYDLNELINEISNWFRAIPSPGRGTTPSGEIYQFENYEPVV…

..............................................................................................

>*Drosophila melanogaster* CCAP [[FBpp0083726](http://flybase.org/reports/FBpp0083726.html), [FBpp0307015](http://flybase.org/reports/FBpp0307015.html)]

***M***RTSMRISLRLLALLACAICSQASLERENNEGTNMANHKLSGVIQWKYE***KR***PFCNAFTGC**G*RKR***TYPSYPPFSLF***KR***NEVEEKPYNNEYLSEGLSDLIDINAEPAVENVQKQIMSQAKIFEAIKEASKEIFRQKNKQKMLQNEKEMQQLEERESK*

>*Daphnia pulex* CCAP [[Dappu1_300598](http://genome.jgi.doe.gov/cgi-bin/dispGeneModel?db=Dappu1&tid=300598), GenBank:[EFX70015.1](http://www.ncbi.nlm.nih.gov/protein/321458956?report=genbank&log$=prottop&blast_rank=1&RID=XKTMW0XB01R)]

***M***TRPLFYSLLMLAWMIISLYISASSSQPLKNNQNDSDSAEEIEQWSFKE***KR***PFCNAFAGC**G*RKR***SMIKDTKHPPYEKAASNHPRLPNADTKLLDKLFAKIQHQRANFVQLDDPEYY*

CCHamide (CCHa)

>*Nipponentomon nippon* (Protura) CCHa1

***M***LSSCRSVALMAFAASIYATFIVVTVESGCSNFGHSCFGAH**G*KR***GGSNDVGGGGGGISGGITESQQGRGEAEEVA***RR***RQLAINDDIIEKIGPIIALADEQDVVPLAVLEEKLLLPTNSLDSLFMQTNLSPQFELWRKYM***KR***TRNQRWRQDRESKE*

>Acerentomon CCHa1 [GenBank:GAXE01136826.1]

***M***LPPRISLALLSITVLIYAAVSVVPARSGCSNFGHSCFGAH**G*KR***GGSP***RR***TNQETDNDVTDYMEPIISLDEREAAVPLSVLE***RR***LLPSATIDKLLLERFTPSFKEWDRFLNRQHNKGARNPESEEGETKF*

>*Acerentomon* sp. (Protura) CCHa2 [GenBank:GAXE01028488.1]

***M***CNIGHLLTVVVVLMCILFTFVQKSESSCFKYGHSCWGGH**G*KR***SYPPSRTHSAAYLRSLERQQNGVR***KR***APTKTGFQIFRIETQPEIHQLLPSSVGE***RR***RLIHPEVNQRSFQGHPIRGDPRADDAVVQQESRVA***KR***RQDYAGDQDNRDVIDIEYVIADDANSDADTNYQKDEEYDSEEEQPIS***RR***RLNMGEYRK*

>*Filientomon takanawanum* (Protura) CCHa1

***M***FTLRRSVALLSFSVIVIAALNIVPAQSGCSNFGHSCFGAH**G*KR***GGVTE***RR***ANQEADSDVTDDIQPILTLDERDLVPLSLLEQRVVPSVRIDKLVFERMNPTFKEWGRYL***KR***RQSQGQDDPEPKD*

>*Filientomon takanawanum* (Protura) CCHa2

***M***CNIGHLFIVMAIMICVVCTLIHQSESSCFKYGHSCWGGH**G*KR***SFPPSRTHSAAYLRSLDRQQNGVR***KR***NPTKMGFQIFPNDQQLESQRLPSVAERRKLFLLNPVVNSIGTSDDDRIIFQGHPMRHYFHGDVRADETDPRRIA***KR***RPLNLRDQENGEMIEPDYSEEGNNEIETNYQTKEEYPADDEARREEDGDYRK*

>*Anurida maritima* (Collembola) CCHa1 [GenBank:GAUE01049781.1]

***M***KQQAEKGCGATLWTLLTLGLVILAITPASEGGCSRFGHSCFGAH**G*KR***ADDVDLRPEPIGTVPLIRTDSGRVIDYDTRISPLVRDWILGMRREASEDGRSKEFLNS***RRK****

>*Anurida maritima* (Collembola) CCHa2 [GenBank:GAUE01052705.1]

***M***WIHIHFIFALGLINILLIPISHGSCWRYGHSCWGGH**G*KR***AGDPPPGDHSNPPIIQSRIPATPLDPDPLLSPASREWAEEPSREGVETM***RR***DGNEIIFNQNLIEPEVGLSSEEVRDLDLDRGEAEIDFEPIPGRQRERSHPGVIFFRIFR*

>*Tetrodontophora bielanensis* (Collembola) CCHa1 [GenBank:GAXI01004805.1]

***M***SIAIVTMTILLICGIRQVQGGCSRFGHSCFGAH**G*KR***ADLPNSYEMSQSNLDVSPNGYILSQQPGYISLPNTNNGRISPYLIDWLLSARKGESKQQELSKQEDIQPLEMR***KK****

>*Tetrodontophora bielanensis* (Collembola) CCHa2 [GenBank:GAXI01036939.1]

***M***CSLKTCSHFVTFVVFFVSLNAVLA***KR***GCAQFGHSCYGGH**G*KR***SQGFDSFSPD…

>*Podura aquatica* (Collembola) CCHa1

…FPYGHLSISLILTFTLLIFISIQNTEGGGCSRFGHSCFGAH**G*KR***ADISAENNSDDVAQFYPLPQLSPNNFQLSNSRISPLLRDWIRNSHRDDALRARLMSRNDDVGVEGVENNFRM*

>*Folsomia candida* (Collembola) CCHa1 [GenBank:GASX01087171.1]

***M***ICLVALVLLWGEVREAQAGCQRFGHSCFGAH**G*KR***GGDVGATGGQQNGAELSPGESVYPAGPGAEYYPAGGFAPRVGMSPYLMDWLLSAHRGQPLSANYARQLQDDLNMADQ***RRK****

>*Bourletiella hortensis* (Collembola) CCHa1

***M***MKQEARVRITLAGLTLLLISGIHHVQGGCSRFGHSCFGAH**G*KR***TDPSGGVLDLNGTPDIAGYNNVLRDLYGSYQQQQQQASRYPPGRMSPYLFEWALAAQRAADLRQPAEHEAKEDGAIVDM***RRK****

>*Bourletiella hortensis* (Collembola) CCHa2

***M***MINSKSMMSKLSVTLIFLILAFICSAQASCWRYGHSCWGGH**G*KR***SGGRAAMATDDSELLPSGDGEELS***KR***QQRIPQELQPLQSMSSATFQGDGMGGRDNIELMSRSPKELYRSKFQSIFPYSSTNNDDLRDEIMEDKQQVDEEDDTGAPADYIQSQEQTEGRENDLTMDAARNKEHTPEADSSEIFIGIPRISSTNMKPLAIATNEDSI***KR***AVGDLSARKFPLENFNLYKLVRIV***RRRK****

>*Orchesella cincta* (Collembola) CCHa1

…GCSRFGHSCFGAH**G*KR***ADIAAGYNGNVDPMMMVAPGGSNQVGAGGAVPSDAIPDGYYPNLLPMSSASNNRMSPYLLEWILSAHRGGDSSRQVPNPSADDMQTAY***RRK****

>*Pogonognathellus sp.* (Collembola) CCHa1 [GenBank:GATD01014443.1]

***M***TILGLGLILLWGELEQVEAGCSRFGHSCFGAH**G*KR***SDGLPTAALNDILIPSGEMPVFAGGYAAQPVRSEYYPNYVPVASNRISPYLIDLLMSAHRNELRQP…

>*Pogonognathellus sp.* (Collembola) CCHa2 [GenBank:GATD01040461.1]

…YYYGHSCWGGH**G*KR***SGNEGLLSANKVFGFHSSPLLQMLMRNKGNSNSAESFFAGRSSENLLDPNLPYNNKNSNGNAKPN…

>*Sminthurus viridis* (Collembola) CCHa1 [GenBank:GATZ01101512.1]

…GLTLLLILGIHQVQGGCSRFGHSCFGAH**G*KR***AESVVPGSSVDLNVPTAGELAYGQPLRDLFNSFQQQQQQPPSRYMNRMSPYLFEWALAAQRAGDMRQPISKEDSGLEM***RRK****

>*Campodea augens* (Diplura) CCHa1 [GenBank:GAYN01132522.1]

…H**G*KR***GENPSDVGAANPRVQNTQQLWEALNMVEDPVTREAAAEAAIDARFQKMSPLLRNWITLLSQRSSNDRV*

>*Lepidocampa weberi* (Diplura) CCHa1

***M***ANSAMPIALLVIALALFIDVASGGCSAFGHSCFGAH**G*KR***AESVDTGAPRIQNTQPLWEALNLLEDSGTREAAAEAAVDARVQKMAPYLRNWITLLASQRSSNDRV*

>*Lepidocampa weberi* (Diplura) CCHa2

***M***SLIAIKWNRILVIVAQGIFLTCLLHSAYGSCNSFGHSCWGAH**G*KR***NGAVAPLSAYEENNNANDYNPPEDTLNREYNSPTRSDINMMENRMVFPRYQALLRGWPSKHFPTQSRGHNTYQDQEGVRETLRSLLDTLMASNNYLEDDPPMNLPLQEQKET*

>*Occasjapyx japonicus* (Diplura) CCHa1 [GenBank:GAXJ01109520.1]

***M***ASNSALSLMLFALSFGLLLHLASGGCSAFGHSCFGAH**G*KR***GEVAMGPRPRPSDGDLWEALEEAAMSDADQQDEAAPGMMPSFALKPQFQKLSPFLREWVLSLKQRNGNNRA*

>*Catajapyx aquilonaris* (Diplura) CCHa1

***M***ASSSFPLVLFLLSFGLTLHLASGGCSAFGHSCFGAH**G*KR***GESAAAAMGPPRPRPPAEGLWEALEEAAMTDSVDQPDADNPSGPAGIPSYPFKPPTQKLSPFVREWLLSLKHNRA*

>*Catajapyx aquilonaris* (Diplura) CCHa2

***M***SSFLMIFYGCLFCSMLHNTAASCSRYGHSCWGAH**G*KR***SGGSLDAPAPSRLEKLFD… (frameshift?)

>*Pedetontus okajimae* (Archaeognatha) CCHa1

***M***VSSCVIVTVLATCLVCTVQLVSGGCSSFGHSCFGAH**G*KR***SQASIQGPLEYGDVSEVVPPGDMDSPFEGNSVPSVNERDILRLSPLIRQWIKVLRQRERESEDAK*

>*Pedetontus okajimae* (Archaeognatha) CCHa2

***M***VGSGVSKVLLTHLIVLSCVIIVSSGSCLSYGHSCWGGH**G**x***R***SDSSLSPQDGIEVA…

>*Machilis hrabei* (Archaeognatha) CCHa1 [GenBank:GAUM01010860.1]

***M***VSSCVLLTVLATCLVCTVQLVSGGCSSFGHSCFGAH**G*KR***SQANLQGPLEYGDLTEVVPPGDMEGTFDGNSGPSINERDILRLSPLIRQWVKVLRQRGREAEDAE*

>*Meinertellus cundinamarcensis* (Archaeognatha) CCH1 [GenBank:GAUG01221839.1]

…GDVLRNFDGNSASALDERTILRLSPLIRQWMNALRQRERESNDEK*

>*Machilontus* sp. (Archaeognatha) CCHa1

***M***VSNCVLLTIAATLLVCTVQLCSGGCSKYGHACYGAH**G*KR***SQGASAGSLEYGDAAEIVPPGEMMRNYDGNSLTALDEQNFLRLSPLIRQWVSVLRQRERDSDDDN*

>*Tricholepidion gertschi* (Zygentoma) CCHa1 [GenBank:GASO01251700.1]

***M***AGWSTVVFLALSTTFLLVSTIQPATA***KR***GCAAFGHSCFGGH**G*KR***SDSGDTSPDSQEIFFRGDATGVYSDGLTRNYQDERYPTAGQSLERISPFLRQWVQSL***RR***TNGEDVK*

>*Tricholepidion gertschi* (Zygentoma) CCHa2 [GenBank:GASO01154233.1 + GASO01109769.1]

***M***IGAGHAGVRSIILLSCFVCYVAGSCLSYGHSCWGAH**G*KR***SGSATGSGLQEENLPVAMSTPEDNRWVLSKLV…

…KYKVWQRLKTS***KR***NEKLARSPMRIEGEPPAVLISDSEFPLPQDQESADEDDDILVVAEDPTSGGP***KR***LRLYKIMEHMARKL…

>*Thermobia domestica* (Zygentoma) CCHa1 [GenBank:GASN01403707.1]

***M***ASWSTSLLLAAVSLLILINTIEPTFA***KR***GCSSFGHSCFGAH**G*KR***TDMRAISPEAEETFLRNMNEALSASNVENYEQQRYPVLSQESMASISPFLRQWVQSL***RR***TPEDIDVK*

>*Thermobia domestica* (Zygentoma) CCHa2 [GenBank:GASN01402928.1]

***M***SNIDCMDMKITIAVLCCCLVTGAWGSCLSYGHSCWGAH**G*KR***AGPPASANLQNEDLPAVAVASPEDTRWFLSKLIEQSLPDTSDNSPSDETYKLWQRLKAF***KR***SEISDKVDRLPSLARLSSGEPLGIVLPDAETDALAQESSQDGGDILLLAEETPSGGTQKLRLYKIMEHIARKID*

>*Atelura formicaria* (Zygentoma) CCHa1 [GenBank:GAYJ01006533.1]

***M***ARWSAAILVAATGLVLLLLSVEPAAA***KR***GCSSFGHSCFGGH**G*KR***SDPGDTTREDEESFIGPATDELSGGLPRNYQDERLYSLLNQPLERFSPLVRQWVNRVQSL***RR***TAGEGDVK*

>*Atelura formicaria* (Zygentoma) CCHa2 [GenBank:GAYJ01025318.1]

***M***RTTPGHTEARIAVILLCCLVSYTSGSCLSYGHSCWGAH**G*KR***SGKSIGGSHDGRTAVSVVSPEDTRWFLSRLMEQAVPDSSEASGSVENYKNWQRLKFMRPGDSMERDRSFHLESETEEKEDEE*

>*Nicoletia phytophila* (Zygentoma) CCHa1

***M***ASWSTTVIIAVTALLIVVAAAQPALA***KR***GCSSFGHSCFGGH**G*KR***SGVGDISREDEETFVRGATDELSGGLPRNYQDERFSSLLNQPIDRISPLLRQWVQSF***RR***TSGEVDVK*

>*Nicoletia phytophila* (Zygentoma) CCHa2

***M***NITGQEVKVVVVLLCCLVSYASGSCLSYGHSCWGAH**G*KR***SGLPYNIQDGRSSVALASPEDTRWFLSKLMEQAVQDTGDTPGSAENYKIIHRLKSIKPIENLEKV***RR***SQPVSMDSESSEVLMPSSDLSMAPAPDAADPDDDILVLTEDALRGQPRKLRLYKIMERT**G*KK***LV*

..............................................................................................

>*Xibalbanus tulumensis* (Remipedia) CCHa1

…GVVSGGCSNFGHSCFGAH**G*KR***ADGADMIGDARAVNDPGNMNMDYPIMSQYDDSSQKLSPFLKLWVRLYRQRMGNDFLGQ*

>*Anaspides tasmaniae* (Malacostraca) CCHa1

***M***TGGGIMAPLVMALLMLAGQTSGSCSQFGHSCFGAH**G*KR***NDDSAFRSPPDAVFPAAIPRPDTLTDVMYQASNPKPDTINDVMYPVINQRPDLEESSEDIGRVLDRNTITNPEIVESVRNWLSVLS***RR***LR***KR***TMFQMPVPMAYYH*

>*Lithobius forficatus* (Chilopoda) CCHa1

***M***ASGTRLIFIAIVWSTACLTATAARGCLSYGHSCLGAH**G*KR***SSSSSLATVGNAAYFQRLPARLYRQMDVDNELDSSSGSSAAAAG…

>*Lithobius forficatus* (Chilopoda) CCHa2

…***K***GCSNFGHSCFGAH**G*KR***TDPSEGGVAPYPLPYADTGTEGGPDTYDARVRQELSGQQ…

>*Hanseniella* sp. (Symphyla) CCHa1

***M***NAANTCFVYFILTIVSASALKGCTSFGHSCFGAH**G*KR***MDPSEANLAPPPGVRLTSEEFGPIIMSPNSGPRAGADMPARLKGFLRLWAEVANRNIESEDGYN*

>*Hanseniella* sp. (Symphyla) CCHa2

…ASTSFLLLCVMCCLTFHASGQCGRFGHSCWGGH**G*KR***SSSQTISEDTYSNNINNDDLLIRLLMLSVTSQENIPVDYQVDSSQDYDINP…

>*Eudigraphis takakuwai nigricans* (Diplopoda) CCHa1

***M***VVSFGVSLVAVCVFVTSFQPRQVSGLRGCSSFGHSCFGAH**G*KR***SELGSEGDGVFPDSRTPDLDAILLNSATGTSAAADELESAPPRLLPDDDGAELRSQQPNHLLDLLKGSRWAAGSGRPDGYTTVMRRIYRNLAQINRPVLHAGAP*

>*Eudigraphis takakuwai nigricans* (Diplopoda) CCHa2

…LLIVACVLWLLVLEPTPVEGQCRRWGHSCYGGH**G*KR***SDILPLIARPTATTSDRWLYQALPQLAALRQPPFYG…

..............................................................................................

>*Drosophila melanogaster* CCHa1 [[FBpp0112144](http://flybase.org/reports/FBpp0112144.html)]

***M***WYSKCSWTLVVLVALFALVTGSCLEYGHSCWGAH**G*KR***SGGKAVIDAKQHPLPNSYGLDSVVEQLYNNNNNNQNNQDDDNNDDDSNRNTNANSANNIPLAAPAIISRRESEDRRIGGLKWAQLMRQHRYQLRQLQDQQQQGRGRGGQGQYDAAAESWRKLQQALQAQIDADNENYSGYELTK*

>*Drosophila melanogaster* CCHa2 [[FBpp0082230](http://flybase.org/reports/FBpp0082230.html)]

***M***KSTISLLLVVICTVVLAAQQSQA***KK***GCQAYGHVCYGGH***GKR***SLSPGSGSGTGVGGGMGEAASGGQEPDYVRPNGLLPMMAPNEQVPLEGDFNDYPARQVLYKIMKSWFNRPRRPASRLGELDYPLANSAELNGVN*

>*Daphnia pulex* CCHamide [[Dappu1_304056](http://genome.jgi.doe.gov/cgi-bin/dispGeneModel?db=Dappu1&tid=304056), GenBank:[EFX80320.1](http://www.ncbi.nlm.nih.gov/protein/321469340?report=genbank&log$=prottop&blast_rank=1&RID=XKU4YXT301R)]

*M*HIFFYVIHVTAMLAIVSGNCNKYGNACFGAHG*KR*SDF*KR*TSAVDLSDQIWPVAANWNPTRPDEPIQERRQMKPLPALQLESVLVYNDIPRSAEHSRYLNQEDYNN*

CNMa

>*Campodea augens* (Diplura) CNMa [GenBank:GAYN01012995.1]

…YIVTSLCVCPIRSENTPDEYASSPEEVAFESEEKQRWPTTDDFSQENKDKYRIPPQTHPLAPHIIQLENLLQRAFSNPIYRFPSEVNDEIYNYVRQGKEPPTHPLYYQSATVRNSPESQEFIVPPPIVLGKR***KK***AQYMDYVCHFKVCNF**G*RKR***KGVSGPVSYNF*

>*Lepidocampa weberi* (Diplura) CNMa

***M***FPSASLSFVTCVFLWCVLSCSSVHCDDFEYSPSALSQDTLSPPHQEAAKQWWDEGETPKQASPLFRPELLPPQLLSSYRSLTQGGFPYPISRFPAPSSDAVANNIHQRHSSLHLPAMLRPRVELPADSKFFNALFEVEKR***KR***PYMDYMCHFKVCNI**G*RKR***EDGHYRRF*

>*Occasjapyx japonicus* (Diplura) CNMa [GenBank:GAXJ01073669.1]

…LQAAPASFSFYKNSLLGADTDAIDDKE***KR***GSYMSLCHFKICNM**G*RKR***NLRSMNPWIRQ*

>*Pedetontus okajimae* (Archaeognatha) CNMa

***M***IHNSWRFVFVVVSLTVSGFLSISSVSAQEVPQLTEDQLPSSVRDNTLYRLLTALQPPEPPELNDLTMQHLLPESDVHQEG***KR***GSYMALCHFKICNM**G*RKR***NMRGYPWLRL*

>*Machilis hrabei* (Archaeognatha) CNMa [GenBank:GAUM01176396.1]

***M***IPSSWRRLVLVVVGITLSGFVGISTVSAQEATQLTEDQIPSFVRESGKLYRLLAALQPPEPPEHDFTMQHLLPESEVLQEG***KR***GSYMALCHFKICNM**G*RKR***NMRGYPWLRF*

>*Meinertellus cundinamarcensis* (Archaeognatha) CNMa [GenBank:GAUG01237542.1]

***M***LPPHWSRLFVVAVLMTSGVLGISAQISDDRTRETNDQMPSIIRESALYRLLSALQPLEPPEMQNYSADRINQESDILQEG***KR***GSYMALCHFKICNM**G*RKR***NIRGYPWLRL*

>*Machilontus* sp. (Archaeognatha) CNMa

***M***LSPIWSRLLVVAVLMATGVMGVSVLATNERPTLINDQIPPINKDSALFRLLTVLQQREPPELKDVSMERSLQDLDLLDEN***KR***GSYMALCHFKICNM**G*RKR***NLRGGNTWMRF*

>*Tricholepidion gertschi* (Zygentoma) CNMa [GenBank:GASO01257211.1]

***M***QVLCLSVSLFWTLVAATPVASARTVYTGSQGVPQAGIPHYPIDQDSVDLYGNPDPKKENQLRQMANLYDMLQDLELQQENQPPMLPRGPVFSLDEYRDPYDDGSFKG***KR***GSYMSLCHFKICNM**G*RKR***NRRWIRL*

>*Thermobia domestica* (Zygentoma) CNMa [GenBank:GASN01391823.1]

***M***HTFWIAVSAVVLASMIVGAQDLKEGIRRQDYPQIPLDQGQIDYLPNLDPDKENKLREMSSLYQMLQDAAFQRDMEYRLPQEQIFTPDDFRDRTDDGTTKG***KR***GSYMSLCHFKICNM**G*RKR***NLRPLFRP*

>*Atelura formicaria* (Zygentoma) CNMa [GenBank:GAYJ01317325.1]

***M***QANIMCAAVLWLVAVLAVGSEARASSIEPDQFMPPVPDSLQYPEQEEASAFQNVNKVKENKLRQMVSLYQLLQNMAYVQPEDSRLPAAPLFSSEDYRDPNDDGTNRG***KR***GSYMSLCHFKICNM**G*RKR***NLRWNPWLKL*

>*Nicoletia phytophila* (Zygentoma) CNMa

***M***QVRSILVSVMWLVAGTEVGLCLGTSQMLPSAPDVSHYPDSQTPLEVFTNDDIEKENKLREMVSLYQMLQEMAYQPADSSRLPQGQVFSPEEYRDPLEEGSTKG***KR***GSYMSLCHFKICNM**G*RKR***NLRWNPWLRM*

..............................................................................................

>*Lithobius forficatus* (Chilopoda) CNMa

…***R***FSLMCHFKICNL**G*RRR***RIQTSAADDLSDDQRFEASDVERL*

>*Hanseniella* sp. (Symphyla) CNMa

…GAMVNKDIIR***KK***ANGRYVMCHFKICNL**G*RRRR****

>*Eudigraphis takakuwai nigricans* (Diplopoda) CNMa

…SLMLFRAMRVLMKVLPEERLPFPEGEYSESAELDGEPNESKRSSYR***KK***ANSKVMCHFKICNL**G*RKR***RR*

..............................................................................................

>*Drosophila melanogaster* CNMamide-PB [[FBpp0289937](http://flybase.org/reports/FBpp0289937.html)]

***M***SALSAPTTCGCSPVHWAIVIVLLSVAIGPGDAMARPARNTQLLFSELLGGGNDDNNYYGDQLKYQQQQQQQQEQKQQRVPAFARKWPSLRDLLLTVDYDDFGVTQESEEQVAPSSRLLARLHRLGDNGGGEELRYNVVNELTNMPSKKVMPGHPLKDHNT**KK**NVQF***RK***QYMSPCHFKICNM**G*RKR***NAGFNSY*

>*Drosophila melanogaster* CNMamide-PD [[FBpp0304918](http://flybase.org/reports/FBpp0304918.html)]

***M***SALSAPTTCGCSPVHWAIVIVLLSVAIGPGDAMARPARNTQLLFSELLGGGNDDNNYYGDQLKYQQQQQQQQEQKQQRVPAFARKWPSLRDLLLTVDYDDFGVTQESEEQVAPSSRLLARLHRLGDNGGGEELRYNVVNELTNMPSKKVMPGHPLKDHNT***KK***NVQYMSPCHFKICNM**G*RKR***NAGFNSY*

>*Daphnia pulex* CNMamide [[Dappu1_443191](http://genome.jgi-psf.org/cgi-bin/dispGeneModel?db=Dappu1&id=443191)]

***M***LAILIAGLLLSAVPNIRTQPSLNEILDTPPPTGNPLSWARQHSNTEEMLPFERLNPRFWLGPEYRQALRQIAQMREEDPQIFNGLNVYRAADGFIAQGSNAMDRTDIGVRLSPARERLQVANPDAQSNMG***KR***DSYLSMCHFKLCNL**G*RK***RRISQGGHTDLVTSDLNAEK*

Corazonin

>*Nipponentomon nippon* (Protura) corazonin1

***M***RGQSSSLLLSLVILSALIAPFTCQTFQYSRGWVN**G*KK***RSDPELIRASLLQAQAEANSMFPPRYKLSPSDPKVIEQLVADHKLNPVLSSGYGYLFPATQVRINRNPVPILLRALLEEYNKPELIEAKEPGQSSSSSELNQADD***KK****

>*Nipponentomon nippon* (Protura) corazonin2

…VN**G*KK***RSDPEVTKGNHQD***KR***PYMYRLPEMIEPNYLENVVAQGKLNTASNGHQYLPKGPADVQLLIRALWQELNRMHYYSNPPIEKGTEGQSSPVEADQDEDGQ*

>*Acerentomon* sp. (Protura) corazonin1 [GenBank:GAXE01006697.1]

***M***TSCFRALPSCRVSCPPLLTVLVLAALVSPLLSQTFQYSRGWVN**G*KK***RADPEMMAAKPNLETVQE***KR***PYLYRIPEMIEPKYLDNVVTRSRPSGVNSGVQYLPMKAPSDIQLLLHGLWQEMNRFRSIQGSPEKVSQGQSSTSEVNDPEDSLDKK*

>*Acerentomon* sp. (Protura) corazonin2 [GenBank:GAXE01006696.1]

***M***TSCFRALPSCRVSCPPLLTVLVLAALVSPLLSQTFQYSRGWVN**G*KR***ADPDMRAAKPNLETVQE***KR***PYLYRIPEMIEPKYLDNVVTRSRSSAVNSGVQYFPMKAPSDIQLLLHGLWQEMNRFRSIQGSPEKVSQGQSSTSEVNDPEDSLDKK*

>*Filientomon takanawanum* (Protura) corazonin

***M***MLNRIPLTSVKVSISSLLLVLVLAALISPLFCQTFQYSRGWVN**G*KK***RSDPEVKPIQQQE***KR***PYVYRLPELVDPNFLEKVVAQGRLNTGSSGIHQYLPKGPSDVQLLIRALWQELNRMHHFSNQPIEKGTEGQSSPMEPDQEEDGQ*

>*Anurida maritima* (Collembola) corazonin [GenBank:GAUE01051001.1]

***M***RIGQVSLLKAVIAIFVLISVGCQGQTFQYSRGWTN**G*KR***GAYSVGNRGSVNPLVSGQSPTYDSSTVLSGSSSEESSIPIPPVSSSGRGGLGRIGRQPGRFQSLDYALYL**G*KK***LGNGPSNNAAVGSGN***KKR***PLWIIDADQDGNPNVEIQ*

>*Podura aquatica* (Collembola) corazonin

***M***RSSLRSITGESSILPNFRRGIVSEFAFRLSVVILVVIVIGCESQTFQYSRGWTN**G*KR***GQRIFLQQAEAQKIPNDVETLNNDGMNDETLIPSSAMLHQINLKGQNFL**G*KK***RMMLAMRGGNVHQFEKQPIWILRTDSTSSYDSINPGGLNTSEF*

>*Folsomia candida* (Collembola) corazonin [GenBank:GASX01005098.1]

MTNFGNQASLFEDSKPEFHANGKKCRKPKEAWARWGLLLLVGATLLATAEAQTFQYSRGWTN**G*KR***SGGYGVGGPYDLGTGEGMLGVDPLEVIIPPGHLQMSLNPQSRYRAGEYTQYLV***KR***SATLGNTKNKPMWIGTIVGMDTGVDGAGGSSITSPNAKPVVGESVTSANQRQRSGSAPSDF*

>*Bourletiella hortensis* (Collembola) corazonin

MDQSSLASSITSNETVPKKTKLSTGILWKSVVVLLVVVIAVCEAQTFQYSRGWTN**G*KR***SRGMENRYPSGPGPAQPFDIDVGVVEVPVDDVPVPLRINLSPQLRTRSTDYNQLVNRKVGRGAIHKSATWDGTLMGLEPENNSNNNAEF*

>*Orchesella cincta* (Collembola) corazonin

MAIRGDSISSSLPFPLGKSSYSRKFSRTY***M***SWTALCVLSLYIIATAHAQTFQYSRGWTN**G*KR***SGPTGGAPAVGLVDLENAYLAPEASDLGLTFPGHIQMNLSPQARLRTLDYNQYLARKLGVGSTGNLNTRKQPLWFGTIMGMDTADSPAPASQTASGSSSSNPSGTSTDF*

>*Pogonognathellus sp.* (Collembola) corazonin [GenBank:GATD01085393.1]

***M***AKLLMTWKIFLFVLLTAIMGCEAQTFQYSRGWTN**G*KR***SSSAGGSAYPPSALNQGQLYDMDNGILGVGLPIDDPTASVSIPAAVHFLNRKLALGSNSNGNVINSRKPQPIWIGTIMGIDQNQELSTSGRPSNSNNNSNNP…

>*Sminthurus viridis* (Collembola) corazonin [GenBank:GATZ01016940.1]

***M***DHIIAHSTSGSSSVSSQSKQKIINGVFWRALLVMLIMMVASCQAQTFQYSRGWTN**G*KR***SGPNPVPVGYPYGQGSSQLVDLENNLMGVPLEEPSLAMPGLHVNMNSQGRVRPVDFSQEG***RRKR***AQQP*

>*Jordanathrix leptothrix* (Collembola) corazonin

…NESAWKIIIVMLVVEMMCCPCHAQTFQYSRGWTN**G*KR***SGASRVGYLPGQGSSRVFEVDSGVLGVPLEDPSVSVSVPATHMNISPQGRVRSLDYNQILNRKMSNNGFTSRKQPVWIGTLMGADQEANLNNSADF*

>*Campodea augens* (Diplura) corazonin [GenBank:GAYN01141637.1]

***M***NTFSILTCIVLSLAILCCLQWPSTEAQTFQYSRGWTN**G*RK***RSDPAFSQQQSFVRHPHRGSTIGLTNEGEARLKNLINLFGNDAFKGLQINGKDGSLEDRPNSLKNRNLWLVLQTPSENENEDEIEDEVS*

>*Lepidocampa weberi* (Diplura) corazonin

…YSRGWTN**G*RK***RSSPPAPPTLQDPALIRKLLRSQHHQPxLWRERGTTMEEGMISEPSCVSLGRTSRQVGTSFPQRQSVAQTGTCG*

>*Occasjapyx japonicus* (Diplura) corazonin [GenBank:GAXJ01098411.1]

***M***SRHIPSSILVLLLLSAIVVLVQSQTFQYSRGWTN**G*RK***RAEAPGTRTAGFPFA…

>*Catajapyx aquilonaris* (Diplura) corazonin

***M***SPRQVPVAILVFLVMSTVISFVQSQTFQYSRGWTN**G*RK***RADAGPGASRTSTLFPFGPLPQSPSGTDSDLASSSPADSYQRYKSIMGSRLKDVQKMDQLQNGLRKMLLNLQSVPSSFVDWEDVDDV*

>*Pedetontus okajimae* (Archaeognatha) corazonin

***M***RITRSTTTVFMLFFIVGSVMAQTFQYSRGWTN**G*RK***RAVPVAPLVAGSQTRTSEPCGVPWISSLYKLRSSMEPRVNNPTQLLPSDINTDRLQRMLDAEAPLLD*

>*Machilis hrabei* (Archaeognatha) corazonin [GenBank:GAUM01171188.1]

***M***SHRTTALFMLVFVIGSAMAQTFQYSRGWTN**G*RK***RALPVAPLVANSQTRTEPCGVPWSNSLYRLRAAMEPRLIPSQLSPGDMNTERLQRFLEAETP…

>*Meinertellus cundinamarcensis* (Archaeognatha) corazonin [GenBank:GAUG01033537.1]

***M***NRTLTVLLLIVVLVGSALAQTFQYSRGWTN**G*RK***RGMPVTPIEINPQSRPTDQCSGPWSSALSRLRSALEARNNPSQISSSEINIERLQRLLDENGPLSD*

>*Machilontus* sp. (Archaeognatha) corazonin

***M***KRALAAILLLVIIVGSSLAQTFQYSRGWTN**G*RK***RGMPVAPLAVSSQTRPVAELCGGSWSTALARLRSALEQRNIPSQPSSSEINIDRLQRLLDENGPLVD*

>*Tricholepidion gertschi* (Zygentoma) corazonin [GenBank:GASO01239244.1]

…FTILLLFWCGVATLAQTFQYSRGWTN**G*RK***RAPTLLTPVVEDPVPCDVRITDPY***RR***LRSVLENRVPDWRGPIPDRFHRVSPSEDDSLDN*

>*Thermobia domestica* (Zygentoma) corazonin [GenBank:GASN01385153.1]

***M***YLYHRLCILILLWCTGAVIAQTFQYSRGWTN**G*RK***RASAASMLPVSTEDAAPPPSPCDPRTVDQYQRLRSVLDGRLQDWRIVSPERF***KR***STSSSVSKQDGGR*

>*Atelura formicaria* (Zygentoma) corazonin [GenBank:GAYJ01020859.1]

***M***YPSRLLAVLLLLWFAAATVAQTFQYSRGWTN**G*RK***RASASMSSPMDGISPCSAENTPDQFQRLRSVLEGRLQNWQLNGDRY***KR***STVGRHSASRN*

>*Nicoletia phytophila* (Zygentoma) corazonin

***M***CSSRILAVLVLLWLAAAAVAQTFQYSRGWTN**G*RK***RASPSLMTSPVEDPAPCSTDQYLRLRSMLEDHFQDWRSPSQERF***KR***SSGRGAASNRI*

..............................................................................................

>*Anaspides tasmaniae* (Malacostraca) corazonin

***M***VARGGQLVIALFISCLLIQVTPQTFQYSRGWTN**G*KK***RSGDSAVVRQPEETATYVMQPDTDVSVVNTEDLDYTPLKQLNLNALDYLDTGFKIADLQGLKTSELDDIGVKLADMRGLGWK…

>*Hanseniella* sp. (Symphyla) corazonin1

***M***KRIETALILLAIILLASAQTFQYSRGWTN**G*RK***RSDQVTKFYTPLISTTGVI***RR***PIGEHREKTLELL***KR***IYQKLQENNRDQISSLVSQEDMTSGMT*

>*Hanseniella* sp. (Symphyla) corazonin2

…QYSRGWTN**G*RK***RSGHVTSYLQPIELSAQSDSDL***RR***LSLL***RR***IYEELQNVNPDSEVSALT***RR***DVTSQIASTDPETISS…

..............................................................................................

>*Drosophila melanogaster* corazonin [[FBpp0082386](http://flybase.org/reports/FBpp0082386.html)]

***M***LRLLLLPLFLFTLSMCMGQTFQYSRGWTN**G*KR***SFNAASPLLANGHLHRASELGLTDLYDLQDWSSDRRLERCLSQLQRSLIARNCVPGSDFNANRVDPDPENSAHPRLSNSNGENVLYSSANIPNRHRQSNELLEELSAAGGASAEPNVFGKH*

>*Daphnia pulex* corazonin [[Dappu1_443193](http://genome.jgi.doe.gov/cgi-bin/dispGeneModel?db=Dappu1&tid=443194)]

*M*FINQYVRYSSSSFAMAARLYFVLLLVVVSAMAQTFQYSRGWTNG*RK*RSDPSFVQQQQWIQRNGHPMVIPAEFRSNSFEDWSRYRINGEKVNEDGDSWLVHVSHCAKLATSLGSVLKNKDAKSDDNPLIDVIH*

Corticotropin releasing factor-related diuretic hormone (CRF-DH , DH44)

>*Nipponentomon nippon* (Protura) CRF-DH

***M***THSWHTLLFCFACRLLFISGTEARLLEYQPDVQLDPDPKLDTSGEDPRIELLNELILFPPFRQRVRLLPIENAAQVARLNGKLDSNLNRGLNTWLVHHGIQQRN***KK***GQNIEGLGGIVGAAGGGGGGGGDAGPSLSIVNPLDVLRQRLMLEFARRRLRQSQTQIAANAEILKTI**G*KK***RQGHSNSHLNRAQPDDSSELLNWIEPSRSDD***RR***ALPSTYSYRRQQNSEWRQ*

>*Acerentomon* sp. (Protura) CRF-DH [GenBank:GAXE01139229.1]

***M***KRCQSASSALQFCPFSLLLFVSLFASFECRLIETQDEPHSPEFNLNYDDAIKPNPRADDEARYELPNNDARSPLHLDYHRVNDNFQNKYDAAPPKLDNIESLNTWLLHHGVNR***KKR***AHSSEGGMGVSEEGPSLSIVNPLDVLRQRLMLEFARRRLRQSQTQIAANAEILKSI**G*KK***KSGRERNSAMHRSPQLDDSSSSGEISPIEMLNWVQTDGGRNSQQKQAQSKNRCGLPACDYQRLWRP*

>*Filientomon takanawanum* (Protura) CRF-DH

***M***KRCPSPPSSLWSSFLPSLCCPLSILLLLSIFASSVESRLIDSQDSRSPVQFNSHSYPDDAIKADDTRYELPDEVFLSRLLQRNPAQFDYRRANDHFQRKYDQTPKLDTIDILPSHELSTWLLHHGVPRN***KR***AHSVEGGGLVGGSDEGPSLSIVNPLDVLRQRLMLEFARRRLRQSQTQIAANAEILKSI**G*KK***RAGPQRSTAMHRNPETDEVEMSQAEMLNWIQGRSPKQTTKDATRHRLTSSDSYQRQWRP*

>*Anurida maritima* (Collembola) CRF-DH [GenBank:GAUE01053205.1]

***M***SSIFGIRGIGFMLLVLCFLGTEGREIVGL***RR***ERNENGEMGDRIQIQEHDIHPSPFKNFLDQGKEAEEENDGQNKGSPEWETVIDPKLYIITEYDGKGESEATSNANEVSESEGMEESPFSRL***RR***NSNAGHSLSIVNPLDVLRQRLLLEIARRRSLKQNHHQIIANDLILKNI**G***

>*Anurida maritima* (Collembola) CRF-DH-like[GenBank:GAUE01008646.1]

MGSKSGSGSLRAEKRAVYQLHHRTNSAWQELINLINFELIRPKSAGKSKSRSTFNCSRGWTSLILIVIGVILLSEFQMVSSQPVIPDKQIEKPSSTIKSQVSSHHPHRHHHNQPRHSKHERGLRQHHLKQFKESFEMLDTSQSLSIVTPLEVLRQNMLLELQR***KR***IRHARLKQVQFNQEFLKKIG*

>*Tetrodontophora bielanensis* (Collembola) CRF-DH [GenBank:GAXI01012215.1]

MQQQEIPQFLDEQQFKKNSLQKFVHDYGGPTIPHHIRTPPLRYLYQNNGNPPIEQQYNGLNPKLKFGRQGGGGGRGTTDQQHLWNSFHDNKNEIGGIIDEIDGNKLLNDGKWEEWELELDPTYYIHTTKESPSINNIQQR***KR***RNAGPSLSIVNPLDVLRQRLLIEIARRRLHSKNQQITANTEYLRTI**G*RR***RR*

>*Tetrodontophora bielanensis* (Collembola) CRF-DH-like[GenBank:GAXI01154735.1]

MFPTKQDIIIGDKSFDRISLSKWRTFLSINNLMKFKSPKLSSWTLITFIVILSLALILESSTVSSLPTKSKS***RR***DHHSHNYYHHNHHHHHHHHHHNRSSRHHHVKQPILEMHEPVADSSGSLSIVTPLDVLRQNMLAEMQKT***R***MRHAKNKVVEYNREYLEKI***GRRRR****

>*Podura aquatica* (Collembola) CRF-DH

…CFCLIFLITITTTEGIGVSGGRLTNFGPGHLGALYTKNSENNDEEVENVNKVSKAKFDKNEMLSSPEWETVVDPNVYIVTEYENSDEAR…

…RRNLKQNQHQIIANAEILKNI**G***

>*Podura aquatica* (Collembola) CRF-DH-like

MMMMMVKKLLKNGFLASLNSKKSIVIFLLVIVMMSFCVESASSASLHHKNSHKLRPSSHQISQTRHRHHHTLRPRQFHTRNFVDAPDSSSSLSIVSPLDVLRQNMLNEMQKKYKREE***KR***QLVESNREFLAKI**G***

>*Folsomia candida* (Collembola) CRF-DH-like[GenBank:GASX01090537.1]

MTYRLAEDSSYQFPSPLPQIVTKCSSTSAKVVAMSVVRSQAFTNHASTGSSRARFNNLLCNLTPKITSICKSTKYISPIRRSCGFATSLLQVNKISCLVLVTIALMATLAGSCEAVPLRPSRSIGHHHSSHRLSLPSISVPSSTGSEAAASSASLHGLDATSSLSIVSSLDILRQNMVMEIL***RR***KHRQNKQRQVQLNKEILDRI**G***

>*Bourletiella hortensis* (Collembola) CRF-DH

***M***TGGSQNYMWSLLLIAIALLSVSSSADCLPVDLAVARDPSSALEPSKYSSNGRETAGEEFNLESAGAGAGANYQRLVHQGQLYLPALFQSERLSSSSQSAPGGSLQGSDYPLNTQNEEDSIGTEWETVIDPHLYVLNEWSSSRSPRVQQRK***KR***SAGPSLSIVNPLDVLRQRLLLEIARRKMIKSQNQILANAEILKNI**G*RR***RR*

>*Bourletiella hortensis* (Collembola) CRF-DH-like

***M***GHHCQRQCLIILFTIFLLATLDNVSGRTITSVLTPPPRPRNSESSLDHSIQQAVDRSSSSLSIMSPLEVLRQSMAAEIYRQRLRQA***KKR***QMATNKEYIQRI**G*R****

>*Orchesella cincta* (Collembola) CRF-DH

…ASNFLRFHHLPDQSTFTTGENHFQRAPIQKRK***RR***NSGMGPSLSIVNPLDVLRQRLLLEIARRKMRQSHNQIIANEEILRKI**G*R***…

>*Pogonognathellus sp.* (Collembola) CRF-DH [GenBank:GATD01101213.1]

***M***ILFLVVVFSSTSSVLCVPLEQPELTGSFLSSKHKQQSPYDLESKGQLYLPSSLNPSQEQRNDPSFYKPRLQIQERT***RR***DAGPSLSIVNPLDVLRQRLLLEIARRRMRQSHRQIMDNAEILKQI**G*RR***K*

>*Pogonognathellus sp.* (Collembola) CRF-DH-like[GenBank:GATD01014822.1]

MSQNCSHTRNRPCSSHSTSSCSIINVNNIINIINSNNLRRPKSCNSWLRTTFFLLICFVLILDLCANVSGHPLNKSRATHDFKHSSRSLSRFQRSQKQNVENTESGLSLSIVSSLDVLRQNMVLEM***KR***QHIRHAKHKQIETNKEILDRI**G***

>*Sminthurus viridis* (Collembola) CRF-DH [GenBank:GATZ01098618.1]

MESHSDIHRKRIQGGDPGTTFTAPSSNWALTLVTIILFLVSSAQCLPVDLDRPEPPQLSLTTQGDVRQPNQQLVHQGQLYLPSLFQNGRESMSSQGSDYPVVSQSQDDSLGTEWETVIDPHLYVLNEWSPSGSRIQQRK***RR***NAGPSLSIVNPLDVLRQRLLLEIARRRMIKSQNQILANQEILRNI**G*RRR****

>*Sminthurus viridis* (Collembola) CRF-DH-like[GenBank:GATZ01006265.1]

***M***TRLSFSLRPIALVLFIIIGILHLSTVTGKSIP***RR***AHVPHSSETFEYIQSDRSSSSLSIVSPLEVLRQTMAMEVARSRVREAKN***R***QLAINSEFLRKI**G*R****

>*Campodea augens* (Diplura) CRF-DH [GenBank:GAYN01002200.1]

…NHKPSNQWERATLPRLYVLNEDTNEEQKGTNSLMSPRS***KR***SSGSLSIVNPLDVLRQRLLLEIARRRMEESQSQIEANAEFLKTI**G*KR***DAHYYDSEDSWYPSPSSLFESSDSER*

>*Lepidocampa weberi* (Diplura) CRF-DH

***M***ASSSRVLPVLIAFLAGTSLTAGSEQTNRNDRINISNGVAAGLQLSNPRTWLPGSQTTILADESTGSRHHKAEGDYDRPVSPRAYFINEDHRQQVSARS***RR***SAGGSLSIVNPLDVLRQRMLLEIARRRMEESQSQIEANAEFLKTI**G*KR***NANDDDKESRRWSSSHPDLSSLYDNISSER*

>*Catajapyx aquilonaris* (Diplura) CRF-DH

…LWCSCSSSPGSHHNRQHPLEEPW***RR***SPRSFSLSVVNNLDVLRHLLLHGRRYPGGAHHFHAQVEANAELMRTL**G*KR***NAHQDPDSYERTPLILYSKLFSTPANRR*

>*Pedetontus okajimae* (Archaeognatha) CRF-DH

***M***SLTTTAFVVFMMLVGYNEAKSMLSKDVWDFEREQGTSSNEEPEWKALPHPHFYVLTESASSQSEPVPV***KR***TSPSNSLSIVNPLDVLRQRLLLEIARRKMRESEQKIIDNREFLNKL**G*KR***TNERFNGALTPIDVPGRINLALSDQLQSDEINKGSNLIPKDIFVRTSL*

>*Machilis hrabei* (Archaeognatha) CRF-DH [GenBank:GAUM01184133.1]

***M***FTEMSLTTACIYLVFLLGYNEAESVLSKDAWDSQREPKTSTFDETEWKAVPHPVYYVLTESRNDKTEPVLE***KR***TSTNSNSLSIVNPLDVLRQRLLVEIARRRMRESETKIMANRHILDNI**G*KR***TQESSNTFLTSEALPGRLRVEQNDRLQPEEISRDINFNPKEIYVRNSL*

>*Meinertellus cundinamarcensis* (Archaeognatha) CRF-DH [GenBank:GAUG01019754.1]

***M***SLKAVWFITTSLVAFGESQALVSSNNWNLQDQQKETVADPTDWTTITQPHFYVLTESESSQPQLWQEK***KR***TGAGNSLSIVNSLDVLRQRLLLEIARRKMRETEGQIIANRIHLNTI**G*KR***TSQDDDRPVNVLSPLVVPARFHTISNKDSNWNDVSEEYLKMQNTDANADRPLSLNAHY*

>*Machilontus* sp. (Archaeognatha) CRF-DH

***M***YLKAVWLIATSLVVYIEGSALATPQEWTFEQQEETVADPTEWTALSHPHFYVFSEPQRTLQQSERE***KR***TGAGNSLSIVNSLDVLRQRLLLEIARRQMREKEDRITANREILKNI**G*KR***TSKDNDDRPLGVLSPLVVPGRLHTLPSTDSQSDDVSEDSESLRPHNSYASADSVVRPLSLNARF*

>*Thermobia domestica* (Zygentoma) CRF-DH [GenBank:GASN01407760.1]

***M***SPSYLTTFVTFLVCCITVSNAATLTIPYNRLMPLM***KR***PRTPSFPILLGGEPVLSGEEESQMLSQYGQAATPILDPHDDLSEWETVDDPRFFVLTDSDRNLDG***KR***V***KR***NGPSLSIVNPLEVLRQRLLLELARRKMRVSANQIQANAKILDTV**G*KR***DVTAQASDNESKSKNEEFPRDNVNNSNENRLNSEETGNTRSESSYYPLKSSNHRWMGDVLNNFASQLHQTHQQETF*

>*Atelura formicaria* (Zygentoma) CRF-DH [GenBank:GAYJ01034371.1]

***M***KLQTSTLIAVLLSCVALAAALPLSVPYRYPLLQEGQEELLPSVVSRLRQVASNLRRGEDDIPSWQSVEDPGFYVLTELEDKEARRV***KR***NGGGPSLSIVNPLDVLRQRLLLEIARRRMRQSEGQIQANRELLKSI**G*KR***DTSSQLLHSSSRSARSSEDLKETYQDDSRMKMLSRKDPTDQ*

>*Nicoletia phytophila* (Zygentoma) CRF-DH

…APPSWDSPSDTWFDLADEEDEGRRV***KR***NGAGPSLSIVNPLDVLRQRLLLEMARRRMRQSEGQIQANRELLKSI**G*KR***DTTSSTQSFNKQRPMSSIGGSDTRWTDPITNNDHELHSPLRQEVL*

..............................................................................................

>*Xibalbanus tulumensis* (Remipedia) CRF-DH

…WTSVNEPRFYVLTEKEGGAQNKRSK***RR***GDGSGVPSLSIVNPLDVLRQRLLLEIARRRMRHSQTQINANAQLLKNI**G*KR***DTYR…

>*Lithobius forficatus* (Chilopoda) CRF-DH

***M***PNNSLLAILLFWVSFSMATRPMPLSVTWYSPVSARSQRANQMSDSSSTNDGSDSQESISVIETSPDATTNLMEKSTSILYGDEPPLTSLRLNKMVDDQWLRNTLEPELEDTQADSSITDQHSPLMPEPRNMADLFDLEKTRSK***KR***GDGHSLSVVNPVDVLRQRMLMEAARERMRQNQDQIEANAQLLKEI**G*KR***QIWSQQPRWIRAWDATKVAFETSRTQAS*

>*Hanseniella* sp. (Symphyla) CRF-DH-like

…DVLRQRLLRDMAI***RR***LQQSQSRIDANTDLLRTI**G***

>*Eudigraphis takakuwai nigricans* (Diplopoda) CRF-DH

***M***VCPRTSLLLPLIHAFLLSLGWCETQTESVPTPITDLHSAGSSRLPDPSSPSSGRPLPARVRRYADSENGEYRAQLIQSAEDALTDTLHKLNAIAPDRINGSPSADGDQTAMLFSKDDDQEASDSDQRPRWRSAATDHGVAMGSRSFPLPLLDVARFGHY***KR***NGGPSLSIVNPLDVLRQRLLLDMARRRQKETQERIESNAQLLNSI**G*KR***EISSRQPSTWNDPNPYT*

..............................................................................................

>*Drosophila melanogaster* CRF-DH [[FBpp0081558](http://flybase.org/reports/FBpp0081558.html)]

***M***MKATAWFCPVLLTLLCATRLVCTAQRGAVGAGGAAGGSGAAAGGAEVGGSGRTNGYPLDYPDGTRNSQDDFLLA***KR***NKPSLSIVNPLDVLRQRLLLEIARRQMKENSRQVELNRAILKNV**G*KR***VVLRGGGGGGGSGAGGLAPKVSRRYRQQWPVERELERERQRERERERDAVREEQLDRQQLLPWKHFPSQLWSYGWALSPYKESSQLQFADSQQSASTGPQSQALPKQLQLLSYAKKPLDVAGMSLARHRVSGNEANETNHENDDGNGASKNPARYVDDGDNEGEDSYNDVGTEGVGLGLGMGVGLGLERFEVLEDKPNWANEEPNELVVVNANDRVPWSFPYRFHKSQHNVN*

>*Daphnia pulex* CRF-DH a [[Dappu1_443196](http://genome.jgi-psf.org/cgi-bin/dispGeneModel?db=Dappu1&id=443196)]

***M***RWHVATLAAVWLIIMGWCGSAVMGQPIAGSMDQPHPAHHPPAGQTIPEMWALMDPADGPPDTIRDGDDLNGDLYYFPAVPIM*KR*QQSLDDDSGWISQDRS***KR***QSQSHSGSSGNHHSQLSIISPIEALRSRLRLEMLRRQYGNQIKQNQDKLERV**G*KR***R***KR***SDQSSPIAVDGSDE*KR*MSQPSVAPAAIKAP*

>*Daphnia pulex* CRF-DH b [[Dappu1_443198](http://genome.jgi-psf.org/cgi-bin/dispGeneModel?db=Dappu1&id=443198); GenBank:[EFX89039.1](http://www.ncbi.nlm.nih.gov/protein/321478081?report=genbank&log$=protalign&blast_rank=1&RID=XNSW7UE8015)]

***M***RWHVATLAAVWLIIMGWCGSAVMGQPIAGSMDQPHPAHHPPAGQTIPEMWALMDPADGPPDTIRDGDDLNGDLYYFPAVPIM***KR***QQSLDDDSGWISQ***KR***AGM***KR***NRVRVEKILDTVHRSIVLGCVGLTVYGFYLAGLRFHRFYTVLKPAGEER***KR***LKELELLSEGQDKSADSQEFFLPEIPKLDKEKF*

Calcitonin-like diuretic hormone (CT-DH/DH31)

>*Nipponentomon nippon* (Protura) CT-DH

***M***KMNVSVMPCALLLSVLIALVVNTYGVPFARFDNPSEDTVDNDMLMELINRLSQYNKLGYQQNSA***KR***GLDFGLGRGYSGSQAAKHLMGLAAANFAGGP**G*KR***KRETAAGQQSQPEIMVPVPAGAL*

>*Acerentomon* sp. (Protura) CT-DH [GenBank:GAXE01138372.1]

***M***RMDAHVVPSVLLLATILALISHCYSLPYTRFDTIQGSDDGSADSDVLMELVHKLSLFNKEGFQNSE***KR***GLDFGFGRGYSGMQAAKHRMGLAAANFAGGP**G*KR***KRETGASQPEIMIPVASLS*

>*Filientomon takanawanum* (Protura) CT-DH

***M***SVPLVPCVVVLASLLALVSNANGVPFSRFDNPSDDASDSELLMELIHRLGQYNKQGYQNSE***KR***GLDFGLGRGYSGSQAAKHLMGLAAANFAGGP**G*KR***KRGADETQPERTVPVSSGL*

>*Anurida maritima* (Collembola) CT-DH [GenBank:GAUE01009999.1]

***M***NRAFFWTLSGSCILLMTLLQLSHAIPIYSQTYQNRHKDYLVEVEDPDAVLDMLAKLGQSILRAQHEGSNA***KR***GLDLGLGRGFSGSQAAKHLMGLAAANFAAGP**G*KR***KREYATSRGNGDFYTFGSQPEVSSAPLSNESDWNNGPKE*

>*Tetrodontophora bielanensis* (Collembola) CT-DH [GenBank:GAXI01155353.1]

***M***HRGIMWFLLGFVITFITIIHSSQAIPFYPSAYTNSRHKDYLVEIEDPDAVLDMLARLGQSILRAQHEGSNA***KR***GLDLGLGRGFSGSQAAKHLMGLAAANFAAGP**G*KK***KRGDSVSELGNSLITYGIPSQDLNSPVSNSNEWIVGGSKE*

>*Podura aquatica* (Collembola) CT-DH

***M***MHRGLVWLTISSFTVLFIIKSSQAAPFYPGTYNHNSRHKDYLVEVEDPDAVLEMLARLGQSILRAQHEGSNA***KR***GLDLGLGRGFSGSQAAKHLMGLAAANFAAGP**G*KK***KRDGTIMDNGLLLRYGLPES…

>*Folsomia candida* (Collembola) CT-DH [GenBank:GASX01009791.1]

***M***HKTSMWMIVGCISVMAFLRVAFAYPNYQPAPEGFRPMVEVEDPEAVLEMLARLGQSILRAKHDGSNA***KR***GLDLGLGRGFSGSQAAKHIMGLAAANFASGP**G*KR***KRMEGALDYPVSSSGLDVPPLSGSEWYNKS*

>*Bourletiella hortensis* (Collembola) CT-DH

***M***HRALGWVLAAFVITFMALLHSSHAIPFYQSPYNNARHKDYLVEVEDPDAVLDMLAKLGQSILRAQHEGSNA***KR***GLDLGLARGFSGSQAAKHLMGLAAANFAAGP**G*KK***KRGSDMSSAGRGDMTFGLSPAAAQDYLPVSPASSNEWALGNKE*

>*Orchesella cincta* (Collembola) CT-DH

***M***HRGAIWAMTGCIISLVAILQSAHAIPFYPSVYTNARYKDSLIEVDDPEAVLEVLAKLGQSILRAQHEGTNS***KR***GLDLGLGRGFSGSQAAKHLMGLAAANLAAGP**G*KR***KRTGPKSYDDSMQDSPVSLATPNDWMMENQK*

>*Orchesella cincta* (Collembola) CT-DH-like

***M***HLKLQHSITLILLSFITLAYLSERCYGASVKFVPELKRAESNYKWFKIDKNGSQEEKATPLLHSQFEDEDPRLNDENIPLPLALKMLYE***KR***GMFDLGTGRGYSGFQAMRGNLGLAFASPGSH**G*KR***K*

>*Pogonognathellus sp.* (Collembola) CT-DH [GenBank:GATD01016084.1]

***M***TRIAVFLWLGVSLTLFSILASSNALPFYPSVYTNTKNKDFLVEIEDSDAVLDMLAKLGQSILKAQHEGTNS***KR***GLDLGLGRGFSGSQAAKHLMGLAAANFASGP**G*KR***KRGEELKNINFRYVSPDISDIPIQDTNAEWILTNPNRQ*

>*Sminthurus viridis* (Collembola) CT-DH [GenBank:GATZ01007841.1, GATZ01007842.1]

***M***HRGAVWILAGFAITFMALLHASHAIPFYPSVYNNARHKDYLVEVEDPDAVLEMLAKLGQSILRAQHEGSNA***KR***GLDLGLARGFSGSQAAKHLMGLAAANFAAGP**G*KK***KRGDMSSLSSTGRGDMTFGVPASAQEYLPVSPVSSNEWMGNKE*

>*Jordanathrix leptothrix* (Collembola) CT-DH

…HLMGLAAANFAAGP**G*KK***KRGDDISSFSSPGRVDMTFGVPSAVAQDYLTASPASSNEWMVGNKE*

>*Campodea augens* (Diplura) CT-DH [GenBank:GAYN01125587.1]

***M***NRTVISCFLLCATVLMLTRQSRGVPISRGKYSVEIEDPDYVLEVLARLGQSIVRAHDYPNSS***KR***GLDLGLGRGFSGSQAAKHLMGWTAANYAGGPG***KR***KRNSITDFAAAAAAERPIAS…

>*Lepidocampa weberi* (Diplura) CT-DH

***M***SVNGMILSSFLIAISFVLLTSHANGVPLNRGKYLVEIDDPDYVLEVLARLGQSIVRANDYPNSS***KR***GLDLGLGRGFSGSQAAKHLMGWAAANYAGGP**G*KR***KRNSGDMDGAANPLFPAPEVESETEMTYKS*

>*Occasjapyx japonicus* (Diplura) CT-DH [GenBank:GAXJ01111602.1]

***M***NSAIFAGLLLVGSILLLTSTPAKGSPVDRLEYEGMYGPEDRLRNLILDNLVKSYYRNEAANS***KR***GLDLGLGRGFSGMQAAKHLMGLAAANFAGGP**G*KK***KRSYMGSQPEVDHS*

>*Catajapyx aquilonaris* (Diplura) CT-DH

***M***MNSTLFACLAIMGSLLLLTSTPATGSSIYRYQQYPSLEDQANRIWESLILNDLMRSSDAANS***KR***GLDFGMGRGFSGSQAAKHLVGLAAANFAGGP**G*KK***KRSMESEAEDN*

>*Pedetontus okajimae* (Archaeognatha) CT-DH

…GIDLGLGRGFSGSQAAKHLMGLAAANFAGGP***KK***EADSSL*

>*Machilis hrabei* (Archaeognatha) CT-DH [GenBank:GAUM01178264.1]

***M***NASLVTFAVLLSTILLLRATNSSPVSRHNTYLLESPEMEPDYMLEMLARLGQSIIRANELENS***KR***GIDLGLGRGFSGSQAAKHLMGLAAANFAGGP**G*KR***KRTAHFDDDSQEDASTM*

>*Meinertellus cundinamarcensis* (Archaeognatha) CT-DH [GenBank:GAUG01245552.1]

***M***NLALVSFAVLLSTLLLLGTTTASPILRHNSLLPDSPEVEPDYMLEMLARLGQSIIRANELENS***KR***GLDLGLGRGFSGSQAAKHLMGLAAANFAGGP**G*KR***KRTASQYDDEQDDVTSV*

>*Machilontus* sp. (Archaeognatha) CT-DH

***M***NATLVSFAVLLSTLLLLGVTSTPMSRHSSLLPDSPEVEPEYMLEMLARLGQSIIRANELENS***KR***GLDLGLGRGFSGSQAAKHLMGLAAANFAGGP**G*KR***KRTVQYDDDQDDVTSA*

>*Tricholepidion gertschi* (Zygentoma) CT-DH [GenBank:GASO01244605.1]

***M***NSSSLLGFATLLGAVLVVSVSCSFGAPLNSHKAFLSELDDPDPDYVLEMVERLSQRIAQANDLENS***KR***GLDLGLSRGFSGSQAAKHLMGLAAANYAGGP**G*RR***RRSSALEENSSSS*

>*Thermobia domestica* (Zygentoma) CT-DH [GenBank:GASN01019819.1]

***M***NPSSVISCTILFGAILFFGVQISQSAPVNSHKSYISDLDEPDPEYVLEMLARLGQSIMRANDLENS***KR***GLDLGLSRGFSGSQAAKHLMGLAAANYAGGP**G***RR*RRNAPWEDEDNFTS*

>*Atelura formicaria* (Zygentoma) CT-DH [GenBank:GAYJ01313544.1]

***M***NPASLTSCAVLLGTFFLLCVHSTNSAPLSGHKSYISDMDEPDPDYILEMLARLGQSIIRANDIEPN***KR***GLDLGLSRGFSGSQAAKHLMGLAAANYAGGP**G*RR***RRSAAWEEDAFTS*

>*Nicoletia phytophila* (Zygentoma) CT-DH

***M***NPSTFVTCSLLFGAFLFLSIQTAHSAPYTSHKGYLSDLDEPDAEYVLELMTKIGNSIMRANDIEPN***KR***GLDLGLSRGFSGSQAAKHLMGLAAANYAGGP**G*RR***RRSASWEQDTYTS*

..............................................................................................

>*Xibalbanus tulumensis* (Remipedia) CT-DH

***M***NQIVATCLALFGVFLLLTASTARSAPLSSHLAETEDPDYVLDLLSKLGQSIIRADELENS***KR***GLDLGLGRGFSGSLAAKHLMGLAAANFAGGP**G*RR***RRSAGAKEGKPSDSL*

>*Anaspides tasmaniae* (Malacostraca) CT-DH

***M***SNSAVVFVSLVAVLVFITTVNANAINRDARAVVEIDDPDYVLELLTRLGHSIIR…

>*Lithobius forficatus* (Chilopoda) CT-DH

***M***HHVTKISLAVIVLTVLICSQTTNAEKV***KR***QVHTLVEIDEPDEVLDVLTRLVRAILRSTAYDAID***KR***NLDLGFSRGFSGSQAAKHMMGMAAANYAGGP**G*RR***RRSPEPMDTTLEE*

>*Hanseniella* sp. (Symphyla) CT-DH1

***M***ASFLSTQLMHLTVCILMFLCVLSALSSAYPTNLEKSRDDFDDTEEKVEFIRRVLTQLLQERPIGYSSD***KR***SLDMGFSRGFSGSQAARHLIGLAAANYAGGP**G*KR***RRRDAHEDLQQ*

>*Hanseniella* sp. (Symphyla) CT-DH2

***M***TSFLSTHQLMQMSLCVLLFLFVLTTFSSALPTSVDKSREDVEDTEEKVEFIRKVLTQLLNERQLGYTSD***KR***SLDMGFSRGFSGSQAARHLIGLAAANYAGGP**G*KR***RRRETAEDLH*

>*Hanseniella* sp. (Symphyla) CT-DH3

***M***SSFTIHQLLQISVVVVLSNMVLTSAYSFDVSPEYYEDYNNGIEPFWKVLALLKTGRKNSSH***KR***NIDLGMSRGYSGSQAARHLIGLAAASHTGG…

>*Eudigraphis takakuwai nigricans* (Diplopoda) CT-DH

***M***SPRGVSTLSLLAAVFCSFILLAALQTPVHSAALARSVRSAPDLNDPEQMLEVLEQIERNLNLIRESPIIETA***KR***NLDLGFGRGYSGSQAAKHLMGLAAANFAGGP**G*R***KRRSLDATASLSRSDSRFQDTDFATSV*

..............................................................................................

>*Drosophila melanogaster* CT-DH PA [[FBpp0079307](http://flybase.org/reports/FBpp0079307.html))

***M***TNRCACFALAFLLFCLLAISSIEAAPMPSQSNGGYGGAGYNELEEVPDDLLMELMTRFGRTIIRARNDLENS***KR***TVDFGLARGYSGTQEAKHRMGLAAANFAGGP**G*RRRR***SETDV*

>*Drosophila melanogaster* CT-DH PC [[FBpp0079308](http://flybase.org/reports/FBpp0079308.html)]

***M***TNRCACFALAFLLFCLLAISSIEAAPMPRYQSNGGYGGAGYNELEEVPDDLLMELMTRFGRTIIRARNDLENS***KR***TVDFGLARGYSGTQEAKHRMGLAAANFAGGP**G*RRRR***SETDV*

>*Daphnia pulex* CT-DH [[Dappu1_299999](http://genome.jgi.doe.gov/cgi-bin/dispGeneModel?db=Dappu1&tid=299999), GenBank:[EFX90445.1](http://www.ncbi.nlm.nih.gov/protein/321479489?report=genbank&log$=prottop&blast_rank=1&RID=XPGGXB27014)]

***M***SRFVMTIFFLLVACLALIVPGSAAPPRRPMLVDLDDPDSVMEVITRLE*R*SLLRNSDYEHQ***KR***GVDFGLGRGYSGSQAAKHLMGLAAANYAIGP**G*RKR****R*DTTESTPEDVKTGAIN*

EFLamide (EFLa)

>*Nipponentomon nippon* (Protura) EFLa

***M***TRAAHLGFIVLYYFFVWDLVAASSSVAKDDNWCSTPDGICNKDSNDPLDSDSVNSSEDSKSALSPDRDGELAQHFEN***KR***GLPVSDLFKLFVLSMNDKDRNQRDELFNVRQQHQHYHHDREYPMALRYGGASNNGFYDIPYRPV***RR***LGSEFL**G*KR***SAPLDSSWLSDQSSSMEPDS…

…L**G*KR***AMGSEFL**G*KR***AMGSEFL**G*KR***MGSEFL**G*KR***AMGSEFL**G*KR***AMGSEFL**G*KR***GMGSEFL**G*KR***AMGSEFL**G*KR***AMGSEFL**G*KR***SSDFSQSLENDQS***KR***ALGNEFL**G*R****

>*Acerentomon* sp. (Protura) EFLa [GenBank:GAXE01002455.1]

***M***TKGFLIGIHLLYLVIASVSSSTPNPNALEEGGSSCYSSNEGPCIEKTADDEAIENENANSKTAASQPELPNSLSDAEASSANRFHLASSD***KR***GLPVSDLFRYFVLTYGDKEAHEGPQL***RR***QQSDYLDRIHPFSLTRNPGSSTFYDIPHRSI***RR***LGSEFL**G*KR***SSQQGPEEDDDSISNESPEV***KR***ASFSKLFRNRFGPSVYSQ***KR***LGSEFL**G*KR***AMGSEFL**G*KR***AMGSEFL**G*KR***AMGSEFL**G*KR***GMGSEFL**G*KR***AMGSEFL**G*K*…**

…QLLD***KR***AMGSEFL**G*KR***AMGSEFL**G*KR***ALGNEFL**G*KR***SSDDLSESDFP***KR***ALGNEFL**G*R**** [GenBank:GAXE01016499.1]

>*Filientomon takanawanum* (Protura) EFLa

***M***TKGVQLGIQHLIYLIIVLSSAVKSTTNPNALSQGESQCYSSNDGCNERKQDEKTETIENENVNSHSELENTFNEGPSTDDANRFHSD***KR***GLPVSDLFRYFVLTYGDKDTNL***RR***EDPLSTRQHDYFERVQPFASNRVSGSASHNFYDIPHRSV***RR***LGSEFL**G*KR***SGVDSDSDQSDDSSEESNS***KR***AGLGRLFWNRYDPLSYASQ***KR***LGSEFL**G*KR***AMGSEFL**G*KR***AMGSEFL**G*KR***AMGSEFL**G*KR***AMGS…

…FL**G*KR***AMGSEFL**G*KR***AMGSEFL**G*KR***AMGSEFL**G*KR***AMGSEFL**G*KR***AMGSEFL**G*KR***SDGNEFLEKSDVDHNSEVS***KR***ALGNEFL**G*R****

>*Anurida maritima* (Collembola) EFLa [GenBank:GAUE01047606.1 + GAUE01034916.1]

***M***ILSEKRLSVLWFFAFFPANLFVSIESAEPPHLFQRPSDSPDETGGLSLSKDFLRYYILPYSTEPELPPHYAAGFKGEVPFSRHS***R***KLGSEFL**G*KR***SARGVSTEKHQVISKMQAEKNLEKESDATSLEEFVPSIPFFKPVPPVGGETKP…

…FL**G*KR***KMGSEFL**G*KR***NGSSEDLQGSEKSSGLSSSANET*

>*Tetrodontophora bielanensis* (Collembola) EFLa [GenBank:GAXI01153395.1]

***M***MMKVECLTSYCPLLILLIVCKTIVVAKTSTGSMIELPSTTTSQSSSSLSSLKLLPKDDNHNNQKSNDGRSEMNKIVKYVRAAKYLQSPNGENSMMLIQSRPVRA***R***LGSEFL**G*KR***SEMPIHEGKSRIQ***KR***RAMGSEFL**G*KR***RAMGSEFL**G*KR***NYQIPQIDEYYTISSETGGNGGTGTDWTKPNESQIYIEQGIEPLSYQ***KK***NNNNMRRASDYI**G*KR***KIHDYPTWKQPTLNSRLIQFD***KKRR***VMGSEFL**G*KR***SSFQTGKDELNEFGSEKGNF*

>*Podura aquatica* (Collembola) EFLa

***M***KMDNSLCLSFLFTTMIFSITSSAHVNSFHNSFFHPQVIGPGDFQPKTKEVFFFYPMRRGSESGQVLENLESLHNPQLYFQRAT***RR***LGSEFL**G*KR***NGEGEFESE***KR***KMGSEFL**G*KKRR***LGSEFL**G*KR***NAAGGEVSQYELS***KR***IGSEFL**G*KR***SSSGSEMDFEYYDSTVLPPPNANLNANFYHSPLSSSSSSLP…

>*Folsomia candida* (Collembola) EFLa [GenBank:GASX01011536.1]

…LS***KR***ARMRMGSEFL**G*KR***RLGSEFL**G*KRKR***GALGSEFL**G*KR***SPASFLLRPSPDFSW***KR***ARLGSEFL**G*KR***QPSPPHSLLPTTNDF*

>*Bourletiella hortensis* (Collembola) EFLa

***M***RAERVALPLLVVLFITKFSLAVSDGQRLLQASDEDETFRSNFNPSNGRNGMGIPFRPV***RR***IGSEFL**G*KR***SEQPHPYVDESLFEPMED***KR***ALGSEFL**G*KR***RLGSEFL**G*KR***SSEPSWDGESMWGGPLNALATNENSLD***KR***ASPSQYVA***RR***RLGPWGDWTY***KR***GLGSEFL**G*KR***VLGSEFL**G*KR***SPTSGQNQLL*

>*Orchesella cincta* (Collembola) EFLa

…SEFL**G*KR***GMGSEFL**G*KR***GMGSEFL**G*KR***GMGSEFL**G*KR***RMGSEFL**G*KR***RMGSEFL**G*KR***DGDLDSFYPESSHQDMSPSTNH…

…DLVNLVD***KR***SMGSEFF**G*KR***KPSGDAFSW***KR***GRLGSEFL**G*KR***SSSEPEQAYPYLQSEDNDMDLLTSHSGVGKVRDT…

>*Pogonognathellus sp.* (Collembola) EFLa [GenBank:GATD01061821.1 + GATD01085941.1]

…MTLHPNNAHYGRGSMGIPFRPV***RR***IGSEFL**G*KR***SVGMPMRTSEEMDEYSSPD***KR***GVGSEFL**G*KR***RLGSEFL**G*KR***SYSDDNVEDE…

…KNDLYDWSW***KR***GRLGSEFL**G*KR***RMGSEFL**G*KR***DGSSNVDGQQENQSGGGGGLGPREMTAVSSDNIRQLPN*

>*Sminthurus viridis* (Collembola) EFLa [GenBank:GATZ01016749.1]

…FHNNYPTPNHYGRNPMGIPFRPV***RR***IGSEFL**G*KR***SERLTFDDHQSEMSEA***KR***ALGSEFL**G*KR***RLGSEFL**G*KR***SEAEGCLSGLEEIPWPRPSQLYDQEVD***KR***ALGSEFL**G*KR***RQNLWDWSW***KR***GLGSEFL**G*KR***VLGSEFL**G*KR***DHAGLNEMRQDLESTGPASMLSQN*

>*Jordanathrix leptothrix* (Collembola) EFLa

…RPLIDDRLHDLSED***KR***ALGSEFL**G*KR***RLGSEFL**G*KR***SENVEMDDTLSLGEPLSQLQWARQHFPGSITFPASAVAHDYSP…

…EFL**G*KR***VLGSEFL**G*KR***NVGGGQDETVHGQVDSGALEIPSTQN*

>*Campodea augens* (Diplura) EFLa [GenBank:GAYN01130765.1]

…PPMGRDSSNAPTEQLHRQV***RR***ISMGSEFL**G*KR***NDYQDLETEMESPLPPLNYHFFNRAPLYNGYNSFNAPP***KR***RINYNLWR***KR***LMGSEFL**G*KR***LPMGSEFL**G*KR***AMGSEFL**G*KR***AMGSEFL**G*KR***AMGSEFL**G*KR***AMG…

>*Lepidocampa weberi* (Diplura) EFLa

***M***MMGIATLWAAFIRWLVGEKGISNKNTLGEPTLH***RR***QHSPNTNFKXNEASFCSFPLANMRCLSLSLSSPSSSPSPSSFILLFFLYFIFSVASGQHSSSSSGLLQQQRKDENETQGKSASVGNSSPLHQLPPLIRLV***RR***IGSEFPGNGYQESDADLESPLPPLNYHFFPRGQGGNSAFAFSAPP***KR***KINYYLWR***KR***LMGSEFL**G*KR***LPMGSEFL**G*KR***AMGSEFL**G*KR***AMGSEFL**G*KR***AMGSEFL**G*KR***AMGSEFL**G*KR***GGASDSGESENFSIQNSSNAQQESYVPNYPKP*

>*Occasjapyx japonicus* (Diplura) EFLa [GenBank:GAXJ01010969.1]

***M***QRGGKISLLLCFVLLCFSAGHARASQEDSAQSQPLSQQSVVSRDEWDAEAGPPDEPQAGEDEEENNEVEDMVQKRALSARELLHFFLMAMHDQDDGMFSGSSSSGIPFRPV***RR***MGSEFL**G*KR***SVEDPADLHLGDFPNIYMEPEDEGSPSDDVWIPQ***KR***RMGSEFL**G*KR***RMGSEFL**G*KR***RMGSEFL**G*KR***RMGSEFL**G*KR***RMGSEFL**G*KR***RMGSEFL**G*KR***RMGSEFL**G*KR***RMGSEFL**G*KR***RMGSEFL**G*KR***ALGSEFL**G*KR***SIESNSSQERSLNAEPHPAEPVNQTDAKLYD***RRKR***SE*

>*Catajapyx aquilonaris* (Diplura) EFLa

***M***HRVGKFSSLCFLFFLCVSAGYVRANQDDNASQPTNQQSAATRGDWDSEVAPPEELQPEDEEYDGVEDAVQ***KR***ALSARELLHFFLMAMHDQGESSGPYGGGYSSGIPFRPI***RR***MGSEFL**G*KR***SAEDAIDLHLGDFPEIYPEPEDEGAAAEDMWFPE***KR***RMGSEFL**G*KR***RMGSEFL**G*KR***RMGSEFL**G*KR***RMGSEFL**G*KR***RMGSEFL**G*KR***RMGSEFL**G*KR***RMGSEFL**G*KR***RMGSEFL**G*KR***RMGSEFL**G*KR***SVAPESNPSEDRSPSPTEGD***RRKR***SE*

>*Pedetontus okajimae* (Archaeognatha) EFLa

***M***LLCPRFLCAGPFLLCLCIHSCCSIASQTDNENALVLGRLDPEVYEAS***KR***GPPARDLLRFFLLAFHNNQQDGTKGSGIPYRPV***RR***LGSEFL**G**x***R***GLGSEFL**G*KR***SQQPYEFLQTYEPEDIENSVSDLSNIDRQRKLSPEQYEEREEESSNID***KR***SLHKSEFF**G*K***NYADLDSSGE***KR***GRLGSEFL**G*KR***SLGSEFL**G*KR***GMGSEFL**G*KR***RMGSEFL**G*KR***RMGSEFL**G*KR***RMGSEFL**G*KR***MMGSEFL**G*KR***MMGSEFL**G*KR***GLGSEFL**G*KR***ALGSIDANHSEPKQNEKYIKQL*

>*Machilis hrabei* (Archaeognatha) EFLa [GenBank:GAUM01065624.1 + GAUM01163451.1]

…QMSSEAYDERDEQPPNVD***KR***SPLSSSFF**G*K***SYSERDSSGE***KR***GHLGSEFL**G*KR***ALGSEFL**G*KR***RMGSEFL**G*KR***KMGSE…

…SEFL**G*KR***SMGSEFL**G*KR***SMGSEFL**G*KR***LVDDPELL**G*KR***MMGSEFL**G*KR***GLGSEFL**G*KR***SLNSANENEEGAEQIVDFQKQL*

>*Meinertellus cundinamarcensis* (Archaeognatha) EFLa [GenBank:GAUG01211503.1 + GAUG01034050.1]

***M***RLSHDVLCVGSFLLCICLEVCCYAAIPSDIASNEFDTESLDSEEFFAS***KR***GPPTRDLLRFFLLAFHNNNHDSSDGSGIPYR…

…MGSEFL**G*KR***AMGSELF**G*KR***AMGSEFL**G*KR***AMGSEFL**G*KR***AVGSEFL**G*KR***LGSEFL**G*KR***DMGSEFL**G*KR***TMGSEFL**G*KR***NMGSEFL**G*KR***AMGSEFL**G*KR***AMGSEFL**G*KR***GMGSEFL**G*KR***KMGSEFL**G*KR***DLDFLDSGVDDYDALENDESSSDVVE***KR***RTASEFVGNRKMGSEILGQGRMGSQLS**G*KR***LVGSEFL**G*KR***ALGTESMGKGAGELVKDS***KR***CKEANENPNDSL*

>*Machilontus* sp. (Archaeognatha) EFLa

***M***RHCFTLLSAGPFLLCFCLVTFCAAAITSDIAAANKLDSGFDPDEFVES***KR***GPPTRDLLRFFLLAFHNNHHDASEGSGIPYRPV***RR***LGSEFL**G*KR***NLGSEFL**G*KR***SLDPDGDIPQEAPMEYLIVDNEPSLNTDD***KR***APMGSEFL**G*KR***DEERD***KK***S…

…GSEFL**G*KR***VMGSEFL**G*KR***ALGSEFL**G*KR***SEVTEEKNKLCEEQKENSRNSL*

>*Tricholepidion gertschi* (Zygentoma) EFLa [GenBank:GASO01058606.1 + GASO01241633.1]

***M***QLRTLPVVVLLFWYARASPENAEYGPELLNPEE***KR***GIPTRDLLRFFLMAFHNQQDGRLNGIPYR…

…GSEFL**G*KR***ALGSEFL**G*KR***ARASEFLGNRALGSEFL**G*KR***ALGSEFL**G*KR***NLGSEFL**G*KR***ALGSEFL**G*KR***ALGSEFL**G*KR***ALGSEFL**G*KR***ALGSEFL**G*KR***GPLGSEFL**G*KR***AMESEVFEDPNVNEVEKPSNPEDNLETNSTQIFMLE***RRKR***SPEEK*

>*Tricholepidion gertschi* (Zygentoma) EFLa-like[GenBank:GASO01250556.1]

***M***QLRTLAVVLLLLWYAGPGSAENELGLLNPEE***KK***EVSIFSLMPLPYKQD**G*R***QAGSIGGDLLGKLSNMESQMHEKQAQGVELFKNLTLGSSSEPTAA***KR***GLGSEFL**GK***

>*Thermobia domestica* (Zygentoma) EFLa [GenBank:GASN01066898.1 + GASN01398433.1 + PCR full-length KT152028]

***M***VDMKSRFFHLFILICLSTGVTPENTEESPEIYDAEE***KR***GLTARELLRFFLMAFHNQPDSGLGMHGIPYRPV***RR***LGSEFL**G*KR***SSTDDHVDD***KR***VMGSELL**G*KR***ALGSEFL**G*KR***ALGSEFL**G*KR***ALGSEFL**G*KR***ALGSEFL**G*KR***ALGSEFL**G*KR***ALGSEFL**G*KR***ALGSEFL**G*KR***ALGSEFL**G*KR***SLGSEFL**G*KR***SDEFD***KR***VMGSEFL**G*KR***ALGSEFL**G*KR***ALGSEFL**G*KR***DSESDYLE***KK***ALGSEFL**G*KR***ALGSEFL**G*KR***ALGSEFL**G*KR***TPEQEENLENHKEVVAEPWTFEGSPEHNSTQILLLERK***KR***FA*

>*Atelura formicaria* (Zygentoma) EFLa [GenBank:GAYJ01318516.1]

…TLVILLCCCSPAVPENTEDVQVSEVAGSEE***KR***GLPTRDLLRFFLMAFHNQQDGGGSNLHGIPYRPV***RR***LGSEFL**G*KR***SYLYHLQGNPIPQSELD***KKR***ALGSEFL**G*KR***ALGSEFL**G*KR***ALGSEFL**G*KR***ALGSEFL**G*KR***ALGSEFL**G*KR***ALGSEFL**G*KR***ALGSEFL**G*KR***ALGSEFL**G*KR***ALGSEFL**G*KR***ALGSEFL**G*KR***GLGSEFL**G*KR***GLGSEFL**G*KR***ALGSEFL**G*KR***QPSPLLHDDSEPEYVE***KR***ALGSEFL**G*KR***AFEPEFIDKTASPSEFS**G*KR***ALGSEFL**G*KR***ALGSEFL**G*KR***VMGSEFL**G*KR***GFEPGTDGVQSSNETDVSWIAGDNPSSNSMHVFTV***RRRKR***SD*

>*Nicoletia phytophila* (Zygentoma) EFLa

***M***VEMLEKLLPLLLLLGCCSVATPENTDDRQAWIEAE***KR***GLPTRDLLRFFMLAFHNPEDAGGRGSGLHGIPYRPV***RR***LGSEFL**G*KR***SDPDYDADYDQEIDPEPS***KR***ALGSEFL**G*KR***ALGSEFL**G*KR***GLGSEFL**G*KR***ALGSEFL**G*KR***AL…

…EFL**G*KR***ALGSEFL**G*KR***ALGSEFL**G*KR***ALGSEFL**G*KR***ALGSEFL**G*KR***ALGSEFL**G*KR***GLGSEFL**G*KR***ALG…

…SEFL**G*KR***ALGSEFL**G*KR***EQDSDLSDESSIDSDLI**G*KR***RLGSEFL**G*KR***ALGSEFL**G*KR***ALGSEFL**G*KR***ALGSEFL**G*KR***ALGSEFL**G*KR***ALGSEFL**G*KR***ALGSEFL**G*KR***DLEQENSEELRNSEGDQPWTFSEVPDNNATHPLTQ*

..............................................................................................

>*Lithobius forficatus* (Chilopoda) EFLa

…AEN***RR***AGPGS***KR***IGSEFM**G*KR***NDDAMD***KR***LGSEFL**G*KR***MGSEFL**G*KR***MGSEFL**G*KR***MGSEFL**G*KR***MGSEFL**G*KR***MGSEFL**G*KR***LGSEFL**G*KR***MGSEFL**G*KR***IGSEFL**G*K***…

>*Hanseniella* sp. (Symphyla) EFLa

***M***RCAIRTALLLILLLAASIQANASSDQSEHSGQQNLRKFSSNGFIHDPYLVYDALR***KR***LR**G*RR***MGSEFI**G*KR***SVEETRFPEFAEVEMPLYEEALDTPLDNIE***KRRR***PGSEFL**G*KR***RMPGSEFL**G*KR***RMP…

…RSENEITIESSLIDRPD***KRRR***PVSEFL**G*KR***RMPGSEFL**G*KR***SENEYMLIGSMD***KRRR***PGSEFL**G*KR***SENEDYLIDGPN***KRRR***PGSEFL**G*KR***EYVFNTLPQSFPAHEIFTVEPEAKLEGEILSNDHDKD***KR***AR…

>*Eudigraphis takakuwai nigricans* (Diplopoda) EFLa

…SEFL**G*KR***MGSEFL**G*KR***SQD***KR***IGSEFI**G*KR***SNDDSVSQE***RRKR***STRQR*

..............................................................................................

No EFLa known from *D. melanogaster*

>*Daphnia pulex* EFLa ([Dappu1_309437](http://genome.jgi-psf.org/cgi-bin/dispGeneModel?db=Dappu1&id=309437), GenBank:[EFX70415.1](http://www.ncbi.nlm.nih.gov/protein/321459361?report=genbank&log$=protalign&blast_rank=1&RID=XWB42D7J014)]

***M***RMEILQHHSACQRMLAVLLLLLSASSGFVPTADEQSPAVENWNDLVLRC***RR***SADDGSDSVTKEGSSLLPIRPV***RR***LGSEFL**G*KR***AAAVVLTLENLCSELLFSEEEVDENWLCQCIRNWEQSTLPASSSLGGQHNMDEDSLAPAAAARVN***KR***GLGAILLS**G*KR***MNRDKWNNNALT***RR***VMGSEFL**G*KR***AIMGSEFL**G*KR***AIMGSEFL**G*KR***GYN**G*R***SNGLSGPVKI*

Eclosion hormone (EH)

>*Nipponentomon nippon* (Protura) EH

***M***GQMRCLIILTAGFLLIANEVHSGFYQSQQRSRDLVTLCITNCGQCKKMFGDYFHGQVCAESCISTQGLSLPDCNNPTTLRRFL***KR***FY*

>*Acerentomon* sp. (Protura) EH [GenBank:GAXE01136170.1]

***M***ATLRPLVLLTTAALVMLLVSESESGMFQRPMDLVTLCITNCGQCKKMFGEYFHGQVCAESCISTQGLSLPDCNNPNTLSRFL***KR***FYRK*

>*Filientomon takanawanum* (Protura) EH1

***M***ACLRSLVLIIAAAIIILFTDQSESGMFQRPMDLTTLCITNCGQCKKMFGDYFHGQVCAESCISTQGLSLPDCNNPTTLSRFL***KR***FYRK*

>*Filientomon takanawanum* (Protura) EH2

***M***LWLSHVQVMLATVLLSMLIHLSTSAISSSNHQLRICLKNCVQCKKMFGYYFEGRACADA…

>*Anurida maritima* (Collembola) EH [GenBank:GAUE01050635.1]

***M***SSYGMRVRILALLVITFFSLFLRDVEGGTLYSARTRSGRIKICLNNCSDCKQLYDEYFHGRHCAEKCIYKKGRFIPDCHDLRSIAEFINKLE*

>*Podura aquatica* (Collembola) EH

…PTCIVNCAQCVKVYGDFFDGHSCAEMCMRAWDEYRVDCADPNTIARFIRP*

>*Folsomia candida* (Collembola) EH1 [GenBank:GASX01081514.1, GAMN01003940.1]

***M***RQKWVSLAVILMVLFAGLLPTLGDASTSTSLSRVKICFKNCGQC***KR***MLGDYFDGRRCADHCISQKGRFIPDCHDIFSISAFLSKLDY*

>*Folsomia candida* (Collembola) EH2 [GenBank:GASX01075476.1, GAMN01023689.1]

***M***TIWILTIVTFVATFAYIGMKPVYASPTCILNCAQCVRMLGDYFDGQRCADYCISQKGRFIPDCDDIFSISAFLSKMD*

>*Bourletiella hortensis* (Collembola) EH1

…FCLLLSVKMGNAMLSATSRKSIKICLKNCGQCQRVYGDYFEGRRCADHCIATKGRSIPDCNDVNSIGTFLSKLE*

>*Bourletiella hortensis* (Collembola) EH2

***M***GQLKVHRMAILIALASILLLSVNPRRATAMHLSCISNCSQCVKIYAEFFDGRQCAEYCIGMRNWSDGHIDCNDPTSISRFIRYT*

>*Orchesella cincta* (Collembola) EH1

***M***NLYRCKSPVVALILAIIILMLSTLSTSSVKARMKICLKNCGQC***KR***IYGDYFEGRRCADSCVIQKGRFIPDCHDDYSISDFISKLE*

>*Orchesella cincta* (Collembola) EH2

…LLVSVVMAITVSALIQPLNANPTCILNCAQCVKIYGDYFDGHTCARVCLDSRSKFDSFVDCADPTTIARFLKTF*

>*Pogonognathellus sp.* (Collembola) EH [GenBank:GATD01013184.1]

**M**NSVLLLKRVTVLGLLCFCVTSLVLLATGSEAAISTVSRTGIKICLKNCGQC***KR***MYGDYFEGRRCGDFCLHQKGRFIPDCHDLYSIGDFISKLE*

>*Sminthurus viridis* (Collembola) EH1 [GenBank:GATZ01100885.1]

***M***NIRVISSLLLLLCLFTWSQAMLSSSSRKSIKICLKNCGQCQRVYGDYFEGRRCADHCIATKGRSIPDCNDVNSIGGFLSKLE*

>*Sminthurus viridis* (Collembola) EH2 [GenBank:GATZ01096961.1]

***M***AFTMYFRLSPTFTVVILGLIVLSSAPRANGMQLSCIANCSQCVKIYAEFFDGRQCAEFCIGMRNWGDGHIDCNDPTSISRFIRYQ*

>*Jordanathrix leptothrix* (Collembola) EH

…FSSTANVNVSGMHLGCIANCNQCVRVYADFFDGRQCAEYCLGMDTLWGEAPIDCNDPTSIAKFIRFQK*

>*Campodea augens* (Diplura) EH1 [GenBank:GAYN01126096.1]

***M***VIFVFLTFFFVIPNSEGKSMVNICVANCGQCQQMFGEFFQGRACAESCLATAGLSVPDCNVPATLKKFL***KR***YKPSSSGVVQALDEYIIQEN*

>*Campodea augens* (Diplura) EH2 [GenBank:GAYN01126882.1]

***M***QWTRILLAALLVIFVTSQSEAAIAVPICLKNCAQCENWYSDYFDGELCAEDCVRLKGKFIPDCADANSIARFLSRVE*

>*Lepidocampa weberi* (Diplura) EH1

***M***SLSPSKRTFVVFSCFLILLATGVESRSMVNICVANCGQCQQMFGEFFQGRACAESCLATAGMSVPDCNVPSTLHKFL***KR***YKTMQQHPNASPPLDDMVIPES*

>*Lepidocampa weberi* (Diplura) EH2

***M***RESMQLKLSLFLLTIIFSLHMSSSNKPAIAVPICLDNCRQCKIMYAEYFDGELCAEACVKLRGKIIPDCADSESIAPFLSRAE*

>*Occasjapyx japonicus* (Diplura) EH1 [GenBank:GAXJ01084469.1]

…NAVGICIANCGQCQQMFGAFFHGKACAEACLATAGLSIPDCNNPATLRTFL***KR***RYNHNF*

>*Catajapyx aquilonaris* (Diplura) EH1

***M***ACSSCVLVFGLLASLQMTVSGANNWERNVVGICIANCGQCQQMFGDFFQGKACAEACLATGGLSVPDCNNPATLRNFI***KR***FKKY*

>*Catajapyx aquilonaris* (Diplura) EH2

…GGGPGADQVWCALPGPVSVCLKNCGQCQTMYGDYFEGPVCAEACLKLRGKLMPDCANATSIAPFLSKME*

>*Pedetontus okajimae* (Archaeognatha) EH

***M***DKRFVLALSLAALLLFLDVTPTTANPVGICIKNCAQCKKMFGPYFEGQLCAEACVKFKGKIIPDCTDVDSIGPFLNKFE*

>*Meinertellus cundinamarcensis* (Archaeognatha) EH1 [GenBank:GAUG01235878.1]

***M***NKLFFSLAVLFTVFIVLDFSLVNASPVGICIKNCAQCKKMFGPYFEGQQCADACIKYKGKLIPDCTNGDSIAPFLNKFE*

>*Meinertellus cundinamarcensis* (Archaeognatha) EH2 [GenBank:GAUG01238390.1]

***M***VNSAVYLLSVVLLSNIAQQEGKVLPDVFVCIRNCGQC***KR***MYGDYFLGQSCAEACYSTAGHSSPDCNNPLTLNRFL***KR***L*

>*Machilontus* sp. (Archaeognatha) EH

***M***GKFFLSMAALFTVFLFLDFSLVSGSPVGICIKNCAQCKKMFGPYFEGQQCADACMKFKGKIIPDCTNGESIAPFLNKFE*

>*Tricholepidion gertschi* (Zygentoma) EH [GenBank:GASO01247720.1]

***M***TGGKAAIFMAILLLVLLMSTMVLSDNGSLGLCIRNCAQCKKMFGPYFEGQLCADACVKFKGKIIPDCTDAASIAPFLNKFE*

>*Thermobia domestica* (Zygentoma) EH1 [GenBank:GASN01387853.1]

***M***VTSCRTRFWIAIVGFLIICSSSAQNNYLSQVGVCITNCGQCKEMFGDYFHGRACAEACLATAGLTSPDCNNPSTLQQFL***KR***YK*

>*Thermobia domestica* (Zygentoma) EH2 [GenBank:GASN01368208.1]

***M***TGGRKNHQHQSLMTSAVLLLLLLSTIAVTDASPVGICIKNCAQCKKMFGPYFEGQLCADACVKFKGKIIPDCTDVGSIAPFLNKFE*

>*Atelura formicaria* (Zygentoma) EH1 [GenBank:GAYJ01301288.1]

***M***STVRFSLQVVTVCVMCVCLCVAAENSLLNQVGVCITNCGQCKEMFGDYFHGQACAEACISTSGLTSPDCNNPSTVMRFL***KR***FK*

>*Atelura formicaria* (Zygentoma) EH2 [GenBank:GAYJ01290973.1]

***M***KGQKSTHVKMAMLLVICLLSSSVLTEANPVGLCIRNCAQCKKMYDGYFEGQQCADFCVKLKGKYIPDCTDAASIGPFLNKFE*

>*Nicoletia phytophila* (Zygentoma) EH1

***M***MVVSGRTTIYLAAAGVLLMCCYNAVADNTFLSQVGVCITNCGQCKEMFGDYFHGQACAESCLATAGLTAPDCNNPSTLLRFL***KR***F*

>*Nicoletia phytophila* (Zygentoma) EH2

***M***AGHRTSQVSMTTLLILACLMSAAVLTDANPVATGLCIRNCAQCKKMYDAYFEGQQCAEFCLKLGGKFIPDCTDANSIAPFLNKFE*

..............................................................................................

>*Xibalbanus tulumensis* (Remipedia) EH [GenBank:JL143836] (Christie 2014)

***M***MGRRAVLYTTGVTMLILILTMTADSSYVGSCIRNCGQCKKMYGDYFHGQACAVSCIETSGMTVPDCNNPSTINRFL***KR***LI*

>*Anaspides tasmaniae* (Malacostraca) EH1

…VVLWTVLVLVLMLSCACRGASITGMCIRNCGQCKEMYGDYFKGHECAQSCIMTLGNSIPDCNNPSTFNRFI***KR***FL*

>*Anaspides tasmaniae* (Malacostraca) EH2

***M***ADSRKVVYSALLVLSVVLCVLPCRSMCAVV***KR***TTMTGNSMVAICLTNCAQCKDQYGDQFDGRACGSYCSRQTSPVIPDCGRSPQALVRSFMKT*

>*Lithobius forficatus* (Chilopoda) EH

RWLRILILSLWLLAAIFICCGKDEVESQSGLYRCIQNCAQCVQLWERGLYKG***KR***CATACLRHRGLRIVDPECSDLRMFNFKPRIIRKLLAAKGTKPVHRHGHGDKET…

>*Hanseniella* sp. (Symphyla) EH1

***M***PGLPLTTFVLVISVCLLVNLCPASARSSLTIICVRNCHQCKLLFEKYFNGELCADMCKYIETNDFQMPDCADGNSISQFLNRFTD*

>*Hanseniella* sp. (Symphyla) EH2

***M***DSVKSLLVLLLLWDLRPTCARSSPTVICLKNCLQCKLMMHRHFNGELCANTCQDIETNNFLMPDRADGNTIIPFLNTFNN…

>*Eudigraphis takakuwai nigricans* (Diplopoda) EH

***M***ISCFPRHNCNQTKARRSSLSLLVWLLVFEWVNTQELVTDSSPLAIRP***KR***TYSFKEALYICLHNCGLCVQFWEYGLYSGRRCALKCIKVYTAHE***KR***GKLPEILSVDPDCEDPRMFNLSPDVIQTLVDIQREEEASESRTTRPLNEVSDEFDELTASVSPVDVELLRSYTT***KR***SHLKSRRRHHRHHD***KR***D*

..............................................................................................

>*Drosophila melanogaster* EH ([FBpp0082885](http://flybase.org/reports/FBpp0082885.html))

***M***NCKPLILCTFVAVAMCLVHFGNALPAISHYTH***KR***FDSMGGIDFVQVCLNNCVQCKTMLGDYFQGQTCALSCLKFKGKAIPDCEDIASIAPFLNALE*

>*Daphnia pulex* EH1 [[Dappu_240158](http://genome.jgi-psf.org/cgi-bin/dispGeneModel?db=Dappu1&tid=240158), incomplete in GenBank:[EFX83453.1](http://www.ncbi.nlm.nih.gov/protein/321472483?report=genbank&log$=prottop&blast_rank=1&RID=XPH4X5H5014)]

***M***NGTISRRTCSTQHQQNGGGMTTQSGQKMSTMAIMTLLIWLAMTVDVAQPADTLYLCMKNCEQCKSMYGAYFEGDLCAKSCFRLKGAFIPDCIDVASIGQFLNKNE*

#### >*Daphnia pulex* EH2 [DappuEHL; [**Dappu1_442999**](http://genome.jgi-psf.org/cgi-bin/dispGeneModel?db=Dappu1&tid=443000)]

***M***AVVVQKSSILLLFFLSVWISFFVVVSSQGSDGSKKPMLGNIHLCMMNCGQCKEMYGEYFEGQRCAEFCLASYKPSQAGSVSGGGGGSGWAPMPDCNEPETVDQFLKLSLMPQSDGPSVQQLIDDSDSDGGGGYMSPISQALMGSAYSGRYAGRKPSASAQQNTLYSSRTGAEFKGHNQLLKNRKWKKRINGSSKLLGGSPSMPRWYFV*

Elevenin

>*Nipponentomon nippon* (Protura) elevenin

MIEVHKDYIKVQKGEVVFLKHKLVAVLNKMNNLVASLTFRRVSSNSDSVSVRSRKSGRPTRIRNSNWYSGGISLWYFLSLTLALIMFMESVQLMEVDCRKFVFAPLCRGVVA***KR***TSDSKETPSLVKAALLLGLQDGIQLDEMIPYMKD***KK***HSINKHKQLQDDRILVSPILMEEYGARSNNINENSEYDE*

>*Acerentomon* sp. (Protura) elevenin [GenBank:GAXE01020869.1]

MICSTRSSKHSSLRIERRPRKPLISFIWMLICLLALLESVHLMEVDCRKFVFAPLCRGVVA***KR***NSQDDLKYPSAILRVLLDGLSELEMTSEMTHRGRKQERHNNNGQDYSQMDPDRPIVLNPILVKYKQQPRDYEDE*

>*Filientomon takanawanum* (Protura) elevenin

***M***VLWTRFAVSGCSGPHRSSRRLLCVLWFLLLLAGSVHLMEVDCRKFVFAPLCRGVVA***KR***GNNRQDKNPSSLIKTLLDGLHQSELDLASMLEKNEQQQNNNNNKQDYSEVVPDRPIVLNPVMIKYNRNDYEDE*

>*Campodea augens* (Diplura) elevenin1 [GenBank:GAYN01013487.1]

***M***SIPQTFQGITVVFALCCIVPLMSESAKTLDCRRFVFAPMCRGVTA***KR***SQELFGNSPLPNADVTSIDDGQILNQINPAVLREALGLIALKNRQRFQGPWTASDFSRSSSDSTGYSEE*

>*Campodea augens* (Diplura) elevenin2 [GenBank:GAYN01013486.1]

***M***SIPQTFQGITVVFALCCIVPLMSESAKTLDCRRFVFAPMCRGVTA***KR***SQELFGNSPLPNADVTALDDGQILNQINPAVLREALGLIALKNRQRFQGPWTSSDFSRSSSDSTGYSEE*

>*Lepidocampa weberi* (Diplura) elevenin

***M***SSNSALETVALVCALCCLLPLATESTHTVDCRRFVFAPMCRGVTA***KR***SQESGAQPIGYPVPANADVSNEQNNGRSAEETIANNRVYAAILREAIGLLNRNHQRQELPWSVNDYSSDASGVSGD*

>*Pedetontus okajimae* (Archaeognatha) elevenin

***M***NRSQHIVWGFFGFALLLSFSTCTESLDCRRFVFAPLCRGVSA***KR***SSPAIPTGDLQMERDLPSLETMLDLYLTSMRA…

>*Machilis hrabei* (Archaeognatha) elevenin [GenBank:GAUM01109798.1]

***M***NRSQHIVWALFAFALLLSFSTCTESLDCRRFVFAPLCRGVSA***KR***S…

>*Meinertellus cundinamarcensis* (Archaeognatha) elevenin [GenBank:GAUG01238118.1]

***M***NRGQHVKWGLLAFTFFLSFSTCTESLDCRRFVFAPLCRGVSA***KR***SSPSVTGDLQSDREMSSLENILNLYLSSLRGNTADNVESSSSSRISPPSSHQNPKMAAILKDMLYQHLDRGDDPNMVTDEAD*

>*Machilontus* sp. (Archaeognatha) elevenin

***M***DRSQQIKWGMFAFTLFLSLSTCTESLDCRRFVFAPMCRGVSA***KR***SSLPAVIGDLPSDSIRDTPSFGNLLNMYLTTLRGNSDDDVQIHSNSRIPISVSSSQQDPKVSAILRQMLYRHLDRGDD…

>*Tricholepidion gertschi* (Zygentoma) elevenin [GenBank:GASO01250104.1]

***M***SGGWRWQSITRIVSLALLVSLIVTSEALDCRKFVFAPMCRGVAA***KR***APPPSRTANLSTDRQMPALDEILGMYMTSQGAEGEPEVESDRTVPRVLAEDQGSKLDRLYKWYLSQ**KR**NVIDEYED*

>*Thermobia domestica* (Zygentoma) elevenin [GenBank:GASN01383033.1]

***M***PRGWRYQALIRSLSLILLVGLFVTSEAIDCRRFVFAPMCRGVAA***KR***AGMGSNLPVDRDVQSLEDLFGLYLDSQGGSPSLPDAGGSRPSSRMWNDDQGSKLETLYKWYLN***KK***QQ…

>*Atelura formicaria* (Zygentoma) elevenin [GenBank:GAYJ01316417.1]

…GYQPAASLALIIALIVTSEAIDCRKFVFAPMCRGVAA***KR***APASSRTATLLTDRQIPPLEDVLGLYLTSQAEDIEPETDSARTPSRSWGKEKGSELESLYKWYLSQ***KR***GDQVVYEEK*

..............................................................................................

>*Xibalbanus tulumensis* (Remipedia) elevenin

***M***NETCARQLIYSSLVVVLLSLLVTTEALDCRRFVFAPMCRGVTA***KR***ASPPSKHSHTWKEYSDLLPLEDLNSAY…

>*Lithobius forficatus* (Chilopoda) elevenin

***M***RKGSSTSSSVILSAVILILLVSHTAAIDCMKYVFAPKCRGISA***KR***GAEPVAKYPSLSDSLDSDYRSQSDYVSDSDSLTDSYGLKVGRMPDERYANTSPAPVVPHDQTNAQASGAQNRYLKALLRMYLDRRAEDYDV*

>*Hanseniella* sp. (Symphyla) elevenin

***M***SSAAVVVSCVLVLCLILPVSSIDCRKFIFAPRCRGISA***KR***AQLEPLYSSSDNSEEGRGHPQVPMGANLVSFETDGDDDRPLKGSSLYQLSPDDSRHIQSSLREKYVNALLQKYWR***KR***ANYGAKNSLKE*

>*Eudigraphis takakuwai nigricans* (Diplopoda) elevenin

***M***SGFPDVRHPPTILLIVILTTLLMATPTYTLDCRKQVFHPHCRGITA***KR***AQYPPVFITDESSLGFRDAILDPLKPPVEEENSDANQDLPNTVYNPSQDEYMRAVFRKYWNHRPDFTNLNL*

..............................................................................................

no elevenin found in *D. melanogaster*

>*Daphnia pulex* elevenin ([Dappu1_303013](http://genome.jgi-psf.org/cgi-bin/dispGeneModel?db=Dappu1&tid=303013), GenBank:[EFX89671.1](http://www.ncbi.nlm.nih.gov/protein/321478714?report=genbank&log$=protalign&blast_rank=1&RID=XPVH2KCW01R))

***M***MRFNSMSSSSTILLLFAGIVIFAAVHVHSRDLDCRRFVFAPMCRGATI***KR***SFIPNIGVNQDDVHELLLDYPKQQVMDAETQPLLIPILIDRRIFEARSAANRHQPQLDAED*

Ecdysis-triggering hormone (ETH)

>*Nipponentomon nippon* (Protura) ETH

***M***VTNLVPFLTILILVLSSFYSSATTQFFMKVPKTVPRI**G*RR***SPSPGGSSGHETEVAYG…

…SALGEPQFYLKSSKVVPRL**G*RR***QLPTWLGVEMALFARQPQLEEMSNRSGSDPDDPDDPEPDSEDKDPPGVRARGTT**G*RR***STTTMIWINQSFSFCPITNYSHINKADQSNNNTYLE*

>*Acerentomon* sp. (Protura) ETH [GenBank:GAXE01025926.1]

***M***AVTTSGPILTMLIFVLSSFYSSATTQFFMKAPKTVPRI**G*R***SGSPPLSESSLEHMTSFGGIGGDSQFYLKSSKSVPRL**G*RR***SHYPTWLGVEMALFSPQDNQLNEDVEQEAPEIPTNKELNRNQQQQELDVASSSSAESDVR***KR***NREQLGNYSR*

>*Filientomon takanawanum* (Protura) ETH

***M***TKSGPILTILILVLSSFYSSATTQFFMKAPKTVPRI**G*R***SGGRAPLVESSSEHMTSHGGTTGDSQFYLKSSKSVPRL**G*RR***SHHPTWLGVEMALFSPQDDQINDQMVFLKQDQGEVESYKNTGTTQETDNRGMSTNLLKSFGSPEVAELRDNYDDMEKGY*

>*Anurida maritima* (Collembola) ETH1 [GenBank:GAUE01051554.1]

***M***GGRFTGLGFLGLSILVILISTGNCQPRNYYKGDKLVRLPPKSLPRI**G*RR***SEMSHLDASGVMRIRDDGNGDILPDKFQIPTIGDNNGLTGLYFLF***KR***VRENNFRPEEPTGIY*

>*Anurida maritima* (Collembola) ETH2 [GenBank:GAUE01010939.1]

***M***TWYLLFLITILGSFQLIESQPQKYLTGNGVRDVFE***RR***DSKNKITSLPSKILPRI**G*RR***SRNSDFGVPVKEPGDEEEDEAGQAGHVELSPIGIP***RR***YMSLSRTPPEPSTEKQKLLLLLKYLLKHGVIQPEDSMVFFGFNGKSD*

>*Folsomia candida* (Collembola) ETH [GenBank:GASX01074734.1]

…ILGGSFPISFCFIVVTMVVIILRLTHAQPQHFFKANAPVSKSLPRI**G*RR***SDLETFPLPLMTDNRPWSEGNVVEHRNGNQQPQEVRGGGGESYAIPFLEEEESRLAPNILKCLDHQQQPSTFSRGIDPPDA…

>*Bourletiella hortensis* (Collembola) ETH

***M***RSHHGKSLPFCWTLVFIMTLTYFHELTQAQPQVYFKASQLPAKSLPRI**G*RR***SDIDPYAGAAANPTDLWNDIFSYDNNNNINSNSYNPRPESVLLWRKWLTGENIFPEDLSLPVGPNGLYKIKDK*

>*Orchesella cincta* (Collembola) ETH

***M***TRVRQQAGLFPLGLPILLITIIPFLLVQDINGQPNYYFKASAPAVKSLPRI**G*RR***SDDGGISDTSFPSNGAGGQNLLSLSKSLESYPPGILELWNNNPSDSFGGNVGSLNRSPLYNLMSSSGVGPSSLIPPIPVSSPSSSATQLEKSVLWRKLMGDINNYDEAALLENLYHSKS*

>*Pogonognathellus sp.* (Collembola) ETH1 [GenBank:GATD01090509.1]

***M***KNRQNRALRFYWLFLVILLSIFFDRSSGKPNLYFPGQANFKSLPRM**G*RR***SGSDSISGRHPFNDLFRSVPSFDKTSPYQLGASEESTESTWNNLIPSNDITSPGNGLILTYNRKPTIWTTFTGIGSNKENPTDNSQEFVLFSNEQ*

>*Pogonognathellus sp.* (Collembola) ETH2 [GenBank:GATD01088352.1]

***M***KSNNKRGGLLRFCWLFLVVLSSLFFRISSAGEPMSGLYLPGIKSIPRI**G*RR***SGAGDFSGRSAKEDPFIYPVL***RR***NLMYEKFGDSTMNLNTLDTFVPVQDIVLSPTTGMGMFASPKFLFWKKFPPASANSNNGFRIPMSFPVSVPVAGADRESWMEDPNEIQRLRERI*

>*Sminthurus viridis* (Collembola) ETH [GenBank:GATZ01100829.1]

***M***KASRGQGLTYCWTLILLLLVFFHEMVLGQPQVYFKASLPAKSLPRI**G*RR***SDLDSFPVNNEGWNDVYNYNGNYVPRPESVLLWKKLMGDNNMYPEDSQPQLTNLLSSSLYKFKAEK*

>*Jordanathrix leptothrix* (Collembola) ETH

***M***FVLLAFFHHLVASQPQVYFKASLPAKSLPRI**G*RR***SGNSDGLSFPSRPPSSASSSSDVFGGGSSALSSGSSDPFNDIFYNSLNLNNNNNYNPHPDAVLLL***RR***FLAEREREQRDSSNNFYPQDLQSLPASSAFYTKYASSQDATK*

>*Lepidocampa weberi* (Diplura) ETH1

***M***ARLSVGILVCAAAVVFVLLADLASGSPQFYLKEKNIPRL**G*RR***SWTPPANKGSSVNRGQFPGDNNSGALGAEDGEAAVVLDPRMPVVIDD…

>*Lepidocampa weberi* (Diplura) ETH2

***M***ARLSLGIYFIASVLLFAFSVDLGSASPQFYLKDKNIPRL**G*RR***SW…

>*Occasjapyx japonicus* (Diplura) ETH 1 [GenBank:GAXJ01106413.1]

***M***GFTGTNGSACKWYLLAVMLAMILMAAGDSSQFFLKTSKSVPRM**G*RR***GDPNAQEAMSPFFLKTSKSVPRL**G*RR***GQPEWPPRQDAQEAQGGVPSDDYAPAISAIQDIPPALRQKLWLVMATAAPEDNNSNNNNKGLWWMG*

>*Occasjapyx japonicus* (Diplura) ETH2 [GenBank:GAXJ01042873.1]

***M***QPFLVQHLRATSRALLLLSTLCMVLGLSLADADSGTFFLKATKNVPRI**G*RR***SGGGDNTFFLKA…

>*Catajapyx aquilonaris* (Diplura) ETH

***M***SLSGYKNSVCCKWYVTLVMVSMALMVAGDNSQFFLKTSKSIPRM**G*RR***GDPNAQEPVSPFFLKTSKSIPRL**G*RR***GQPEWPLNQDSQEGQAPSEELVLTSMQDIPPAIRDRLLTLLSNTEDNNNNSNNGNNRGIWWMG*

>*Pedetontus okajimae* (Archaeognatha) ETH

***M***CQQIWIAKITGGFLLVLTMQAAVIADTDGSSSSSGTQFFLKSKTIPRL**G*RR***SESDDMDAQSYASPDGFFFLKEEKIVPRI**G*RR***RNLPAWAGLRSASSWDYEALPWYRAGSLGAEEDPSLARIVQYVAPRH***RR***IDPDVNPAPLMEIGGDDNVLSRSEIKWLENFAGGLPSPIGIPRWWRSAPHEEQPVEEIPAIYM***RR****

>*Machilis hrabei* (Archaeognatha) ETH [GenBank:GAUM01181769.1]

…VVTADETSPSGTQFFLKSKTIPRL**G*RR***SESDSKPPQEGFFFLKEEKIVPRI**G*RR***MNLPAWAGLRSASAWDSEALPWYRAGSLGAEEDPTLARFVQYVAPRY***RR***IDPAVGSIPGVENENVIDETVLSRSEIKWLENFAANFAGAQPTPVVIPRLLHSTHGQQSLEEAPAIYM***RR****

>*Meinertellus cundinamarcensis* (Archaeognatha) ETH [GenBank:GAUG01022965.1]

***M***TMECNNRLGSLVTLFTGVIIMMLMFGETVVIATDTGSSTGTQFFLKASKSVPRI**G*RR***SESDDAGSQGYAAPDGFFFLKAEKNVPRL**G*RR***RNLPAWAGLRSGNEWDYEAWPWYRSGTSEVNGSPSLAKLVQLSMPRY***RR***VDSGIPILAADNNDIDDDALYRSEIQWLENFAGSQPGAARSWRSIRPLDTTMINEETVDESPAVNNM***RR****

>*Machilontus* sp. (Archaeognatha) ETH

…YSSPDGFFFLKAEKNVPRL**G*RR***RNLPAWAGLHSASDWDYEAWPWYRTGSSEAGANPSLARLVQYSMPRY***RR***IDSGIPIVAVEENVDSNVDNNGLDDNALYRSEIQWLENFAGASRGPLRAWRSADGDVTLEETPAVIM***RR***R*

>*Thermobia domestica* (Zygentoma) ETH [GenBank:GASN01371266.1]

…KSVPRI**G*RR***SDPDNYFLKASKSVPRM**G*RR***RTLPPWVLSESADWDSWPWFRAENWGSAPDPGPGALKEGPVGFIRPQRAEQDIPKLETDKDGNVVWWNSNSDEMATLNTALWKNGMTPFE***RR***ERAPYNILFVEQE…

>*Nicoletia phytophila* (Zygentoma) ETH

***M***SVSFGKNSLASSVAFLLLIAATMTLADNENFLKSSKNVPRI**G*RR***SDDDRPFLKATKTIPRI**G*RR***RDVPPWTDKVPEEGEDWDSWPWFRGVNWGAATEPGPGGVRQSAAGFVRPQRTAPPQQLEGLYQLDKVGWQGGNEDLKYNYLANLDKYYRELQRSLDLQ*

..............................................................................................

>*Xibalbanus tulumensis* (Remipedia) ETH

***M***RRLQLAAVVLFFVTLLHAGFARQFFTKASKSVPRM**G*RR***SSLLQENLPLLHNTSKFGLLEGHDDVQ…

>*Lithobius forficatus* (Chilopoda) ETH

**M**KTLCGRNSSASFYLFALWLWLVSMASLLPTSNAQFFAKTSKNLPRI**G*RR***GSQDQEMTEVIPSSVKALLAFVRKFDEDASGCLSPEELAAIPVFRLAIDYEDFTPFEIASDVIEEYKVEEDIKLKEIIERLLATAYSQQ*

>*Hanseniella* sp. (Symphyla) ETH

***M***WIGFSGSSLVAPILTVLLLVQILVIVPSEAQFFVKTSKSVPRV**G*RR***TDPQLPPLSERIPDVMRTMLTFIRKYDRDGNGCLSPEELMGVALFRQAAENSDFSQLEYTPYDADDYDGQVSLQQQNPF***KR***FISSLF***RR***KA*

>Eudigraphis ETH

***M***GVWGRLLVLGGLALAFVPAGDAQFFTKTSKSIPRL**G*RR***SGAPTPKPWPARPGDLMGD…

..............................................................................................

*Drosophila melanogaster* ETH [[FBpp0072331](http://flybase.org/reports/FBpp0072331.html)]

***M***RIITVLSVSLLVGLVAISQADDSSPGFFLKITKNVPRL**G*KR***GENFAIKNLKTIPRI**G*R***SEHSSVTPLLAWLWDLETSPS***KR***RLPAGESPAKEQELNVVQPVNSNTLLELLDNNAIPSEQVKFVHWKDFDRALQADADLYSKVIQL**G*RR***PDQHLKQTLSFGSFVPIFGDEQNPDFMMYKNNEDQELYGGGNRYDRQFLKYNIL*

>*Daphnia pulex* ETH [[Dappu1_325362](http://genome.jgi-psf.org/cgi-bin/dispGeneModel?db=Dappu1&id=325362), GenBank:[EFX73380.1](http://www.ncbi.nlm.nih.gov/protein/321462356?report=genbank&log$=protalign&blast_rank=1&RID=XPW4D2E101R)]

***M***YRELIMIKGLFLTWLLLASALSDPSPEPFNPNYNRFRQKIPRI**G*RR***GEGIIAEYMNSESFPHEGSLSNFFLKASKAVPRL**G*RR***KDISTESGRAAMVGEEPFGRISNEIPIMNQKQDLWPNMNINELTGALNKELNYPGPRIPKDLQDNYIQDLIHSWINQYENLNEN*

Extended FMRFamide (FMRFa)

>*Nipponentomon nippon* (Protura) FMRFa1

***M***MQSSLVVIGCLLMTSHGVLSSHQSEEQRDLVYGSGEIQPSSSEYRMERSHPESQSVLHLPGH***KK***DARGGEGGEQYSNGGSSQQSFLHGLLA***RR***SDLDKNFIRF**G*R***AGLNLASPVRAVHPMDFARL***RR***QNTNFIRF**G*R***AGPSIKENNSEFRN*

>*Nipponentomon nippon* (Protura) FMRFa2

***M***WTQALLSFGCLLMTSQCRPSQSEEREIFTPILYDRSADSEPMTSSEYRNQQQRIQRIRDTVNDDNIGVHLALE***KR***NTRVLNDMGEAYTSGVGAAQENFLHGLLS***RR***SALDNNFIRF**G*R***SGSASGFPRL***RR***QNTNFIRF**G*R***SGSQPQKG…

>*Acerentomon* sp. (Protura) FMRFa1 [GenBank:GAXE01133498.1]

***M***ASMLLQTWITVIGCLLMTTLAYCCVHQSEERMDPINSNEFQISSSEYQLERNRNEDDRSRFSQN***KR***SPTGEGGEVFSHGGSHKSHLHGLLA***RR***SDLDKNFIRF**G*R***SGFQSSRLKSMSSQLGDFPRHSRQDTNFIRF**G*R***SGS*

>*Acerentomon* sp. (Protura) FMRFa2 [GenBank:GAXE01021018.1]

***M***CRRTLPLVIGCVLMASSVVSSNQQSDDGVRGQMNSNEIRTSSGEYRMERNRHDDQFLSPVL***KR***GDGEEGGEQYATGGSAQQSFLHGLLA***RR***SDLDKNFIRF**G*R***AGFEPSQLRALSSALSTGKFPRL***RR***QNTNFIRF**G*R***SGT*

>*Filientomon takanawanum* (Protura) FMRFa

***M***LKSSFVVIGCLLMTSHQASPSHQSEEQRERDLVYGSNDIQPSSSEYRVERNHPEPHSVIHMPGH***KK***DGSHDGGELYSNGGSAHQAFLHGLLA***RR***SDLDKNFIRF**G*R***AGLELASPVRAASPMDFARL***RR***QNTNFIRF**G*R***AGPSIKESNSEFRN*

>*Anurida maritima* (Collembola) FMRFa [GenBank:GAUE01036845.1]

…GSFGNS***RR***MSDPNFLRF**G*R***SPSDLENNYSNMFE***KK***SANGVSANFLRF**G*K***SGSDPNFLRF**G*KR***TSSIFGNGAESLEPNFLRF**G*RR***VLP…

>*Tetrodontophora bielanensis* (Collembola) FMRFa [GenBank:GAXI01122451.1]

…EMLKYLKPITSKSIIENPIKWYLSSNFNNMFMSGGGGDGSFPRHT***R***PDSNFIRF**G*R***SHNTGGGHGGGN***KK***NNFIRL**G*R***ENPKQNNFIRL**G*R***SSSSLSSSPTSLTTSDI…

>*Podura aquatica* (Collembola) FMRFa

***M***KMSDGCSLLLFFTFFSMSLSPIFSLPSFPPWGGGTDIDPDLSPKDAPFWALGLNNERDSIPSWANSL**G*R***QIRADNSIPF**G*R***QEQVSQGTQGEKSTNN***KK***NNFIRL**G*R***ENPRQNNFIRL**G*R***GRIEEAARILSNS**G***

>*Folsomia candida* (Collembola) FMRFa [GenBank:GASX01021052.1]

…IRL**G*R***ENPRRNNFIRL**G*R***SGLWANNERFGSKIGYFPDYYSQLFGGNPNIWDFAAQQPIPFDPSDVLQG*

>*Bourletiella hortensis* (Collembola) FMRFa

***M***QMRQRILTSFISLLVLLLPAYGYHIEGKSSSNSNKDQY***KR***QVRAAEAPPIQPQQDQSGQDLVADPDE**G*R***SRSNQLFSLYPSYLVNRYYLPHGSLPGYPTALSGAKWLSGFPMDLPRA**G*R***ADSNFIRF**G*R***STYPGTFRAGS***KK***NNFIRL**G*R***ENPRQNNFIRL**G*R***SGSSWDNYDRVLPKVGFFPDYYYGSAASPSSGYHWSRFTDDVTDDTTN*

>*Orchesella cincta* (Collembola) FMRFa

***M***YWSEAIMSNVTFLMLLLILMSSSQVSTSQPLAKQQDNTIPSLRLLSPSSSNREVSSSLPLPQQSSSSSLGSPLMKSRWVRDIHEGVSEDELPLFNQDYSLASPQHQQQSPPQSSTSKAKGIYQPLTGSGELFRLFRPLALSPEKTIGMTKWYLPGFGYFSRG**G*R***ASSNFIRF**G*R***SGNSRFVEEEDENTPFNR…

>*Pogonognathellus sp.* (Collembola) FMRFa [GenBank:GATD01084722.1]

…DNSNGEHNGQSPATPAFNNYPN***KK***NNFIRL**G*R***ENPRQNNFIRL**G*R***SGYWDSRERPGVGFFPDYVYTPYASINSPMWYPKEENSELARLYETSQMSPQV*

>*Sminthurus viridis* (Collembola) FMRFa [GenBank:GATZ01103412.1]

***M***HLTETIVISLASCLLVVLLPVDAYNVDSKPSHETLNRHVRAIEAASTQPQQQQPEQQDQQSVY**G*R***SGELDFLDEPLKVSGDQRFARLPSPFPMEKSRYFFPSHDLLT**G*R***FGGKWLSGSVYPRI**G*K***ADSNFIRF**G*R***SFPGGFGGNS***KK***NNFIRL**G*R***ENPRQTNFIRL**G*R***SGSLDSSDRFVPKIGFFPEYYSVGSHLSRSKDLSVNQDVPSYDYN*

>*Jordanathrix leptothrix* (Collembola) FMRFa

…TKSTG**G*R***WMLGHVGGGYPRTARADNFIRF**G*R***SFTSGYGSNM***KK***NNFIRL**G*R***ENPRQNNFIRL**G*R***SGPWDSSEKYHSKIGYVP…

>*Campodea augens* (Diplura) FMRFa1 [GenBank:GAYN01138863.1]

…RNFMRF**G*R***SGEVNRNFMRF**G*RR***LQDNFIRF**G*K***ALRQPIATIKIGSKSDSRK***KR***SIDLEDTDSEDSSVTYVPPSMYYADRLRNVRPFYVPIETNFEPPESGSVYAAERWAPEQYSAPTKFTSYFGGKPYIRPE***KK***TWGNIDNNFIRF**G***

>*Campodea augens* (Diplura) FMRFa2 [GenBank:GAYN01061345.1]

…RF**G*RR***LQDNFIRF**G*K***ALRQPIATIKIGSKSDSRK***KR***SIDLEDTDSEDVTYVPPSMYFA DRLRNVRPFYVPIETNFE…

>*Lepidocampa weberi* (Diplura) FMRFa

***M***GQSNKTLLVIIWWYYSLSALSKGEETNIESLKIHPELSGDFQVD***KK***GRSKEEWVPYIY**G*R***HIDENEESVLGH***KR***SADPNYYQAILS***RR***SAMDRNFMRF**G*R***SGGMGRNFMRF**G*RR***LQDNFIRF**G*K***SPHQPIATVKIGPKNEE***RRKR***SVFDEEDSAMSPFPDTVPYISPSMFFNDRIQNFQPFYVPLDSNYENSRSSIYERWAPDQYSAPTKITSFIASLSKPYIRPE***KK***TWGNIDNNFIRF**G***

>*Occasjapyx japonicus* (Diplura) FMRFa [GenBank:GAXJ01112289.1]

***M***KSGGLRLVLLPVLIWMLAEVSCSPVEPSRLGSDPSNDADDA**G*R***ASRSVKVAPRSQLEQSFLRF**G*K***SQPDFDWPQAPSG***KR***VPEEMLDDGSDDPEPRSRVAVPGNNNSP***KR***TKDDYYRAILS***RR***SDLDKNFMRF**G*R***AQGMDRNFLRF**G*R***TNSQHLFFPGAPKFPTLKFHSPQSG**G*R***SD***RR***R***KR***SADDYDETEVEEEAEEPSVPSLLSESERLRHVRPFFVIDPTMVTGVSKFFPDGLWRVPPIMR**G*R***FTTYFPVNSYPA***RR***AMS***KR***TWGNIDNNFIRF**G***

>*Catajapyx aquilonaris* (Diplura) FMRFa

***M***KCSRVVLVLPLLIWLLSGVSCNPVGSSSSPTGSADEEA**G*R***ESRSIQVAPRSQLEQSFLRF**G*K***SHPDFDWSHPSAG***KR***APEEMLDDDDDEEVES**G*R***LGVPPSQKPP***KR***TKADYFRAILS***RR***SDLDKNFMRF**G*R***AQGMDRNFLRF**G*R***TFFRNEVSQKPGSRQ***KR***SVVTTLDDWDEQEQDEEEEEPSVPSLLSETEHRHVRPFFVIDPTMVSGVSKFYSDGLWRLPPIVR**G*R***FTSYFPLGNIYP***RR***TGAVT***KR***TWGNIDNNFIRF**G***

>*Pedetontus okajimae* (Archaeognatha) FMRFa

***M***ALLLRAWLLWLVMLLAAAVVNATFEDAKEYDAYE***KK***DDNDLTDEVVVDDSMEDGGTSAQLA***RR***TSLDKNFLRF**G*R***GPDSTPQHLRLARTQDTNFMRF**G*R***AGLNTNFMRF**G*R***GSDKNFMRF**G*R****AGSHEILKT***G*RR***LDKNFMRF**G*R***NRLENNFLRF**G*R***SDKVKELD*

>*Machilis hrabei* (Archaeognatha) FMRFa [GenBank:GAUM01118713.1]

…EFLRP**G*R***NLDKNFMRF**G*R***NRLENNFLRF**G*R***SDKGISSSKVVLKDLE*

>*Meinertellus cundinamarcensis* (Archaeognatha) FMRFa [GenBank:GAUG01029219.1]

***M***LNRIHQQ***M***AAVSQLWLVWLVLLVIAVAHSLPFEPKDYDALE***KR***SGPDASEEVAEDEEEANNEDAVRSAYMS***RR***SALDKNFLRF**G*R***APPDNDFVRLARGPDTNFMRF**G*R***GPDTNFLRF**G*R***GNDSFIRY**G*R***SNNGYMRF**G*R***GLETNFMRF**G*R***NRLEDNFLRF**G*R***NDKSHLHNNAALKDLD*

>*Machilontus* sp. (Archaeognatha) FMRFa

***M***AVVFRVWLLWLVLLFVSTVHCLPSADSKSYDSPVEERDDDVEGTDADLVVDDVDPRSVEDATRMAYLT***RR***SALDKNFLRF**G*R***GPPPDSSSAFDRL**G*R***GQDTNFIRY**G*R***GSDSNFIRL**G*R***GQDTNFMRF**G*R***ADNKGFLRF**G*R***GLETNFMRF**G*R***NRLEDNFLRF**G*R***SDKSHPGVTKSQLKDLE*

>*Tricholepidion gertschi* (Zygentoma) FMRFa1 [GenBank:GASO01254297.1]

***M***MLLILLLTIVSCLKPFCIADQVLHPIH***KR***AALNQGFLQDLDNMVDPGMVTPEEEEANDI***KKR***SDGSMYKAMALLS***RR***SDLDKNFMRF**G*R***SPSTNFMRF**G*R***GEAGSGVPRQGQ**G*R***DSNFIRF**G*R***NTNSNFMRF**G*R***IPSTNFMRF**G*R***QLDNNFMRF**G*R***QLQDNNFMRF**G*R***DVSHSYATPMTEEGIEQESSHA***RRKR***SIIEEETPGYLLRSVDGEQIAVPFMMQGTSQFFQLPDEETEPEVRSR**G*KR***MDPGQHENNHIRF**G***

>*Tricholepidion gertschi* (Zygentoma) FMRFa2 [GenBank:GASO01215899.1]

…EIE***KR***LNKEFYQRILENRSPLDKGGIRY**G*R***SAGGSGYTRQGN**G*R***ASNYIRF**G*R***NSNFMRF**G*R***SPSNNLLRL**G*R***QLDNNFIPFVRPQQDTNFMRFS***R***DLSNSYTMPVAEEGSLQESSHDVT…

>*Thermobia domestica* (Zygentoma) FMRFa [GenBank:GASN01032603.1 + GASN01036915.1 + PCR full-length KT152026]

***M***IGRHLLLAILCCTIFTSSCHPVQQSLSNDELSTGHQVSCGT***RR***DTNTPAGIAQEIRSAEPESKEEHEKEDKTTSHGVT***KR***ADNDVYRAMALLN***RR***SALDKNFMRF**G*R***SGNSNFMRF**G*R***SGNSNFMRF**G*R***SGKSNLMRF**G*R***SGSSNFMRF**G*R***GNGSNFLRF**G*R***DPADDTGVSHNSEEVKVNDNDMLRDPREDNTNFMRF**G*R***PNSNQNFMRF**G*R***ADPASKFMRF**G*K***SQSSNFMRF**G*R***GGKVSNFMRL**G*R***DSYPNNLLHSNDQIQDKYSYSHGIHETPNILTTEDLSEEASKSRI***KR***SPLDVSDEQDVKAIFAVPEDEFEEDDAPSSGSDEIESEPLIFPAQTPKFFILPEATISKPS***KR***NHLVTDNNNFIRF**G***

>*Atelura formicaria* (Zygentoma) FMRFa [GenBank:GAYJ01009430.1 + GAYJ01148448.1 + GAYJ01315371.1]

***M***IRAPVILVVVCCWLAAASLSASQTSELSRDAAETAEDHKSEEPLQQELVGEESIDGEGDWDSNSDGDGSNEAHSLPI***KR***DSSNVYRAIALL***RR***RSTADKNFMRF**G*R***SGGGSSGNTATEAQENAQTTQPGISI***RR***NDNFMRF**G*R***GAEDNFIRF**G*R***GSADNFMRF**G*R***GSADNFMRF**G*R***…

…NDKNFIRF**G*R***GSAADNFMRF**G*R***GSADNFMRF**G*R***NNATDYMRLVRDSANDNFMRF**G*R***GSADNFMRF**G*R***GPNDNFMRF**G*R***…

…NDNFMRF**G*R***GSADNFMRF**G*R***GSADNFMRF**G*R***GSADNFMRF**G*R***PLNDNYIRL**G*R***SVPDSNTDDVGAEIMSRYHLEKNLQDAENKSAYHTRE***KR***SVTDDYDDDPEKPSFEPEDYVMKTQYLLQHPEEEKEIGPVSLDTSLSSTNVHDPPPMSVIPRYVLVPTDDFHPYGAHT***KR***NRVKDNNYIRL**G***

>*Nicoletia phytophila* (Zygentoma) FMRFa

***M***LHLVLFLAMAACCSVASLSLQENAALSPVQDNQKPEDSFHSQPGDTSAEETSQEEWNHEDDTERGGDVDEVPS***KR***SNDIYHAMALLN***RR***SVLDKNFMRL**G*R***SPSTNFMRL**G*R***SPSTNFMRL**G*R***SPNTNFMRL**G*R***SPNTNFMRL**G*R***SPNTNFMRL**G*R***SRNTNFMRL**G*R***GQNSNFIRL**G*R***DPSSETYSTEAADNKNPTDNRFPRSQSSNFMRF**G*R***GSSDFIRF**G*R***GLNDNFMRF**G*R***SQNDKNGNFIRF**G*R***GLNDNFMRF**G*R***QMGSNFIRL**G*R***DVQEDKMDEVIDDNHPNAEKEVKSVRT***KR***SPEIEENEVPDGISYDPEPEEKMDDNEKLVEEPFVLGISPSGSNLRLLPVSSSIPVAVAVPTSQFPLYMEHS***KR***SNSKENNNNYIRF**G***

..............................................................................................

>*Xibalbanus tulumensis* (Remipedia) FMRFa

…SVSEPNKQETRDIGLEINPDETETNIATSGVSDGSPKV***KR***QYDTYKEILS***RR***SDLDKNFIRF**G*KR***SLGSNFIRF**G*KR***ALDSNFLRF**G*KK***SLGDNFLRF**G*KK***DSDGSF…

>*Lithobius forficatus* (Chilopoda) FMRFa

…RNFLRF**G*R***DPTNRDSLAEA***KR***ATLDRNFLRF**G*KR***WPSNEDDNSDDFSSED***KK***YQIARNLVSALHQKREVEEHHQNSAKFDEDNAEDDDIETVDQEQ***KR***GALDRNFLRF**G*R***QQFDFSASNRNHYMADSS***KR***AALDRNFLRF**G*KR***NEDDYRHVATMNAADNHHDEDSNV…

…MRELSR***KR***GALERNFLRF**G*R***SGEEQEEHHQVE***KR***AALDRNFLRF**G*RR***DGYD***KK***AALDRNFLRF**G*R***NLHHSLDGDGD…

…VRSLCTQPTSLGGLTNDSPYSIICSQDSSDEVSLSSEEVNTLAQVLSE**G*R***YDDPSGDGPEDDSDNAFLRNLRMEGHKFLRF**G*R***TPPDHKFMRF**G*R***DPNENLLRFAREPDHKFMRF**G*R***TPPDHKFMRF**G*R***QPDHKFMRF**G*R***ETADRNFMRNQREPQHNFMRF**G*R***SVNTGEENGFIPFMKFNSPENSFMRAARQPDHKFIRF**G*R***QPDHKFMRF**G*R***NNETPAANSNNNMSSELLDSNNNKNSNTYQETSHDKQTP*

>*Hanseniella* sp. (Symphyla) FMRFa

***M***SEVYLAVIVLVVVFCLPQSISSSHSDEITKILLQEAEKSGILEPEDIDRPRTSAGGGGLPAFS***R***YSREPQHNFLRF**G*R***APEHSFIRF**G*R***SKGASVPNFDETEKDSSRKIR…

…LAFNRFNREPQHNFVRF**G*R***APEHSFIRF**G*R***SRSNSGILTPSDGTRNIRGGAQGVNHNFMRF**G*R***NPEHNFMRF**G*R***NPEHNFMRF**G*R***PDHQFMRF**G*R***PDHQFMRF**G*KR***PDHQFMRF**G*R***P…

>*Eudigraphis takakuwai nigricans* (Diplopoda) FMRFa

…PGHNFMRF**G*R***DPGHNFMRF**G*R***EPGHNFMRF**G*R***DPSHNFMRF**G*R***DPGHNFMRF**G*R***VAAMGHNFMRF**G*R***SPSHNFMRF**G*R***SKEDESDNQTTLEVTDA…

…SPALDRNFLRF**G*R***APALERNFLRF**G*R***APALDRNFIRF**G*R***SKLSNNFIRF**G*R***GDDDETEDDDEDQAMA***KR***GGSLNSNFIRF**G*R***DTSRVTGDNRTEIDRDNPSEDHYLV***KR***AVASDWYLYGGGYPRMSSRLGSY***KR***GSLHRNFIRF**G*KR***SGGDQETRV***RR***EVHGTPPRVGHHSHHPPGIHVE***RR***SAETQDEWNQDVKANFLRF**G***

..............................................................................................

>*Drosophila melanogaster* FMRFa [[FBpp0087471](http://flybase.org/reports/FBpp0087471.html)]

***M***GIALMFLLALYQMQSAIHSEIIDTPNYAGNSLQDADSEVSPPQDNDLVDALLGNDQTERAELEFRHPISVIGIDYSKNAVVLHFQKHGRKPRYKYDPELEA***KRR***SVQDNFMHF**G*KR***QAEQLPPEGSYAESDELEGMA***KR***AAMDRY**G*R***DPKQDFMRF**G*R***DPKQDFMRF**G*R***DPKQDFMRF**G*R***DPKQDFMRF**G*R***DPKQDFMRF**G*R***TPAEDFMRF**G*R***TPAEDFMRF**G*R***SDNFMRF**G*R***SPHEEL***R***SPKQDFMRF**G*R***PDNFMRF**G*R***SAPQDFVRS**G*K***MDSNFIRF**G*K***SLKPAAPESKPVKSNQGNPGERSPVDKAMTELFKKQELQDQQVKNGAQATTTQDGSVEQDQFFGQ*

# >*Daphnia pulex* FMRFa [[**Dappu1_115117**](http://genome.jgi.doe.gov/cgi-bin/dispGeneModel?db=Dappu1&id=115117), GenBank:[**EFX67846.1**](http://www.ncbi.nlm.nih.gov/protein/321456746?report=genbank&log$=prottop&blast_rank=1&RID=XSUNM5ZS015)]

***M***NGLRFLMLILGLMMIVGQVRPDDETSEEDVDDENFLSGSNAAAAAGSSDSAEEDDDNNNNKGGIELYLSAMKDLYRQS***KR***HEAEPLVSSRSGASAPIAALY***RR***SALNKNFIRF**G*R***SGGGVMKTDVSRQMQPIRLMGLNESDS**G*K***DEERSFHPARPSRSLRSNFIRF**G*R***SFFPTSNRWGETV***RR***SA***RR***TPSAGRQMMSLVDLPASSSSSS***RR****

Inotocin

>*Nipponentomon nippon* (Protura) inotocin

***M***LSFVSLLVLVFLFVSGCDSAPCFITNCPPG**G*KR***APLPRYQI***KR***GVPPGWSGHEIRAARSSQENTLDNLMNHLMAVNRQPVLFPQLQ*

>*Acerentomon* sp. (Protura) inotocin [GenBank:GAXE01123413.1]

***M***VYPNTLFLLVFLLVWVPLIRSKPCFITNCPPG**G*KR***SHHNEHAQNHNRLKQRFHYDPQVMEDHPLSARIHRTIDLLNKISSKLLNDGLHEAGGTQHGVR*

>*Filientomon takanawanum* (Protura) inotocin-like

***M***IRCVLLLVFLLLLLDPPCRAKPCFITNCPPG**G*KR***AIRPIVRFIRTRKQNLHTLPRSHSEPPHKGRTPLTPGHHIDLLNKVTSRTLHAGHRMGQRSS*

>*Anurida maritima* (Collembola) inotocin [GenBank:GAUE01051410.1]

***M***RKYHTSIICLVWLISVSAAFGCFITNCPPG**G*KR***SGPLSTQARQCESCGPEGSGGRCYGPNICCSPGSGCLIGTPETVACRSEARFSTPCLNPGISCSGSGSLSIKGQCTGDGICCSSETCTHDENCWERSMTSGTGDSQEFNQDLPLREDRFLIPLPATHEKFRSFPISQIIRV**G*RR***KNQFEDLRNHLLHRPQMDFQPPISSTESLYP*

>*Tetrodontophora bielanensis* (Collembola) inotocin [GenBank:GAXI01072001.1]

…IPEQHNTNKVRECASCGPVGQGGRCYGPETCCSPNFGCMVGTPETHTCLIEDRINSVCHNPGIPCTVNVDNGNINGFCATG…

>*Podura aquatica* (Collembola) inotocin

***M***SSWNNSQIYFILLNLLTLITLNSACFITNCPPG**G*KR***SVGFHNVQRECESCGPLGSGGKCYGPNICCSPSLGCLFATPETHPCLSENIFRTPCTNPGNSCTGLHSNIRNGKCAAPGICCSSESCSLDENCFDVRNEVKNWTQ**G*RR***GFYKN***KK***LTSSGRQLSNEAFSPPEFDA…

>*Folsomia candida* (Collembola) inotocin [GenBank:GASX01087107.1]

***M***KTSLLFAFGIVMVESLLVSGCFITNCPPG**G*KR***SGADTKIRQCEQCGPPGMNGLCYGPHICCSSELGCLFGTSETLTCFQENLMYNTPCQNPGESCSGNDATNPINGQCATLGVCCSSDTCTIDDNCHKKDEQQNVSGHSRSTVAMDGSHTKEHATTMFQNPLDRWIP***RR***FGLGRPTGQRKHVRIGVKPDAQILAPENSLMDRLVIFPDTSGRISSDEY*

>*Bourletiella hortensis* (Collembola) inotocin

***M***VNYNYEICLFVVTLTAVSGCFITNCPPG**G*KR***SGIEPKIRQCEQCGPPGSGGRCYGPSTCCSPELGCVMGTPETIPCQFENRFTTPCQNPGASCSGPNGDLNNAQCGAEGICCNSDTCVMDDTCHDEAAGKSSAGVVHHHSQHGPSSFPLDRVFQRFLD**G*RR***HRLPARLPLPTQSSAASIGNDY*

>*Orchesella cincta* (Collembola) inotocin

***M***SLLHVVQFLILSLVCVSGCFITNCPPG**G*KR***SESEEKTHQ…

…STACKQETHSPTLCHNQAISCDGTGETGPINGQCAAVGVCCTPETCTLDDDCVNDHLVTVTDGRKGNH**G*RR***IPLMKHLDETENEVPLVHGPIDAWFYRRLIHSKAKLVDSRNPSELEQAIMPQGPPYFPAGSPRLADYF*

>*Pogonognathellus sp.* (Collembola) inotocin [GenBank:GATD01083368.1]

***M***IQFVTYNLWLGVLLVSFVAASGCFITNCPPG**G*KR***SGTTALTVRQCERCGPPGMMGRCFGPQICCSPEFGCFFSTAETFVCQLENRFPTPCHNPGSSCSGNGDTGLINGQCG…

>*Sminthurus viridis* (Collembola) inotocin ( GATZ01101765.1]

***M***INLNCGICLFLITLSVVTGCFITNCPPG**G*KR***SGIERKTRQCEQCGPPGAGGRCYGPSICCSPELGCLIATPETYSCQLENRHTTLCQNPGATCAGPNGDISGGQCGAEGICCNSETCTMDETCHDEASVTRESVAKLSGLYHHRYPLDVVFQRFVDGQHSQHHRLPARLPLPTQSSSSSNDY*

>*Jordanathrix leptothrix* (Collembola) inotocin

…AVKDESVVKTGSGLYRHVYPLDIVLQHLLNGPHSPSQQLAARLPLPTQSQSPSSRNNNNDDY*

>*Campodea augens* (Diplura) inotocin [GenBank:GAYN01126761.1]

…GRCFSPDLCCGPEIGCYFRTKESSVCKSENLFPVPCENPAKSCGTDRNGKCAGQGLCCTEDSCTVDSSCHVILKDLPPPPPAAVAPPSPWLKYPSVFRVLQQQQQPNTDLSSATGALWDDMLEN…

>*Lepidocampa weberi* (Diplura) inotocin

***M***LPYCKHHLVAILFVLFVGESFCCFITNCPPG**G*KR***SQGNVKAQGHRQCLSCGPSGSGRCYGPELCCGPEIGCYFRSKESAPCRAENLFPVPCENHAKPCGQERSGKCAGPQLCCTEDSCTIDSTCHVISKDPFPSLLHYPGFRFTSDTNPPAGPSWEDMADNLQQPAGFIIEA*

>*Occasjapyx japonicus* (Diplura) inotocin [GenBank:GAXJ01105745.1]

***M***SSPVRIHPRLLALACVFCISSACFITNCPPG**G*KR***SQQAHYKPQASRQCAVCGPNGQGRCFGPELCCGPEIGCYMRTRESSSCRAENLFPVQCENRARPCGSERSGKCAAVGICCTEDSCTSDQTCRASPGKEAVAAAVLPRGPSRPQLPSDLVSFLEEVVDNMAPASGSEV*

>*Occasjapyx japonicus* (Diplura) inotocin-like

***M***NPKLTLAILVVALAVLSLTEAQRCPPG**G*KR***CRGSCCGRFQRCCPRGPRKCCPITSACTPLGACVGISDAAADSSASSSEELGDDIADAVADDIADAADADDAMSDDAPLSDVSSDADDH***RR***GGHHHHHHGHHDAGQ*

>*Catajapyx aquilonaris* (Diplura) inotocin

…RTREASACRAENLFPVQCENKARPCGSERSGKCAAMGLCCTEDSCTSDSACRGAPSKEVVAASRGQRPQQLPSDLVSFLEEVVENIAPASAPEV*

>*Pedetontus okajimae* (Archaeognatha) inotocin

***M***KNSSSSTFALFSLLLFISCVTSCFITNCPPG**G*KR***AQGAPGPRGEPRQCPSCGPRRSGRCFGPDLCCGDFGCRLGGRDASCRREMSAPGLCINQGRACGVGVCAAQGVCCSETSCALDASCMDSWSPRQLISVNSGFSSVLENMLEEIPVETGDD***RR****

>*Machilis hrabei* (Archaeognatha) inotocin [GenBank:GAUM01180328.1]

***M***SADMTNSASSTFALFTLLLFVGCVTSCFITNCPPG**G*KR***GLGSPGPRGEPRQCPSCGPRHSGRCFGPDLCCGDFGCRLGARDASCRREMSAPGLCINQGRPCGVGVCAAQGVCCSETSCALDASCMDSWSPRQLVPVNNGLSSVLENMLEDIPMESGDD***RR****

>*Meinertellus cundinamarcensis* (Archaeognatha) inotocin [GenBank:GAUG01237411.1]

***M***KSSMSTPAALFALLFVVSITSSCFITNCPPG**G*KR***AMGAPGPRGVQRQCPSCGPHRSGRCFGPDLCCGEFGCHLGGRDPVCRGEMSAPGLCTNPGRPCGVGKCAAQGVCCSETACALDSMCLDSWPSRNVVQMNSGLASVLENMLEEVPSENGDD***KK****

>*Machilontus* sp. (Archaeognatha) inotocin

…PPG**G*KR***AMGSPGPHGIQRQCPPCGPHRSGRCFGPDLCCGDFGCRLGARDPCRGEMSAPGLCVNAGRPCGVGKCAAQGVCCSETSCAFDSTCLDSWSTRAILPMSNGLASMLENMLEDVPAEAADD…

>*Tricholepidion gertschi* (Zygentoma) inotocin [GenBank:GASO01245888.1]

***M***VARKNPQTVVICLMAAMSAATACFITNCPPG**G*KR***SMGQVGTHATRECTSCGPAKLGRCYGPAICCGPQIGCLVGTREAAVCQTENFYPVPCANRGGQCGGESGRCAATGVCCTEESCSIDPSCHVTPQETILTPVSRQMYPINSAMNNYLEEAVDALPSHVILEN***RR****

>*Atelura formicaria* (Zygentoma) inotocin1 [GenBank:GAYJ01010327.1]

…ACFITNCPPG**G*KR***SMGQMGLHVNKQCTSCGPARTGRCYGPAICCGPRFGCLVGTRDTAVCQTENFTPVPCGNRGPSCGESGRCAANGVCCTEETCTIDPSCRISNEDISSSRQMFPITNSVSMYLEELNDDLPARVGSD***RR****

>*Atelura formicaria* (Zygentoma) inotocin2 [GenBank:GAYJ01211146.1]

…SLSKGILSVTIFFLAVISVTTACFITNCPPG**G*KR***SMGQMGLHANKQCTSCGPARMGRCYGPAICCGPRFGCLVGTRDTAVCQ…

>*Nicoletia phytophila* (Zygentoma) inotocin

***M***FLKYSLLTAAFVLAALSATTACFITNCPPG**G*KR***SMGQMGLRVSRQCTSCGPNKSGRCFGPSICCGPHFGCLVGTREAAVCQTENFYPVPCMNRGATCSGESGRCAANGVCCTEESCSVDSSCRMNGDEVPALTRTLYPINNAVNAYLEDVVDDLSPHMAVD***RR****

..............................................................................................

>*Xibalbanus tulumensis* (Remipedia) inotocin

***M***SNDVLLSVVCIFYVASLSSACFVINCPTG**G*KR***SMGPLKASASRQCTSCGPDDLGRCYS…

>*Anaspides tasmaniae* (Malacostraca) inotocin

***M***THSVLLVLVLTTLVGIATPCFITNCPPG**G*KR***SRVAPLDQLIHAHKPCSPCGPGLTGRCVGPGICCGRSLGCLMGTKEAQMCRVENLSPITCSNAHLRSCGRDAHCAARGICCSGSQCEFDSTCDDIEEPSNSFLSEDNQWNL*

>*Lithobius forficatus* (Chilopoda) inotocin

***M***ASHAAQSSVFLVVLLCVFHITLACFITNCPPG**G*KR***SLGDQKGTTRQCTACGPGGLGHCFGPNLCCGPQIGCFLHTRESAVCRFENLYPVPCDNDAPPCADSGKCAADGLCCTTDECRMDESCHQGKEVATHRISNLYQRMLSNDVDVNDVIASHR*

>*Hanseniella* sp. (Symphyla) inotocin

***M***NRNYSLDLTQLSILLLCVIAMTSACFITNCPIG**G*KR***SGHVTSTVSRQCSSCGPGGEGRCFGPNACCVSGMGCFLGAAHTASCRLEQSYPLPCDNGTPACGESQGGRCAVQAICCTPA…

>*Eudigraphis takakuwai nigricans* (Diplopoda) inotocin

***M***WPVTVTASLLLLVSVTSACFISNCPNS**G*KR***SDPSSKPTASRQCLSCGPRRLGRCFGPNICCGSTIGCVLGSREIDICKLEETYPVPCANKG…

..............................................................................................

No inotocin known from *D. melanogaster*

>*Daphnia pulex* inotocin [[Dappu1_59567](http://genome.jgi.doe.gov/cgi-bin/dispGeneModel?db=Dappu1&id=59567), GenBank:[EFX71881.1](http://www.ncbi.nlm.nih.gov/protein/321460843?report=genbank&log$=prottop&blast_rank=1&RID=XSVNJK3N014)]

***M***AGLWTFCLIALSMTEMIIPLTAKPCFITNCPPG**G*KR***SSQLVEPSSYLECAPCGPAGKGTCLGANLCCGSHFGCFFKTEETNVCLLTNLKSTQICNQHFWKTDLKSASCSLNGDKIDGICVADLLCCSLGNLPQDDL*

Ion transport peptide (ITP/ITPL = ITP long form)

>*Nipponentomon nippon* (Protura) ITP

***M***ALKSSSHTTKALVVALAVVSTLLVASPGADGRVVSSHPLS***KR***SFFDIQCKGVYDKSIFARLDRICEDCYNLYKEPQLHSLCRSNCFSSQYFKGCLDALLLNEESGKFDEMIEFV**G*KR***K*

>*Nipponentomon nippon* (Protura) ITPL

***M***ALKSSSHTTKALVVALAVVSTLLVASPGADGRVVSSHPLS***KR***SFFDIQCKGVYDKSIFARLDRICEDCYNLYKEPQLHSLCRNDCFSSKYFEGCLDALMLREQSDKARIEGWIRHVHGELPF*

>*Acerentomon* sp. (Protura) ITP (TSA1, s11447)

***M***ALKSALHTSAVTFVALCLVSSTLLVSPVDSMVLSHHPLS***KR***SFFDIQCKGVYDKSIFARLDRICEDCYNLYKEPQLHSLCRSNCFSSQYFKGCLDALLLNEESGKFDEMIEFM**G*KK****

>*Acerentomon* sp. (Protura) ITPL [GenBank:GAXE01011436.1, GAXE01011435.1, GAXE01011434.1]

***M***ALKSALHTSAVTFVALCLVSSTLLVSPVDSMVLSHHPLS***KR***SFFDIQCKGVYDKSIFARLDRICEDCYNLYKEPQLHSLCRNDCFSSKYFEGCLDALMLREQNEKTKIQGWIRHLHGELPF*

>*Filientomon takanawanum* (Protura) ITP ***M***ALKSSLHTTAALFVAFSIVTTTFLVSPVESMVLSHHPLS***KR***SFFDIQCKGVYDKSIFARLDRICEDCYNLYKEPQLHSLCRSNCFSSQYFKGCLDALLLNEESGKFDEMIEFM**G*KKK****

>*Filientomon takanawanum* (Protura) ITPL

***M***ALKSSLHTTAALFVAFSIVTTTFLVSPVESMVLSHHPLS***KR***SFFDIQCKGVYDKSIFARLDRICEDCYNLYKEPQLHSLCRNDCFSSKYFEGCLDALMLREQTEKTKIQGWIRHLHGELPF*

>*Anurida maritima* (Collembola) ITP (reconst. from GAUE01003274.1, GAUE01003276.1, GAUE01003275.1]

***M***HSKMSKVSVGKSHRSIGIILAVAVLSLFYVESTQGFVAGSRALN***KR***SFFDIQCKGVYDKTIFARLDRICDDCYNLYREPQLHSLCRSNCFGSPYFKACLDALLLNDDEAKYEEMIEIL**G*KK****

>*Anurida maritima* (Collembola) ITPL (GAUE01003276.1, GAUE01003274.1)

***M***HSKMSKVSVGKSHRSIGIILAVAVLSLFYVESTQGFVAGSRALN***KR***SFFDIQCKGVYDKTIFARLDRICDDCYNLYREPQLHSLCSENCFSTDTFIGCLDSLQMADQWENFRSLITTLHADTSNSLSDSEFDSD*

>*Tetrodontophora bielanensis* (Collembola) ITP [GenBank:GAXI01012682.1]

***M***FAIVITVFIFIMSISSSQGFVAGSRPLG***KR***SFFEIQCKGVYDKDIFARLDRICDDCYNLYREPQLHSLCRSNCFGSPYFKGCLDALLLNDDEAKYEEMIEIL**G*KK****

>*Tetrodontophora bielanensis* (Collembola) ITPL (reconst. from GAXI01012682.1, GAXI01117803.1]

***M***FAIVITVFIFIMSISSSQGFVAGSRPLG***KR***SFFEIQCKGVYDKDIFARLDRICDDCYNLYREPQLHSLCSENCFSTKTFAGCLDALRMADEYPAFKELISTLHADTSNSLSE*

>*Podura aquatica* (Collembola) ITP

***M***SPSSVSQLFHFRMPLLIFTLGTIFLASVSSTEGFVPGSRPQS***KR***SFFDIQCKGVYDKAIFGRLDRICEDCYNLYREPQLHTLCRSNCFGSPYFKGCLDALLLNDEEAKYEEMIEIL**G*KR***K*

>*Podura aquatica* (Collembola) ITPL

***M***SPSSVSQLFHFRMPLLIFTLGTIFLASVSSTEGFVPGSRPQS***KR***SFFDIQCKGVYDKAIFGRLDRICEDCYNLYREPQLHTLCSEKCFSTKTFVGCLDALQLIDDWPYYRGLITTLHADTSNSLHSDKTNSLLSSPSLDY*

>*Folsomia candida* (Collembola) ITP (reconst. from GASX01085982.1, GASX01010497.1]

MHSSSNESVVATKVNHGRRFTSRALTSPAFLVTLGTTLLIATVVVGQADSFVMGSRPLS***KR***SFFDIQCKGVYDKGIFAKLDRICDDCYNLYREPQLHSLCPSNCFGSPYFKGCLDALLLNEEDGKFDEMIEIL**G*KKKK****

>*Folsomia candida* (Collembola) ITPL [GenBank:GASX01085982.1]

MHSSSNESVVATKVNHGRRFTSRALTSPAFLVTLGTTLLIATVVVGQADSFVMGSRPLS***KR***SFFDIQCKGVYDKGIFAKLDRICDDCYNLYREPQLHSLCSQNCFSSSYFMNCLDALTLNDEQKTYEEYIERIHAPTPVER***KK****

>*Bourletiella hortensis* (Collembola) ITP

MNTARSSQTGSHNGAKGSPKLVSSSSTPGYRAIVQPRVVA***M***AVILCMLSSTLMAESFVVGSRPLS***KR***SFFEVQCKGVYDKDIF…

…DDCYNLYREPQLHTMCRSNCFGSTYFKGCLDALLLNEEEGKFDEMIEIL**G*RKK****

>*Bourletiella hortensis* (Collembola) ITPL

MNTARSSQTGSHNGAKGSPKLVSSSSTPGYRAIVQPRVVA***M***AVILCMLSSTLMAESFVVGSRPLS***KR***SFFEVQCKGVYDKDIF…

…DDCYNLYREPQLHTMCRQDCFSTEFFPKCLEALALDHDKHDYREMVNTINAYNPFEGNPLD**G*RR****

>*Orchesella cincta* (Collembola) ITP

***M***NTSQNMSTFLMRNNYGRPTSTSKPSPNPLSSPISQSFRLIAGLLVLSVVLGPVQSFVMGSRPLS***KR***SFFDIQCKGVYDKGIFAKLDRICDDCYNLYREPQLHSLCRSNCFGSPYFKGCLDALLLGEEEGKFDEMIEIL**G*KKK****

>*Orchesella cincta* (Collembola) ITPL

***M***NTSQNMSTFLMRNNYGRPTSTSKPSPNPLSSPISQSFRLIAGLLVLSVVLGPVQSFVMGSRPLS***KR***SFFDIQCKGVYDKGIFAKLDRICDDCYNLYREPQLHSLCSQNCFSSPYFMACLDALTLIED***KK***TYEDYIERIHAPTPVE*

>*Pogonognathellus sp.* (Collembola) ITP (reconst. from GATD01001993.1, GATD01001992.1]

***M***IRLMVGVMILSIVTGSVQSFVMGSRPLS***KR***SFFDIQCKGVYDKGIFAKLDRICDDCYNLYREPQLHSLCRSNCFGSPYFKGCLDALLLNEEEGKFDEMIEML**G*KKK****

>*Pogonognathellus sp.* (Collembola) ITPL [GenBank:GATD01001993.1]

***M***IRLMVGVMILSIVTGSVQSFVMGSRPLS***KR***SFFDIQCKGVYDKGIFAKLDRICDDCYNLYREPQLHSLCSQNCFSSNYFIQCLDALTLSAEQKTYEDYIEKIHAPTPLD*

>*Sminthurus viridis* (Collembola) ITP1 (reconst. from GATZ01017029.1]

MNSKGSCKARKQSSPSTRCQQVG***M***RVLALALVLCILGSSTVDSLVMGSRPLS***KR***SFFDIQCKGVYDKGIFAKLDRVCDDCFNLYREPQLHSLCRSNCFGSQYFKGCLDALLLNEDESSYDEMIEIL**G*KK****

>*Sminthurus viridis* (Collembola) ITPL1 [GenBank:GATZ01017029.1]

MNSKGSCKARKQSSPSTRCQQVG***M***RVLALALVLCILGSSTVDSLVMGSRPLS***KR***SFFDIQCKGVYDKGIFAKLDRVCDDCFNLYREPQLHSLCRSKCFTTSYFVGCLDALTLSDDEEQYQRLITTINAHNPLLENSDIAETQR*

>*Sminthurus viridis* (Collembola) ITP2 (reconst. from GATZ01017029.1]

MNSRSISSLQMQSVSSLRCQQSGLRLLLGLGLLLVVVGSATVESSVMGNRPLS***KR***SFFDIQCKGVYDKSIFSKLDRVCDDCFNLYREPQLHSLCRSNCFGSSYFKGCLDALLLNEEEGKFDEMIEML**G*KK****

>*Sminthurus viridis* (Collembola) ITP3 [GenBank:GATZ01089349.1]

MQRSFGKRFDKVGVWALSFFALVLFLGTTAGSSAVAPRRPYAVS***KR***SFFSIQCRGAYDKDIFEKLNRVCDDCFNLFREPELHVLCTSNCFGSTYFKGCLDALLLSEEE…

>*Sminthurus viridis* (Collembola) ITP4 [GenBank:GATZ01082494.1] ***M***DTSFSQRYRNMGVWALALCLLVIILETSAEGRAMTGSRPLS***KR***SFVNIQCRGQYDRDIFSKLDRVCDDCFNLYREPEVHILCRSN…

>*Sminthurus viridis* (Collembola) ITPL5 [GenBank:GATZ01095285.1]

…REPQLHSLCRKSCFRTPYFFGCLDDLAMGDDREHYQRLISTINAHNPFMETTESSEIF**G***

>*Jordanathrix leptothrix* (Collembola) ITP1

…KKLVIIVCICIFGTSLRTVGSSRLAVGSRALS***KR***SFFNSRCKGIYNKGIFARLDRVCDDCYKLYQEPEIHSLCRSNCF…

>*Jordanathrix leptothrix* (Collembola) ITP2

***M***GRQKQGQIGFQLLTLALVVVACIIGTATASGVRAGVGSFGVGSRSRSLT***KR***SFGEIQCKGIYDKNAFSKLDRVCDDCFNLYQETQLYTLCRSNCFGSTYFKGCLDALLLNEEEDKYDEMIEIL**G*KK****

>*Jordanathrix leptothrix* (Collembola) ITP3

SSRGKGKKGQFQSQVLPPAIILFVIVIVAICTVGTTAAAAVIESETGNFGIGKGSRSPALLLS***KR***SFSNIDCKGVFDKNIFSQLERVCDDCFNLYQETTLYSLCRSNCFGSEYFKGCMDALLLDEEEERYDEMIEIL**G*KK****

>*Jordanathrix leptothrix* (Collembola) ITPL4 (?)

…LVLISCIGSMTATGSFIVGGRPLS***KR***SFFDIECKGVYDKGIFAKLDRVCDDCYNLYREPQLHALCSQKCFS…

>*Campodea augens* (Diplura) ITP [GenBank:GAYN01020445.1 + GAYN01118577.1]

***M***YHIRPFVLLGILLVLVYGALSSPRPNTLTLGHPLS***KR***SFFDIQCKGVYDKAIFAR…

…RSNCFSSEIFKGCVDALLLNDDSAKFDEMIEFL**G*KR***K*

>*Campodea augens* (Diplura) ITPL [GenBank:GAYN01020445.1 + GAYN01118577.1]

***M***YHIRPFVLLGILLVLVYGALSSPRPNTLTLGHPLS***KR***SFFDIQCKGVYDKAIFAR…

…CFTSGYFKGCLEALLLNDKEDEIRDEIQKIRGVPPPL*

>*Lepidocampa weberi* (Diplura) ITP

***M***CVGRPFHFLLVGVVLLAVCDDVFSSPRPNALSLGHPLS***KR***SFFDIQCKGVYDKAIFARLDRICDDCFNLYREPQLHTLCRSNCFSSDIFKGCVDALLLNEDSAKFDDMIEFL**G*KR***K*

>*Lepidocampa weberi* (Diplura) ITPL

***M***CVGRPFHFLLVGVVLLAVCDDVFSSPRPNALSLGHPLS***KR***SFFDIQCKGVYDKAIFARLDRICDDCFNLYREPQLHTLCSENCFTSGYFKGCLEALLLKDKEDEIRDEIQKIRGVPPPL*

>*Occasjapyx japonicus* (Diplura) ITP [GenBank:GAXJ01099843.1]

***M***AAVRTQVALSLIAVALLVATLCPQKSCAMVVGHPLS***KR***SFFDIQCKGVYDKAIFARLDRICDDCFNLYREPQLPALCRSNCFNNPYFKGCLDALLLTEENGDFDRMIDIL**G*RR***K*

>*Occasjapyx japonicus* (Diplura) ITPL [GenBank:GAXJ01099843.1 + GAXJ01010150.1]

***M***AAVRTQVALSLIAVALLVATLCPQKSCAMVVGHPLS***KR***SFFDIQCKGVYDKAIFARLDRICDDCFNLYREPQLPALCRQDCFTSAYFKGCLDVLLLDPNEYRDLVLHINGVDLPN*

>*Catajapyx aquilonaris* (Diplura) ITP

***M***AAVRIQLSHSLMVVSLLVVALCPYTSNAMVVGHPLS***KR***SFFDIQCKGVYDKAIFARLDRICDDCFNLYREPQLPSLCRSSCFNNPYFKGCLDALLLTEENGEFDRMIDIL**G*RR***K*

>*Catajapyx aquilonaris* (Diplura) ITPL

***M***AAVRIQLSHSLMVVSLLVVALCPYTSNAMVVGHPLS***KR***SFFDIQCKGVYDKAIFARLDRICDDCFNLYREPQLPSLCRQDCFTTAYFKGCLDVLLLDPNEYRDLVLHINGVDLPN*

>*Pedetontus okajimae* (Archaeognatha) ITP

***M***HCSRSSLAMAVAVALILAASLSLLAQPADGMVFNHPLS***KR***SFFDIECKGVYDKGIFARLDKICEDCYNLFREPQLHSLCRSQCFSTQYFKGCLDTLLVNDEEQHFNAMIDTL**G*KK***K*

>*Pedetontus okajimae* (Archaeognatha) ITPL

***M***HCSRSSLAMAVAVALILAASLSLLAQPADGMVFNHPLS***KR***SFFDIECKGVYDKGIFARLDKICEDCYNLFREPQLHSLCR***KK***CYTTEYFKGCVDTLLLHEEARKLQDMIQKVYGAGD*

>*Machilis hrabei* (Archaeognatha) ITP (reconst. from GAUM01017110.1, GAUM01183242.1]

***M***HCSRSSLAMVVAVALILAASLSLLAQPADGMVLSHPLS***KR***SFFDIECKGVYDKGIFARLDRICEDCYNLFREPQLHSLCRSQCFSTQYFKGCLDTLLLNDEEHHFNAMIDAL**G*KKK****

>*Machilis hrabei* (Archaeognatha) ITPL [GenBank:GAUM01017110.1]

***M***HCSRSSLAMVVAVALILAASLSLLAQPADGMVLSHPLS***KR***SFFDIECKGVYDKGIFARLDRICEDCYNLFREPQLHSLCR***KR***CYTTEYFKGCVDALLLHEEVDDLQDMIRKVHGAGV*

>*Meinertellus cundinamarcensis* (Archaeognatha) ITP [GenBank:GAUG01020822.1]

***M***QSSRSSLVLAVAFALLTVASLTLLARPAEGMVLGHPLS***KR***SFFDIECKGVYDKGIFARLDRICEDCYNLFRDPQLHSLCRSECFSTPYFKGCLDALLLNEEEGRYNAMIDKL**G*KR***SEE*

>*Meinertellus cundinamarcensis* (Archaeognatha) ITPL [GenBank:GAUG01020821.1]

***M***QSSRSSLVLAVAFALLTVASLTLLARPAEGMVLGHPLS***KR***SFFDIECKGVYDKGIFARLDRICEDCYNLFRDPQLHSLCR***KR***CYTTDYFKGCLDTLLLHGEVEEIQGMIRKVHGAGV*

>*Machilontus* sp. (Archaeognatha) ITP

***M***QSRSSVIVAVAAALLVVATLSLLASPADGMVLSHPLS***KR***SFYDIDCKGVYDKGIFARLDRICEDCYNLFREPQLHSLCRSECFSTPYFKGCLDALLLNEEEGRYNAMIDKL**G*KR***SDE*

>*Machilontus* sp. (Archaeognatha) ITPL

***M***QSRSSVIVAVAAALLVVATLSLLASPADGMVLSHPLS***KR***SFYDIDCKGVYDKGIFARLDRICEDCYNLFREPQLHSLCR***KR***CYTTEYFKGCVDSLLLEKEVEDIQGMIRKIHGAGV*

>*Tricholepidion gertschi* (Zygentoma) ITP1 (reconstructed from GASO01016886.1]

***M***QLITRITVCLQALVVLLSCFLVGPAECRVLGHSVK***KR***SFFDIQCKGVYDKSIFAKLDRICEDCYNLYREPQLHSLCRSNCFTSPYFKGCLEALLLTEKEEKFNEMIEFL**G*K****

>*Tricholepidion gertschi* (Zygentoma) ITPL1 [GenBank:GASO01016886.1]

***M***QLITRITVCLQALVVLLSCFLVGPAECRVLGHSVK***KR***SFFDIQCKGVYDKSIFAKLDRICEDCYNLYREPQLHSLCRKSCFTTEFFEGCMEALLLKDKMENIQKWIKQLHGADPGV*

>*Tricholepidion gertschi* (Zygentoma) ITP2 [GenBank:GASO01013068.1]

***M***QTMYLQVAVLLLSCFLVGPSESMVFSHPLS***KR***SFFDIQCKGIYDKSIFAKLDRICEDCYNLYREPQVHSLCRSNCFGSQYFKACLESLLLMEEEQKYNRWIELL**G*KK****

>*Tricholepidion gertschi* (Zygentoma) ITPL2 [GenBank:GASO01013069.1]

***M***QTMYLQVAVLLLSCFLVGPSESMVFSHPLS***KR***SFFDIQCKGIYDKSIFAKLDRICEDCYNLYREPQVHSLCRQDCFTTQYFKSCLVVLLLEDQEEQIKTWITQLHGVDAGA*

>*Thermobia domestica* (Zygentoma) ITP (reconst. GASN01020061.1, GASN01020059.1]

***M***QTRITACLLAAVVFVSCFLVVPTNSYIIGHTLS***KR***SFFDIQCKGVYNKEIFAELDKVCEDCYNLFKEPQLHTLCRSDCFGSRYFKGCLEALLLSEEEKKYNQMIEFL**G*KK****

>*Thermobia domestica* (Zygentoma) ITPL [GenBank:GASN01020061.1, GASN01020059.1]

***M***QTRITACLLAAVVFVSCFLVVPTNSYIIGHTLS***KR***SFFDIQCKGVYNKEIFAELDKVCEDCYNLFKEPQLHTLCRKGCFTTFYFKGCLDALLIDNSELETIKTKIRLINGADPGV*

>*Atelura formicaria* (Zygentoma) ITP (reconst. from GAYJ01270728.1, GAYJ01029755.1]

***M***QTRITACLLCVSVLAACLLISPTESMVASHPLS***KR***SFFDIQCKGVYDKSIFAQLDKICEHCYNMYREPELHSLCRSNCFGSSYFKACVEAQLLNEEWDEY***KR***MIEIL**G*KK****

>*Atelura formicaria* (Zygentoma) ITPL (reconst. from GAYJ01270728.1, GAYJ01029755.1]

***M***QTRITACLLCVSVLAACLLISPTESMVASHPLS***KR***SFFDIQCKGVYDKSIFAQLDKICEHCYNMYREPELHSLCRNDCFTSDYFKGCVDVQLLHDELDKIQSWIKQLHGAEPGI*

>*Nicoletia phytophila* (Zygentoma) ITP

***M***QMQTRIALCVLSFALLMSCLLVSPTQSMVVGHPLS***KR***SFFDIQCKGVYDKTIFARLDKICEHCYNMYREPEVHSLCRSNCFGSPYFKGCLEALLLTEEEEKYNQMIEYL**G*KK****

>*Nicoletia phytophila* (Zygentoma) ITPL

***M***QMQTRIALCVLSFALLMSCLLVSPTQSMVVGHPLS***KR***SFFDIQCKGVYDKTIFARLDKICEHCYNMYREPEVHSLCRKDCFTSDYFKGCVEVQLLHDELDKIQSWIKQLHGAEPGI*

..............................................................................................

>*Xibalbanus tulumensis* (Remipedia) ITP

***M***QSLRLNLPLAVLAGVFTIQLVIPTEAMIVPRTIS***KR***SFLDIECKGVYDKSIFTKLNMVCEGCYHLYRDPELHTLCRSNCYNNKYFKGCLEALLMKDQEEEFLQMVDYV**G*RKKR***SFPEV*

>*Xibalbanus tulumensis* (Remipedia) ITPL

***M***QSLRLNLPLAVLAGVFTIQLVIPTEAMIVPRTIS***KR***SFLDIECKGVYDKSIFTKLNMVCEGCYHLYRDPELHTLCKQDCWSTKYFEGCLNALVRGAEKEEIKDILNDLH**G***

>*Lithobius forficatus* (Chilopoda) ITP

***M***NSSLSLGAMALLAAIACLVPTVPARSFFRQDAPDSEFEVQAHHVLS***KR***SFNKIGCLGFYDKSDFARLDRICEDCCHLYRDSDIHPMCRKDCFTSETFEKCAKALLINLKEEKIQDMVDRLY**G*K***E*

> *Hanseniella* sp. (Symphyla) ITP1

***M***RGIGHLLWAGVGAVFLVLVLLVDSRSIQ***KR***SFADLRCRGDYSRSIFARLDRVCQECYTLYREHSLHRYCRNNCFKNDIFDRC***KK***ALMLDE*

> *Hanseniella* sp. (Symphyla) ITP2

***M***QRSGLRTLAGVGVVLLAVIGLVGARYVE***KR***SFADLRCLGEYDKSIFARLDRICHECYTLYREQTLHRDCRDNCFKNDIFSGCIKALLLEDERSKLEDMVDLMY**G*R***Q*

>*Hanseniella* sp. (Symphyla) ITP3

…DLRCLGEYDKSSYGRLDRICHECYTLYREESLHRDCRENCFKNDIFNGCIRALLLEDEKSKLEGMVDLMY**G*R***Q*

>*Hanseniella* sp. (Symphyla) ITP4

***M***RMRGIGPNCLVGVGVVLLALLGLVAARVIE***KR***SFADLKCLGEYDKPSFARLDRVCHECYTLYREESLHRDC…

>*Eudigraphis takakuwai nigricans* (Diplopoda) ITP

***M***VRVSCSRGSRCVVVLAILLAHVLLISPQGSAALII***KR***SFVKIGCFGEYDKSTFAKLDKICEECYNMYREPGLHNYCRRDCFKNDYFTNCVKALLLDDQEQQIKDMVNRLYGAAE*

..............................................................................................

>*Drosophila melanogaster* ITP [[FBpp0289423](http://flybase.org/reports/FBpp0289423.html)]

***M***CSRNIKISVVLFLVLIPIFAALPHNHNLS***KR***SNFFDLECKGIFNKTMFFRLDRICEDCYQLFRETSIHRLCKQECFGSPFFNACIEALQLHEEMDKYNEWRDTL**G*RK****

>*Drosophila melanogaster* ITPL PC [[FBpp0288657](http://flybase.org/reports/FBpp0288657.html)]

***M***CSRNIKISVVLFLVLIPIFAALPHNHNLS***KR***SNFFDLECKGIFNKTMFFRLDRICEDCYQLFRETSIHRLCKANCFVHETFGDCLKVLLIDDEEISQLQHYLKVINGSPYPFHKPIYH*

>*Drosophila melanogaster* ITPL PD/PF/PG [[FBpp0288658](http://flybase.org/reports/FBpp0288658.html)]

***M***CSRNIKISVVLFLVLIPIFAALPHNHNLS***KR***SNFFDLECKGIFNKTMFFRLDRICEDCYQLFRETSIHRLCKKDCFDSKWFGECLKVLLIPEEEISNLQHFLRVVNGSPISFNMGPQT*

>*Daphnia pulex* ITP1 [[Dappu1_249221](http://genome.jgi-psf.org/cgi-bin/dispGeneModel?db=Dappu1&id=249221)] ***M***AAVQQNGRQTHSSYSIRSLLGLTTLLVVLLAILPYYPTSAMSALSSGHHSLS***KR***SFFDINCKGLYDKSIFARLDRICQDCYSLYREPELHTLCRSECFTTPFFKACLKVLLMEDQDPDYSEMIDKI**G*R****

>*Daphnia pulex* ITPL1 [Dappu1_55390](http://genome.jgi-psf.org/cgi-bin/dispGeneModel?db=Dappu1&id=55390)]

***M***AAVQQNGRQTHSSYSIRSLLGLTTLLVVLLAILPYYPTSAMSALSSGHHSLS***KR***SFFDINCKGLYDKSIFARLDRICQDCYSLYREPELHTLCRKNCFTTNYFKGCLDALLINDEKDIQRVMKDISIIHQIPI*

>*Daphnia pulex* ITP2 [DappuITPN; [Dappu1_307029](http://genome.jgi-psf.org/cgi-bin/dispGeneModel?db=Dappu1&id=307029), GenBank:[EFX63566.1](http://www.ncbi.nlm.nih.gov/protein/321452094?report=genbank&log$=prottop&blast_rank=1&RID=XSWS5ETG015)]

***M***DSGRQSHSSCSIRTLLGLTTLLVVLLAILPYYPTSAMSALSSDHHSSCKGLYDKSIFYRLNRICHDCFSLYRSPELHTLCRSECFTTPFFKACLKVLLMGDQDPDSSEMIDKI**G*R****

>*Daphnia pulex* ITPL2 [=DappuITPLN, [Dappu1_57088](http://genome.jgi-psf.org/cgi-bin/dispGeneModel?db=Dappu1&id=57088)]

***M***TFQLLNKSAKTGLIVLLIMASLILAEQPPGISIFPHPLSKHSFTEISKCDGVYDMNIYAQFNQICLNCYNLYRQPEIYRGCRKECFTSEYFGGCLAVLQITDKKDKILEDLSIIHRQMESPKEN*

Insect kinin

>*Nipponentomon nippon* (Protura) kinin1

…RTPEEVPADIPQQAEDNEAVQYEEAQWNSDPVTSGLSGHGTE***KR***SVKQFKTWS**G*KR***GWVWMPQDKAGSGEVLGGKAQ***RR***SWTRS… (frameshift?)

…AEEGHEQEEKGDGSSHKLA***KR***ELADDAGSEAHVPIKDTTKQTALTGGNSRSEQYSQPTRYYNILTEVR***KR***NSLRSKLHPGDTVFGSREQ***R***FQPWA**G*KR***DGHT*

>*Nipponentomon nippon* (Protura) kinin2

…QLAKPKINQQSDLVSSQNDKVITVK***KK***SKLEFHPNFSSGEYKARDQ***R***FQPWG**G*KR***SNKK*

>*Acerentomon* sp. (Protura) kinin [GenBank:GAXE01028577.1]

***M***VMYSRRSEASFPILLALSAMAWLLALGESATVAQDEPEDQLQISPQFLQNAIPYNSNSHEGKFNKD***RR***LALFGIANRYRYPILYSQGSSGEMGDPILRGQ***R***MFSNWG**G*KR***GGFNSWA**G*KR***VPEEAPSHLPQSLQEAQHAFEEPDEDVTDDGVSDMLTRVQ***R***SSFGQWG**G*KR***NKQFQSWG**G*KR***GWVWVSQGEPFHAGSN***KR***YPV***RR***SFQSWG**G*KR***SQIPSSIRSIQDDTTDLESGNVDRKVEDLEAPFSKPKETSDRLQSSSQSSHGIQVNKKNKMAEFQRNYNSGEYRPRDQ***R***FQPWG**G*KR***NS*

>*Filientomon takanawanum* (Protura) kinin

***M***VSHRPHSESWVSFVLVISSLIVLSVLEAEGETSPYLLSPESNQQPGIKYYQNAIPYISDSGEKSSKERKFSLFGLANRYRYPILYPLGGGSMESVSPIHRGS***R***MFSNWG**G*KR***GAGFTSWG**G*KR***GAGFTSWG**G*KR***TPEEVPLHLPQINFEDEGHEQEVEWSQMPDAEMDSRMQ***R***SSFGQWG**G*KR***DKAFQSWG**G*KR***GWVWMTQDGKAKAHKTSPI***RR***SFQSWG**G*KR***SEVSKLKPNNIMNSPPEETRNTEAREGDNQNQHNDENAEINQEIIDDPEQLAKSKINETSDLVTIQNDKDIAVK***KK***SKMEFHPNFSSGEYKARDQ***R***FQPWG**G*KR***SSKK*

>*Anurida maritima* (Collembola) kinin [GenBank:GAUE01000288.1]

…FFVLMVLTLTVRSSTSSMDTKVVDSGDNMDESLLPVEGIYGQSFGGGSQEGIGSY*FGL***G*KR***GAPFNSWG**G*KR***Fx*

>*Podura aquatica* (Collembola) kinin

***M***FEMKFFIILVLLSLITLSVSNPLLSENEGSEDGISYEMS***KR***TPFNSWG**G*KR***SLKNGEDSTSNNYLHLSPLFVLFPHQHY**G*KR***EQTGKENI*

>*Bourletiella hortensis* (Collembola) kinin

***M***AVTTNLHFLLGLLFIIVFNSSTLEGAGIGDTAAAVGDGTVSVNNNEDIPRLWR**G*R***ERVDNLRSALELAEIYNNEPFLFPTEP***KR***TFNPWG**G*KR***SGGGQDEMFRSYSDASLNDECKSAFGPWC**G*KK***RSSSSNSNNNEYSSSDNRIY***R***KNTRSGFNPWG**G*RR****

>*Orchesella cincta* (Collembola) kinin

***M***KSIFAAVVTCLFVISLRFYPATAAKEEPEDESNRSLNNVYDSVLEELASSLRPEDLYPLCTSFAAYYDRLQ***KK***SFNPWG**G*KR***VDTGMNAFSDF***KR***NNFNPWG**G*KR***SVAGEAPGME***KK***ENNFNPWG**G*KR***SPVGESFPELD***KK***QNNFNPWG**G*KR***SVAGEGSNME***KK***ENNFNPWG**G*KR***STAGEPFPELD***KK***QNNFNPWG**G*KR***STAGERYPELE***KR***NNFNPWG**G*KR***FYTPDEFEK***KR***NNFNPWG**G*KR***HAFLLSRTIKK*

>*Pogonognathellus sp.* (Collembola) kinin [GenBank:GATD01092450.1]

***M***TRISIASVVALLVINASIRGNNVMAAMEDAEDRFLGKTEMNEEEESLSTLDGKQMIRTPELDLILDDVISLCPQLYGASERYIF***KR***NFNPWG**G*KR***SSQTALHHQIANMN***KK***GGTFNPWG**G*KR***DAINNNSPTASKNDPLI***RR***TPFNPWG**G*RR****

>*Sminthurus viridis* (Collembola) kinin [GenBank:GATZ01065124.1]

…DDLPSVGFNELDMDPIFRPEDLPNICRNFYLASLP***KR***NFNPWG**G*KR***GGSFNPWG**G*KR***HSMGSIESSSNRVTKNDSPDKSRA…

>*Jordanathrix leptothrix* (Collembola) kinin

…TKEELDGGGLSFPSSDGDYSSGGNEVNMLDGDGSNSNNLVPENNLGNLCELLYNSRFN**G*KR***NFNPWG**G*KR***SASASASSRYLGYSPECDYYRQISEY**G*KR***ATAFNPWG**G*KR***SSNSNADSSSSGNSRNNRVAKNDKT***R***TAFNPWG**G*K****

>*Lepidocampa weberi* (Diplura) kinin

***M***SVKSAVLTLLLISAVTAQFEPWG**G*KR***GNGFNPWG**G*KR***GNGFNPWG**G*KR***TGFNPWG**G*KR***SDETPLYLIQHSAEDTGSQDEFPERHPLPDIYPCLAFLGYPAEMSSEQSPLHPFV**G*KR***SSSTSSTSNKPTNVP***R***SPKFYPWG**G*KR***SDHDHPAEDAPNEE*

>*Occasjapyx japonicus* (Diplura) kinin [GenBank:GAXJ01105973.1]

…LLDVLNAIPAALYDNDWAGVDGNDAAAAGKQMDIASLVPGSGLEYAGPVNFLISKLCNRQLSQQSSMDSNP***KR***SEEPFCSTFLGLPMDSGS***R***QASFNPWG**G*KR***SATKPRPEKPSST***KK***QAQTSQFYAWG**G*KR***SVRMQPSTLVPPMSLENQAT*

>*Catajapyx aquilonaris* (Diplura) kinin

…STSAGFNSWG**G*KR***NPVTWDPDAAVPPAPFNSGDSSISYEDRLNLESADPDTPTFLYKAQHGGKQAQPEDGTVTFLISHLCRNQKLLRNAPEQSSVTDKSDEKPMEGVPFCFTFLGLPLDSE***KK***QDVSNSWG***TKR***ISPIKPQVEKPST***KK***HSQTPQFYAWG**G*KR***IARMQPLFSTLLPPIIMENQMT*

>*Pedetontus okajimae* (Archaeognatha) kinin

…LDCYSSGKSQSNQIKDLVDLFPSTNHIKDSYT***KR***QPFSPWG**G*KR***SQSSSENKNNDVVFSSWG**G*KR***SSIIPIVLSDDLFLSSPSQS…

>*Meinertellus cundinamarcensis* (Archaeognatha) kinin [GenBank:GAUG01086363.1 + GAUG01196449.1 + GAUG01249532.1]

…***KR***ATKQSPAFSTWG**G*KR***SDFSNWG**G*KR***SVEKSYLSNGVNDGIDVNKSPSFSSWG**G*KR***SAFSNWG**G*KR***AGFSNWG**G*KR***A…

…GFSNWG**G*KR***AGFSNWG**G*KR***ESLFNSGEDADEFANEFSDFPRWIGEGHNILDEERPTLSDRNGNIDVLDIA***KK***ASFSTWG**G*KR***AGFSNWG**G*KR***AG…

…FSNWG**G*KR***AGFSNWG**G*KR***GDEGNLKANLASWSKNKNEAVD***KR***PSFSNWG**G*KR***SVFDDQFQSTLSNDEIGDSQIP***KR***AGFSNWG**G*KR***APLSTWG**G*KR***APFSTWG**G*KR***APFSTWG**G*KR***APFSTWG**G*KR***AGFSNWG**G*KR***DDPSNWEYTTPGISSLGDNTNNVEFPYIDIQGEPDSVLNVQ***KR***SPISNTKNKNYLSNDYNNEAIFAVTEFETKHHEPRDFVNDTQPINRNHILKLDANKNLKKSNEEDHTRS***KR***SVSLPLWKALVCLDCNTVKEPTSNYQSLNNRQNRDWY***KR***KVPFNPWG**G*KR***SVSTVETAASKAGDFYPWG**G*KR***SSPTQVIQLPADILWSSNFMLRNSDENNRNDSDEEIALLL*

>*Machilontus* sp. (Archaeognatha) kinin

…**G*KR***AGFSNWG**G*KR***NGFSSWG**G*KR***NGFSSWG**G*KR***SGYSNWDLENDYDYRVPDIQEWSDENVDYPNWDDNGEDLHRF***KR***AGFSNWG**G*KR***GDSESNW**G*R***KTDYSDY***KR***AGFSNWG**G*K***…

>*Tricholepidion gertschi* (Zygentoma) kinin [GenBank:GASO01242861.1 + GASO01018172.1]

***M***SLWPHFWLLFLVARAMRCVELGDTEGQQTDVAEGLTPLGNLCADQSDETTFIFSICQMLEQLLQSEPDPDCDRGRQVKCLSSSSGG***KR***TPGFSSWG**G*KR***VGPGFSSWG**G*KK***SGPGFSSWG**G*KK***SGPGFSSWG**G*KR***LGVGPGFSSWG**G*KR***GEGPESSSHGESLAGPGFSSW**G*KR***AGPGFSSW…

…FSSWG**G*KR***VGPGPGFSSWG**G*KR***EEYDTPSHGETFSRHAFTSWRD***KR***PGPGFSSWG**G*KR***VGPGFSSWG**G**KRDEPESLSQVEVQAVPGFSSWED***KR***AGPGFSSWG**G*KR***VSNGPGFSSWG**G*KR***DESDSLSQGDMQEGPVFSSWS**G*KR***AGPGFSSWG**G*KR***DGSGFSSWG**G*KR***NSESNDQEIGALFSPENAGNDDPNTITASTTEKDVRS***KR***SVELPGESPQQEKAGGGLSPCEDCNETLGWPG**G*KR***GSFSPWGN***KR***IANQRFRNQQLQRLLVRQY***RR***QGDFFPWG**G*KR***SDSKLVEPPVISSTSDSNSIWEN*

>*Thermobia domestica* (Zygentoma) kinin [GenBank:GASN01031436.1 + GASN01292275.1]

***M***LISWLWMLLLGYAAADTEILPWSDPKPDTGVPDDENTTLPDLIKTCKEQSTGSSSISPLCQLIENVVQSSEDTSLSLHEEPKTVQQVTTSCSNDDGDDDEDNNENYNEASENDDAD***KR***PIRAFSNWG**G*KR***GSSFSSWG**G*KR***GSAFSNWG**G*KR***GSFSSWG**G*KR***TPFSTWG**G*KR***NTAFNSWG**G*KR***AGFSSWG**G*KR***NFIPIFPS***KR***EGGPGFSSWG**G*KR***DGSTFSSWG**G*KR***NPEQNDVQSTPLYELLNEENE…

…DKNCDDITNWPST***KR***ATIGWNGS***RR***SIPTQQRTKQQDKSTGIQFFPWG**G*KR****

>*Atelura formicaria* (Zygentoma) kinin [GenBank:GAYJ01020764.1 + GAYJ01002885.1]

***M***SPHPLVYLLLLGLTVVIQAQTSPSAPRETDAADPVADDSRLSRLLGACREQAEASGCLSPLCAMAAQLMQDTQQDDGVLEAITQALEAYDKNTPALPCLGEEDTLTANELRTELSDAGD***KR***GHFSSWG**G*KR***GFSSWG**G*KR***GNQFSSWG**G*KR***GNQFSSWG**G*KR***…

…GFSSWG**G*KR***DVVADSSEKDDRETTTEEQAPRSDGKY***RR***GFSSWG**G*KR***DTGDVLES**G*KK***KEFSSWG**G*KR***TLLPWSDNKQDSEEDYSGSQEETEVAVPKSAV***KR***DATRH***KR***SLTSSEMWDADSCLDCKHPEI***KR***FLSWSSWGDGPSSATEPQDQRPLKNRQH***RR***QGDFFPWG**G*KR***SQWSLSLIPLRDGSLELDK*

>*Nicoletia phytophila* (Zygentoma) kinin

…HDPQFGTWA**G*KR***GPQFGSWG**G*KR***SQFSSWG**G*KR***GPQFTSWG**G*KR***GTQFTSWG**G*KR***DPQFSSWG**G*KR***DPKFSSWG**G*KR***DT…

…GTQFASWG**G*KR***GPEFSSWG**G*KR***DFDESEEEDSEEEPQEEE***KR***GFSSWG**G*KR***DSESSTMLATQATDD***KR***GFSSWG**G*KR***TGQGFSSWG**G*KR***NGFSSWG**G*KR***NGEDPIPLDE***KR***QSFSSWG**G*KR***QAFSSWG**G*KR***QPFSNWG**G*KR***QGFSSWG**G*KR***QGFSSWG**G*KR***SLPEDETEESESAESIEAPGQEESLEPCSDCKPQPK***KR***FYYAPWGNWHTSPITQDSNDQKVVKIRLH***RR***QGDFFPWG**G*KR***SNST*

..............................................................................................

>*Drosophila melanogaster* kinin [[FBpp0075465](http://flybase.org/reports/FBpp0075465.html)]

***M***AKIVLCMVLLAFGRQVYGASLVPAPISEQDPELATCELQLSKYRRFILQAILSFEDVCDAYSSRPGGQDSDSEGWPFRHYAPPPTSQRGEIWAFFRLLMAQFGDKEFSPIIRDAVIERCRIKSQLQRDE***KR***NSVVLGKKQRFHSWG**G*KR***SPEPPILPDY*

no kinin known from *D. pulex*

Myoinhibitory peptide/Allatostatin B (MIP/AST B)

>*Nipponentomon nippon* (Protura) MIP

…***KK***EWKSFSGSW**G*KR***EPSSLMDQEVDDSQYSEEKDTSQTQETD***KR***TTWNRLGGSW**G*KR***RAPELAGIQIAQYADSRPFLME***KK***SDWSSFKGSW**G*KR***GPGWNNLKGLW**G*KR***SAGNKWNNLSAAW**G*KR***NTQEDIDTQAQSGENLNNTHRE*

>*Acerentomon* sp. (Protura) MIP [GenBank:GAXE01112447.1 + GAXE01138710.1]

***M***LLTDRVPNRQLLFPLLCLVPIFAFSLATLTSDSDGVISSEEASAPEVAEPFQLQ***KK***AWKNLQGSW**G*KR***AWDKLQGSW**G*KR***…

…LQNSW**G*KR***AWDKISPAW**G*KR***GPILKDSEIKITEFTPSED***KR***EWKSFSGSW**G*KR***DQSEDDLVGSGTNGDAGM***KR***TTGLNKHNPSW**G*K***MSITGDDTLD***RR***QEQFDNTPFLVD***KR***SQWSNLRGSW**G*KR***GPGWNNLKGLW**G*KR***SAPS***KR***SNLSAAW**G*KR***PQNEDIERSSDIPETATN*

>*Filientomon takanawanum* (Protura) MIP

***M***RVHPMSSSVQVFAIVCVVPLFTGCSLAGSFPDPDPGRPPSLIPSNAVLVSSPEVAEPLHLE***KR***AWQNLQGSW**G*KR***AWDKLQGSW**G*KR***RWQDLQNSW**G*KR***AWKDLQNSW**G*KR***AWDKFSPSW**G*KR***PYLGDSEIEITEYSPAED***KR***EWKSFSGSW**G*KR***EQPGVDAAEEAGSGRGP***KR***AGWNKLNSAW**G*KR***TPDEEAPQHDSRPFLMA***KR***SDWSNFRGSW**G*KR***GPGWNNLKGLW**G*KR***SGTNKWNNLSAAW**G*KR***SVNDEKDRPSEQQSDGQSN*

>*Anurida maritima* (Collembola) MIP1 [GenBank:GAUE01050706.1]

***M***KMLLQRPLHFLFMTTFGLFFFIKLVFPFDPSQETGNGDIWN***KR***AWNDVTGSW**G*KR***GWSDMEGGW**G*KR***VWERLDGPSEKELSLSSVPVNELEESPELA***KR***AHWSNLKGLW**G*KR***FGGKEDSQLWNEMSLNRLRQ***KK***AWENLRNSW**G*KR***SENIIRDELEKA***KK***Q*

>*Anurida maritima* (Collembola) MIP2 [GenBank:GAUE01053856.1]

***M***ISERLWLLVTLSAILFFILLSSSHGVVASSPLGYEDNEEGEMMN***KR***GWKDVSGSW**G*KR***GWNDLEGAW**G*KR***AWERLNGAW**G*KR***LSLIPVPDEDGDDVVPSEIN***KR***PEWAKFKGSW**G*KR***ALLRQMQQRL*

>Tetrodontophora MIP [GenBank:GAXI01154530.1]

***M***MLSMTHSMFLVLSALLVVVVWAAGDVKTGDEDDLVLN***KR***GWNDVSRSW**G*KR***GWGDLQGQW**G*KR***GWDRLNGAW**G*KR***LSPGDSDLEEFWDGIE***KR***PEWGKFKGSW**G*KR***ATAGSSAGWSNLKGLW**G*KR***AWDKLANSW**G*KR***SENEDDLPNLNDFN*

>*Podura aquatica* (Collembola) MIP

***M***ISVNQWMLLLLSGVLIVLISATPEVKSPTTNSEDDDDELMA***KR***GWKDISGSW**G*KR***GWNDLQGAW**G*KR***AWERLSGAW**G*KR***LSIPPYEEQDEYLSEIN***KR***PEWAKFKGSW**G*KR***GNGGSQGWSNLKGLW**G*KR***AWDSKFGNSW**G*KR***SAMEIEQEQPGSLSEMN*

>*Folsomia candida* (Collembola) MIP [GenBank:GASX01058719.1, GASX01025593.1]

…SLICLLALLASSSQADPSPDSQHEASPDDSEAVTSGQDD***KR***GWKDLQQGW**G*KR***GWSDLQQPW**G*KR***GWSDLQSSW**G*KR***AWQDLQAAW**G**…

…KGIFGITHTSLDGRKMNSFNCWIILSASAFLLLLISTTASSQEATSLQDEDLSNS***KR***AWKDVGASW**G*KR***GWKDMQG…

>*Bourletiella hortensis* (Collembola) MIP

***M***IYFNRWVLLVVALVLAIVVSASQQDNTKADEDIVI***KR***GWKDTSGAW**G*KR***GWNNLQGNW**G*KR***GWERLNPVW**G*KR***NAAAASAGTDEPDMEDFWDGID***KR***PDDWANLRGAW**G*KR***TAGQGWSNIRGLW**G*KR***SWGNINNAW**G*KR***STNDEEAGDSN*

>*Orchesella cincta* (Collembola) MIP

***M***NYLNCCCLVFILSSFLLVVISASAISSQELKDPTTEEDLGSTISDTVS***KR***AWKNLGAAW**G*KR***AWADMQKAW**G*KR***RGWDRLSAAW**G*KR***MAPSEFASEPSNDSDEVLYGMD***KR***PEWSSLKGSW**G*KR***ANGHSGWSNLKGSW**G*KR***AAWNKLDKAW**G*KR***STDTDDETGN*

>*Pogonognathellus sp.* (Collembola) MIP [GenBank:GATD01015795.1]

***M***NYLSPWLLLLLSSALVFVIVASQEIPNGDDEDIVI***KR***GWKDMGTPW**G*KR***AWGDMQGTW**G*KR***GWDKLNGAW**G*KR***MTTDGPDSNDFWELID***KR***PEWGKFKGSW**G*KR***AGTGWNNLKGLW**G*KR***SWGKMNNSW**G*KR***SSEIGE*

>*Sminthurus viridis* (Collembola) MIP [GenBank:GATZ01104982.1]

***M***IYTNRWILLLLSMVLVVVVSASQETKNTADEDIVI***KR***GWKDAAGGW**G*KR***GWNNMQGNW**G*KR***GWERLNPVW**G*KR***MAADEPADLEDFWDGVE***KR***PDDWANLKGAW**G*KR***SAGQGWSNIKGLW**G*KR***SWGNIQNSW**G*KR***SANDDDVAAEFN*

>*Jordanathrix leptothrix* (Collembola) MIP1

***M***NWCIIAFFPLILLVVGTVVSESGQTRSQDGNDEDILLVS***KK***AWKDIHPVW**G*KR***CWDCIEENMD***KR***GWERLQTGW**G*KR***SRLETPLTLALTNDQKTPDWQKFRGTW**G*KK***SDANSGWRNLKGTW**G*KR***SLEIEESTEKP*

>*Jordanathrix leptothrix* (Collembola) MIP2

…SQETKNTADDDIVM***KR***GWKDTSGVW**G*KR***GWNNMQANW**G*KR***GWERLNPVW**G*KR***TGTEEQPDSQDFLDGID***KR***PDDWQNLKGAW**G*KR***SAGQGWSNIKGLW**G*KR***SWGNIQNSW**G*KR***AANEDEFASDVN*

>*Campodea augens* (Diplura) MIP [GenBank:GAYN01132746.1]

…DENDDNEIDYDVA***KR***NWDNLRGAW**G*KR***AYDDEEQVD***KR***GWNNFKGSW**G*KK***DAGWNNLRGLW**G*KR***SDSWNKLSSAW**G*KR***SPVEDESATSKDTLAHTD*

>*Lepidocampa weberi* (Diplura) MIP

***M***HNVVMTTIHLASTLLLVASFCRASSETLNNNAGASSSQNDNQEHKSSVE***KR***GWNDLQGAW**G*KR***AWDDLQGAW*GG*DDLQGAW**G*KR***DDLQDEQE***KR***AWNDLHSAW**G*KR***AWDDLHSAW**G*KR***AWGDLQGAW**G*KR***AWNELNSAW**G*KR***SGEGWERLQGAW**G*KR***SGDATEEDDEMV***KR***NWENLHGAW**G*KR***GYEDQEEAD***KR***TDWNNFRGSW**G*KR***GGNNNDVAWSNLKGLW**G*KR***GDRDWNKLSAAW**G*KR***SVGAEESSTSKDAEAHAAE*

>*Occasjapyx japonicus* (Diplura) MIP1 [GenBank:GAXJ01008949.1]

***M***QYVLVGLIYFTSALVGSSVIGRVQAESPAAVAAGLHVPLAEAPLTGADHPSDLQD***KR***AWADLQQGW**G*KR***GWSDLQQGWx***KR***GWNDLQGSW**G*KR***GWNDLQGAW**G*KR***GWNDLQHAW**G*KR***GWNDLQGAW**G*KK***RGGAGWEKLRGAW**G*KR***GTDGGPDPGEEYARLMMMQQQGQREGDEEGEEEEYGGQQHEED***KR***DWNNLRGSW**G*KR***SAAGEDFD***KR***GDWSSFRGSW**G*KR***DPGWSNLKGLW**G*KR***SDSNWNKLSAAW**G*KR***SVPEESPAISNAATRTEE*

>*Occasjapyx japonicus* (Diplura) MIP2 [GenBank:GAXJ01060696.1]

…DLLDED***KR***GWNNLRGAW**G*KR***SEDD***KR***GDWNSFQGSW**G*KR***DPGWNNLKGLW**G*KR***SDNWGKLAAVW**G*KR***STEGQTGVKQEDNR…

>*Catajapyx aquilonaris* (Diplura) MIP

***M***QYVIVGLIYFTSALVGSSVIGRVQADSVAVHVPIGEEPLHGADNQALEG**G*KR***GWSDLHQQGW**G*KR***GWGDLQGSW**G*KR***GWNDLQGAW**G*KR***GWNDLQGAW**G*KR***GWNDLQGAW**G*KR***AWEDLQSSW**G*KR***GGNWDKLRGAW**G*KR***DEPEAAEDYEEEAAAAPPPLPPMGI***KR***DWSNLRGSW**G*KR***SDGGDEEEVE***KR***GDWSNFRGSW**G*KR***DPGWNNLKGLW**G*KR***ADSNWNKLSAAW**G*KR***SVPEESSAVSNGASRKEE*

>*Pedetontus okajimae* (Archaeognatha) MIP

***M***QHAVITSALYAITALILSSLLGGSIAEPSSSHALQGEAVSEGEAAALGQNLLDED***KR***AWKDLKGSW**G*KR***GWNDLQSAW**G*KR***DWGSFKGAW**G*KR***AWNDLQSAW**G*KR***AWDQFRGSW**G*KR***ADALNYDELEDELEDALE***KR***NWSNLRGAW**G*KR***DDEED***KR***SDWSSFRGSW**G*KR***DPGWNNLKGLW**G*KR***SDSNWNRLSPMW**G*KR***SVPSNSQDKPDVTHSEE*

>*Machilis hrabei* (Archaeognatha) MIP [GenBank:GAUM01002707.1, GAUM01002708.1]

***M***QHAVITSALYAITALVLSSFIGASFAEPASNHALQGEPVSEVDSVALGQNLLDED***KR***GWKDLKGSW**G*KR***GWNDLQSAW**G*KR***DWGSYKGAW**G*KR***AWNDLQSAW**G*KR***AWDQFKGSW**G*KR***ADPLNYDELEDELEDALE***KR***NWSNLRGAW**G*KR***DDEEE***KR***SDWSSFRGSW**G*KR***DPGWNNLKGLW**G*KR***SDANWNRLSAMW**G*KR***SVPSNLQDKPGISPSEE*

>*Meinertellus cundinamarcensis* (Archaeognatha) MIP [GenBank:GAUG01032986.1]

***M***QHAVITSALYIVSLYVLLSLVGSALAEGDQSASPVLEAGPLLTSDGNLLNLDQSQLDED***KR***AWKDLQGSW**G*KR***AWNDMQNVW**G*KR***GWHDLQNAW**G*KR***DWGSFKGSW**G*KR***AWDDLQSMW**G*KR***AWNKLGGSW**G*KR***NGYPYDYVDDALEDDMD***KR***NWNNLRGAW**G*KR***DDDE***KR***SDWSSFRGSW**G*KR***SPAWNNLKGLW**G*KR***SDNNWGKLSAVW**G*KR***SVPATAPEKEDGSHSEE*

>*Machilontus* sp. (Archaeognatha) MIP

***M***QHAVITRALYFISFAVLSSLLGIVNADGEPQSNQEPILAADGNLLNLEQNLLDED***KR***AWRDLQSSW**G*KR***GWNDMHNVW**G*KR***GWRDLQSAW**G*KR***DWGSFKGSW**G*KR***AWDNLQSMW**G*KR***AWNKLGGAW**G*KR***SGYPYDYEDDVEDDVD***KR***NWSNLRGAW**G*KR***EDDD***KR***SDWSSLRGTW**G*KR***TPGWNNLKGLW**G*KR***SDTNWGKLSPVW**G*KR***SVAAAAPEKVEATHSDE*

>*Tricholepidion gertschi* (Zygentoma) MIP [GenBank:GASO01254175.1]

***M***QYVVSGSLICLLALLASSSQADPSPDSQHEASPDDSEAVTSGQDD***KR***GWKDLQQGW**G*KR***GWSDLQQPW**G*KR***GWSDLQSSW**G*KR***AWQDLQAAW**G*KR***AWSDLQSPW**G*KR***DWGSFKGAW**G*KR***GWDDLQPLW**G*KR***GWDSFHGSW**G*KR***SDDDTDDVADLDDNELDED***KR***NWSNLRGAW**G*KR***ADDE**G*KR***GDWNNFRGSW**G*KR***EPGWNNLKGLW**G*KR***FDSSNWQRFGAKDQLKVIQE*

>*Thermobia domestica* (Zygentoma) MIP [GenBank:GASN01407677.1]

***M***QHVVTGSLLYLVVVVTYSRGDPSSGEHVASSEAVTSGQED***KR***AWTDLQQGW**G*KR***GWNDLQQAW**G*KR***GWNDLQGAW**G*KR***AWQDLQSAW**G*KR***AWNDLNSAW**G*KR***GWDSFRGSW**G*KR***AWEDLQPMW**G*KR***AWESLHGAW**G*KR***ADDDEDEDILASYLEDSDLAED***KR***NWENLRGAW**G*KR***SDDEDV***KR***DWNSFRGSW**G*KR***EPTWNNLRGLW**G*KR***SDNNWGKLAAVW**G*KR***SVEGETGIKEKSALAAN***KR***RK*

>*Atelura formicaria* (Zygentoma) MIP [GenBank:GAYJ01022660.1, GAYJ01022659.1]

***M***QYVVTGPLLCLLFLVSSSTQSDPSPDHVSSPEEPAVSQTDQED***KR***GWNDLQGGW**G*KR***GWNDLQGGW**G*KR***GWNDLQGGW**G*KR***GWNDLQSGW**G*KR***GWNDLQSAW**G*KR***AWNDLQAGW**G*KR***AWNDLQSSW**G*KR***DWDSFRGGW**G*KR***AWDELQPMW**G*KR***GWDNFHGSW**G*KR***SDDDLDYSLDYNDLLDED***KR***GWNNLRGAW**G*KR***SEDD***KR***GDWNSFRGSW**G*KR***DPGWNNLKGLW**G*KR***SDNWGKLAAVW**G*KR***STEGQTGVKQEDNRQE*

>*Nicoletia phytophila* (Zygentoma) MIP

***M***QYVVPGHLLCLLILVTSSTQGDPAPETGSSQGSSAVATTPDQDN***KR***GWSDLQHSW**G*KR***GWNDLQAAW**G*KR***GWNDLQSAW**G*KR***GWNDLQSAW**G*KR***GWQDLKAASW**G*KR***DWDSFKGAW**G*KR***AWDDLQPMW**G*KR***DWGNFHGSW**G*KR***SENDDDVNLDYLDESLLEED***KR***GWNNLRGAW**G*KR***SDDD**G*KR***SDWSNFRGSW**G*KR***DPGWNNLKGLW**G*KR***AEPGWNKLAAAW**G*KR***SVDGQTGSKPEEVNREE*

..............................................................................................

>*Xibalbanus tulumensis* (Remipedia) MIP

…LAED***KR***GWKDMKSAW**G*KR***GWQDMQAAW**G*KR***AWDDLQTLW**G*KR***AWQDLQSAW**G*KR***ACSDLQSTW**G*KR***AWNDLQSAW**G*KR***AWSDLQNTW…

>*Anaspides tasmaniae* (Malacostraca) MIP

***M***HHALVAAPLLLLVLIVRVTATQDASPEPLTEED***KR***GANWSNLRGSW**G*KR***GSDWSSLLGSW**G*KR***GSDWSSLRGSW**G*KR***EDDTDEGDLQD…

>*Lithobius forficatus* (Chilopoda) MIP

***M***QRILAAAVSSLLVLCLHQAGVLADEGESGAPGAHDPSPDAHASSEAA***KR***AWNDLHGAW**G*KR***GWNDLQGVW**G*KR***APPNLSSVWR***KR***GDWNAFRGSW**G*KR***EGNWNNLKGLW**G*KR***ADGNWNNLKGLW**G*KR***SDGQWNNLKGLW**G*KR***NPLDESDDDLMLDMEALQNEIALQDSK*

>*Hanseniella* sp. (Symphyla) MIP

…***KR***AWRDLGNTW**G*KR***SGNWRELQSVW**G*KR***ANDWRDLQSMW**G*KR***AWEKFQGSW**G*KK***DGGNNWNSFRGSW**G*KR***REPGWNNLKGLW**G*KR***AADGQNWSKLSGYW**G*KR***R…

>*Eudigraphis takakuwai nigricans* (Diplopoda) MIP

***M***ALTSVPVIAAVWCLLSYNAILITAGETADTDQHLRRNQASEAEDESEAALAENANNDDVSELEDEDSDFN***KR***SWNEMRGAW**G*KR***GWNEMPAMW**G*KR***NWNQMPAMW**G*KR***SWNEMPAM…

…RGWDQFNGSW**G*KR***NPLNMAYWNRLRESQE***KR***EPAWNNLKGMW**G*KR***SDDSWTKLNEFSTRSNAEETK*

..............................................................................................

>*Drosophila melanogaster* MIP [[FBpp0075003](http://flybase.org/reports/FBpp0075003.html)]

***M***AHTKTRRTYGFLMVLLILGSACGNLVASGSAGSPPSNEPGGGGLSEQVVLDQLSESDLYGNN***KR***AWQSLQSSW**G*KR***SSSGDVSDPDIYMTGHFVPLVITDGTNTIDWDTFERLASGQSAQQQQQQPLQQQSQSGEDFDDLAGEPDVE***KR***AWKSMNVAW**G*KR***RQAQGWNKFRGAW**G*KR***EPTWNNLKGMW**G*KR***DQWQKLHGGW**G*KR***SQLPSN*

>*Daphnia pulex* MIP [[Dappu1_305794](http://genome.jgi-psf.org/cgi-bin/dispGeneModel?db=Dappu1&tid=305794)]

***M***QFWQCPLLLMVSLIAAINTQQTPSQRLEPNQVAGLVELHQQLEQPREHQQQQQHHQQQPEQPAQADNKDYAPSPAVLLQLTPSDWKNTQD***KR***NNWNRMQGMW**G*KR***SQQQSDESALSEMTPPRQMV***KR***AWSDLSQQGW**G*KR***SWTQLHGVW**G*KR***RWDQLHGAW**G*KR***TPDQLEDDSKAEQPENSQEDEDQQSSSEVEREEANDSDDTVENS***KR***SGWNKMQGVW**G*KR***SSSSSKTNSGPAIEGMGNNDLLLLISGTGDQLYQQREDDQAKANVEDAGNEESVD***KR***GWNQLQGVW**G*KR***ALSAMAAGY***KR***NWNNLRGAW**G*KR***EIPAAIAKGMEWSR***KR***ESGWNNLKGLW**G***

Myosuppressin (MS)

>*Nipponentomon nippon* (Protura) MS

***M***HQSSTLAVFPLLALMSLTAALYLVSGAGASPPPQCAPEYMEELPPRIKKLCAALSSISEFTNGLEHYLDEKVLRENGALLDSGV***KR***QDVDHVFLRF**G*RR***R*

>*Acerentomon* sp. (Protura) MS [GenBank:GAXE01003847.1]

***M***SRSSSQISSFPLLALISLTAALYLVSSQPGASANPPPQCAPEYMDDLPPRLKKLCAALSNISEFTSGLEHYLDEKVLRENGALLDSGV***KR***QDVDHVFLRF**G*RR***R*

>*Filientomon takanawanum* (Protura) MS

***M***SKSSSVSMLSLFALVSLTAALYLVIPGASASPPAQCAPELLDELPPRLKVLCAKLISISELTNGLEHYLDDKVLRENGALLDSGV***KR***QDVDHVFLRF**G*RR***R*

>*Anurida maritima* (Collembola) MS [GenBank:GAUE01000055.1, GAUE01000056.1]

***M***KQPVSVLVLVLVSIGTLLLEEHRVASMAVAVSDMAQQCDPDVLQQVDSPKI***RR***ICNFLQNYAVAMQQLNREGTGPLIYPDGMMENGV***KR***QDVDHVFLRF**G*RR***RR*

>*Tetrodontophora bielanensis* (Collembola) MS [GenBank:GAXI01014890.1]

***M***KQSTMAYFIMAMLAVLLVEQVFSMPAEGMPQCDPEYLQQVDSPKIRKICMFLQNYANAMQNYKEGLLVDNPLGMMENGV***KR***QDVDHVFLRF**G*RR***RR*

>*Podura aquatica* (Collembola) MS

***M***KQNSGIVFLLLASVAMIVLEEQRVSGMTMPDVVQQCDPEILAQTDNPKIRKICSFLQNYAIAMQQYNREGVLYPDMMENGV***KR***QDVDHVFLRF**G*RR***RR*

>*Folsomia candida* (Collembola) MS [GenBank:GASX01084629.1]

***M***LRWTLALLIVGLAAGLAFGMPPQCSPEVLEEIDNPKIREICHFLQTYAEAVEGGAAFVKGGSHYPLGLIDNGV***KR***QDVDHVFLRF**G*RR***RR*

>*Bourletiella hortensis* (Collembola) MS

***M***KTTVGILLVAAIAAIVVKEVYSMPPQCSPEMLDEVENKDIRKLCEFLTSYTAAIEQYNRGRGLDRPPYNQLIDNIDR***KR***QDVDHVFLRF**G*RR***R*

>*Orchesella cincta* (Collembola) MS

***M***KSTVGIFVVAMLAAFAIKAAHSMPPQCSPDALEDTDNPKIRQICEFLRNYAAAVEEYQKGMTGGPHFPFGLIDNGV***KR***QDVDHVFLRF**G*RR***R*

>*Pogonognathellus sp.* (Collembola) MS [GenBank:GATD01015193.1]

***M***KSTMGILIVAMLAAFAVKQAYCMPTHCDSDALDEVDNPKIRQLCELLQSYAAAVEHQYRQSGAPSGLIDNGV***KR***QDVDHVFLRF**G*RR***R*

>*Sminthurus viridis* (Collembola) MS [GenBank:GATZ01100909.1]

***M***KTTLGVLLVAAITALVVKEVYSMPPQCSPEVLEEIENKDIRKLCEFLATYTAAVEQYNRGRLTREPYPGLIENGV***KR***QDVDHVFLRF**G*RR***R*

>*Jordanathrix leptothrix* (Collembola) MS

***M***KSTVGILLIATLAAVVVTEAFSMPPQCSPEMLDEVENKDIRKLCDFLTTYTAAVEQYNRDRLARANYAGLIDTGV***KR***QDVDHVFLRF**G*RR***R*

>*Campodea augens* (Diplura) MS [GenBank:GAYN01124783.1]

…MAVPPPQCNPELQEELPPKV***RR***LCLALQTLQELSNTMEHYLDDRALRENGPLVDTGV***KR***QDVDHVFLRF**G*RR***K*

>*Lepidocampa weberi* (Diplura) MS

***M***NSCALLFLVTLSSSLLVQVFATPICNPELQDEVSPKVKKLCLALATLQELSNTMEHYLDDRAIRENGGALVDSGV***KR***QDVDHVFLRF**G*RR***K*

>*Occasjapyx japonicus* (Diplura) MS [GenBank:GAXJ01011331.1]

***M***TTAKMSSGRWTTALLAASLLLSAWLTSPEAGGRGLAGASALPPPTVHCSPEILDELPPKLRKICDALATLNEFSTNVEQYLDDKAYRDNAPRMDSGV***KR***QDVDHVFLRF**G*RR***R*

>*Catajapyx aquilonaris* (Diplura) MS

***M***VFGRAMTAILVTCSLVLALWLASGQASPMPAVQHCNPDMLEELPPKLRKICEALATLNEFQSNVEQYVDEKAYRDNVPLMDSGV***KR***QDVDHVFLRF**G*RR***R*

>*Pedetontus okajimae* (Archaeognatha) MS

***M***MRSETLLALLASFVILLLTSSHQSTAAASPPTCTTDIEELPPRFRKICAALATIAEFSSAMEQYLDDKVLRENAPQLLDNGV***KR***QDVDHVFLRF**G*RR***R*

>*Machilis hrabei* (Archaeognatha) MS [GenBank:GAUM01020537.1]

***M***RSETLMALFSAFVILLLTSSQQSVVAASPPPPCTTDLAEELPPRFRKICAALATIAEFSTAMEQYLDDKVIRENVPLLDNGV***KR***QDVDHVFLRF**G*RR***R*

>*Meinertellus cundinamarcensis* (Archaeognatha) MS [GenBank:GAUG01217533.1]

***M***RPETLALHLATLLALLVTSQQSAVSSPPCNTDAMEDLPPRFRKICAALATIAEFSSAMEQYLDDKVLRENVPLLDNGV***KR***QDVDHVFLRF**G*RR***R*

>*Machilontus* sp. (Archaeognatha) MS

***M***RAESLVIVCAAFVALLVTSQQASVVASAAAVVSPPPCTADLVDDLPPRFRKICAALATIAEFSSAMEQYLDDKVLRENVPLLDNGV***KR***QDVDHVFLRF**G*RR***R*

>*Tricholepidion gertschi* (Zygentoma) MS [GenBank:GASO01223859.1]

***M***KSVYLFVLLSAILTLLVTSRSVSAVPPPQCSPEFVDELPPRVKKICAALATIAEFSSAMEQYLDDRVLRENTPLVDNGV***KR***QDVDHVFLRF**G*RR***R*

>*Thermobia domestica* (Zygentoma) MS [GenBank:GASN01405381.1]

***M***KSSICLVSLVSVLLALLMTSQLVIAVPPPQCNPEFLEELPPRVKKICAALATIAEFSSAMEQYLDDKVMRENAPLVDNGV***KR***QDVDHVFLRF**G*RR***R*

>*Atelura formicaria* (Zygentoma) MS [GenBank:GAYJ01000236.1]

***M***RSIYLVSLLLALVTLLVTSQGASAVPPPQCNPEFVEELPPRVRKICAALEKIAEFSSAMEQYLDDKVLRENTPLVDSGV***KR***QDVDHVFLRF**G*RR***R*

>*Nicoletia phytophila* (Zygentoma) MS

***M***RSVYFTSLLLVLVTMLVTSLRTTSAAPPPQCGAEFVDDLPPRVRKICAALEKIAEFSSAMEQYLDDKVLRENTPLVDSGV***KR***QDVDHVFLRF**G*RR***R*

..............................................................................................

>*Xibalbanus tulumensis* (Remipedia) MS

***M***KSSLMLTLAAIFMSLVFVVSHTTAVPPPQCNPELLDDLPPRLRKICTALATIAEFSNAMEQYLDDKVIKENMPLMENGV***KR***QDVDHVFLRF**G*RR***R*

>*Lithobius forficatus* (Chilopoda) MS

***M***AQLRYRSVSALFCILLVAITRHVTSLPPPQCESDEPLPPRLEKVCSALRVISEYTHLLEDYWDEQVLSSLAANDV***KR***EEPGMSHVFLRF**G*RR***R*

>*Hanseniella* sp. (Symphyla) MS

***M***PVRLHRLVQVYMFVIVILLLDAQGFSHPQCTGNLQDLAPTQHKMCTALKAISELADMMEQYLDDGVARSFDRGYTL***KR***DEAGFNHVFLRF**G*KR***RRR*

>*Eudigraphis takakuwai nigricans* (Diplopoda) MS

***M***ASRVTIVAVVGLMVVASVSFKLIDCSPVPVPICDSASLQDLTKEEQIACEMLRDLVMNRFMTKPEDDDMNANAFPVEHELPYKLAQQNSPNF***R***SEPSLQHAFLRF**G*RR***R*

..............................................................................................

>*Drosophila melanogaster* MS [[FBpp0083991](http://flybase.org/reports/FBpp0083991.html)]

***M***SFAQFFVACCLAIVLLAVSNTRAAVQGPPLCQSGIVEEMPPHIRKVCQALENSDQLTSALKSYINNEASALVANSDDLLKNYN***KR***TDVDHVFLRF**G*KR***R*

>*Daphnia pulex* MS [[Dappu1_443203](http://genome.jgi.doe.gov/cgi-bin/dispGeneModel?db=Dappu1&tid=443204)]

***M***PATRGNHHTAATMAVAAVLTFVVLVVVGAPSFAEAGSIPPPHCNPDVSDALPPRLRRICAALYGIAEISNAVEQYLDDKSNATMRDPSMTDSDRGV***KR***QDVDHVFLRF**G*RR****

Natalisin (WAARamide)

>*Nipponentomon nippon* (Protura) Natalisin

…NLQRSGE***KR***EIRKSDSGETDEAAFWAAR**G*KK***APRGGTDLTSRE***KR***ALEETEDALDSDYYGNQLEDALTFRDLLRMRVARDGVMEPGQ***R***NLVDPFWAAR**G*KR***DQVDDSFWAAR**G*KR***EAPGRA***KR***STANEQRWLQGRSDPFWAAR**G*KK***DASGEEDGFWAAR**G*KR***FLMRHPPVVHRSDGGPFWAAR**G*KR***TDSNVPH…

>*Acerentomon* sp. (Protura) natalisin [GenBank:GAXE01015024.1, GAXE01015023.1]

***M***VNLTPECRTVGVVVVSMVVTLTMYVAADKVSFNEQAEQSGLSHSKQFLQKEPEYSEENHNLPLTNSNRQTNF***RR***PADSKTLMY***KR***GSSEEEDAAFWAAR**G*KK***GSIQPWLDKPGR**G*KR***DTELTPGDDDVFDDDDTLESGGATLRDLLRMRITRDGMDGSMHQSNLVDPFWAAR**G*KR***GPTDELFWAAR**G*KR***STTSEEAPFWAAR**G*KR***PH***KR***SLRPRQPELFLQQRSADPFWAAR**G*KR***SAEDERIFWAAR**G*KK***MAVPV***RR***AGPFWAERAALDMSDFRKLPW*

>*Filientomon takanawanum* (Protura) natalisin

***M***IQLSSEFRTIVFVTFAVHLILKATADKVSFNDRLGQTGLSQPRSPQQRDFEYSDESANHNTPTSNTQQTNY***RR***QTKEPMQ***KR***EKSSSSEEQDPAFWAAR**G*KK***ESSGLRGSKAGR**G*KR***DLELMPGDDDVFDDDDAIANGAALRNLLRMRVSRDGVESIHQSNLVDGPFWAAR**G*KR***DPADDLFWAAR**G*KR***STSEEAPFWAAR**G*KR***LLP***KR***EFLQQRTDPFWAAR**G*KR***GEEGVFWAAR**G*KK***MAVPV***RR***AGPFWAERASMDMGDLPRLPW*

>*Bourletiella hortensis* (Collembola) natalisin

***M***AATGLRVYVILFICASVFSSVFCNNN***RR***SESGKGGNAETPFWAAR**G*KR***GDLNGGVIRCGIVKPGVFEAESDLPSSWVDIESGNDGLDEIATRL***KR***SSGLVHSKGDTVLEPLPLWLSRASLELDRPGYQQFFYPSSHSNERILSLWKQLAE***KR***TERSGNEKAGNGESAPFWAAR**G***

>*Sminthurus viridis* (Collembola) natalisin [GenBank:GATZ01098377.1]

***M***KGLVLLAIALTICSVYSESS***RR***SENTPPDGAKERDTPFWAPR**G*KK***SHTMAQSIRCGLLRPFEETHEEPSWTEMDSSERFYDKNVDLWSHRF***KR***SSDLSGPPSLDNEL***RR***RLWLLKSYMEPSSQMESDKNLNLLTSWLKLVE***KR***KAGNGNSGNGNTN…

>*Jordanathrix leptothrix* (Collembola) natalisin

***M***NPCRILFLLISLILFGLVSSTQ***RR***SENNRQSNTGSSKDQTSDTPFWAAR**G*KR***ERNELSGLIKYELIRPPPLPLFGDPEDAAEDPASSSSPDFSTIPVNEGNPVRPEPLHPWSYKT***KR***NSLLPAYPGENSEELNHGDELMPGWLAQASKDHLNGGVIGRG…

…FTALLGLAGAERA***KK***NAEKNSGRGGNVGGGAGAGDTPFWAAR**G***

>*Campodea augens* (Diplura) natalisin [GenBank:GAYN01120357.1]

…***RR***LAQALGIL***KR***QGRGLNLLRISSIRSPDNFVAQR**G*KR***LRDTSGNWNPEWTSDNYGTNDVFWPAR**G*KK***QNEFNPNEEFEEEPQEDRQERGGFSFL***RR***ENDPFWAAR**G*KK***…

>*Lepidocampa weberi* (Diplura) natalisin1

***M***GFRRSILSLHFFSQYALFYLTFIFLHSTVESFPYNQPDDTYLISSDISQMPADESEDLVASSTTHPLRP***KR***QTELNARLARVLNLLHRQSRGNSRSTQSPENFVSQR**G*KR***LPDGGRDTFWPSR**G*KK***DFQQEEGEDLWRIPFWITDAGGNKDPLLVWPAR**G*R***TYDLDDVVELLSAASFP*

>*Lepidocampa weberi* (Diplura) natalisin2

…***KR***LPDMPTDGARVNDLFWPSR**G*KK***QWDSQEEEEQEIQDS**G*R***KDQEPFWITQGNKGPVLVWPAR**G*R***MYDLHDVVELLSATSLS*

>*Occasjapyx japonicus* (Diplura) natalisin [GenBank:GAXJ01089881.1]

…DQWSSHPKTIWASEP***KR***GSGDDPFWAAR**G*KK***TLEEPQRQREGDGRE***KKR***PPIDLLFQTDPFWAAR**G*KK***APPLFLQGNTYSSSTDPFWAAR**G*KR***WLFLPP***KR***FPAINLAAP…

>*Pedetontus okajimae* (Archaeognatha) natalisin

…TEPNFWATR**G*KR***ESKVAEPPFWATR**G*KR***ESKADEPTFWATR**G*KR***ESKEDDEPTFWGAR**G*KR***ESEEAEPPFWATR**G*KR***ESNE…

>*Machilis hrabei* (Archaeognatha) natalisin [GenBank:GAUM01118562.1 + GAUM01082750.1]

…AR**G*KR***QSEEVEPTFWASR**G*KR***QSEETDPSFWAAR**G*KR***QSDEAEPTFWAAR**G*KR***QSEEAEPTFWAAR**G*KR***QSEEAEPTFWAAR**G**…

…RGIKEDFTPFWKTQNKSESSNTVTGEKQESKENREESDPHFWAV***RRKR***ES***KK***VYPTFWAAR**G*KR***ESKDEPTFWAAR**G*KR***Q…

>*Meinertellus cundinamarcensis* (Archaeognatha) natalisin [GenBank:GAUG01195620.1 + GAUG01051047.1 + GAUG01214136.1]

…FWAAR**G*KR***NSEESDPTFWAAR**G*KR***NSEESDPTFWAAR**G*KR***NSEETNPTFWAAR**G*KR***NSNELNTKIRAKSENKETTTWKQKQKTELPDKNIKAD…

…LGTNSLKE***KK***DIERIFDPHWTA***RRKR***ESHEDVSTFWATR**G*KR***QSEEISDPGFWASR**G*KR***NSEESEPTFWAA…

…MNYNFWATP**G*KR***FISKKHILDYIFWSSR**G*KR***PTSRDDLLHDHFWATR**G*R***SILPDLYDLDLEDQNFNQCKANQLSSNNVFDDQMR***KKR***QVENKPHTKTPWNAFLAAINKYNRDIRHD…

>*Machilontus* sp. (Archaeognatha) natalisin

…**G*KR***PTSSQAETLNDNVWAMRVNRQSTSKEDILNDNFWAPR**G*KR***PFSKEDILNYNFWSSR**G*KR***PRTKNNIFDNFWAAR**G*R***SLPQDEDDFDVDEGVDAVPLNLEM…

…FEDQSDEEQNPPNP***KR***NGVLQKLLKSKLSFRGSAPVSD***RR***ESLEDEPGFMESEKPFWAAR**G*KR***GDIDTLLNNLYAREGSFRDIR…

>*Tricholepidion gertschi* (Zygentoma) natalisin [GenBank:GASO01205242.1 + GASO01101846.1]

…SDDPLGPFWAAR**G*R***SIDDDSLGPFWAAR**G*R***SITDPHNSFSATRERGTDDSLGPFWASR**G*R***ALDETV***KR***ADSDITSEKATVNSAESGPFWASR**G*KR***QQTPNMNAAEA…

…PFWAVR**G*KR***TLLRDLEMATKDPFWPVR**G*KR***ETKADEDDEVNDDDHKFWTTMEEKSASRSGNDSKTDESHRM***RR***SPEYEEE…

>*Thermobia domestica* (Zygentoma) natalisin [GenBank:GASN01347902.1 + GASN01118969.1]

…AR**G*RR***LVNNDQHLTNRANYEEEPPFWAMR**G*RR***ISGNQPSVFIHDDGPFWAAR**G*RR***TEEEEPFWAAR**G*RR***TEDEEPFWAAR**G*R***VDDDEPFWAAR**G*RR***TEDEEPFWAAR**G*R***S…

…**G*R***SINTDGPGLSHMKGDDDDEGPFWATR**G*RR***TKPLLSSQGGNGLMYGLEDPRHKSEIIFSILANGREQEKAGKTAH…

>*Atelura formicaria* (Zygentoma) natalisin [GenBank:GAYJ01308988.1 + GAYJ01311217.1]

***M***TPSQGSRMMTEAAALFLLLAARCVTALDPANQQTIGDAGHTGKTAARNDSFPLDYGEFSEQFSRFMGWPLPETQDDKDAARA***RR***TDMRAVLKDGGDPEFWPVR**G*RR***GDNTNSASQEDPFWAAR**G*R***SVTSADQYSPFGTVTGQVLKQPVPIGHIGRVTNVQHNHTPEHSSMGKNT…

…ADTEEPFWAAR**G*R***NVETEEPFWAAR**G*R***SANEEEPFWAAR**G*R***STGIVDHDKSFKGAQGATSNFIKNPQRVVLSQQSTTQNEVTTFHKHGRETGDSTSHLEPLWVSRARSAKHHPYRSLIFLQFTPKFSSTERLKSTIDEKTPLWNTNGN***KR***NSKQVANART***KR***INADQPRGTASDLCGNTQSACRGTSSSTDSSEARGTNTIDPAG…

>*Nicoletia phytophila* (Zygentoma) natalisin

…GLPGPSEPFWAAR**G*R***SAGHGEPSEPFWAAR**G*R***STGQDESSGPFWAAR**G*R***STTLDEPSGPFWAAR**G*R***STALDESSG…

…VDQETNQPAPDSKLEQPTLSTGSQVSRTHQGSGSHQGFQEPSQTAGY***R***SSSQESPADLFWASH**G*R***SSGPQDVSDLFWAAR**G*R***SSGHDEPSEPFWAAR**G*R***STALDESE…

…SNLREMENNTSPASGY***RRKR***SNHCDKDKCKKDYDVTDLLRSILEQNNRLDSNLLQTDADSVLAHQVQPPEPDDNNLHWTED**G*KR***TVVRGLGDLVKDPFWAAR**G*KR***NNNQKEESFWTHATNNMSGSEKLKSGTNDSEDLLSTLRSIIHVMDELQVSTKW***KK***SSDRSRNLMSNLLLHPDAAQE***KK***NAGRLSILQDILPGPRQEFWAAR**G*K****

..............................................................................................

>*Drosophila melanogaster* natalisin PC [[FBpp0111541](http://flybase.org/reports/FBpp0111541.html)]

***M***RLTLAWLSLCLAIYCGGGHGHGNVVLSLPPSLIATATKAALSHQRQQKQQQQHQKKDARVLFDSPADALRDMMHNGNGNGNGPMDSGKFSLSDVEQPAAQRSEDFNRNAYDLGARQSAPQEIAMGMELGMGLGLGPNNYRTTPPHRYWGQRCQGRSGGSGTSKCPQEYYRTMLAARNKEALSRLHMQLSSMQDSDSGASSSSDSEEEHVDDEEQSNNEVFMLLTGEQDLMKFLHWAMQVLYPIERPLGNLSDGAAENYYPGMFLWKKLNLSGHLEPPLIVDEPQYVLV***RR***EKLFDGYQFGDMSKENDPFIPPR**G*RK***HSGSLDLDALMNRYEPFVPNR**G*KR***DKVKDLFKYDDLFYPHR**G*KK***HRNLFQVDDPFFATR**G*KK***LQLRDLYNADDPFVPNR**G*KR***HLTASAGKLGETMAGGGKWPDDSNNYWPLRMSTHKINGYDQSVRPSLSVEDAAASLASWRLPANRLHSTRSMSADLRQQLLLPHVRFIGNPNMRQQQQQQQQHQVKTSSWQAEERLRRSILAPGESNDAHETQLTLSHPANPHLVTDTDNLNI*

>*Drosophila melanogaster* natalisin PD [[FBpp0290303](http://flybase.org/reports/FBpp0290303.html)]

***M***RLTLAWLSLCLAIYCGGGHGHGNVVLSLPPSLIATATKAALSHQRQQKQQQQHQKKDARVLFDSPADALRDMMHNGNGNGNGPMDSGKFSLSDVEQPAAQRSEDFNRNAYDLGARQSAPQEIAMGMELGMGLGLGPNNYRTTPPHRYWGQRCQGRSGGSGTSKCPQEYYRTMLAARNKEALSRLHMQLSSMQDSDSGASSSSDSEEEHVDDEEQSNNEVFMLLTGEQDLMKFLHWAMQVLYPIERPLGNLSDGAAENYYPGMFLWKKLNLSGHLEPPLIVDEPQYVLV***RR***EKLFDGYQFGEDMSKENDPFIPPR**G*RK***HSGSLDLDALMNRYEPFVPNR**G*KR***DKVKDLFKYDDLFYPHR**G*KK***HRNLFQVDDPFFATR**G*KK***LQLRDLYNADDPFVPNR**G*KR***HLTASAGKLGETMAGGGKWPDDSNNYWPLRMSTHKINGYDQSVRPSLSVEDAAASLASWRLPANRLHSTRSMSADLRQQLLLPHVRFIGNPNMRQQQQQQQQHQVKTSSWQAEERLRRSILAPGESNDAHETQLTLSHPANPHLVTDTDNLNI*

>*Daphnia pulex* natalisin [[Dappu1_97675](http://genome.jgi-psf.org/cgi-bin/dispGeneModel?db=Dappu1&id=97675), GenBank:[EFX86577.1](http://www.ncbi.nlm.nih.gov/protein/321475615?report=genbank&log$=prottop&blast_rank=1&RID=XVKJCM1W014)]

***M***ELIKIIFVLASGWATLAIAGNTDQDMFWAAR**G*KK***ASIEGNWPDSVVMEPFVAAVYD***KR***DGTFWAAR**G*KK***YAADGGDGVPFWATR**G*KK***GDLEIPFWAAR**G*KR***IPQSEMNETEEEGNRRE***KR***SAGRDTSIIHSGQRFRNRPARPASQAAEPFWAAR**G*KK***NSNVSKFPLL*

(long) Neuropeptide F (NPF)

>*Nipponentomon nippon* (Protura) NPF1

***M***RTPALTSASCCLFLSLTILLIATQLPCGQSRPEPAQLTQMADALKYLQELDKYYSHMSRPRF**G*KR***DGIPMARDMNKIDAVQRILEAMNE***KKK****

>*Nipponentomon nippon* (Protura) NPF2

***M***PLVSNSMLLAVLATVVVKSMASSGDNPSHSSALMENPSMPLDPNTVAAARPSRPKVFTSPDELRQYLDALSNYYAIAGRPRF**G*KR***GSLRAASPSKQQYQPIWMVGGDSPPRVNSGILKTDPFRSEQDYDD*

>*Nipponentomon nippon* (Protura) NPF3

***M***PVASNSLLLAFLATAFVNSMANSGADQPAAMLEGAPGPAGMQLDPNAVAAQRPSRPKVFTSPDELRHYLDALSNYYAIAGRPRF**G*KR***TSSSLRSVSNGQAYQPLWLIPDQVFVKPKPVDPYFNMAADYDSTT*

>*Acerentomon* sp. (Protura) NPF1 [GenBank:GAXE01006614.1]

***M***GTGSTHTTSLLLAVSLSLVIIANIIQSSEGRPEPAQLTQMADALKYLQELDKYYSHMSRPRF**G*KR***DGMPITKDLNRIEAVQRFLDAMNEQQQK*

>*Acerentomon* sp. (Protura) NPF2 [GenBank:GAXE01137093.1]

***M***PLVSNSMLLAVLATVVVNSIATSPVDGPKQSSPMLENPQMIEFGSNLAAAARPSRPKVFTTPEELREYLDALSNYYAIAGRPRF**G*KR***ASLRPSKQILQPVWMVPEPSSLFKQDSYRSQQQEYED*

>*Filientomon takanawanum* (Protura) NPF1

QWCPTPLLLVATLVILATVLPAGQGRPEPAQLTQMADALKYLQELDKYYSHMSRPRF**G*KR***DGIPLAKDLSKIEAVQRILEAMNEQPQK*

>*Filientomon takanawanum* (Protura) NPF2

***M***PLVSNSMLLAVLATVVVKSMASGDTSNNPSGLLDNQSMVLDPNTVAAARPSRPKVFTSPDELRQYLDALSNYYAIAGRPRF**G*KR***GSFRGAFPAKQQYQPIWMADAEHQQRASSGILKTDSFRHEQEYDD*

>*Anurida maritima* (Collembola) NPF1 [GenBank:GAUE01008465.1]

***M***SYGARFLGSVSVLTTILWFIFFITRNEASGASVYYSPKALGVSNYDQLNSLFKDYLKVFEKLKTGKPKHISGQVNLRPARSELVGDMAHALRLYQLAELDRMYGAKSRPRF**G*KR***AEVTQEIEPAFTAGYDSDSVNLDVPERTFASHTYEDRSR*

>*Anurida maritima* (Collembola) NPF2 [GenBank:GAUE01009857.1]

***M***GQRVRVGIWMMVVVSVVLYLKNAAVTSGEPISSMEIEKLEKPQRPRVFTSPEELKSYLGALAKYYATMSRNRF**G*KR***SLRIESRMSDFLAPQHAYDLLQNNNDK*

>*Tetrodontophora bielanensis* (Collembola) NPF1 [GenBank:GAXI01145608.1]

…LPTTIALFAIFLICLTGNSNGTPVMYSVSDPAYDQLAALIKFMEKVKTQTQPKQISGQVSLRSPRSDFVGDMAHQFRLINQLQELDRMYGSKSRPRF**G*KR***NGMNQNDPSIYGYYDAINNANLIDEDGS***RR****

>*Tetrodontophora bielanensis* (Collembola) NPF3 [GenBank:GAXI01137106.1]

…LLTLLREYIKLIERVQNQHKHISGQMSSRTPRSDLIGELSNSQRLHQLQELDRMYGAKSRPRF**G*KR***IWLNQDEGQVFGYNDAANNINIPVTNEGDQN***RR****

>*Podura aquatica* (Collembola) NPF1

***M***SKLILQLVLGLVLIYAHLISNVKGNYFSSGDELGLFKNFLRTMGNN***RR***ISQPKIITGQLNTRAARSEVGEMANALRLYQLQELDRMYGQKSRPRF**G*KR***SGEDLPNYKSGYDTSSSVHKN*

>*Podura aquatica* (Collembola) NPF2

***M***GSGLGIRISIFFALISTIIFFLATKVNCETLSSLEIDKLEKPQRPRVFTSPDELKAYLGALHRYYTSVSRNRF**G*KR***IPLPIQRGSNLLQTFTPNEEFEFMRIEK*

>*Folsomia candida* (Collembola) NPF2 [GenBank:GASX01087014.1]

***M***HCMTAWVLVIVVGSAILVAANGPPPTELLTEGDIPMLTSSMEMEGKPPRPRLFQSPEQIRMYLDSLGKYYNYQNRNRF**G*KR***GSGPDPYLQFKDPYEIKGVPEYDFRSPL*

>*Bourletiella hortensis* (Collembola) NPF1

***M***MWKFGSTIAIFAVALWFSVGKSSCDAAPASADVTYTDYNHLNAMVREVLQLLEKVKSQPHHEKLIGQVNSRSPRSGDLVGDMAHALRLYQLQELDRMYGSKARPRF**G*KR***AEVTLDNVPTYGYEGGFNGISMED***RRRR****

>*Bourletiella hortensis* (Collembola) NPF2

***M***GSFGFWLIVVLGVVYLQNGSSVKGEALTSMEAEARPQRPRVFTSPDELRNYLEALSKYYALAGRPRF**G*KR***AHIKDLSAANSMPPSLSLLDVYDALQNDK*

>*Orchesella cincta* (Collembola) NPF1

***M***MTWSKVVSSHPSPHYSLVTFALVLVLFVATLPSSSHCLPLGSSQDVSSVDFAQLQSLVREALKYMQRYNRDEPTRISGQVNVRYPRSELGDVALRLYNLQEMDRKYGAKAR*PR*F**G*KR***NEVTQDNFIPMYGDEDVKDSTIGANGESPPPVSSPLFFIP*

>*Orchesella cincta* (Collembola) NPF2

***M***TSVGLWLVLFLGFVTIERIYGGGVPPDVQLGSMEAPAFPERPVLFTSPSEVKHYLDSL**G*KK***ISFSSRQRY**G*KR***AILQVLRDANSARLEQPSDSQDECNCERLRK*

>*Pogonognathellus sp.* (Collembola) NPF2 [GenBank:GATD01088638.1]

***M***LLLGMVCLENSNMVAGDALSSMEAEALTNKPMRPKIFSTADELRNYLDQMSKYYALAGRSRY**G*KR***GLSKNASPKRSFSNNSPAGSIDIYDLIQQDK*

>*Sminthurus viridis* (Collembola) NPF1 [GenBank:GATZ01102783.1]

***M***IWKFSSGGVVAIVTVAVWFSFGNSCEAFHSSVDLNNYDYNQLTAMVRDALRYLEKVKSQPRYDKLTGQVNSRSQRSELVGDMAHALRLYQLQELDRMYGSKARPRF**G*KR***AEVTLENVPTYGFEGNFNGGVSVED***RRKR****

>*Sminthurus viridis* (Collembola) NPF2 [GenBank:GATZ01104831.1]

***M***GSFGFWLIVVLGVIYLQSESSVRGEALSSMEAEARPQRPRVFTSPDELRNYLEALSKYYALAGRPRF**G*KR***VITKNASPVNSVSNLSILDVYDALHDDK*

>*Jordanathrix leptothrix* (Collembola) NPF1

***M***MWKFSSGGFLAVIAVMVWLSMGKPCDAFHPSVDLNNYDYNQLSNMVKDALRYLEKVKTQPRIEKLTGQLNTRPPRSDVVGDMAHAIRLYQLQELDRMYGSKARPRF**G*KR***AEISLENVPTYGYEDNSNVGDD***RRRR****

>*Jordanathrix leptothrix* (Collembola) NPF2

***M***GSFGFWLIVILGVIYLQSESSVRGEALTSMEAEARPQRPRVFTSPDELRNYLEALSKYYALAGRPRF**G*KR***ALTKDVSPVNSMSNLSLLDVYDVLQNDK*

>*Campodea augens* (Diplura) NPF1 [GenBank:GAYN01133742.1]

***M***RIMLVVSFAVLLVLSEVHKSVGRPDPGNQENMAAVADALKYLKELDKYYSQVARPRF**G*KR***METLDDDDAGVLPLDPLSRFPHHLSGQ***KRRR****

>*Campodea augens* (Diplura) NPF2 [GenBank:GAYN01140478.1]

***M***SCTTLRLLVATALVLVVLSVTTSCVAEPPLSGESESRPSRPKVFTSPDELRTYLEALSNYYAIAGRPRF**G*KR***SQSSHSVKFPSHLFPLTSVDLYDLLQYDE*

>*Lepidocampa weberi* (Diplura) NPF1

***M***SYLAGLSPFLVGVSFLFLMTHFHHAAEGNPAPTSQESVISIADALRYLRELNAHITKPSIRTSSANQASVDKVANALRLLHLQELEKYYAQRAKPRF**G*KR***TETTGDSNLPEVLPMETY***RR***LAESFMDQGLV*

>*Lepidocampa weberi* (Diplura) NPF2

***M***AKSTIRLLVVAGLALVVLAFSSLCGAEPPMSGEAEARPSRPKVFTSPDELRTYLEALSNYYAIAGRPRF**G*KR***SQVSSHAGRIPSHLYPLSSSDLYDLLQYED*

>*Occasjapyx japonicus* (Diplura) NPF1 [GenBank:GAXJ01005164.1]

***M***MARCWFLLTLVAALVVTQQGGVTEARPDPGQLAVLADAFKQLHDLDKTYSKITRPRF**G*KR***MEMAPYPPDEAVAEAGERLF**G*R***FGGEQAED***RR****

>*Occasjapyx japonicus* (Diplura) NPF2 [GenBank:GAXJ01006470.1]

***M***SLSSLSLLLAVGVGLGLVTLIVPSAADSGLSVESESRPSRPKVFTSPEELRTYLEALSNYYAIAGRPRF**G*KR***TGSRPSISNSRVPNNALPLLSSSNLYDLLQYDE*

>*Catajapyx aquilonaris* (Diplura) NPF1

***M***ARCWFLLVLVVALSLAQQVNMAVALPDPGQLAAYADAYKQLKELDKYYSQVARPRF**G*KR***MEMPPYPPEEALAEAGERLFGHLGGGQPEA***RR****

>*Catajapyx aquilonaris* (Diplura) NPF2

***M***SLSSLSMLLVVVVGLGLVTLIVPSLADPTLSVESESRPSRPKVFTSPEELRTYLEALSNYYAIAGRPRF**G*KR***TAARPNKVPSYVLPLSSSNLYDLLQYDE*

>*Pedetontus okajimae* (Archaeognatha) NPF1

***M***VAAMTARIALATLCLLVIASQFSPIVAKPDPGQLAAMADALKYLQELDKYYSQVARPRF**G*KR***MDYGSNADAQESNMGETSDKLWGHLT***RRR****

>*Pedetontus okajimae* (Archaeognatha) NPF2

***M***GTSSVCLLLFLFVIGLALPASCEEPLNPENVSRPSRPKVFTSPDELRTYLEALSNYYAIAGRPRF**G*KR***VSLKPRDAYNYRYPLSTSDIYDLLLQFDE*

>*Pedetontus okajimae* (Archaeognatha) NPF3

***M***TAAMTARITFITLCLVINESLFSPIVAKEDPSKLEYLQEIVDYISKFNHTTPRSDSPKIDISGMKKALKYLQLQELDRLYSQRSRPRF**G*KR***MDYG…

>*Machilis hrabei* (Archaeognatha) NPF1 a [GenBank:GAUM01013146.1]

***M***VAAMAVRIALATLCLLVVASQLSPVVAKPDPGQLAAMADALKYLQELDKYYSQVARPRF**G*KR***MDYGSTSDAQESNMADPADKVWGHLT***RRR****

>*Machilis hrabei* (Archaeognatha) NPF1 b (reconst. from GAUM01013146.1, GAUM01013145.1]

***M***VAAMAVRIALATLCLLVVASQLSPVVAKPDPGQLAAMADALKYLQELDKYYSQVARPSPRSDSPKIDITGM***KK***ALKYLQLQELDRLYSQRSRPRF**G*KR***MDYGSTSDAQESNMADPADKVWGHLT***RRR****

>*Machilis hrabei* (Archaeognatha) NPF2 [GenBank:GAUM01151628.1]

***M***GTSSVCLVVILFVIGLTLPASCEELGNPENVSGPSRPKVFTSPDELRTYLEALSNYYAIAGRPRF**G*KR***VSIKPKDAYNYRYP…

>*Meinertellus cundinamarcensis* (Archaeognatha) NPF1 a [GenBank:GAUG01009860.1]

***M***VAGMSTRIALATLCLLVVASQLSPIVAKPDAGQLAAMADALKYLQKIDKYYSQVARPRF**G*KR***MDFGSDSNGDAQESSMADSSDKLWAHLN***RRR****

>*Meinertellus cundinamarcensis* (Archaeognatha) NPF1 b (reconst. from GAUG01009859.1, GAUG01009860.1]

***M***VAGMSTRIALATLCLLVVASQLSPIVAKPDAGQLAAMADALKYLQKIDKYYSQVARPSPRSEPGKLDISGI***KK***ALTFLHLQELDRLYAKSRPRF**G*KR***MDFGSDSNGDAQESSMADSSDKLWAHLN***RRR****

>*Meinertellus cundinamarcensis* (Archaeognatha) NPF2 [GenBank:GAUG01245158.1]

***M***GSSSILILFVLFLIGLAIPVLCEDALSPENASRPARPKVFTSPDELRTYLEALSNYYAIAGRPRF**G*KR***VALKPQDVSAYRYPLSTSDIYELLLQYDE*

>*Machilontus* sp. (Archaeognatha) NPF1

***M***VATTSTRITLAIFCLLVVASQFNPIVAKPEAGQLAAMADVLKYLQELDKYYSQVARPRF**G*KR***MDYGMTGDAQESMPDSSDKLWAHLT***RRR****

>*Machilontus* sp. (Archaeognatha) NPF2

***M***GSSGLLILVVVFLIGLALPTLCDDSLPENASRPSRPKVFTSPDELRTYLEALSNYYAIAGRPRF**G*KR***APMKPKDAYNYRYPLSTSDIYDLLLQDEE*

>*Tricholepidion gertschi* (Zygentoma) NPF1 a [GenBank:GASO01010534.1]

***M***QRNGRFWVVMAAVCLVAIASQLAPVAAKPDQGQLAAMADALKYLQELDKYYSQVARPSPRSESYRQNDLSRVEIALKLLRLQVLDRIHSNKTRPRF**G*KR***MEMQPITDDGAASDAAEKLWGHLS***RRR****

>*Tricholepidion gertschi* (Zygentoma) NPF1 b [GenBank:GASO01010533.1]

***M***QRNGRFWVVMAAVCLVAIASQLAPVAAKPDQGQLAAMADALKYLQELDKYYSQVARPRF**G*KR***MEMQPITDDGAASDAAEKLWGHLS***RRR****

>*Tricholepidion gertschi* (Zygentoma) NPF2 [GenBank:GASO01021206.1]

***M***GSPSILVMVCLCVIGMSMPCCCDPPVSPESAYRPPRPSVFKTPDELRQYLEDLSNYYAIAGRPRF**G*KR***VPPKPADAFSYRHPLSTSDIYDLLLQADE*

>*Thermobia domestica* (Zygentoma) NPF1 a [GenBank:GASN01018734.1, GASN01018733.1]

***M***QRNYGLLIVAAAVCLLAVASQLTPVAAKPDPGQLAAMADALKYLQELDKYYSQVARPSPRSESFRRSDLTKVEKAIKLLQLRELDRLYSARSRPRF**G*KR***MELRPVPEDASASDGSDKLWGHLT***RRR****

>*Thermobia domestica* (Zygentoma) NPF1 b [GenBank:GASN01018733.1]

***M***QRNYGLLIVAAAVCLLAVASQLTPVAAKPDPGQLAAMADALKYLQELDKYYSQVARPRF**G*KR***MELRPVPEDASASDGSDKLWGHLT***RRR****

>*Thermobia domestica* (Zygentoma) NPF2 [GenBank:GASN01381375.1]

***M***GSSNVVALVCICVTGLLLVLPCCCEPPIGPDAPKGPKVFTSPDELRTYLEKLSNYYAIAGRPRF**G*KR***APLKMATDAFNYRYPQSTSDMYDLLVSLDE*

>*Atelura formicaria* (Zygentoma) NPF1 a (reconst. from GAYJ01020747.1, GAYJ01020746.1]

***M***QNRTGLWLIVAAVCLLAVASQLTPVAGKPDPGQLAAMADALKYLQELDKYYSQVARPSPRSESFKSGELARMEHALKVLHLQELDRLYSQKSRPRF**G*KR***MELRPIPDDGQMSDVSEKLWGHLT***KRR****

>*Atelura formicaria* (Zygentoma) NPF1 b [GenBank:GAYJ01020746.1]

***M***QNRTGLWLIVAAVCLLAVASQLTPVAGKPDPGQLAAMADALKYLQELDKYYSQVARPRF**G*KR***MELRPIPDDGQMSDVSEKLWGHLT***KRR****

>*Atelura formicaria* (Zygentoma) NPF2 [GenBank:GAYJ01027054.1]

***M***GRTSVLVVLGVCLVGLVLPSCCEPPLSPENVSRPSRPKVFTSPDELRTYLEALSNYYAIAGRPRF**G*KR***VPPKSVDTYNYRFPSNTNDIYDMLQFDE*

>*Nicoletia phytophila* (Zygentoma) NPF1

***M***QRSTKLWIIGAVVCFLAVASQLTPVAAKPDPGQLAAMADALKYLQELDKYYSQVARPRF**G*KR***MELRPIPEDGTPLDASDKLWGHLT***RRR****

>*Nicoletia phytophila* (Zygentoma) NPF2

***M***GSPAVLLLVCLCVIGLVMPCCCEPPLSPENVSRPSRPKVFTSPEELRTYLEALSNYYAIAGRPRF**G*KR***VPPKPADPFSYRYPSSTSDIYDMLSQFDE*

>*Nicoletia phytophila* (Zygentoma) NPF-like

…KNVRLLDRKYSCFNFLCFFIFMTTLALGTESRYIGTDAEDLFSINDEMSLDRKPVFRNVQEL***RR***YLEQLDEWLAITGRPRF**G***

>*Nicoletia phytophila* (Zygentoma) NPF-like

…DEFDYV***KR***AEADLTDRILNDPAALRDYLRQINEYFAIIGRPRF**G***

..............................................................................................

>*Xibalbanus tulumensis* (Remipedia) NPF (completed with JL205812.1] (Christie 2014)

***M***QRSVATWVVLSAFFLLSFSAHFTTVTAKPDPGQLAAMADALKYLQELDKYYSQVARPRF**G*KR***MDSRPLGSVENVIDGNEKLWSHL***RRR***R*

>*Anaspides tasmaniae* (Malacostraca) NPF

***M***QRTGSQSWAWACFWVGVVVLGVLQQSVVEGKPDPTQLAAMADAIKYLQELDKYYSQVARPRF**G*KR***SSFAIPPSDSLLEASERLLETMVR***KR****

>*Lithobius forficatus* (Chilopoda) NPF

***M***LSTGVRAATLALCVLLVLQVHWPVATAAPGPQELANMAEALKYLQELDNYYSKVARPRF**G*R***SLPVRTPEQDVAADSEDRKNLLF***RRR****

>*Hanseniella* sp. (Symphyla) NPF1

***M***RPTVSWMLIGSLLFVVLYHLNILNVTAQEDATVGHVLRDLQHLDKYFNKAGKPRF**G*KR***IPPRATGDGYSEMENE***RR***PTLKRSSCSKDCECSRQLETWS*

>*Hanseniella* sp. (Symphyla) NPF2

***M***HSSLLSLCSILMLLVILLLGSEFSKAEKEVAETPLDESFRNPEELRAYLASLNDYYSVRGRPRF**G*R***SHGKQNNAQVSKFNTRHIDSFHLNY*

>*Hanseniella* sp. (Symphyla) NPF3

***M***VSPSMVAVSCAVLFLFMISTVTSQGDKDAGEDSFRNPEELKAYLTALGDYYSVRGRPRF**G*R***SQGKPVGDLSRQFAVPKHIDPFHWAN*

>*Hanseniella* sp. (Symphyla) NPF4

***M***LPSTTLVAVVCLTIVILVAPGFCQVDDRNVEATSEGSFRNPEELRAYLTDLGDYYAVRGRPR*

>*Eudigraphis takakuwai nigricans* (Diplopoda) NPF1

***M***ARLTYSSFLLAGVCVVLLLGANFQFLEARPSPEQVASMAEALKVLQQLDKHYAQIAKPRF**G*R***SLNSQSYQDMSESEAAYLRDLLEEY***RRR****

>*Eudigraphis takakuwai nigricans* (Diplopoda) NPF2

…LGLSEATSGFVLLLISTALLVQNIHAVPEIQQVLDNFQSEYDYYPVLERKDANSGHIFRKPNELKTFLSVLGGQLSVSGRPRF**G*R***SQYKQPLLLALD*

..............................................................................................

>*Drosophila melanogaster* NPF [[FBpp0082778](http://flybase.org/reports/FBpp0082778.html)]

***M***CQTMRCILVACVALALLAAGCRVEASNSRPPRKNDVNTMADAYKFLQDLDTYYGDRARVRF**G*KR***GSLMDILRNHEMDNINLGKNANNGGEFARGFNEEEIF*

>*Daphnia pulex* NPF a [[Dappu1_443023](http://genome.jgi-psf.org/cgi-bin/dispGeneModel?db=Dappu1&tid=443024), EST_[FE400552.1](http://www.ncbi.nlm.nih.gov/nucest/FE400552.1)]

***M***SSSNNSIQQFLPRSCSLAALVFLVVMAVLAVCVTTTKADGGDVMSGGEGGEMTAMADAIKYLQGLDKVYGQAARPRF**G*KR***GRLPYFDMEDLPLHPQHAY*

>*Daphnia pulex* NPF b [[Dappu1_443207](http://genome.jgi-psf.org/cgi-bin/dispGeneModel?db=Dappu1&tid=443208)]

***M***SSSNNSIQQFLPRSCSLAALVFLVVMAVLAVCVTTTKADGGDVMSGGEGGEMTAMADAIKYLQGLDKVYGQAARPSIRGGKIVQLLQLKQLD***RR***YDNSLVRPRF**G*KR***GRLPYFDMEDLPLHPQHAY*

Neuropeptide-like precursor 1 (NPLP1)

>*Nipponentomon nippon* (Protura) NPLP1

…ETTNPSPTPDPKTDTHIHEDDSEIE***KR***SVEDNDESETGSLYEDWQNTM***KR***NLAALAAQDNDESETGS…

…***KR***NLAALAASHNLPP***KR***YLGSLIANSNYQT**G*KR***TLASVRQ**G*KR***NLASLAASGGYGD**G*KR***NLASLAAGGNIP**G*KK***…

…GDDGNLDDTGDTTGNGGNRW***KR***SLSSLARGNSMPVKGWGYIHNRDQRISRGPGHRDTDMEEQDEDNEPDEIVDTPE***KR***FLGSYVRSGWFPREFRAYSYYPSQE***KR***HIGAVARWGWYPESRYTRYGGGATGLG***KK***SIQSVARFQHPMKSW**G*KR***SDIGSDYESLNDDNNDVHIIYCDEIPDWLLSDDDVIRSSESENSVE***KR***YLLMPAIDNILLRKSFPLRKM**G*KR***PLTGDDDLPVVCIQRNK*

…YP**G*KR***NLASLAASNNYP**G*KR***NLASLASQNAFPP***KR***NLASLMSGSGFTSRVGRDQEFPALDTPQEYTDEDMIDLDQYSFDNSDGNQN…

>*Acerentomon* sp. (Protura) NPLP1-1 [GenBank:GAXE01007993.1 + GAXE01026343.1]

***M***GSKRFLLFILLNCEIFYTLAATSGDTSSLKPAVDPAKQPSHADDVIQ***KR***SVATSDEEQEDEADQVSSLYEDWQSSM***KR***NLASLAAQNSFPP***KR***NLASLMAGNNRPP***KR***NLASLASQNGLP**G*KK***SIASLAAQDDLPE***KR***NMASLAAGRNLPP***KR***YLASLAANGNYA**G*KR***TLAAFRAFQ**G*KR***NLASLAAGGGLS**G*KR***NLASLAAGGNLS**G*KR***NLASLASSGNLS**G*KR***SLASLAAGGGIP**G*KK***SIASLAAGGGIN**G*KR***NLASLMASNNLPP***KR***SLASLRSGSSLYSFARPSRDRDNGLMLSPEEDLEEDMMNINELPEDSDSEPSDGSYTSGSGGGRW***KR***NIASLARSNIMPVKGWGYINNRDLRLSSRPKGSKFNNQNEVEEEEGEEGDDGVVDSVQ***KR***FLGSYVRSGWFPREFRAFSYYPSQE***KR***HIGAVARWGWYPEARFSRFSGGATGLG***KK***SIRSMARIGHPMRQW**G*KR***GDNDVLSNWSEETDDNRIHVVYCDEIPDWLDADSDVTVLDQSDLNEDNSIE***KR***YLLMPAIDNLLLRKSFKGKV**G*KR***PLSEEQDNPIYCIPRN*

>*Acerentomon* sp. (Protura) NPLP1-2 [GenBank:GAXE01088595.1 + GAXE01060397.1]

…PASAKPDAIPEKADDARADDVIQ***KR***SVANSNEEYDNDQVSSLYEDWQTSM***KR***NLASLAAQNSFPP***KR***NLASLMANNNLP…

…***R***YLGSLAASGNYD**G*KR***TLAAFRAFQ**G*KR***NLASLAASGGLN**G*KR***NLASLAASGSSWS**G*KR***NLASMAASGSLP**G*KR***NLASIAAGG…

>*Filientomon takanawanum* (Protura) NPLP1-like

***M***GSKRYLLFLFLNCEVIFILAAAYGETTTSAQPNAEKHSDETPRAADAMQ***KR***SIEADDEEGEDADDASLYDDWQTTI***KR***NLASLAAHNGFPP***KR***NLAAALMANLPP***KR***YLGSLAAQNGLP**G*KK***SLASLASQNSLP**G*KR***NLASLAASRNLPP***KR***YLGSLVANSNYQ**G*KR***TLSSLRALQ**G*KR***NLASLAASGGLS**G*KR***NLASLAAVGSLS**G*KR***NLASLAAGGSIP**G*KK***SIASLAAGGGVN**G*KR***NVAS…

…***R***NLASLRSGTSLYSFARPSRDREIPLSMSQEEELEDILDMSEINQDSDPEGKYSEGQESQDATSGSGGGRW***KR***NVASLARGGAMPIKGWGYISNRDLRMSNRGSSRYNNQNEDQAEPEEDDVMENVQ***KR***FLGSYVRSGWFPREFRAYSYYPSQE***KR***HIGAVARWGWHPEARFSRFSGGAWQGV**G*KK***SINAMARIGHPMRQW**G*KR***SDSDDLSNWSEESSNNEDNQVHIIYCDEIPDWLDAEDDVTAPAQSQIEENSVE***KR***YLLMPAIDNILLRKSFPRKL**G*KR***PLSSEENEVPIYCIPRI*

>*Anurida maritima* (Collembola) NPLP1-like[GenBank:GAUE01051514.1]

…GSIAKSSGLRFPLHSDVVRLNTLGRMQLVPSW***KR***GIPPPLRVMYGAHLGRILNGGDANRQ***KK***GDSDAKLSHDDDSGRQKGESNFESEFYEGPFPMDSVYDEDGGDEGPLRS***KR***FMGSLARGGWFPRQFRGYGTGTQWE***KR***HVGSFARLGWMPHSKATHPFGRDV***KR***TLASIRKSQARNPLIESRVN*

>*Tetrodontophora bielanensis* (Collembola) NPLP1-like[GenBank:GAXI01145942.1]

…EMGILPSSRGLKFARVVKSEHGTVLGDNTSFLHRLVDDNPRTLFGISTAGDVEWNDGSFDQEELNSNSFPQFMETEAAGVK**G*KR***FLGALARGGWFPRQFRGVVEPQWD***KR***HIGSAARLGWMPYKMNRKNDGDY***KR***NIGSLRKTGSKVKLHR*

>*Podura aquatica* (Collembola) NPLP1-like

…IFSNSNTKQQAADEMSYGGTFFEYPFTLNHIPTK**G*KR***FMGSLARGGWFPRQFRGLISEPQWE***KR***HVGAVARLGWMPHSKLSKINGNNHPNSESN***KR***NIGSFRNIQSKTS…

>*Folsomia candida* (Collembola) NPLP1-like[GenBank:GASX01089076.1]

…LLQTSDVQCATENFRSPAAYDYTDELGRSTSREAFIPSISSQIGYNPDVGTVEDLQ***KR***HLGSVVALSGGKDKLLLPRQLTPEML***KR***HLGSIVAASATGSKQKQILSQME***KR***YLGSVLSKSGFASNSARQRSGSSDESLTKS***KR***HLGAMAKINSKPF***KR***TREFDDTESGEISDDLFYDYPVDINNRVDGDSDGFQDSNVVSIKT***KR***FLGSLARGGWFPRQFRGFTAENPYD***KR***HVGSAARVGWLPLSKSDRDYFGEGTGF***KR***NIQSVKQIQYAAPVNTWGWGRQRE***KR***SNTISY*

>*Bourletiella hortensis* (Collembola) NPLP1-like

…PPSCSFYTYRSGMASSGINIFAMVFSIAVLLLCVWQVSCTESQPEDEQQYADYDDNSNVM***KR***HLGSVAAYSGPRSFGRPNSFVVSSNDDNYKY***KR***FLGSLARGGWFPRQFRGFVQTGPSFQ***KR***HIGSVARAGWLPYARAQDQEDQYF*

>*Pogonognathellus sp.* (Collembola) NPLP1-like[GenBank:GATD01013308.1]

…NGGMAVGNWGKPDEMLGKQLLQENLKDEMS***KR***HFGALAASGAYGSRLGMRPGNILPNHGGDEMT***KR***HLGSVAASSGTGSRLPPRLGQNYGRDEMMA***KR***HLGSVAAVSGIRPRVIKSYSDFNSELNDVDHEFNSNPALASRFLIKDLNPNENEVGGESSAESPKRAEFVDDQTLLK**G*KR***FLGSMARGGWFPRQFRGFIEQPMD***KR***HIGAVARLGWIPYNSKMKLDEGGEGSF***KR***NVQSLRKNSLTRPGGN*

>*Sminthurus viridis* (Collembola) NPLP1-like[GenBank:GATZ01006111.1]

***M***ASTGLQTYFYSLTVLLFLFLFTWHVSLAAPTAATEEETYPDYDDAMA***KR***HLGSIAATSGLRHGFVSARTSYPMEDNIYKF***KR***FLGSLARGGWFPRQFRGFVYSGPNANTYQQQ***KR***HIGSVARLGWLPYKNNREDYHM*

>*Jordanathrix leptothrix* (Collembola) NPLP1-like

…WFPRQFRGYVYPSPMGSGFQQ***KR***HIGSIARLGWLPIRDDNMGF*

>*Campodea augens* (Diplura) NPLP1 [GenBank:GAYN01135219.1 + GAYN01141124.1]

…GDLN**G*KR***SFSSLVRNGGVA**G*KR***SLSSLARNGDVS**G*KR***SFVSLVRNGDLN**G*KR***SLSSLIRNGDLN**G*KR***GLSSLARNGDFTG**G*KR***DLASPNGEIPIEVVVFPNDELETEEEE***KR***NVASILKNGGVN**G*KR***TVFDVDGIEMEKKNIASVLKNG***KR***NLASLARN…

…DISWVITLPSWRW***KR***SISSLARSNHLPPI***RR***MFWPAIRNSKEEYDNEEGEQEISAQ***KR***FLGSLARVGWFPRQFRALLEEPYPQAG***KR***GIPHSEEPDNESTSPLYYLADDPAEYYQDPDEMDEDELE***KR***YLILPAVDNILL***RR***LSAKTNNL*

>*Lepidocampa weberi* (Diplura) NPLP1

***M***KTLLVLLPLLLVINKQFTTTHATNITAPVE***KR***SIATLARNGDITSFGSQDIPEEELEED***KR***SVQSIARN**G*KR***GIPTPLMAGNLPQLPLD***KK***SLASLVRNGEVT**G*KR***SLASLVRNGDVS**G*KR***SLASLVRNGDID**G*KR***SLASLVRNGEVN**G*KR***SLASLVRNGDFD**G*KR***SLASLIRNGDIS**G*KR***SLASLVRNGEVT**G*KR***NVASLARTGGVG**G*KR***SLASLVRNGDLG**G*KR***SLASLLRNGDIG**G*KR***SLASLVRNGDF**G*KR***GISSLVRNGDFADSGELDIADAELPSEEEEALAELLEEE**G*KR***SVSSVLKSGNPAG**G*KR***NIGSVLKS**G*KR***NIGSVLKS**G*KR***NLGSVLKN**G*KR***TIMDDDVDLQSSWRL***KR***NIASLARGNHLPPS***RR***IWWPAYRYDDKEDGAQEEDQHEIEGITAE***KR***FLGSLARVGWFPRQFRGFVDNPPSPS***KR***HLSALAQLGLLNPSNPQYQQDKRDLSSPSEEDMEEDIDPNSSISLQWEDKPAYFVRDTDAEGDDIFYVPPEEEDELE***KR***YLILPAVDNLLL***RR***PLQYRAGPNAI*

>*Occasjapyx japonicus* (Diplura) NPLP1 [GenBank:GAXJ01013405.1]

…RVPLLCLVTCLLGCLVAQVTGSTSPIGPPTTPTVPKPTSATPEAPLKLE***KR***HVGAFLRLHQANNNNNNNQQPLRPMSES***KR***QLASLARNGDFTTLVDLPLDPEDVFQMDTDDEGEAGDVDDMEDVQD***KR***HLGSLARSYSFDPSYVRAI**G*KR***YLGALARGGGLSPYQ**G*KR***NLASLARGGGGFG***KR***DGVLDLDEVD***KR***QLSSLARNGDLYSRIPE**G*KR***YLGSVARTGGFFSHQNQED***KR***YLGSLARGGGHQQALV**G*KR***FLGSLAASGGFPVTRVRKSSPCDRDCADNPVQLTAADEGHSPNASSDDEKDRY***KR***NIASLARLSHLPTVLRVSATPNSFLATPDVTDEGYGMDQPEPEQEASIQ***KR***FLAVPYTKQTFPTRVAGERGVVPPPVPRVHVWRREPAEAPYWLPGPTGMASGSTVFPEFS**G*RR***QEEHPVLGTPGTQQQGI*

>*Catajapyx aquilonaris* (Diplura) NPLP1

***M***DAGTRVLVLCVTCLMSCLLFQVSEGGSSPSTTHTVPKPTSATPLSLDDLKVS***KR***HVGAFLRLGHQQD***KR***QLASLARNGDFTTFVEMPGIRPEPEDLLEEDEEDNYGRDAQD***KR***HLGSLVRTGGYSSFDPSYVRTI**G*KR***FLGSLARSGDLG**G*KR***NIASLARGGY**G*KR***DDDEVFDSSELD***KR***QLSSLARNGDLMYNNRLPE**G*KR***YLGSLARNGAYYRQNSEN***KR***YLGALARGGGL**G*KR***FLGSIAASGGIPITRIRKSSPCEDCEEHSNEAKVDGGSEHNAENAKVSSEQKTTKGAENAPEDLDRF***KR***NIASLARSSHLPVVLRVSATPRSFISTTEDDEDVTPGSEEPVGSVE***KR***FLVPYTKQTFTTRVPGERGMVPPAVPGVHVWRREPAEAPYWVPGSTGVAAGPTIIPEFS**G*RR***QEEHPVFGTPGTHPRI*

>*Pedetontus okajimae* (Archaeognatha) NPLP1

…KIDNFVDDVEDAME***KR***YLGSLARSGYLTTKDSQQ***KR***YLAAMAKNGQLSFQRPLEA***KR***SVDESSTLSLQ***KR***ESLDYPLFATTITTNSTGTSSSEDKDSRSK…

>*Machilis hrabei* (Archaeognatha) NPLP1 [GenBank:GAUM01042215.1 + GAUM01146817.1]

…GGLSSYRPQS**G*KR***YFSSLLKDGHFHLSTPRTDLYNGGPFQPDTSNEAEYEIPDDLDLL***KR***NLAALARNGELKY…

… FTS**G*R***NSTSRNADESKEGDKSRP***KR***NIGSLARDGGLPRASATALEAPE***KR***FLGMKSSHTGGTSMTMTSHPRAKTTSMTK*

>*Meinertellus cundinamarcensis* (Archaeognatha) NPLP1 [GenBank:GAUG01235839.1 + GAUG01219141.1]

…GSLPSYRSYD***KR***YFSSLLRGGNFHHATPRSVHIYGTSHRDSSEEPGNESAESTGDDLDLL***KR***NLASLARNGEIKFGQKQDE***KK***NIGSAARAGFRSSGKRTYESDDEEAE***KR***GVGSLARNGNLPSYRS***KK***DPWDLNENSDDENESMI***KR***YVASLAKGGNLPMFGYQ…

…D***KR***SFASLVRNGHFQKPMDM***KK***HIGSLAAGSRFPFQ***KR***ESPGFSIFARTSSIESQNKTSSSADDSRV***KR***NIASIAREGNFPQHGSDDVGSPD***KR***FLGPLARGGWFPRQFRGPEM***KR***HIGALAS…

>*Machilontus* sp. (Archaeognatha) NPLP1

***M***LTYLQRSANILCIVTCICAVSVVNCDSAEYED***KR***YVGSLARTGDLGFLRQQW***KK***LHPVMGSGKGGGVAFAHH**G*KR***EDEEDAEEYNEEE***KR***…

…***R***NLAALARNGEIRFGQKAEE***KK***NIGSLARAGFRGSQLTG***KR***SYEEDEETE***KR***GIGSLARNGNLPSYRS***KK***ESWAIGESDLDNESVI***KR***YVASLARGGQLPSFRYQQ***KK***QDDDSSVD***KR***SLASLVRNSGGHFAKSQD**G*KR***HLGSLAASASFPFH***KR***ESPAFSLIARTLSADNLNKTTGVRDGSRD***KR***SIASIAREDNFPDTAPADEMQSPE***KR***FIGALARGGWLPQQFREPEI***KR***HIG…

>*Tricholepidion gertschi* (Zygentoma) NPLP1

***M***WFSLRPLARPAVPRPLFWILIFAISLKQIDAQKTNTESGTGDGDSKME***KR***NVGVLARTGALPFTRQSEEVSEQDTEDSEDIQ***KR***YLASLARNGDLPFIRKEW***KK***FHPMMSGGRNSGKYDNLEET***KR***SLGSLARNGHLS***KK***SDDLTPEEMLDEILADEIS***KR***SIAALVRNGDIPSLRQEFMKSTDEPEETEAAADDDVSEE***KR***FMAALARNGNLPS***KR***EGVGFSDVFVNGGFD***KK***NIGALAKSGFFPLVRPAS***KK***HMDGTEDETS***KR***SLAALAKNSRPPSFSLSPKSSDLTEYE***KR***NIG…

…LDDVIRQLYEEQEA***KR***SIGSLARNSNLMA**G*KR***SLGSLARNRGRPFS***KK***DDIEDDTEDWE***KR***SAASLMRNGLA**G*KR***SLGSLARNRGRAFS***KK***DDIEDDIEDWE***KR***SAASLMRNRLA**G*KR***NIGALARNRLSSFSVS***RR***DDSFEDEE***KR***NIGAMARNWNLPDLPML**G*KR***SDFDLY***KR***YVAALLRQGRARFEQNSGDDANKTENGPQKPLQE***KR***HIGSLAAQSTYPLR***KK***SPQSALYHSDESEQREIV***KK***STTVPRA***KR***SITEDPIQEKNDTRREEAGPEEDLSKFVHGRS***KR***QVSSFLRLLNKQVPHQMTARNSDEYPLPVFQGSELLDYEEPDDYQENVE***KR***FLGSLARGGWFPRQFRGYLTHSLTPE***KR***HIGALARLGWLPSIRSPRYSR…

>*Thermobia domestica* (Zygentoma) NPLP1 [GenBank:GASN01034845.1]

***M***WPLTRK***M***YKGTPGHGHFSLFWILILTICISYCQGDTTHDQNEASESKTID***KR***NVRVLARTGMLPPTRQENEQQSDTKSNDNNNNNDNEQDVE***KR***YLAALAKNGDFPFIRQQN***KK***FHPVMSGNSNAGSYDTKAD***KR***HLEELI***RR***GELELEMLDDLLQDYHKHGFPAFIMNSEIQENEDGIVPTEDVEEEDPSIVQE***KR***MIAALAKSGNL***RR***PGT***KR***ESLGFSDVFLNGGA**G*KR***NIGSLARMGYLPNRGFD**G*KR***SFSSLLKSGRPTYA***KK***GDIDVLDDDDLVSYE***KR***NLRALLRKGFIPPNQRYQRDDGREDDEDDTENEGEEEEESEEESA***KR***SLSSLIRSRQRPFTHREW***KR***DSLDNALEDLYDER***KR***SVSSLARNWNLPEA**G*KR***DYKSKYLTRNKNKAVS***KK***FSPFDLDEEDIDDLED***KR***SVSSLMRSRVQPL**G*KR***NIVSLARNRNFPFSRRDDYKMESEYD***KR***NIGALARNWDFPYNVPKVL**G*KR***SSDSNDVSDVSKRYFATLLRKGRLPVEVDAKDITETSNASTQLDSSKLEQ***KR***HIGSLAAQSTYPLRKKAPQNSGSVYDNQNPTTSPRT***KR***SVGLIAHDSSLVQDSSDTLESSTMNDNHGIRS***KR***HIGSILRTPSYYPHQMALRSSDEYPLPVMQHSDWFEYDDAEDDVADVPD***KR***FLGRIPYFGRKKQHTPR***RR***HT***RR***N*

>*Atelura formicaria* (Zygentoma) NPLP1 a [GenBank:GAYJ01016012.1]

***M***GLLTPDPAVWSALRTVWWVVVVTLTLISQTHGTTTEGSSPPDDQAVQ***KK***SVGSMARAGLLPSARTRQEDDNEGDVE***KR***YLASLARNRDSPFVNQEW***KR***FHPTMSGVSSNSNYHMVQ**G*KR***YVGALARGGLFPFSRPES***KK***NYHDELLEDILSDEMA***KR***GIQSIVRNGNLPTTSREESDDTEEDEGEQHAEEE***KR***MVATLARAGSLPFGNKEEARYYIVDPEVE***KK***NIGSMARTGFRSYNA**G*KR***SLSSLAKTGRLPFLPLED***KR***NLGSFLSSGFRPSSGAYD***KR***SDELEEIIRSLEENDEEE***KR***GIGALARNNGLPHQQW***KK***DSIEDILEELYKSEEA***KR***NLASLARNSYISTG***KK***SISSLARNSYMST**G*KK***SISSLARNGGRAYDKKDDSQEDYIFDDED***KR***GLSSVIGSRFS**G*KR***GIGSLARNGYLPFTKKEFQFEEEM***KR***YHPHRYPKL**G*KR***SSDMFPNNPLQEEEIS***KR***YLASVVRQGRSSGDGHEEESKSTEVLSKPPQE***KR***HIGSLAAQSVSPLM***KK***SQESSQSSHRDDHSEISASSSKP***KR***SISEESHASSDAKQSKDEDVSRV***KR***HVGSFMRSTNRFPQRVGRSSTDEYASPVYQNTNMYDYDDVDEFTNMGE***KR***FLAPRIIRDSGGT***RR***MPPGVWERGVMPGGTGVLPLYWEDSLLEPEE…

>*Atelura formicaria* (Zygentoma) NPLP1 b [GenBank:GAYJ01016011.1]

***M***GLLTPDPAVWSALRTVWWVVVVTLTLISQTHGTTTEGSSPPDDQAVQ***KK***SVGSMARAGLLPSARTRQEDDNEGDVE***KR***YLASLARNRDSPFVNQEW***KR***FHPTMSGVSSNSNYHMVQ**G*KR***YVGALARGGLFPFSRPES***KK***NYHDELLEDILSDEMA***KR***GIQSIVRNGNLPTTSREESDDTEEDEGEQHAEEE***KR***MVATLARAGSLPFGNKEEARYYIVDPEVE***KK***NIGSMARTGFRSYNA**G*KR***SLSSLAKTGRLPFLPLED***KR***NLGSFLSSGFRPSSGAYD***KR***SDELEEIIRSLEENDEEE***KR***GIGALARNNGLPHQQW***KK***DSIEDILEELYKSEEA***KR***NLASLARNSYMST**G*KK***SISSLARNGGRAYDKKDDSQEDYIFDDED***KR***GLSSVIGSRFS**G*KR***GIGSLARNGYLPFTKKEFQFEEEM***KR***YHPHRYPKL**G*KR***SSDMFPNNPLQEEEIS***KR***YLASVxFSCETRAVFWRWTR***RR***IKIN*

>*Atelura formicaria* (Zygentoma) NPLP1-like[GenBank:GAYJ01315610.1]

***M***QDLWRISAILFLVCIAASQALKDAGQSPSMAEHLVK***KR***NIRVLARTGMFPYVKQEEEESEEEDTEHEEQEDI**G*KR***HLWRGMGVT***KK***SHPLMYGNKSKVVRSLAD***KR***FLRAQVGGRFPLMWRAKGLEQGALMDFPQDAAMEEMDEVGRD***KR***IVAPHARERSHELHIDQEEDPEDYILGGQGLEALHPFPTQHGPVRQHRVV***KR***AVRDLCNYACPSFCPEYRRE*

>*Nicoletia phytophila* (Zygentoma) NPLP1

***M***WLTGWILMTVVCSSAISQLQHEADKQNIYKRNIRVLVRTGMFPYTRPTEDPEEYEKLRSQSGSGILFFKKSHPMIFGNRSHVPEN***KR***FDEKWGDNVEQEAGEDGLEIPSSCITHSEPTSRS***KR***GVQALCGVACNRFCFCDNGNIVTKG…

…RELEEME***KR***GIGSLARNHAHPHQPWKKDTMEDVIRNLYEEEE***KR***NIGSLARSFNIPT**G*KK***SVASLVRNGGDSMTKKEDPDLDVDED***KR***SLSSLVRNRPT**G*KR***GIGSLARNGNLPFTKKELEEIEEEM***KR***SNSFRYPKL**G*KR***SPDSGEQHSVH…

…EVSHRDDHSQISSSSKM***KR***SVATETSSQTHAVRE***KR***HVGAFMRTVGSFPHQMSLRSPEEYSAPVFQNDWLDYEDVEDVLPVEEKRFLGSLVRGGWFPRQFRGYMAHGVE***KR***HIGS…

..............................................................................................

>*Lithobius forficatus* (Chilopoda) NPLP1-like

…ADEE***KR***HVGSLASSNMFPAADEE***KR***HVGSLARSNMFPAADEE***KR***YVGSLARNNMLPMVAEE***KR***HVGSLARNAMLPMLSED***KR***HVGSLARSQMLPGVMGTD***KR***NHLENYDNPDGYDIAEWPTS…

…***KR***HVGSLARSNMFPSADEE***KR***HVGSLARSNMFPSADEE***KR***HVGSLARSNMFPAADEE***KR***HIGSLARSNMFPAADEEKP…

..............................................................................................

>*Drosophila melanogaster* NPLP1 PA [[FBpp0072348](http://flybase.org/reports/FBpp0072348.html)]

***M***QAVLQSAHSSRRLMLLLSMLLNAAIQPRSIIVSATDDVANVSPCEMESLINQLMSPSPEYQLHASALRNQLKNLLRERQLAVGEEQPLGEYPDYLEED***KR***SVAALAAQGLLNAP***KR***SLATLAKNGQLPTAEPGEDYGDADSGEPSEQ***KR***YIGSLARAGGLMTY**G*KR***NVGTLARDFQLPIPN**G*KR***NIATMARLQSAPSTHRDP***KR***NVAAVARYNSQHGHIQRAGAE***KR***NLGALKSSPVHGVQQ***KR***EDEEMLLPAAAPDYADPMQSYWWYPSYAGYADLDWNDYRRAE***KR***FLGRVLPPTRATASTHRSRL*

>*Drosophila melanogaster* NPLP1 PB [[FBpp0110161](http://flybase.org/reports/FBpp0110161.html)]

***M***QAVLQSAHSSRRLMLLLSMLLNAAIQPRSIIVSATDDVANVSPCEMESLINQLMSPSPEYQLHASALRNQLKNLLRERQLAVGEEQPLGEYPDYLEED***KR***SVAALAAQGLLNAP***KR***SLATLAKNGQLPTAEPGEDYGDADSGEPSEQ***KR***YIGSLARAGGLMTY**G*KR***NVGTLARDFQLPIPN**G*KR***NIATMARLQSAPSTHRDP***KR***NVAAVARYNSQHGHIQRAGAE***KR***NLGALKSSPVHGVQQ***KR***EDEEMLLPAAAPDYADPMQSYWWYPSYAGYADLDWNDYRRAE***KR***FLDTSKDPELFGIEHGNDATTAEPADEAYMESDAEAGSEQLPSPQ***KR***HIGAVYRSGFLPSYRYLRSPGGSSGFGGAGGRFSRSGRDARQFV*

>*Drosophila melanogaster* NPLP1 PC [[FBpp0288531](http://flybase.org/reports/FBpp0288531.html)]

***M***QAVLQSAHSSRRLMLLLSMLLNAAIQPRSIIVSATDDVANVSPCEMESLINQLMSPSPEYQLHASALRNQLKNLLRERQLAVGEEQPLGEYPDYLEED***KR***SVAALAAQGLLNAP***KR***SLATLAKNGQLPTAEPGEDYGDADSGEPSEQ***KR***YIGSLARAGGLMTY**G*KR***NVGTLARDFQLPIPN**G*KR***NIATMARLQSAPSTHRDP***KR***NVAAVARYNSQHGHIQRAGAE***KR***NLGALKSSPVHGVQQ***KR***EDEEMLLPAAAPDYADPMQSYWWYPSYAGYADLDWNDYRRAE***KR***FLDTSKDPELFGIEHGNDATTAEPADEAYMESDAEAGSEQLPSPQ***KR***HIGAVYRSGFLPSYRYLRSPGGSSGFGGAGGRFSRSGRDARQFAGYFHQHERLRQPTAAVCKQCFIPNQPMINWSGAGVRGRLSNYYVDPESSLARLPSLSSNSLPSGRPPRPLLRSGAPVYPPFHTWGTPPRITALHRREFRRNSQNYEY*

No NPLP1 known from *D. pulex*

Orcokinin

>*Nipponentomon nippon* (Protura) orcokinin AB1

***M***PTTHKLTVRNASHSIVVLFCSGLLVAGFKLVESNESSDELLSRTLEKLIYLASLNNPAYQTDNRQQGHRQPIE***R***TLDSLGGGNLL***K***GTRSIEDDNRVMRQI***R***GLDSLSGITFGQN***KKR***AYTNNRIYFPSSNLF***KR***NFDEIDRVGFGNFA***KR***NFDEIDRVGFDSFV***KR***SAPSESPTNKQ*

>*Nipponentomon nippon* (Protura) orcokinin AB2

***M***FENHQLSAVSQSIAGLVCCLLFLSGFHPATSSDADDSAQFSRTLDKLIYLASLNNPIPVEYRQHYVHRQPVE***R***TLDSLGNGNLL***K***DERSIGSQQDGGHFLTERQV***R***GLDSLSGITFGTNK***KR***AYTNNRIYFPASNLF***KR***NFDEIDRVGFGNFQ***KR***NFDEIDRVGFDSFV***KKR***SAPSSNTLKQ*

>*Acerentomon* sp. (Protura) orcokinin AB [GenBank:GAXE01020157.1, GAXE01020158.1, GAXE01020156.1]

***M***SSFRLSTSFTQIISFAVILFVLFVAAFTQVESNEREDEVTRTLEKLIYLSSLNDPTYQPSSDYRQQGHRQPIE***R***TLDSLGGGNLL***K***GTRSVDGPSHVMTERQV***R***GLDSLSGITFGTNK***KR***AYTNGRIYFPQSNQY***KR***NFDEIDRVGFNNFA***KR***NFDEIDRVGFDSFV***KR***SSEPSSKQ*

>*Filientomon takanawanum* (Protura) orcokinin AB

***M***SPSQTITLRNISHSIVVLFCSGLLVAGFKLVESNESSDELLSRTLEKLIYLASLNNPAFQTDSRQGHRQPIE***R***TVDSLGGGNLL***K***GTRSLIDVMPERQT***R***GLDSLSGITFGQN***KKR***AYTNNRIYFPSSNAF***KR***NFDEIDRVGFGNFA***KR***NFDEIDRVGFDSFV***KR***SAPSEHPTNKQ*

>*Anurida maritima* (Collembola) orcokinin A [GenBank:GAUE01051922.1]

***M***VRVDVTLVCICLPLILAAPTDDSLKQQISGLYRNDAGYWNMLAPGRGSPHPL***RR***LEDALLQQQYGSGLH***KK***FDSLSGRTFGGE***KR***NFDEIDRTGFGSFV***KK***NFDEIDRNGFGFN***KR***NFDEIDRTGFGSFV***KR***ST*

>*Anurida maritima* (Collembola) orcokinin B (reconst. from GAUE01006830.1, GAUE01051922.1]

***M***VRVDVTLVCICLPLILAAPTDDSLKQQISGLY***R***NDAGYWNMLAPGDEGIEVET***R***AVDSLGRGNIL***KR***SFENGLGLGGEWSLAKNGWRFGAGDKIS**G*KR***GGIDSLGRGNIL***R***SVVEAQEDPLFTYFQNWLSSERGPRPVAYVH***R***SLDSLGRGNIL***KR***STRDGE**G*R***KEGI*

>*Tetrodontophora bielanensis* (Collembola) orcokinin A [GenBank:GAXI01014575.1]

***M***VRVDLTLFCVCIPLIFGAPVDESVKQQLTSGLFRNDDGYWHMINPGLRGGTIVGGHPL***RR***LDSSPYGLQ***KK***FDSLSGSTFGQQ***KR***NFDEIDRAGFGTFV***KK***…

>*Tetrodontophora bielanensis* (Collembola) orcokinin B [GenBank:GAXI01014576.1]

***M***VRVDLTLFCVCIPLIFGAPVDESVKQQLTSGLF***R***NDDGYWHMINPETIGDN***R***YNSNEPFEI***R***T***R***GGGLDSLGGGNIL***R***NLNSLDGAGEWGNTKSSSWRIDSQRQPGYSN***KR***GGGLDSLGHGNIL***R***HINNYDDEQMYNLA***R***SLTSLGKGNLL***R***SLDSLGKGNIL***KR***SA*

>*Podura aquatica* (Collembola) orcokinin A1

***M***RGFEAKMLILAFTTTVMLPFIFCAPVTDDTSLKHQIPSGMYRNDAGYWNMIVPGRDGSSGSQLDYQTHPFIRYGLNNYQ***KK***FDSLSGSTFGSQ***KR***NFDEIDRSGFGSFV***KK***NFDEIDRVGLGFN***KR***NFDEIDRTPFGSFV*

>*Podura aquatica* (Collembola) orcokinin A2

***M***GKLELTLFCAYAFLPFIFGAPTPSDESLKQQQQIAPLPELYKNEAAYWNMVYPQSQ**G*R***NLPLTHPFIHYGLANAMLQNQIDALTAQNFGPQ***KR***NFDEIDRAGFGSFV***K***…

>*Podura aquatica* (Collembola) orcokinin B

…QKHLIKIQLHKIPKQKQPKI***KK***T***KK***GEGEEVENERLKFLS***KR***LSDDGYWSSSKSSGSGS**G*R***GSGWRFVNERFN**G*KR***GGGLDSLGRGNIL***R***SVNYPNEKFNNYENYDDENGGNDEREKEREKELFNYVFDKW…

>*Folsomia candida* (Collembola) orcokinin A [GenBank:GASX01000091.1]

***M***RRIGILASATLCILLPLVLGAPASDDSLKQQIASGLLRGDDFWSSLNQGRFYDGYPS***RR***PSSWAPHNYQ***KR***FDSLSGDAFGLQ***KR***NFDEIDRNGFGSFV***KR***NFDEIDRNSFGNFV***KR***NFDEIDRTGLGFHK***KR***NENDGYGSIV***KR***FVL*

>*Folsomia candida* (Collembola) orcokinin B [GenBank:GASX01000092.1 + GASX01007637.1]

***M***RRIGILASATLCILLPLVLGAPASDDSLKQQIASGLL***R***GDDFWSSLNQELYSDDPLQ***R***LASQYNVRT***R***SGGLDSLGRGNIL…

…RGGGLDSLGRGNIL***RR***VSYDDEVANFIHALGSAGGKL***R***SLDSLGKGNIL***KR***SVRVA*

>*Bourletiella hortensis* (Collembola) orcokinin A1

…EEPQWSFLHPVSVVARSNPRGIGKHPSFDPLSGAGFGFQ***KK***FDSLSGITFGNQ***KR***NFDEIDNAGFGAFV***KK***NFDEIDNVGMGFR***KR***NFDEIDNAGFGAFL***KR***NT*

>*Bourletiella hortensis* (Collembola) orcokinin A2

…QWNFLNPASNGRHL***RR***LESLGRAGLQ***KK***FDSLSGMTFGNQ***KR***NFDEIDNAGFGAFV***KK***NFDEVDNVMGMYP***KR***NFDEIDNAGFGAFL***KR***ST*

>*Bourletiella hortensis* (Collembola) orcokinin B

***M***VQLAILCLCCLPTFLLAAPLEDGLKQQIAAGLL***R***NEEARWNFLNPELAAELELQG***R***SLDSLGRGHIL***R***SLPDGESTWFGGNNNNRDNFGWRFNGYNSGGL***KR***GSGLDSLGGGNIL***R***SIPDDAKMQRYF***R***SLDSLGRGNIL***KR***STRISA*

>*Orchesella cincta* (Collembola) orcokinin A

***M***IRTDFTALFCILVPLPLILGAPVDDVLKQQQQQMSSGAYREEPYWSYYNQGRGLAS***KK***LDSLAGDVFGLA***KR***FDSLSGATFGTQ***KR***NFDEIDRNGFGAFV***KR***NFDEIDRNGLGFY***KR***NFDEIDRTGFGAFV***KR***AA*

>*Orchesella cincta* (Collembola) orcokinin B

***M***IRTDFTALFCILVPLPLILGAPVDDVLKQQQQQMSSGAYREEPYWSYYNQESDDQPGGAFVV***K***TRALDSLGRGNIL***R***SLNGLGSEWGADKGSNWRIGTLGSP***KR***GGGLDSLGRGNIL***RR***VNYDDEISNFIHSLGPVE**G*R***NAFL***R***SLDSLGKGNIL***KR***SVRTISSTQQQQVKE*

>*Pogonognathellus sp.* (Collembola) orcokinin A [GenBank:GATD01012023.1]

***M***VRVDITALLCICLPLILGAPVDEILKQQLSSGLYRNGNEDSPYWSILNPGREPGNRLPF***RR***FDSLGGETIGNQ***KR***FDSLSGATFGTQ***KR***NFDEIDRAGFGSFVKNNFDEIDRNGFGFN***KR***NFDEIDRTGFGAFV***KR***GA*

>*Pogonognathellus sp.* (Collembola) orcokinin B [GenBank:GATD01012024.1]

***M***VRVDITALLCICLPLILGAPVDEILKQQLSSGLY***R***NGNEDSPYWSILNPEYLNEESQQLLPPPGSFTV***R***T***R***GALDSLGRGNIL***R***SLDGLGGEWGNAKSSGWRFGSSSTP***KR***GGGLDSLGRGNIL***R***HVNYDDEVMANLLHSLASNPTVL***R***SLDSLGKGNIL***KR***SIRVESPSGNSNAKTSNGKNAQD*

>*Sminthurus viridis* (Collembola) orcokinin A (reconst. from GATZ01099247.1, GATZ01101681.1]

***M***FHLTILCLCLPSLILAVPANDEVLKQQIASDLLKNDESPYWNLL***R***PGMGGAKQL***RR***LDSLGGNAFGFQN***KR***FDSLSGRTFGHD***KR***NFDEIDNAGFGAFV***KR***NFDEIDNVGMGF***KKR***EQQFDEIDNAGFGAFI***KR***NRN*

>*Sminthurus viridis* (Collembola) orcokinin B [GenBank:GATZ01101681.1]

***M***FHLTILCLCLPSLILAVPANDEVLKQQIASDLLKNDESPYWNLL***R***PDNPIQLQL***R***SIDSLGHGHIL***R***NLGEGDSSWLGGNGGNAGWRLTGYSGNL***KR***GGGLDSLGRGNIL***R***SIPEEYQ***R***YL***K***SLDSLGRGNIL***KR***STRDFR*

>*Jordanathrix leptothrix* (Collembola) orcokinin A1

***M***VPLTILYFCLPTLLFAAPAEEALKQQLSPGLMRSQESYWNLMNGGNGVRPL***RR***LDSLGGDAFGFQ***KR***AFDSLSGSTFGNQ***KR***NFDEIDNAGFGTFV***KK***NFDEIDNVGMGFR***KR***NFDEIDNAGFGAFL***KR***NT*

>*Jordanathrix leptothrix* (Collembola) orcokinin A2

***M***VQLTILYLCLPTLLLAAPVEDALKQELSPALMRSQEAYWNLLNAGQRHNVGRPL***RR***LDTLGGGAFGLQ***KK***AFDSLSGSTFGSQ***KR***NFDEIDNAGFGAFV***KR***NFDEIDNVGMGFR***KR***NFDEIDNAGFGAFV***KR***SV*

>*Jordanathrix leptothrix* (Collembola) orcokinin B

…**R**GGGLDSLGRGNIL***R***SIPQDDYE***R***YL***K***SLDSLGRGNIL***KR***STRE

>*Campodea augens* (Diplura) orcokinin AB [GenBank:GAYN01016961.1]

…IFTTVCFWLMVTEGFAKALSQQDE***R***SLDTLGNANLL***R***NLPYEVNRYRVQ***K***TLDSLGNGNLL**G*R***SLDTLGGANLL***R***DLQEQVELQNYLRNHRPLAD***KR***FDSLSGVTFGQQ***KR***NFDEIDRAGFDSFA***KR***NFDEIDRSGLDSFA***KR***NFDEIDRSGIGSFV***KR***DTNKSSQHKGSAEKVKQ*

>*Lepidocampa weberi* (Diplura) orcokinin AB

***M***ASSSFIVAAIFCLMVAEGLAKAVGSQEE***R***SLDTLGNANLL***R***SLDTLGNANLL***R***NAPYDLNKF***R***IQ***K***TLDSLGNGNLL**G*R***SLDTLGNAQLL***R***SLEEQELQNYLSHYLQPH***KR***FDSLSGLTFGEQ***KR***NFDPIDRSALGVFK***KR***NFDEIDRSAGFDSFA***KR***NFDQIDRVGFGSFV***KR***DTSSKSQEKAKQ*

>*Occasjapyx japonicus* (Diplura) orcokinin AB [GenBank:GAXJ01110974.1]

***M***DRSAFVMLLAALCCLWAASASGRSISQNEE***R***SLDSLGGGNLL***R***SLDSLGGGNLL***R***GLDPYTEAGQM***R***LNYL***R***SLDSLGGGNLLGS***R***SLDSLGGGNLL***R***SIDSLGGGNLL***R***SIDSLGGGNLL***R***QT***R***GLDSLSGMTFGNN***KR***FDSLSGLTFGNQ***KR***NFDEIDRSGFGNFA***KR***NFDEIDRTGFGRFA***KR***NFDEIDRVGFDSFV***KR***EAPKAPAKEPAKSQ*

>*Catajapyx aquilonaris* (Diplura) orcokinin AB

***M***YSIDRTLTLLAAIGFWATIACGKTLSQNEE***R***SLDSLGGGNLL***R***SLDSLGGGNLL***R***GLDPYTEAGQM***R***LNYLRSLDSLGGGNLLGV***R***SLDSIGGGNLL***R***SLDSLGGGNLL***R***NLDSLGGGNLL***R***QT***R***GLDSLSGMTFGNN***KR***FDSLSGLTFGNQ***KR***NFDEIDRSGFGSFA***KR***NFDEIDRTGFGRFA***KR***NFDEIDRVGFDSFV***KR***EAPKEPAKEPAKNQ*

>*Pedetontus okajimae* (Archaeognatha) orcokinin A

…WEEARNRNGIDNIEAVRPI***RR***AFDSLSAQAFGSQ***KR***NFDEIDRAGFGRFV***KK***NFDEIDRNSFGRFV***KK***NFDEIDRAGFGRFV***KR***EAASSKNTN*

>*Pedetontus okajimae* (Archaeognatha) orcokinin B

…L***R***NLDSLGGGNVL***R***NLDSLGGGNVL***R***NLDSLGGGNVL***R***NLDSLGGGNVL***R***NLDSLGGGNVL***R***SLDSLGGGNVL***R***NLDSLGGGNVM***R***NLDSLGGGNVL***R***NLDSLGGGNVL***R***NLDSLGGGNVL***R***NLDSLGGGNVL***KK****

>*Machilis hrabei* (Archaeognatha) orcokinin A [GenBank:GAUM01003361.1]

***M***ASSTMLCVSIVVALSVLCWTAPTDGQSTRRLEALLEEARN***R***DGFGNIEAVRPI***RR***GFDSLSSPIFGSQ***KR***NFDEIDRAGFGRFV***KK***NFDEIDRNSFGRFV***KK***NFDEIDRAGFDRFV***KR***EAV…

>*Machilis hrabei* (Archaeognatha) orcokinin B [GenBank:GAUM01003359.1, GAUM01001141.1]

***M***ASSTMLCVSIVVALSVLCWTAPTDGQST***RR***LEALLEEARNRDGFGAANSQGKAYYSHTNNKHVSGADLSNNAHLKTQNYEQLSEALHNSYLLNKNTNANRKGKHHNSVPPYATL***R***NLDSLGGGNVL***R***QID…

…DSLGGGNVI***R***NLDSLGGGNVL***R***QIDSLGGGNVM***R***NLDSLGGGNVL***R***QIDSLGGGNVM***R***NLDSLGGGNVL***R***QIDSLGGGNVM***R***NLDSLGGGNVL***R***QIDSLGGG…

>*Meinertellus cundinamarcensis* (Archaeognatha) orcokinin A [GenBank:GAUG01247318.1]

***M***ANSTAMLLSIVSIGLLCWATPAIGGPALGLRKPFEKWWEETVQSGHTGNQEALRPV***R***GFDSLSGISFGGN***KR***FDSLRGGFFGGE***KR***NFDEIDRSGFGSFV***KK***NFDEIDRTGFGRFA***KK***SFDEIDRDGFGSFA***KR***EVPQDT***RR***K*

>*Machilontus* sp. (Archaeognatha) orcokinin A

***M***ASSSMSIMCVVAVGVLCWATPAISGPAVGLRQPFERWLDKSFQGPITGNQEPLRPV***R***GFDSLAGISFGGN***KR***FDTMSGDFFGGQ***K***…

>*Machilontus* sp. (Archaeognatha) orcokinin B

…GGNVM***R***NLDSLGGGNVM***R***NLDSLGGGNVM***R***NLDSLGGGNVM***R***NLDSLGGGNVM***R***NLDSLGGGNVM***R***SLYSQGSGNRKYLVR*

>*Tricholepidion gertschi* (Zygentoma) orcokinin AB [GenBank:GASO01254453.1]

***M***NQGSSTMTFWICLFALSLLTGSVFGKPAPNEDRNTD***R***SVDSLRGGHIL***R***ELDSLGGGHLL***R***DLDQLEGSHYLRPS***R***ANSLDSLTGMTFGGS***KR***SLNPYLSHYPGNL***KR***NFDEIDRAGFDSFV***KK***NFDEIDRSGFDSFV***KK***NFDEIDRVGFGSFV***KR***EAPQGNHKNSQ*

>*Tricholepidion gertschi* (Zygentoma) orcokinin B [GenBank:GASO01208942.1 + GASO01011143.1 + GASO01000928.1]

…DNGYAPPVGGE***R***PF***R***QLDSTAEGSLLD***R***AIDSFDRGNILE***R***QLDSLGGGNGLE***R***QLYSLGLGNILE***R***QLEYIDEINLL***R***QLDSLSEANIL***KR***NLDSLVRGYILN***R***QLDSL…

…NIL***RR***NLDSLGGGNIL***RR***NLDSLGGGNIL***KR***QLDSL…

…DSLGGGNIL***KR***QLDSLGGSNIL***K***QQLDSLVGGNIL***KR***FLDSLGG…

>*Thermobia domestica* (Zygentoma) orcokinin AB [GenBank:GASN01028419.1]

***M***YKDTNNMITCLCIILFGLLATNTLAKPTRDEAQDLRTNGETDFDT***RR***ENFL***R***NFESLGGGHFL***R***NLDSLGGGHIL***R***ET***R***GLPLDSLSGVTFGGN***KR***FDSLSGMTFGNQ***KR***NFDEIDRSGFNSFI***KK***NFDEIDRSGFNSFV***KK***NFDEIDRAGWRSFI***KR***EAPESSVKENHQQ*

>*Thermobia domestica* (Zygentoma) orcokinin B [GenBank:GASN01028420.1]

***M***YKDTNNMITCLCIILFGLLATNTLAKPTRDEAQDLRTNGETDFDT***RR***ENFL*R*NFESLGIDKEDQTSYKTKLA***R***QLDSIGGGNIL***R***QLDSIGGGHILRQLDPIGGGNLL***RR***QTDPIGGGNIL***R***QLDPIGGGNLL***RR***QLDSIGGGHIL***R***QLDPIGGGNLL***RR***QLDSIGGGHIL***R***QLDSIGGGNIL***R***QLDSMDDRNILCDENDNGV***R***QLDSIGGGNIL***KR***KLDSTEDTMDFTKNRNNLAASIGSRKNL***R***QLDSIGGGHIL***R***QLGSIANK*

>*Atelura formicaria* (Zygentoma) orcokinin AB [GenBank:GAYJ01028817.1]

***M***CRTHGAMALSVCLLALSLMCVAVRGKPTRPEERPAHHLNYGDLNGEGNPV***R***NLDSIGGGHLL***R***NLDSLGGGHFL***R***DV***R***GTIPLDSLSGVTFGGN***KR***FDSLSGLTFGNQ***KR***NFDEIDRAGFNSFV***KK***NFDEIDRSGFDRFV***KK***NFDEIDRAGWSSFV***KR***EAPAGPDKTRQ*

*Atelura formicaria* (Zygentoma) orcokinin B [GenBank:GAYJ01028816.1 + GAYJ01238059.1 + GAYJ01307294.1]

***M***CRTHGAMALSVCLLALSLMCVAVRGKPTRPEERPAHHLNYGDLNGEGNPVRNLDSIGASKARPPVALQEDLQKDDLQKIFYSTGVLPHQIHFSDDSSIMT***R***NLDTIGGGHFL***R***QRGSEGSRIA***R***…

…LL***R***QRSSVNNGNLLL***R***QLDSIGGGHLL***R***QRSSIDDGNLLL***R***QLDSIGGGRLLP***KR***SFDDDGKMLL***R***QLDSIGGGHLFQQ***R***SSDD…

… ***R***QRSSDGQKNRIE***R***QLDSIGGGHLLP***R***QLDSIGGGHLLQQ***R***ASEPDGSHRYEN*

>*Nicoletia phytophila* (Zygentoma) orcokinin AB

***M***CRDLSSMVTGVSFFIIALLATTAISKPTRTDERGHVGYGELPPDDNTV***R***NLDSLGGGHLL***R***NVDSLGGGHLL***R***DV***R***GLPLDSLSGVTFGGN***KR***FDSLSGMTFGNQ***KR***NFDEIDRSGFNSFT***KK***NFDEIDRSGFDSFV***KK***NFDEIDRVGWGSFV***KR***EAPSHHKDHH*

>*Nicoletia phytophila* (Zygentoma) orcokinin B

***M***CRDLSSMVTGVSFFIIALLATTAISKPTRTDERGHVGYGELPPDDNTV***R***NLDSLGGGNLL***RR***NLDSLGGGNLL***RR***NLDSLGGGNLL***RR***NLDALGGGNLL***RR***QVDSLGGGNLL***RR***QLDAIGGGNLL***RR***QLDSIGGGHLL***R***Q*

..............................................................................................

>*Xibalbanus tulumensis* (Remipedia) orcokinin A

…EPSN***R***TLIPLPGLSFGKPNQ***KR***NFDEIDRAGFNSFV***KK***NFDEIDRTGFGNFV***KR***NFDEIDRTGFGSFV***KK***NFDEIDRAGFGSFV***KR***EAPEEEKSH*

>*Xibalbanus tulumensis* (Remipedia) orcokinin B

***M***YGKILVPYLAFMCLCVSRTIGKPTSKED***R***ALDSLGGGNLL***R***QADD***R***SLDSLGGGNLL***R***NLDSLGGGNLL***R***SLDSLGRGNLL***R***DAPA***R***SLDSLGGGNLL***R***SLDSLGGGNLL***R***SIHAL***KK***ENFPQTLEPLGWGHQL***R***SLDSLGGGHLL***R***SLDSLGGGHLL***R***SLDSLGGGNLL***R***SLDSLGGENPL***R***SLDSLGGGNLL***R***SLDSLGRGNLL***KR***SGPTEATSSQ***R***SLDSLGGGHLL***R***SLDSLGGGNLL***R***SLDSLGGGHLL***R***SLDSLGGGNLL***R***NAEGSLDSLGGGNLL***R***SAEGSLDSLGGGNLL***R***NTEGSLDSLGGGNLL***R***NTEGSLDSLGGGNLL***R***NAE***R***SLDSLGGGHLL***R***NTDGSLDSLGGGNLL***R***NAEGSLDSLGGGNLL***R***HAE***R***SLDSLGGGNLL***R***NLDSLGGGHLL***R***EV***R***GLDSLTGSTFGGN***KR***FDSLSSKNGLNSSKNDKSIL*

>*Anaspides tasmaniae* (Malacostraca) orcokinin A

…RFTVSPYESERFA***KR***NIDEIDRTPFDNFF***KR***HMVGWRGFVG**G*R***SPTDRLSG*

>*Lithobius forficatus* (Chilopoda) orcokinin A like

…ENLIDSQNSPGILDSSML***R***DYYPGNKYVGKVL***RR***KESTRSSAPSSSL***R***AIDTLSGVTFGNQ***KR***FDSMAGVTFGDT***KR***FDSMSGYTFGSPLQ***KR***SPYLLVRPMSSEMLM***KK***AEELYGSPNMDGVLPKHYYDEDDEGLMAPDSASALQ*

>*Hanseniella* sp. (Symphyla) orcokinin A

***M***AGGTANCFNVLSTFLAVMICLQANIIEGYDADRE***R***SIDSLGGGNFV***R***ALDSLGGGNLV***R***NVLGQRPRSKSEINQILDSGESGSNRQTSGYYAALVPAEVAYILNHKGYGVIPLEENPPRAFNAQRQQ***KR***NFDEINRQ…

…AFDSFY***KR***NLDEIDRSGFGGFY***KR***NFDEIDHTGFGGIQ***KR***STLTEDLGKTKEHLESE***KK***TQ*

>*Hanseniella* sp. (Symphyla) orcokinin AB (?)

…GNFIRALDSLGGANLIRDLEQQEHNA***R***AIDSLGSGNLI***R***SIDSIGGSSML***R***HSPGYPVVVVPPEVASIL***R***HKGYYVTSMGDQLV***KK***QDPLSLWQ***KR***NFDEINRQAFDSFS***KR***NLDEIDRSGFGGFS***KR***NFDEIDRSSFGGLS***KR***NFDEIDRTGFGGLA***KR***NFDEIDR…

>*Eudigraphis takakuwai nigricans* (Diplopoda) orcokinin A

…VPKSIDSMSGITLGHPAE***KR***TFDEIDRAGFDGFH***KR***NFDEIDRVGFDGFV***KR***VPQLPVAEDKVPII*

..............................................................................................

>*Drosophila melanogaster* orcokinin A [[FBpp0072118](http://flybase.org/reports/FBpp0072118.html)]

***M***NLYVLLAVVSVFLNFIHAAPGVDISNDELLDGKYLCEAGSKKYDGPFIVRLISAANGQTVVCYECSQSEFKTKYSVKQCAAGKIGSGHHRDLVPYLVRMDPLYKDTWSSKL***KR***NFDEIDKASASFSILNQLV*

>*Drosophila melanogaster* orcokinin B [[FBpp0304306](http://flybase.org/reports/FBpp0304306.html)]

***M***NLYVLLAVVSVFLNFIHAAPGVDISNDELLDGKYLCEDLPALRELCNGNSVSLRQTGLPNDDLSTSPLFNEFYKLFQRNVNIFLSKNKSPESLQERFSRTVS***KR***GLDSIGGGHLI***KR***TQSRQFLSD*

>*Daphnia pulex* orcokinin AB1 [[Dappu1_309283](http://genome.jgi-psf.org/cgi-bin/dispGeneModel?db=Dappu1&tid=309283), GenBank:[EFX70781.1](http://www.ncbi.nlm.nih.gov/protein/321459731?report=genbank&log$=prottop&blast_rank=1&RID=XYXNJVFY015)]

***M***NCLKFRLVAVAILIFNVVTALNYQSEEAVGVEHERDRDSLGGGHILRGLDSIGESNLLRAIYREKPRDFLRIN***R***GLDSLSGASFGIE***KR***LDSLTGLGFGSQ***KR***NLDEIDRSNFGTFA***KR***NLDEIDRSDFGRFV***KKR***ETMEAESSQQQH*

>*Daphnia pulex* orcokinin AB2 [[Dappu1_256705](http://genome.jgi-psf.org/cgi-bin/dispGeneModel?db=Dappu1&tid=256705)]

…MPFKEKTRKTPRLFADKS***R***TGLVSSGASFVIE***KR***FDSLTGLGINSQ***KK***NLDEIDRSNFGTFA***KR***NLDEIDRSDFSRFV***KKR***ETMEAESSQQQH*

Pigment dispersing factor (PDF)

>*Anurida maritima* (Collembola) PDF [GenBank:GAUE01047612.1]

***M***TRVLGIAVGVILLLTLFNGIEAYPANNIGNSDLEKSQIQALMELSSRLRAARLAQLGYFYGSDSP**G*KR***NSEMINSLLSLPKSMSDA**G*KR***K*

>*Podura aquatica* (Collembola) PDF

***M***NSKFTLLFTIFILSVIAFVPTIVNASRDELDDPALLVELASRLLRASHHLSYGFNSNGVFSPTM***KR***NSEMINSLLSLPKSMND…

>*Bourletiella hortensis* (Collembola) PDF

***M***KFAIFVLLVVAASLVLTSASPPSRDQYGAESVSEAEARAVAELAARLLRAARMSPFGYMGGVPNYM***KR***NSEIINSILGLPKVMNDA**G*K****

>*Orchesella cincta* (Collembola) PDF

***M***NSLLIILSVCVVVISVTDMASANNPLANGEQVDPETAELLERYIKAHRISAMGYMGGLPWFSNQQ***KR***NSELINSILGLPKVMNDA**G*RR***K*

>*Pogonognathellus sp.* (Collembola) PDF [GenBank:GATD01090002.1]

***M***KFIFAVIFLVSVISACALAAPPQQQGQDTEDVMDQLANQLAHRARMSLFAFNGLRELPYLLSQNVN***KR***NSEMINSLLGLPKVMNDA**G*RR***K*

>*Sminthurus viridis* (Collembola) PDF1 [GenBank:GATZ01096428.1]

***M***KFIIACALLVASVVIFSSVEASSATPSDQYLSNSDLEARMVAELAARIARAARISPFGYMGGFPTQQV**G*KR***NSELINSLLGLPKVMNDA**G*K****

>*Sminthurus viridis* (Collembola) PDF2 [GenBank:GATZ01090998.1]

…LVASIVILSCYEASAAAQVDRYLSDSDLEARLVAELAARIARAARISPFGYMGGFPTQQV**G*KR***NSELINSLLGLPKVMNDA**G*K****

>*Jordanathrix leptothrix* (Collembola) PDF

***M***KVAVFLVACVLAVVFFSSMVLAVPASVQQQQQDLFEAASDVEASKAALELANRILRAQRMSLFGFGGGFPGGLV***KR***NSELINSLLGLPKVMNDA**G*KK****

>*Pedetontus okajimae* (Archaeognatha) PDF

***M***KNLWSVFLLLYLLRLPFTCSMPLEEDVKYVEREYAADILSRLLHWARAGEPLGMH***KR***NSELINSLLGLPKVMNDA**G*R***K*

>*Machilis hrabei* (Archaeognatha) PDF [GenBank:GAUM01019803.1]

***M***KNLWSVFLLIYLLRLPFACSLPLEDDVKFVEREYAADLLSRLLHWARAGEPLGMH***KR***NSELINSLLGLPKVMNDA**G*R***K*

>*Meinertellus cundinamarcensis* (Archaeognatha) PDF [GenBank:GAUG01230494.1]

***M***KYLLSAILLLYLLRLPLVSSLPLDEDVKYVQREYAADLLSRLLHWARASEPLSAH***KR***NSELINSLLGLPKVMNDA**G*KK****

>*Machilontus* sp. (Archaeognatha) PDF

***M***KYVWSAFLLLYLLRLPLASSLPLEEDMKLMEREYVTDLLSRLLHWSRGGEPLVAH***KR***NSELINSLLGLPKVMNDAG**G*R***K*

>*Tricholepidion gertschi* (Zygentoma) PDF [GenBank:GASO01247458.1]

***M***QNLASVLVLLLLYVVRFSDVSALEFDNDKYLDREYAKEPPAWLLHLIHGDQALWTH***KR***NSELINSLLGLPKVMNDA**G*R***K*

>*Thermobia domestica* (Zygentoma) PDF [GenBank:GASN01337382.1]

***M***RNFTCIILLLYFLRVATCLDFYEEKYADREYNKDLATWLLQLVRGDQNGCPH***KR***NSELINSLLGLPKVMNDA**G*R***K*

>*Nicoletia phytophila* (Zygentoma) PDF

***M***YLLQTSAKPGLRHIASLILVMYLLRLPVACSLDFDDEKYSDNEYAKEVAEWILQQVRGDRPICNH***KR***NSELINSLLGLPKVMNAV**G*R***K*

..............................................................................................

>*Anaspides tasmaniae* (Malacostraca) PDF1

…LLVVMALVAITNAQEDLKFSEREVVTSLAADILRVVRGSWGGAVPH***KR***NSELINSLLGLPKVMNDA**G*RR****

>*Anaspides tasmaniae* (Malacostraca) PDF2

***M***TVFIIRACFLITAILAAVTSHPVWGDELSIQDLSKVIEEMTSTGEELPLELLVQWVSQGQPSPGQRRPWMAKARPWMAKTHPAAT***KR***NSEILNTLLGSQGLGALRNA**G*R***K*

>*Eudigraphis takakuwai nigricans* (Diplopoda) PDF

***M***RLFQLHHLVAINLLLMMCVWVRSMSAMPASFTDDLDLAERELLAELVYKLSRLPRSFNNNPLPT***KR***NSELINSLLGLPKVMTDA**G*KR****

..............................................................................................

>*Drosophila melanogaster* PDF [[FBpp0084396](http://flybase.org/reports/FBpp0084396.html)]

***M***ARYTYLVALVLLAICCQWGYCGAMAMPDEERYVRKEYNRDLLDWFNNVGVGQFSPGQVATLCRYPLILENSLGPSVPIR***KR***NSELINSLLSLPKNMNDA**G*K****

>*Daphnia pulex* PDF (PDH) [[Dappu1_306549](http://genome.jgi-psf.org/cgi-bin/dispGeneModel?db=Dappu1&id=306549), GenBank:[EFX87718.1](http://www.ncbi.nlm.nih.gov/protein/EFX87718.1)]

***M***HQLSAKLSHLSIALFVLLVSFATDAQSAPPSISSNNRPEAQMSIQEMEKFLEGLTRYLHRQHLDLPKVHQQSQEEQPGSYEADAIDRSGDMSAPTETERSSSSSSSELANHSLLSHPRPPMANKWPWSLSHLERIEDDPDFKERQQPYA***KR***NSELINSLLGLPRFMKVV**G***

Proctolin

>*Nipponentomon nippon* (Protura) proctolin

***M***SSRLVTLLLSLVVTSLIIATFNQSEARYLPT***R***SNSDRLDRLRELLTDLLENEAENRDVI***RR***PLV***KR***SIETGSDSASGSAVSGLRLEKPSRNIKV*

>*Acerentomon* sp. (Protura) proctolin [GenBank:GAXE01137894.1]

***M***MSDRLVTILLSLLVTSFIIATFSSQSEARYLPT***R***SHDDRLDRLRELLQDLLESEVDNRDVI***RR***PLV***KR***NADLTNSIEEESFRNTNRKMKI*

>*Filientomon takanawanum* (Protura) proctolin

***M***SDRLVTLLLSLLVTSLIIATFHQSEARYLPT***R***SHDDRLDRLRELLQDLLENEAENRDVI***RR***PLV***KR***NANLVNSGEEITNRLEDGQRKIKI*

>*Anurida maritima* (Collembola) proctolin [GenBank:GAUE01008967.1]

***M***KMMLAVLLAAFLASEYAVVMVSGRYLPT***R***SQDDKLERLRELLKDLLESQSMNSNRPQETSYDMQGGPLSPSRIGTGIGSGGRAILF***KR***DIHEPNSGYLANPSGNEGRHLDGLESSDRI*

>*Tetrodontophora bielanensis* (Collembola) proctolin1 [GenBank:GAXI01019527.1]

***M***IEGIGK***M***AVLVVMAVCLASESTMVTVSARYLPT***R***SQDDRLERLRELLKDLLESQSYDNRQVLNGDQQGGIELGSSGGPLGIGSGGRSIIY***KR***EAGSVLKQPRILAPSNDQVPIPM*

>*Tetrodontophora bielanensis* (Collembola) proctolin2 [GenBank:GAXI01060275.1]

***M***SEAKFTFLLIAIISALIGQRISLSEARYLPT***R***SQEDRLVKLRELLQDLLKPE…

>*Podura aquatica* (Collembola) proctolin

***M***LRLVNIFIFGFLFVLFVSQQEARYLPT***R***SQDDRLTRLREILHDLLESEKEKKEEEYGGGGVENSNNDMKESLLQYDENNGYNLRAPQLKTKQIF***KR***ATPLFVHHTQDRSINV*

>*Folsomia candida* (Collembola) proctolin [GenBank:GASX01013051.1]

***M***MITLVIAAFLTSQVLVTVSARYLPT***R***SQDDRLEKLRELLRDLLEGDLE***RR***PATSGVDYEMMAQQGRPPVY***KR***STNAYTDLDAAAAAGKVGPNFLHRIVPNLRKSLD*

>*Bourletiella hortensis* (Collembola) proctolin

***M***SGSATIFLWTVITLLVVSEMTIVNVNARYLPT***R***GQDDRIERLRELLKDLLGNQYDYAAGGDGVESAGSSLLAPSAGRGHTVMF***KR***DIPASDHLVIHNTSQ*

>*Orchesella cincta* (Collembola) proctolin

***M***EQLSVRVALVLLAAAFLASTSTMVAVSARYLPT***R***SQEDRLERLRELLKDLFDSDYDHRTYPLADVSVLPERAAIF***KR***SAGLAYAPGVTGHDLVKGLRNGALSSSAALGGNGLVQLPSAMLDSPYADPERKVMK*

>*Pogonognathellus sp.* (Collembola) proctolin [GenBank:GATD01014090.1]

***M***SLARVVALVVTVFLMSESSIVTVSARYLPT***R***GQDDRLDRLRELLKDMLQSDYDRRQVDVAESSPHQQYSMY***KR***SAEYSKIPAHMLSRSSELFSDLGSETEHSSEKNLRSTIKN*

>*Jordanathrix leptothrix* (Collembola) proctolin

***M***VSRKDTISPRVLLWTFLAVVLVSEMTIVSVSGRYLPT***R***SQDDRIDRLRELLRDLL…

>*Campodea augens* (Diplura) proctolin [GenBank:GAYN01134979.1]

***M***KMAFSLRHILCACVLTFALSMWLETEARYLPT***R***SQDDRLERLRELLRDLLESEQGDRLEMD***KR***VFF***KR***DVTSHGPSASAASRKDHLRSPAVATYVAE*

>*Lepidocampa weberi* (Diplura) proctolin

***M***TLLWRHLLCACVLTFALYTWMGVEARYLPT***R***SQDDRLERLRELLRDLFESEDGRPEVD***RR***TFF***KR***DVSTLQAPSFAASRKDHLHSAAVPAFTAE*

>*Occasjapyx japonicus* (Diplura) proctolin [GenBank:GAXJ01012368.1]

***M***RSVSRQLLVACLVACFLCVWVSEARYLPT***R***SQDDRLERLRELLRDLLEGDSDRLDME***KR***MFF***KR***EAGHVSPGSAAGQLEQPGFLGQ*

>*Catajapyx aquilonaris* (Diplura) proctolin

***M***RSVSRQLLVACLVACFLCMWVSEARYLPT***R***SQDDRLERLRELLRDLLEGDTDRLELE***KR***MFF***KR***EAAHGSPGASAGQLEQPGFLAQ*

>*Pedetontus okajimae* (Archaeognatha) proctolin

***M***ACSIRHMLLLCFVVLAIAAWSAESRYLPT***R***SQDDRLERLREMLRDLLEEEPE***RR***EMD***KR***MFF***KR***EVPEPAAFPPHMTLAGQK*

>*Machilis hrabei* (Archaeognatha) proctolin [GenBank:GAUM01184003.1]

***M***ACSIRHMLLLCLVVLAVAAWSAESRYLPT***R***SQDDRLERLREMLRDLLEEEPE***RR***DMD***KR***MFF***KR***EVPEPAAFPPHMTLAGEK*

>*Meinertellus cundinamarcensis* (Archaeognatha) proctolin [GenBank:GAUG01149244.1]

***M***ACSMRHLLLLCLVVLAIAAWSVESRYLPT***R***SQDDRLERLREMLRDLLEEDPE***RR***DFDK…

>*Machilontus* sp. (Archaeognatha) proctolin

***M***ACSMRHLLLLCLVVLAVAAWSAESRYLPT***R***SQDDRLERLREMLRDLVEEEPERRDMD***KR***MFF***KR***EARELPALAPHLNLEAQQ*

>*Tricholepidion gertschi* (Zygentoma) proctolin[GenBank:GASO01253040.1]

***M***ACSVRHMLLLSLVMLAVSAWVSESRYLPT***R***SQDDRLERLRELLKDLLESEIEKSNYD***KR***VFY***KR***EVPEVPAAQVGAQSALVEQ*

>*Thermobia domestica* (Zygentoma) proctolin [GenBank:GASN01398864.1]

***M***ACSVRHLLFLTVIIVAICAWTCESRYLPT***R***SQDDRLERLRELLKDLLENDIEKSEYE***KR***IFY***KR***EAPAQFQTESQMSQHLLTDE*

>*Atelura formicaria* (Zygentoma) proctolin [GenBank:GAYJ01315759.1]

***M***AFSMRHLLLLALVMLTLCAWMSESRYLPT***R***SQDDRLDRLRELMRDLIESEIEKSNYD***KR***VFF***KR***DAPEFPIGRQVPPSSLTEQ*

>*Nicoletia phytophila* (Zygentoma) proctolin

***M***ACSSRHILLLALVLLSVCCWMSESRYLPT***R***SQDDRLDRLRELLRDLIESEIEKSNYD***KR***VFF***KR***DAPEYPAARQVAEPALTEQ*

..............................................................................................

>*Xibalbanus tulumensis* (Remipedia) proctolin

***M***VYSTRHLLLASLVFMALSCWLAEARYLPT***R***SDDNRVERIRELLKDLIESEVE***KR***DYD***KR***FVF***KR***EVPAPVHQMAPVEGLMDH*

>*Anaspides tasmaniae* (Malacostraca) proctolin

***M***VLAFLMVTVTLCHARYLPT***R***GDDSRLEEIKDMLREILERAEGSSSQNHLASAYE***KR***FLF***KR***AASLPVMGMKEIDYQLTNLPQ*

>*Lithobius forficatus* (Chilopoda) proctolin

***M***AGKRVLMVALVLMVVYCWTAQARYLPT***R***ADDSRREEIRELLRELLDGAAE***KR***DYD***KR***FLL***KR***SADVNRAPAEEANFLN*

>*Hanseniella* sp. (Symphyla) proctolin1

***M***AVKPVLLATLAVVMLCVWSTHARYLPT***R***SDPS***RR***EQIKEILRELLDIQPDRPTEMD***KR***FVV***KR***SAILPAFQSAGNNDNWN*

>*Hanseniella* sp. (Symphyla) proctolin2

***M***AQGKTILAAMLVICLCVWTAHARYLPT***R***SDLS***RR***DQIKELLRELLDIQADQSVDVD***KR***YVV***KR***SAELPTHHVTGNEEWN*

>*Hanseniella* sp. (Symphyla) proctolin3

***M***AVKTVFLATLAVVMLCVWSTHARYLPT***R***SDPT***RR***EQIKEILRELLDIQADRHADMDKQFIV***KR***SAVLPGSHATGSNADWN*

>*Eudigraphis takakuwai nigricans* (Diplopoda) proctolin

***M***SSSRTAVFCLVVVGMLLLHSVFLVDCRYLPT***R***ADDT***RR***DEIRDLLRELLEGQLDRHEYDNRFVV***KR***ETSYRPSLRTHSASQPDSQ*

..............................................................................................

>*Drosophila melanogaster* proctolin [[FBpp0079184](http://flybase.org/reports/FBpp0079184.html)]

***M***GVPRSHGTGIGCGSGHRWLLVWMTVLLLVVPPHLVDGRYLPT***R***SHGDDLDKLRELMLQILELSNEDPQQQQQQQQQQQHPQLRLHNEATGGSSSSSNINNPRVSNGNSNAAWLQKLSAMGALDELGGDGARFGPNYGRY*

>*Daphnia pulex* proctolin1 [[Dappu1_442870](http://genome.jgi-psf.org/cgi-bin/dispGeneModel?db=Dappu1&id=442870)]

***M***LKSTSLKALLTLFMVTFVIMASSSGSSWGVDARYLPT***R***SDPLSPIGPPRGEDPRFDRLYDIIIRELLRNGGGDMLDEAKYPIKNQFSSGAY*

>*Daphnia pulex* proctolin2 [[Dappu1_305680](http://genome.jgi-psf.org/cgi-bin/jgrs?id=Dappu1_305680), GenBank:[EFX88050.1](http://www.ncbi.nlm.nih.gov/protein/321477091?report=genbank&log$=prottop&blast_rank=2&RID=XW90GPT3015)]

***M***LKSTSLKALVTLLVVSFVLMASSPRAADARYLMT***R***SDPLLSPIGPPYGKDPRFDRLYDIITKLLQNGGGDLEYQIKSQLDSGP*

RYamide (RYa)

>*Nipponentomon nippon* (Protura) RYa

***M***VSRQGVSLCLLGVSVFLLSVLVSVTSGSEGGDLDSNIDGEYFPNGRY**G*KR***DDANQNGGKIITIPVRHYLELGNHNGDGTIGLMVVK***KK***SNQPTAPEDAEIRDIRQDDPDTYFGGSRY**G*KR***SRSNRYKEFIERIRQQQQRNASL*

>*Acerentomon* sp. (Protura) RYa [GenBank:GAXE01008196.1, GAXE01008197.1]

***M***ICFNQTSICNLTHLVLQTILISMLLFSVFEYCSSSEQRFFNAKKAISDDLNDSSKADTEDVIRIPLARFGYDKLSSGSDSSDFGFKSIGLRE***RR***QDSPDSGTYFGGSRY**G*KR***AQQVPPTLRALISNICGNHHASLQNNRF*

>*Filientomon takanawanum* (Protura) RYa

***M***KFFKQYQLFANLAILLVFTIMLLFSLSNGATLEQFYA***KK***PAxDTVEIVPIPLSRYDYEKLGQSGDISDFGGYLV***KR***SKPNNSRE***RR***QDSPDSGTYFGGSRY**G*KR***AQPHPVPTNLRALVSRICGARALPETDRVNAERSQSWSKFLSTN*

>*Anurida maritima* (Collembola) RYa [GenBank:GAUE01051424.1]

***M***NSSFSFSSGMRVVLSLLSLILVLTLVNGVYTGPRY**G*KR***DSGDTVVGRVL***RR***TNALRNIQSRSAGQ***RR***FYPGTRYS**G*KR***ASWQETPILTSDGDLSAQPLPLSSIPYDCLYVPFVGVRCSHPQETPSYWRSLGNTVTGDEETYAGTVNNENGNNEAVTGVDP*

>*Tetrodontophora bielanensis* (Collembola) RYa [GenBank:GAXI01151347.1]

***M***VSLGSWNHPKTLLLTALTLVIILTVANAAYTGTRY**G*KR***EPFDSIGRMAPRSDRDRNIQSRSSGT***R***FYPGTRFG**G*KR***SYWQTQPQPTPILSELIENSDNFPSSNIPYDCVYKPFIGVRCSSIYRVEENNYLNNNNNDEEQMIRNDGFESGDGSGSSTLVAAAAGAGGP*

>*Podura aquatica* (Collembola) RYa

***M***MIEGSVCVRSGVGLFLALTILTLSLMFVSGAVYSGPRF**G*KR***EQNSEMVGRVLQRTSGVRSLQARSAGE***RR***FYAGTRYG**G*KR***SFLPPIPPQLTINEALGNEGGGGAPYDCIYVPFNGIRCSNFKEDAGEEGGGTGQISDSLKIDNNESEQ*

>*Folsomia candida* (Collembola) RYa [GenBank:GASX01009854.1]

***M***PPMSKVLLVTLAMLTLYSVLVSGAGYLGPRY**G*KR***LPLPMQGGEVSRIVGRSSGPRTIQSRSQA***R***FYAGSRYG**G*KR***SYWPSLMQSDVDSSLDSGANNASPYDCVYLPYVGIRCGVLRSESAFSSGGSDHDPAEDMEMSPSSSSYGGGSPSLTRTSIIDTVPSSGSGSSDN*

>*Bourletiella hortensis* (Collembola) RYa

***M***VMVTLVTLYFLLAISCSSAYMGTRY**G*KR***EMVRSIGQRSIVNRGARFYPGSRYG**G*KR***SYWRPTAFMSEEGETGPESPSMPNSPYDCVYVPFVGVRCSRPEEGTNYWSNDEDLSNGNGMNPSASSSSSSSGGSVDQA*

>*Orchesella cincta* (Collembola) RYa

…GLRY**G*KR***LPYEVTRVNLRSSTPRVIQSRSQV***R***FYAGSRYG**G*KR***SYWDTSMSDSESSPPTDYYQQQVRSGSGTNSPVDCLYFPYVVVRCFPRHFDSTWNNLGESDEDSGSPNTMNGNPSSSSPVGGSDAS*

>*Pogonognathellus sp.* (Collembola) RYa [GenBank:GATD01087802.1]

…PLKVLLVTLVTLTLLLAVHGYLGPRY**G*KR***EPDGSLRGSGRIRINGRSGQRNIQSRSQT***R***FYAGSRYG**G*KR***SYVPAAPAIPDVDNYELPSASNVPYDCGYVPYFGIRCVARQDESFWTSENEEEPMPSANAPLSSGASSDQ*

>*Sminthurus viridis* (Collembola) RYa [GenBank:GATZ01007060.1]

***M***NYLRNFFPLKLAMVALIALYLILAISSSSASYYGPRY**G*KR***ESDLSRFQARSIGQRNNRGA***R***FYPGSRYG**G*KR***SYWQPTSFMPSDADMGSADSLNVPNVPFDCILIPNLGVRCSRPEAVSDWNNNEDEVSAGNAASAVSSSGGSSSAAADQP*

>*Jordanathrix leptothrix* (Collembola) RYa-like

***M***RGTYALLLVAAVVFVSCDAQGFGFSLNPVIEIGGGGGFGGLYNRDRGFNDYNGRYGERDNYGGRGEYYGGRGRDGPYYG**G*RR***DEYRPYPYRY*

>*Lepidocampa weberi* (Diplura) RYa

***M***YRQASYILLGVVGVTLLLSLCQEVQSSSFYANGRY**G*KR***DDHKITERSSKYYGGSRY**G*R***SDASIAVQSRSAGKFYGGTRY**G*KR***DEVFDSFPTLLLSSDVDSTDAYECEFIGVLNFYRCFSRKDEGSVEGQQTRK*

>*Occasjapyx japonicus* (Diplura) RYa [GenBank:GAXJ01111341.1]

***M***MLRHPVWMLAALLGLTLMTVTPTFSQNFYPSVRY**G*KR***ADHAMAERAADKFWGGSRY**G*R***TDSDIEVSARAADKFYGGMRY**G*KR***NDVPLADALPQVGSDFLEGSQLECVYTGVTNIYRCSS***RR***DVSNEEAGSSK*

>*Catajapyx aquilonaris* (Diplura) RYa

***M***LRHPFWMFAVLLGLTLVVVTPTFAQNFYPSVRY**G*KR***GDHAVAERGADKFWGGSRY**GR**TDPDVETRTADKFYGGMRY**G*KR***SDVSIADPLPQVGSEFFEGSQLECIYTGVTNIYRCT***RR***DVSNEEGSSK*

>*Pedetontus okajimae* (Archaeognatha) RYa

***M***YFNSTSSRKMAVYAALIALASLLVLSSAQQFYPNGRY**G*KR***NNDLQLAERTNTHLYPGSRY**G*RR***GSAPNYENRAADKFFVGSRY**G*KR***GHPDDEARLADNDELSQVGCVYTGISNLYRCFRKDNASEETTRM*

>*Machilis hrabei* (Archaeognatha) RYa [GenBank:GAUM01064854.1 + GAUM01127804.1]

***M***HFNSNSSQK***M***ALYAALLALASLLVLSSAQQFYPNGRY**G*KR***SDEQDVQER…

…DKFFIGSRY**G*KR***GQIDDE***RR***LDDIDEFSQVGCVYTGISNLYRCFRKDNTSEDTTRM*

>*Meinertellus cundinamarcensis* (Archaeognatha) RYa [GenBank:GAUG01245527.1]

***M***ALYVVLLVFASLLFLASAQQFYPNGRY**G*KR***DNEHDIEVRSNRFYPGSRF**G*RR***DMSAAMPQTRSQTDKFFLGTRY**G*KR***GKQDDAIRPTESEEEMQVGCVYTGVSNLYRCFRKDNTSEEATRM*

>*Machilontus* sp. (Archaeognatha) RYa

***M***VSLSSSSRQ***M***ALYVTLLFLASLLVLSSAQQFYPNGRY**G*KR***NEIEARSSNRFFTGSRF**G*RR***DASAAVPQTRSGVDKFFLGSRY**G*KR***AEQEDEVRPTESEEQLKVGCVYTGISNLYRCFRKDNASEEIATM*

>*Tricholepidion gertschi* (Zygentoma) RYa [GenBank:GASO01253624.1]

***M***AYQGVSSRHLVATAILVLALCSFLDVTSSLQLYQSGRY**G*KR***ADNAIQGRDTNRFYTGSRY**G*R***SGQNLDVVHRTDKFFLGSRY**G*KR***SDVETGNAILTTVDDDDSQVTCLHTGITNLYRCFRKENVSDDTTIN*

>*Thermobia domestica* (Zygentoma) RYa [GenBank:GASN01389388.1]

***M***VTLSTSSRQLLAVSLVLVTLSSLLVASSAQQFYPNGRY**G*KR***DEHEIEVRDPSSRFYLGSRF**G*R***SGQGMDVVQRTDKFFLGSRY**G*KR***NYDGVDDAVLSSTSDDDAQVTCLYTGFSNVYRCFRKDNASEESTVN*

>*Atelura formicaria* (Zygentoma) RYa [GenBank:GAYJ01031750.1]

***M***VVLGSSARQLLTAAVLLMALLVVASAQKFYPNGRY**G*KR***AEHSVATRDSELAGRY**G*R***GSGNVVQRTDKFFLGSRY**G*KR***SDMDLDNILISSASDDDSVTCLYTGISNLYRCVRKENASEEESVN*

>*Nicoletia phytophila* (Zygentoma) RYa

***M***MVVQQVGSTKRLIAAVLMAMALWSLLLSASAQKFYPNGRY**G*KR***LEHALATRDQGSYYNGRY**G*R***GSEVVQRTDKFFLGSRY**G*KR***SDGEQGSPMVPTLVTDDMSVSCLYTGVSNLYRCSRKENGNEEDAIN*

..............................................................................................

>*Xibalbanus tulumensis* (Remipedia) RYa

***M***SSQGITMRQAVIVAMLGVIVCSLLTTAMAQSFYPNGRY**G*KR***SDHLVQERSTNRFYGGSRF**G*R***SDPEIEMHERSARVYGGSLYGHR…

>*Anaspides tasmaniae* (Malacostraca) RYa

…PQFYANRY**GR**SSPDGSPQVEIRTSHFMGGSRY**G*KR***SGDEKTPALAPATPFITPMGEDKEDASILIVGDSIVCLLVD…

>*Lithobius forficatus* (Chilopoda) RYa

***M***TTSVFRSS***M***LLGAVVMVLVSCLLPKVSDAQQFYPNGRY**G*RR***DAMPALTEISGNREMTVSFFGDGVVQCVYTGYSDYYRCSRKLGTNQPSPSVVD*

>*Hanseniella* sp. (Symphyla) RYa1

…LACLLILPISGQQFYPSGRY**G*RR***DAMPPLAEAAANREMTVAFFGDGAVRCSYTGVADFYRCHRKSESQGPAASSQ…

>*Hanseniella* sp. (Symphyla) RYa2

***M***KTSNSAVSLLLVLALVCSLIEPATFHLFYPSGRY**G*RR***NSAPPLHETAETREMSVAFFGDGTVRCAYTGVGEL…

>*Eudigraphis takakuwai nigricans* (Diplopoda) RYa

…QQFYPNGRY**G*RR***SDMPQLSDISSGREMTVAFFGDGSVQCVYTGYKDYYRCYRKHTNEPLVPSVAD*

..............................................................................................

>*Drosophila melanogaster* RYa [[FBpp0302660](http://flybase.org/reports/FBpp0302660.html)]

MNECVNKLLHLKFLFYFILGIQ***KR***PVFFVASRY**G*R***STTYDESLKS***RR***IFIVPRNEHFFLGSRY**G*KR***SGKYLCLSREINKLIVRKRLRNNDKERTPTLSFITKHFLMRNT*

>*Daphnia pulex* RYa [[Dappu1_251691](http://genome.jgi.doe.gov/cgi-bin/dispGeneModel?db=Dappu1&id=251691), GenBank:[EFX74623.1](http://www.ncbi.nlm.nih.gov/protein/321463608?report=genbank&log$=protalign&blast_rank=1&RID=YEKMXC1R015)]

MFPRRPYACAKLAYYIQQLKKPHV***M***ARKESVFWLFCTLALMMSVVLVDAQTFFTNGRY**G*KR***SEVRSRVASRSADERFFGGPRF**G*R***SGNGGIVLGNSELDA*R*NPERFFIGSRY**G*KR***SEMEQIVPSPQVDESTSNSQEKETFLECNPIGIEQLYHCIERLKSAHHFDLMQHQQV*

SIFamide (SIFa)

>*Nipponentomon nippon* (Protura) SIFa1 a

***M***FPLRSVLVPLAYLLSLVLLLSVATSDGAYRKPPFNGSIF**G*KR***SPTSGAEYDTTGRYAICEVAMEACSTWFGPVQNSK*

>*Nipponentomon nippon* (Protura) SIFa1 b

***M***FPLRSVLVPLAYLLSLVLLLSVATSDGAYRKPPFNGSIF**G*KR***SPTSGAEYDTTGRYAICEVAMEACSTWFGPAQNNK*

>*Nipponentomon nippon* (Protura) SIFa2

***M***LSNRCTLLVLAVLLVVLLSSLSSDAAYRKPPFNGSIF**G*KR***SATNSAEYDTSSRYAICEVAMEACNNWFGPIQNSK*

>*Acerentomon* sp. (Protura) SIFa [GenBank:GAXE01008643.1]

***M***SPFVRSALVPLAYLLCLVILLSSVSSEGAYRKPPFNGSIF**G*KR***SSNTNGAEYDTTGRYAICEVAMEACSTWFGPIQNSK*

>*Filientomon takanawanum* (Protura) SIFa1

***M***FTLRSVFVPLACLLSLVLLLSVATSDGAYRKPPFNGSIF**G*KR***SATSGAEYDTTGRYAICEVAMEACSSWFGPVQSSK*

>*Filientomon takanawanum* (Protura) SIFa2

***M***LSNRCTLLVLAVLLVVILSSLSSDAAYRKPPFNGSIF**G*KR***SATNSAEYDTSSRYAICEVPMEACNTWFGPIQNSKQLIYCAMFIQFLFI*

>*Anurida maritima* (Collembola) SIFa [GenBank:GAUE01049107.1]

***M***GTGKLQIVLLLFTVFGTCVFSSDGVDVTGNRKPPFNGSIF**G*KR***NFFGAGSIGEVAAPVYRSTGFGKVAMASVSASGALCEIAANYCIFQSER*

>*Podura aquatica* (Collembola) SIFa

…VLITVSAFLSPVESSSGNRKPPFNGSIF**G*KR***NAEGANVGETGSGPIYYSPNSVGSIVSKFPTGSLGALCEIAVETCSSIFIPD***KR****

>*Folsomia candida* (Collembola) SIFa [GenBank:GASX01010579.1]

***M***AKVAVIAFTIALIITFLVSSKSVEASGGYGRKPPFNGSIF**G*KR***NSEGVGASSSNGEGYGGGKSLGSLCEIAWETCSALYLETSR*

>*Bourletiella hortensis* (Collembola) SIFa

***M***GRISVILVLVAVIAVVLAVASIAEASSGAYRKPPFNGSIF**G*KR***NSDGAGGADVGGKAMSSLASLCEIAVETCTSLYVDT***RR****

>*Orchesella cincta* (Collembola) SIFa

***M***CKIAVLALALVLILTFVLSGEKGAEASGGYRKPPFNGSIF**G*KR***TSAVGADGGNFPTAPSNSGNGNRAYYNSFCEMALETCFAPYLAESAR*

>*Pogonognathellus sp.* (Collembola) SIFa [GenBank:GATD01067615.1]

***M***GKLSVIIFFFVLIATFYTNSWAEATNGYRKPPFNGSIF**G*KR***TAGADGGLQGKMSPMGSLCEIA…

>*Sminthurus viridis* (Collembola) SIFa1 [GenBank:GATZ01100071.1]

***M***GKVSLLLVIVVIAIGFFVASDLAEAASGAYRKPPFNGSIF**G*KR***SSEGATGGEGMKPISSLGSLCEIAMETCSSLYLESR*

>*Sminthurus viridis* (Collembola) SIFa2 [GenBank:GATZ01031538.1]

***M***AGSNLLLVVTVVAMGLLIATEFTEGAMDPYKQNQPPFNGGIF**G*KR***GS…

>*Jordanathrix leptothrix* (Collembola) SIFa

***M***RSVGLVGRGSVWLVVVMVVAVGLLLSSGVAEGTGGAYRKPPFNGSIF**G*KR***SGEGGVSGGGGGVGGLNGNGLGTMTMDGSKSSLGPMGSLCEIALETCSSLYLESR*

>*Campodea augens* (Diplura) SIFa [GenBank:GAYN01130289.1]

***M***SASTIRLFAFFAMLALLILSEQEVSAATFRKPPFNGSIY**G*KR***NGIGEADPAARSVAALCEIAYDACGSWLQA*

>*Lepidocampa weberi* (Diplura) SIFa

***M***SGILRCILLLAVVALVFLGEQEVAANNVRKLPFNGSIY**G*KR***TSVNDVDASSSIAALCTIAYDTCGVLLES**G*KK****

>*Occasjapyx japonicus* (Diplura) SIFa [GenBank:GAXJ01009004.1; frameshift)

***M***ASVRAFIVLAVLALVLVLSQDATAAYRKPPFNGS…

…***KR***GASSADYEGATKSLTALCEIAYDTCSAWFPPEN*

>*Catajapyx aquilonaris* (Diplura) SIFa

***M***ASVRTFILLAVVALVLVLSQDTAAAYRKPPFNGSIF**G*KR***GSSADYEGATKSLTALCEIAYDTCSAWFPPEN*

>*Pedetontus okajimae* (Archaeognatha) SIFa1

…LLFSDVTTATYRKPPFNGSIF**G*KR***GSTSTDYEGTSKSLSSLCEIALESCSAWFPATEK*

>*Pedetontus okajimae* (Archaeognatha) SIFa2

***M***RSVGLVGRGSVWLVVVMVVAVGLLLSSGVAEGTGGAYRKPPFNGSIF**G*KR***SGEGGVSGGGGGVGGLNGNGLE…

>*Machilis hrabei* (Archaeognatha) SIFa [GenBank:GAUM01180855.1]

***M***LRATVTVFLIVTAILLLSDVTSATYRKPPFNGSIF**G*KR***GSSSTDYEGTSKSLSSLCEIALESCSAWFPITEK*

>*Meinertellus cundinamarcensis* (Archaeognatha) SIFa [GenBank:GAUG01031297.1]

***M***FRSTLTLCLIVAALLLLSDVTAATYRKPPFNGSIF**G*KR***GSAATDYEGTSRTLSSLCEIAIESCSAWFPAAEK*

>*Machilontus* sp. (Archaeognatha) SIFa

***M***HRATFTFCLLIAAILLLSDVAMATYRKPPFNGSIF**G*KR***GSTQADYEGTTRTLSTLCEIALESCSAWFPSTEK*

>*Tricholepidion gertschi* (Zygentoma) SIFa [GenBank:GASO01231104.1]

***M***QKSVMCVFLLALALLLLVNPSTAAYRKPPFNGSIF**G*KR***GNVADYDGAGKALSSMCEIASEACSAWFPAPENN*

>*Thermobia domestica* (Zygentoma) SIFa [GenBank:GASN01010436.1]

***M***QKSMMYLCIMVVALSVLIQCTAAGYRKPPFNGSIF**G*KR***ADYDGTGKALSALCEVASEACSAWFPSADSN*

>*Atelura formicaria* (Zygentoma) SIFa1 [GenBank:GAYJ01286915.1]

***M***QKSVMIVCLFALAVALLVDVAAAGYRKPPFNGSIF**G*KR***DYDGAGKALSSMCEIASEACSAWFPAADN*

>*Atelura formicaria* (Zygentoma) SIFa2 [GenBank:GAYJ01215588.1]

***M***QCSAVKYFFLLVALLLLFSTTSAATYRKPPFNGSIF**G*KR***TGSQDYEVAGKALSAMCEIASEACTAWFPQVDNN*

>*Nicoletia phytophila* (Zygentoma) SIFa

***M***QKSVMLLCVLALAVVLLADSVSAGYRKPPFNGSIF**G*KR***DYDGTGKALSSLCEIASEACSAWFPAAENN*

..............................................................................................

>*Xibalbanus tulumensis* (Remipedia) SIFa [GenBank:JL108619.1] (Christie 2014)

***M***PKLAVFLCVATIALLLLADLAAAGYRKPPFNGSIF**G*KR***GSTAEYEGAGKSLYAMCEIAVEACSAWFPTADN*

>*Anaspides tasmaniae* (Malacostraca) SIFa

***M***MRMRSRLVVAIVVLVVALTLLSSPVSAGYRKPPFNGSIF**G*KR***AGQSALTAAGSESGYEPGKSLAAVCEVALEACAGWFPSVERK*

>*Lithobius forficatus* (Chilopoda) SIFa

***M***ASKLLTFLLIAAFVVVLMTDVTSANYRKPPFNGSIF**G*KR***APEDPTAEKLYAMCTIAMDACSQWFPSAEA…

>*Hanseniella* sp. (Symphyla) SIFa1

…STRIFVLAFAATLAILLLLETTEAGYRKPPFNGSIF**G*KR***AGEVSTDKIYAVCEIAIETCSQLFQSAD***KK****

>*Hanseniella* sp. (Symphyla) SIFa2

…AKVFFLVLVISVSLLLVVDVSASYKKPPFNGSIF**G*KR***AGEVSSERLYAVCEIAIDTCSQFFQMAE***KK****

>*Eudigraphis takakuwai nigricans* (Diplopoda) SIFa

***M***ASRAVVLMLCLMVLLAVMHSTQATYRKPPFNGSIF**G*KR***APGYAGGSDSASDKLYAMCTVAVETCSQLFPNSESN*

..............................................................................................

>*Drosophila melanogaster* SIFa [[FBpp0293592](http://flybase.org/reports/FBpp0293592.html)]

***M***ALRFTLTLLLVTILVAAILLGSSEAAYRKPPFNGSIF**G*KR***NSLDYDSAKMSAVCEVAMEACPMWFPQNDSK*

>*Daphnia pulex* SIFa [[Dappu1_260818](http://genome.jgi.doe.gov/cgi-bin/dispGeneModel?db=Dappu1&tid=260818), GenBank:[EFX67946.1](http://www.ncbi.nlm.nih.gov/protein/321456848?report=genbank&log$=protalign&blast_rank=1&RID=YEMGEN69015)]

***M***RSSFIVVMVCVVVVLTFWGQVAEATRKLPFNGSIF**G*KR***SNQGTDKLESPSNLQLLCDAAMNACSDWLPIGSK*

Short neuropeptide F (sNPF)

>*Nipponentomon nippon* (Protura) sNPF1

***M***TRFDVLRLSLFSCCLVLLIATQLTSAAPSDPDYGNLRELYEAILQRNALEGGGGQHDLV***RK***SNRSPSLRLRF**G*RR***ADPYWQESGMDKMAPSALD*

>*Nipponentomon nippon* (Protura) sNPF2

***M***TRLDILRASLLSLCLVLIVTTEVVRAAPSYSDYGNLRELYEAILQRNALEGGGGGFGHDVV***RK***SNRSPSLRLRF**G*RR***ADPYWQESGLDKVAPSSLE*

>*Acerentomon* sp. (Protura) sNPF [GenBank:GAXE01001478.1, GAXE01001479.1]

***M***ARIDLATLSLISFCLVLLFTTDSTMAAPSDPDYGNLRELYEAILQRNALEGGSPHDLV***RK***SNRSPSLRLRF**G*RR***ADPYWQESGLDKLVPSSLE*

>*Filientomon takanawanum* (Protura) sNPF

***M***SRIDVLRLSLVSFCLVLLITTELTLAAPSDPDYGNLRELYEAILQRSALEGGGGSHDLV***RK***SNRSPSLRLRF**G*RR***ADPYWQESGMDKMAPSALD*

>*Anurida maritima* (Collembola) sNPF [GenBank:GAUE01011070.1]

***M***EWKKSVDLFALSLLWMFLILSQAVAFPAPESSLGSAYSLDDYEQPQQPGLREIFEELERQGGLTGPIMPRSGSYYVLPNRQMA***RK***SQRSSLRLRF**G*KR***SLMTGERKGENPISWKGREIPDEVKLD*

>*Tetrodontophora bielanensis* (Collembola) sNPF [GenBank:GAXI01148414.1]

***M***MEHQKLIQIFGIGLCLLVVITEITSLPFYTPEDYEQVAGIREILQEIDRQGGLTSAMIPRAYHPNHRMM***RK***SQRSNVLRLRF**G*KR***SVDYPKNYIDDVDWPENTELQQRRKRK*

>*Podura aquatica* (Collembola) sNPF

***M***NSFKFGKSMQVLGSIALLLFLISQISAFPFTGIDRTGYLSGEEYDQSGLREVLEELERQGGLTGQIVQRGSNYANSFPNHKMM…

>*Folsomia candida* (Collembola) sNPF [GenBank:GASX01087802.1]

…EYGKTIQIIGVVLCVLLVVTEIYSLPAYSSEDYDQGVREMMEELERQGLTGPMVGRAYHPNHNMM***RK***SQRSNNLRLRF**G*KR***SVPDAVPINTMFKPLDDAQKAE*

>*Bourletiella hortensis* (Collembola) sNPF

***M***ESRRAVQFFGLAFCLLVVVAEIYSTPAYTPEDYEQGVREMMEELERQGLTGPIVPRAYPHHMM***RK***SQRSNSLRLRF**G*KR***SGETSMGWKSADDALPGQD*

>*Orchesella cincta* (Collembola) sNPF

***M***ESKRTVQIAGVVFCVLLVVSEICCYPPTYSHEDYEQGVREIMEELERQGLTGPMVPRAYNPAHRMM***RK***SQRSSNLRLRF**G*KR***SSPPFEGEQVEAIGNPNWKAGSDVQRED*

>*Pogonognathellus sp.* (Collembola) sNPF [GenBank:GATD01101094.1]

***M***ESRKMVQAFGIAFCLLILVSEILSSPSYTPEDYEQAGIREMLEELERQGLTGPMVPRAYKGSNPNHAMM***RK***SQRSNSLRLRF**G*KR***SPVVPDVPILWKTMDEENKED*

>*Sminthurus viridis* (Collembola) sNPF [GenBank:GATZ01013306.1]

***M***ESRKAMQFFGLAFCLLLVVVEIYSLPAYTPEDYEQGVREMMEELERQGLTGPIAPRAFPNHHMM***RK***SQRSNSLRLRF**G*KR***SGDNSVVWKTSDDVNGQD*

>*Jordanathrix leptothrix* (Collembola) sNPF

***M***ESKRTMQFFGLAFCLMLVIVEIYSVPLYTPDDYEQRVQDMVVELERQGLTGPMLNRAFPNDHMM***RK***SQRSNSLRLRF**G*KR***SGDASEAWKAANQQPED*

>*Campodea augens* (Diplura) sNPF [GenBank:GAYN01138702.1]

***M***KGFSAIRCCTMALCVLVVFADFITAAPPAYSDYDSLRELYELLQKDAIASRLAGHEIV***RK***SNRSPSLRLRF**G*RR***ADPLWQDGTFNEAGTAAAEN*

>*Lepidocampa weberi* (Diplura) sNPF

***M***KSISAVRCCTMALCIVLVLADFITAAPPAYSDYDSLRELYELLQKDAIASRLGGHEIV***RK***SNRSPSLRLRF**G*RR***ADPLWQDGSFNEVGGTSASEN*

>*Occasjapyx japonicus* (Diplura) sNPF [GenBank:GAXJ01110623.1]

***M***RVTWAAGYCTLALCLLMLVADQVATAPYSDYDSVRDLYDMMQKEAQAARFGHDIV***RK***SSSNRGPSLRLRF**G*RR***ADPLWQDNSFDEAATAGSEN*

>*Catajapyx aquilonaris* (Diplura) sNPF

***M***RATSAAGYCTLALCLLVIVADLIAAAPPYSDYDSLRDLYEMLQREALASRMGHEVV***RK***SNRSPSLRLRF**G*RR***ADPLWQQMQDNTMDEAGTTGSEN*

>*Pedetontus okajimae* (Archaeognatha) sNPF

***M***KASLVMTGCAVAMCLLIITCHTSTAAPAYPDYENVRDLYELLLQKEALADRLGHEVV***RK***AERSPALRLRF**G*RR***ADPAMQRISAPSSDVADN*

>*Machilis hrabei* (Archaeognatha) sNPF [GenBank:GAUM01179765.1]

***M***KASIIMTGCAVALCLLLVTCHTSTAAPAYPDYENVRDLYELLLQKEALADRLGHEVV***RK***AERSSPLRLRF**G*RR***ADPAMQRLSVASGDVADN*

>*Meinertellus cundinamarcensis* (Archaeognatha) sNPF [GenBank:GAUG01239216.1]

***M***KASLMVSCCAVAMCLLLITCQSSTAAPAYPDYESARDLYELLLQKEALADRLGHEIV***RK***SNRSPSLRLRF**G*RR***ADPAMQGLSEPSADAAEN*

>*Machilontus* sp. (Archaeognatha) sNPF

***M***KATLVASYCAVAMCILLITSPSSTAAPAYPDYENARDLYQLLLQKEALADRLGHEIV***RK***SNRSPSLRLRF**G*RR***ADPSLQGLSEPSIDAADN*

>*Tricholepidion gertschi* (Zygentoma) sNPF [GenBank:GASO01252803.1]

***M***SGFSVMKCFTVALCLLIVVAEISSAAPSYSDYENVRDLYDLLLQKEALEDRLGHEVV***RK***SNRSPSLRLRF**G*RR***ADPMMQGASFAEHPVETTEK*

>*Thermobia domestica* (Zygentoma) sNPF [GenBank:GASN01393471.1]

***M***KGLNVTKCCTIALCILIVVAEISTAAPSFSDYENVRDLYELLLQKEALEDRLGHEVV***RK***SSNRSPSYRLRF**G*RR***ADPMMQGNQFSEHAAEAADN*

>*Atelura formicaria* (Zygentoma) sNPF [GenBank:GAYJ01028431.1]

***M***KGFNVMKCSTVAICLLVLVAEIACAAPSYSDYENVRDLYELLLQKEALENRLGGHEVV***RK***SSNRSPSLRLRF**G*RR***ADPLMQSTAFADRAPEAADN*

>*Nicoletia phytophila* (Zygentoma) sNPF

***M***KGFSVMKYSTVAICLLVLVAELTWAAPSYNDYDNVRDLYELLLQKEALENRLGHEVV***RK***SSNRSPSLRLRF**G*RR***SDPMLQSTSFADHPAEAAEN*

..............................................................................................

>*Xibalbanus tulumensis* (Remipedia) sNPF

***M***QGSLVVKLCAVFLCTMVLVSEIVSAAPTYSDYENIRDLYELLLQKEALADRLGHEVV***RK***SGRSPSLRLRF**G*RR***ADPYWQESSFDAASSSSAD*

>*Anaspides tasmaniae* (Malacostraca) sNPF

…PQLRLRF**G*KR***SGEEVEVATHSMV***RK***DDRSPALRLRF**G*KR***EDSFDQESGDVASQEK*

>*Lithobius forficatus* (Chilopoda) sNPF

***M***GSTSVLRCCAIVLVLAVLTAELTAAAPSFGDYDNIRDLYELLLRNEARGDRLSHQVV***RK***GGRDPSLRLRF**G*RR***ADPAWQSLVSSESGDTN*

>*Hanseniella* sp. (Symphyla) sNPF1

***M***TSSLLMKCCAVTLVTLLVIGELTNAVPSNYGNDYEGLKDLYETLLKNELMNERSSHQLV***RK***ASRDPSLRLRF**G*RR***ADPAWQQLDDSQAESLSKN*

>*Hanseniella* sp. (Symphyla) sNPF2

***M***TSSLAVKCFAVTFVALLFIGELASGAPGPAYGDYEGLKELYDLMLRNEMFNERSRHQMV***RK***AGRDPSLRLRF**G*RR***ADPTWQQYGPEESQAEGTSTN*

>*Hanseniella* sp. (Symphyla) sNPF3

***M***TTSLLLKCMAVTFVALLVIGELANSAPSAYADNDGLKELYEMLLRNELFSERSHHQLV***RK***AGRDPSLRLRF**G*RR***SDPLWQQYGPEENQSDNTATN*

>*Eudigraphis takakuwai nigricans* (Diplopoda) sNPF1a

***M***ACKVNSITCVVALVCIMMVVSELASAAPAYDAENIRDLYELLLRNDLLGDRYPHQLV***RK***AGRDPAVRLRF**G*RR***ADPYWQNFPISADGPSSAENTSN*

>*Eudigraphis takakuwai nigricans* (Diplopoda) sNPF1b

***M***ACKVNSITCVVALVCIMMVVSELASAAPAYDAE*RWRPTPQHIRDLVFHLHRQGIHPSTLQ*NIRDIRDLYELLLRNDLLGDRYPHQLV***RK***AGRDPAVRLRF**G*RR***ADPYWQNFPISADGPSSAENTSN*

..............................................................................................

>*Drosophila melanogaster* sNPF [[FBpp0080859](http://flybase.org/reports/FBpp0080859.html)]

***M***FHLKRELSQGCALALICLVSLQMQQPAQAEVSSAQGTPLSNLYDNLLQREYAGPVVFPNHQVE***RK***AQ***R***SPSLRLRF**G*R***SDPDMLNSIVE***KR***WFGDVNQKPIRSPSLRLRF**G*RR***DPSLPQMRRTAYDDLLERELTLNSQQQQQQLGTEPDSDLGADYDGLYERVV**RK**PQRLRW**G*R***SVPQFEANNADNEQIERSQWYNSLLNSDKMRRMLVALQQQYEIPENVASYANDEDTDTDLNNDTSEFQREV**R**KPMRLRW**G*R***STGKAPSEQKHTPEETSSIPPKTQN*

>*Daphnia pulex* sNPF [[Dappu1_299864](http://genome.jgi.doe.gov/cgi-bin/dispGeneModel?db=Dappu1&id=299864), GenBank:[EFX90018.1](http://www.ncbi.nlm.nih.gov/protein/321479062?report=genbank&log$=protalign&blast_rank=1&RID=YEN446Z0015)]

***M***ELCPRINCWTTRTVLLVTFVVFLIHQDIQQNIASASPTPLLSGFEDYSEDRLNGEQPSLYELLLQREMLADKLDSEGRGHLIV***RK***SDRSPSLRLRF**G*RR***ADPDVPRVSAASNQHD*

Sulfakinin (SK)

>*Nipponentomon nippon* (Protura) SK1

…GAPKTNRELAHLASLMAPYLNSVGGKLLKESRDTGGEAADDEAEAFEES***KR***FDDYGHMRF**G*KR***QN**G*KR***EFDDYGHMRF**G*KKR****

>*Nipponentomon nippon* (Protura) SK2

…SIPSPSLISLTASILVSLLVVGLTSAESSKGVQEQQGQVTSGVISAPKS***RR***ELAHLASLMAPYLSRVATG***KK***E***RR***QQEMALVQLVGSDSGAAAGGDFEDPEMVEE***KR***EFDDYGHMRF**G*KR***QFDDYGHMRF**G*RKK****

>*Acerentomon* sp. (Protura) SK1 [GenBank:GAXE01001454.1, GAXE01001451.1, GAXE01045731.1]

***M***QFSYFPHHSLTTLVSSLLLTMLILGHPSSADTKPLQQTRPNAIAAPKPNRELAHLANLIAPYWNSVGGKSRDFGGGEDFEDSDLFEE***KR***QFDDYGHMRF**G*KR***TNGV***KR***EYDDYGHMRF**G*RR****

>*Acerentomon* sp. (Protura) SK2[GenBank:GAXE01033680.1]

***M***QFSYFPHHSLTTLVSSLLLTILILGHSSAGNKPIQQTRPNAIAAPKPNRELAHLANLIAPYWNSVGGK…

>*Filientomon takanawanum* (Protura) SK

***M***KLCPPHWLSTVMASLFLTMLLAGLTSAQPKSPPQTQSNAIGAPKTNRELAHLASLMAPYLNSVGGKLIKDPRDNEAAADFEDADAFEES***KR***FDDYGHMRF**G*KR***QN**G*KR***EFDDYGHMRF**G*RKR****

>*Anurida maritima* (Collembola) SK [GenBank:GAUE01050644.1]

***M***KSFSVLVVVVVAVSCLMPIIFGEGAKTISSQQQSP***KK***MRISGSTATRIYRLLHSGGINEGPGFEDEDIFIDDDNAIQ***KR***QYDDYGHMRF**G*KR***QSKGDSDDYGHLRF**G*K****

>*Tetrodontophora bielanensis* (Collembola) SK [GenBank:GAXI01001089.1]

***M***FKLIIFIALVIVVYSCCLATGFSQPSGQSSLMQGGGQTQSTLP***KK***VRVTNNPNTNRVYHLLRNSVNPGDDDDMFIDEDNFQ***KR***NFDDYGHMRF**G*KR***QTGGRDFDDYGHSRF**G*KR***GGGGGQTDDYGIGLRF**G*K****

>*Podura aquatica* (Collembola) SK

***M***SKIALGSLIFVLYLFFLTEIYSEAASVVGTSQNAIGQQNQRKVRVLSPALSRLYRLLRSSGSGAEDDDLFLDDESSGMQ***KR***QYDDYGHMRF**G*KR***QSKSGDFDDYGHLRF**G*K****

>*Folsomia candida* (Collembola) SK [GenBank:GASX01086603.1]

***M***SKFTVFATIFVLYACAVALGLGDPSSSNLGGGLPGVVSPKNVRILSPAATRLYRMIRKGQLDDDDLFAEDENNFR***KR***QYDDYGHMRF**G*KR***NQPSKDFDDYGHLRF**G*K****

>*Bourletiella hortensis* (Collembola) SK

***M***RTAIVLVLVVAVYSACFYRASSERATNLASASSSG***KK***IHLTPTASRLYHLLRAGISNSVDDDDLQSDDDNFVQ***KR***QYDDYGHMRF**G*KR***GSSAKDFDDYGHLRF**G*K****

>*Orchesella cincta* (Collembola) SK

***M***CSIRLIAMVLLAYTVCTALCDPINGGGGSGTAPSAPHPNARKLRVLNPGLSRMFRMLRSTGISDDDEVLVDDDGYFQ***KR***QYDDYGHMRF**G*KR***NPKDFDDYGHMRF**G*K****

>*Pogonognathellus sp.* (Collembola) SK [GenBank:GATD01015047.1]

***M***SKLTIFLSIVIVYFCCIAIGYGDSTSGPSTNSGVSA***KK***VRVLSPSASRLYRLLRNGLNQIGDEDLISEDELFLQ***KR***QYDDYGHMRF**G*KR***TNDFDDYGHLRF**G*K****

>*Sminthurus viridis* (Collembola) SK [GenBank:GATZ01100504.1]

***M***RNITILALALALFSICLLKANCERSGTTNSVSGA***KK***IRPLNPSATRLYHLLRLGMNGVDDEDLQSDDDNFVQ***KR***QYDDYGHMRF**G*KR***NPPKEFDDYGHLRF**G*K****

>*Jordanathrix leptothrix* (Collembola) SK

***M***SNTFLFVFVLVIYSSCIILGNCENLGRSSSSSNSVSAS***KK***LRVLNPSAARLFHLLKLGMGGSGSVNVEDEDLQSDDDNFVQ***KR***QYDDYGHMRF**G*KR***NPPSKEFDDYGHLRF**G*K****

>*Campodea augens* (Diplura) SK [GenBank:GAYN01127190.1]

***M***RPLSLVVVLYTSLFLVLLTVECNGSPLKHRGINSFVDIFRNMFARNRQEGEGLDWEDPEDLHDSD***KR***QADDYGHMRF**G*KR***ADFDDYGHMRF**G*R***SSIPVTVERHHV*

>*Lepidocampa weberi* (Diplura) SK1

***M***RSSHLVPMASCTVFIFLLLSCQTDVTSSTQHHRGVNSLAEFL***KK***IIGSRQIPDPIHPSPGSSEKENVDWEDTEDFHEAD***KR***QQGDDYGHMRF**G*KR***DFDDYGHMRF**G*R***SLTKL*

>*Lepidocampa weberi* (Diplura) SK2

***M***QLLNMRPSPLAILCVHSLLLFLLYTSQSEASLQQHHGNQSILDLVRNIFLG***RR***PNPQGSASLSSDKNLNDEFHDSD***KR***QDDDYGHMRF**G*KR***DFDDYGHMRF**G*R***SPQEKA*

>*Occasjapyx japonicus* (Diplura) SK [GenBank:GAXJ01097089.1]

***M***RWLVVLASAASLAVVLVLPSPTMGRPPASQQQQQGAQGQHSSPQQPSPRDLARWASLVVPYLSRDIVGAPPPVLDAVVEEVAEDPRGSSD***KR***QFDDYGHMRF**G*K***…

>*Catajapyx aquilonaris* (Diplura) SK

***M***RLLVVVASVALMSVLVPSQTLGRPQGTQVNPSHSAQQPSSRDLARWASLVVPYLSRDMAAPPLIEEEVAEDPRGSSD***KR***QFDDYGHMRF**G*KR***EFDDYGHMRF**G*R***SVD*

>*Pedetontus okajimae* (Archaeognatha) SK

…FISVMTLLLFYVQDSPRQANQLRPLLRDLSEIEAANEME***KR***QFDDYGHMRF**G*KR***EFDDYGHMRF**G*R***SSPDEA*

>*Machilis hrabei* (Archaeognatha) SK [GenBank:GAUM01021126.1]

***M***RPSLLFVSVMTLLLFCVQASPRQAVQLRPLLRDLTELEAANEME***KR***QFDDYGHMRF**G*KR***EFDDYGHLRF**G*R***SSPDDA*

>*Meinertellus cundinamarcensis* (Archaeognatha) SK

***M***YESFLDNDPKMRLSLFLLPLMTILLWVEASPRQTQQLRPSLRDLAELEVPNEYE***KR***QFDDYGHMRF**G*KR***EFDDYGHMRF**G*R***SSSDDA*

>*Machilontus* sp. (Archaeognatha) SK [GenBank:GAUG01245048.1]

***M***RPSFLLLSLMTYLLLVEASPRQATQLRPLLRDLAELEAVNEIE***KR***QFDDYGHMRF**G*KR***EFDDYGHMRF**G*R***SSTSDA*

>*Tricholepidion gertschi* (Zygentoma) SK [GenBank:GASO01228371.1]

***M***TSRLPLMTMMILTAAHFLQPVRTIPVSAASTVTRGQRDLGRVSNLLGSYVRAHGQVHPPPIQAEDIGIASDLLEDLDLNEE***KR***QFDDYGHMRF**G*KR***EFDDYGHMRF**G*R***SVSRDK*

>*Thermobia domestica* (Zygentoma) SK [GenBank:GASN01335973.1 + PCR full-length KT152027]

***M***RSTMMLLTIVMVLLGIHLLTVHCAPGSSPTTGA***RR***ERDLARLSNLLGSYPRSHRQLHPPPIQTDDPTLTGDILDDLDINEE***KR***QFDDYGHMRF**G*KR***EFDDYGHMRF**G*R***SANDQ*

>*Atelura formicaria* (Zygentoma) SK [GenBank:GAYJ01286450.1]

***M***VRVVPLLMVCSLVLVQAIPQTRSADSLTSQTGVRSQRDVTRLASLLAPYPRIHMGHTPPGQGDDGDLFDDLDINEE***KR***QFDDYGHMRF**G*KR***EFDDYGHMRF**G*R***SVASH*

>*Nicoletia phytophila* (Zygentoma) SK

***M***KSALVILVSLLFLLLVHVLSPVNCTSRPAPKTAPRNERDVSHLSSLLTPFARSRGKIHSPPIPADDLTISDDILDDLDINEE***KR***QFDDYGHMRF**G*KR***EFDDYGHMRF**G*R***SVDDQ*

..............................................................................................

>*Xibalbanus tulumensis* (Remipedia) SK

***M***GMSLVYSSVTVFVLLCTCAYMTSASHPSRKMDLSRLTNFIVPYLEAHSKPDLPPVKQSMRTPVAPAPVDEGNDFEDPDMMKFHGAE***KR***QFDDYGHMRF**G*KR***DFDDYGHMRF**G*R***SAVEE*

>*Anaspides tasmaniae* (Malacostraca) SK

***M***MVSSACWALVWVLATTVWCGGVSAAPSRHTASNMLITPTLRHKLEEGRLPAALLEELVADFEDPELMNFHDTPE***KR***QFDDYGHMRF**G*KR***ADFDDYGHLRF**G*R***STVEARNKH*

>*Lithobius forficatus* (Chilopoda) SK

…DDVDDFEDPDWMDFHDNP***KR***QYDDYGHLRF**G*KR***ADFDDYGHMRF**G*R***STKTN*

>*Hanseniella* sp. (Symphyla) SK1

…SDDYGHLRF**G*KR***ADFDDYGHMRF**G*R***SV*

>*Hanseniella* sp. (Symphyla) SK2

…VVTTGNCKATDS***KR***THRYPHPMGPFLYSEGFQQRLREAIGKNKENSLNTNAEDFDDMDSNELV***KR***QSDDYGHLRF**G*KR***VAFDDYG…

>*Eudigraphis takakuwai nigricans* (Diplopoda) SK

***M***MKLYVIATLVTLVVCLVRSNDTAAVHRNTDATYVRYGRLLIPYLTERDA***RR***WGPGGIRKPSPNSSTHQEVQATATDDANEEGEYPDLLDVNMPS***KR***QYDDYGHLRF**G*KR***DFDDYGHMRF**G*R***STQ*

..............................................................................................

>*Drosophila melanogaster* SK [[FBpp0078628](http://flybase.org/reports/FBpp0078628.html)]

***M***GPRSCTHFATLFMPLWALAFCFLVVLPIPAQTTSLQNAKDD**RR**LQELESKIGGEIDQPIANLVGPSFSLFGD**RR**NQKTMSF**G*RR***VPLISRPIIPIELDLLMDNDDERTKA***KR***FDDYGHMRF**G*KR***GGDDQFDDYGHMRF**G*R****

>*Daphnia pulex* SK [[Dappu1_242979](http://genome.jgi.doe.gov/cgi-bin/dispGeneModel?db=Dappu1&id=242979), GenBank:[EFX80896.1](http://www.ncbi.nlm.nih.gov/protein/321479062?report=genbank&log$=protalign&blast_rank=1&RID=YEN446Z0015)]

***M***PIIRQSSVKTLNLLLYIVRVVCAVDGQEEAQQQQQQQHRMKLTMLATVLAAVLVLGVGRATAAPADSSSTATGRRLLHSPNPTSHSKSIDSWLRWLLLRSRIGDKEKTKNGVPSNSFQLARSPVELGSSNPKLQAKLPPAIVQSNDDDETTGFGDEDFADEDVPLVLPEGRQAAS***KR***QPDDYGHMRY**G*KR***DFDDYGHMRF**G*RR****

Tachykinin-related peptide (TKRP)

>*Nipponentomon nippon* (Protura) TKRP

…LRPGSWPVRGRVDTPLFDPSLLESEFEME***KR***APSGFQGMR**G*KK***PDPLGWGSISGGGKMRSFHDPEDSFD***KR***APSSQFLGMR**G*KK***FQGLEPSGVESEQDIDALVRFLLY**G*RR***QGFDR***KR***APSSAFYGMR**G*KK***ASLPAAQFPSQHQFSPLLPNSNAAADLWAV***KR***APSSNSGFFGVR**G*KR***STSHNGRS***KR***ETDILDSDWFPNADEERDFDA*

>*Acerentomon* sp. (Protura) TKRP [GenBank:GAXE01022709.1]

***M***DSWKFLLVVCAHIVCAISLQSPEI***KR***APSGFNGVR**G*KK***DSPEMETFPGGDFSDGPAVILGYQDTDLD***KR***APSGFQGMR**G*KK***LDGEGPFADDSIAQAFGSFYDSDFD***KR***APSAQFLGMR**G*KK***LMDPDSEPDFEALVRLLLS**G*RR***QGGFDR***KR***APSSAFYGMR**G*KK***SFEQHSLPASANSSPDLIYSL***KR***APSGSGFFGVR**G*KR***SNSNNLHGRSSDDVVKSPNSDWLSRHDQERNLNA*

>*Filientomon takanawanum* (Protura) TKRP

***M***DSWKLLVVIAAHFACASSLQSPED***KR***APSGFQGMR**G*KK***QDTAIESFANSEYPEGPPVLVGYSEGDLD***KR***APSGFQGMR**G*KK***PDPEDPWNDEIRSRLAPAFRAYYENDLE***KR***APSSQFLGMR**G*KK***LMDDSEPDLESLVRFLLS**G*RR***QGTYDR***KR***APSSAFYGMR**G*KK***SYEQVANSNPELLWSL***KR***APAGAGFFGVR**G*KR***STSNFFGR**G*KR***AAADNPSPVEWAVLRDEERSFDA*

>*Anurida maritima* (Collembola) TKRP [GenBank:GAUE01051522.1]

…LAIVLAFDHGKLPLDPSRSNKAQVLVNPYFSSVGEGNYQDYYDLPRQTDSLW***KR***VPSAGFYGMR**G*KK***APSRNFYGVR**G*KK***GPIGFLGVR**G*KK***MDGKVEENLLEEGGERPWMSKEILPSGQVGGD***KR***VQDGHGEDVPWE***KR***VPTQGFMGLR**G*RR***SLQNGYDPLYKLYRLH*

>*Podura aquatica* (Collembola) TKRP

***M***YMSSLLLFCIESGVAVPPLQESSNNLGEEASLLGEETHFYNTQLSPITPFDFYQMPKSYEGW***KR***APSVGFYGMR**G*KK***VPVSNFYGMR**G*KK***GPAGFMGMR**G*KK***NSEHLDENLEEEEFKWKTPLFSPSSYT…

>*Folsomia candida* (Collembola) TKRP [GenBank:GASX01024080.1]

…ENPRNYFQFFHDMEPW***KR***TPSAGWFGMR**G*KK***VVGSNFFGMR**G*KK***GPSGFLGMR**G*KK***MDPFPQQAAIADDTTSNELY…

>*Bourletiella hortensis* (Collembola) TKRP1

***M***KLPICVAGPTLLCTTLLSTFCLVLCFDADGDRQQQHLPSSLKESADHAPWSTLNDRVALKELYEMYRDGEPLW***KR***APAAGFFGMR**G*KK***VPGQNFYGMR**G*KK***GPSGFLGMR**G*KK***MMAESKLARLESPLLDTEDLWVSKELVEGLDEGRQNSQHQLLGDNLPIDHVDD***KR***APANGFMGLR**G*KR***NASPKDLAAPSSTTSNTGDEASRTSGH*

>*Bourletiella hortensis* (Collembola) TKRP2

***M***RKMAEGLSLLVVTFVVFFSSNPVVTVTSLSIDRNLASTVSTVTELEDPVLMGYSAPFGQLYDVEVDGFHEA***KR***APAAYGFYGMR**G*KK***APSMSFYGMR**G*KK***ASFPQVDQSGSGRILDDEPALWIQNGGRGQQGGEVERENGEILIEDSGLLGGGGEHSDPRTILLVTESE***KKR***APSNGFLGLR**G*RR***MMSPILF*

>*Pogonognathellus sp.* (Collembola) TKRP [GenBank:GATD01097045.1]

***M***KLMISSIDATLVCTILVSTLCLALCSDSESMRPPDPPAVSAQDHTFWPTLNDRILKDYYNEMYKGADPIW***KR***APSSGFYGMR**G*KK***VPASNFYGMR**G*KK***GPSGFLGMR**G*KK***MDGSAKFALNGDSETLDNLLEDLWASKESQQGLDLPDKLYNYHLDVN***KR***APADGFMGLR**G*KR***NASP***RR***FYSQTSH*

>*Jordanathrix leptothrix* (Collembola) TKRP

…***KK***SPSTGFYGVR**G*KK***APDNGFYGV***RRKK***LVVQIPSQYFNHGEEEQGYFHGNPHLHPGLTQGNLAFLSQQAQAQRANEEQENNYNKDVNGVENKNKESADATSTNNGAEAV***KR***APSDGFMGLRE***KR***NNDQIIPT*

>*Lepidocampa weberi* (Diplura) TKRP

***M***LKVQDMETLLLILLACGISSLWAGTIQPPPSDTQTLFHHINGGPE***KR***DVSQHIHDGSIRDRKAMSSGFLAMR**G*KK***GSWGADDAIPRALYEELLA***KR***APSSGFFVMR**G*KK***TPDRFFGMR**G*KK***SGFDAEDRNGLEEENEPEVDVIPLSRQEMASFLSELQRQRLEGDSSLDSSGLHSEGGYLHLR**G*KR***NAYSVHPRAGSLLGSSNGHQDQMIP*

>*Occasjapyx japonicus* (Diplura) TKRP [GenBank:GAXJ01007845.1]

***M***ARLILAMALALVVVLLAAAGPSLAQTGAPHSEADSSWRDRQTQEQLQAGPE***KR***DLGSQLKEEERERKALSGFFGSR**G*KK***MQNPAFLTAYGTRLGFLNPYSRSLVDEEDFA***KR***APSGFFGMR**G*KK***APSGFFGSR**G*KK***APSGFLGMR**G*KK***DDGSYLENELDRSGSMEALPWSQDMANWLNQMHQLEANYDSTGMGSGGESAADPVDDQNLHFLLEA***KR***APNGFLGMR**G*KR***TAKGSIRFAPVGPKNAVGEDRA*

>*Catajapyx aquilonaris* (Diplura) TKRP

***M***ARLSVTLSWILVMLAAVGPSLAQTEAPHSKHSSWIERQIDQLQPGLTLAGHPESSGPA***KR***DLGSQLKQEEDRVRKDLSGFFGSR**G*KK***MLHNPALLSRLAGSFLNPYVDEEV***KR***APSGFFGMR**G*KK***APSGFFGMR**G*KK***TPSGFLGMR**G*KK***SEEDGMYPMDQFDRSRLAAMLPWSQDVSNWLSQMHQLESNYDSTGMGSGGEDPVDPSDDQNLHFLMEA***KR***APNGFLGMR**G*KR***TAQGFIRIAPLGPKNADEKDHA*

>*Pedetontus okajimae* (Archaeognatha) TKRP

***M***KGKRMLSLTALLLAIMTVLLMADPARGQSED…

…PWKAHGASSIVTTDAKNLDTQRKSDSTSVDPSHL*

>*Machilis hrabei* (Archaeognatha) TKRP [GenBank:GAUM01183514.1]

***M***KGKRALSLTALLLAVLTLLLLADSARAQSED***KR***GGPSGFMGMR**G*KK***DEGGTEGESGLD***KR***APSGFMGMR**G*KK***EGLEGSFVPYSAGQDYVLAGDKSTSPNSGFYEMR**G*KK***APSGFLGMR**G*KK***ESETSDEDSDDGDGKEGDYSTMWQPGSVQDDADAWDGYLTEES***KR***ARSGFMGMR**G*KK***KTAAGYYRYR**G*KK***APSGFMGMR**G*KK***SPSGFMGMR**G*KK***DEDDNETGHSFDDIDSLLSFLINANAEAYQRNLLNTDMHIPQ***KK***GPSGFLGMR**G*KR***WIPQGASSFVTTDVTNPEPQRKSDSSSLSSSHL*

>*Meinertellus cundinamarcensis* (Archaeognatha) TKRP [GenBank:GAUG01101837.1, GAUG01073893.1]

***M***SSLLTLVLSMVTLFLVASPVLGQED***KR***APSAGFMGMR**G*KK***DDTVADTEGTLD***KR***APSGFMGMR**G*KK***EGLQK…

…ADEDDNDTSNGFEDIDSLLSFLMNNDPEEIQRQLEA***RR***LYSPD***KR***APAGFLGMR**G*KR***WSPWSVHGVGDVVA**G*RR***GG…

>*Machilontus* sp. (Archaeognatha) TKRP

***M***YTSGKMTLSLITLLIIASSALGQIED***KR***APSGFMGMR**G*KK***DGASSDTEAGVT…

>*Tricholepidion gertschi* (Zygentoma) TKRP [GenBank:GASO01258119.1]

***M***EQQRSLLLPLLLMVVSLSVLPPSSADEL***KR***APSGFMGMR**G*KK***DRDDDFLED***KR***APNGFFGVR**G*KK***ENGPEPLSEDYYLPYDED***KR***SPSGFFGMR**G*KK***APAGFLGMR**G*KK***EFIPGYLADQDGDEEDDWEQMTAFGPDIWSSDNS***KR***APSGFFGMR**G*KK***VPSGFFGLR**G*KK***VPSGFFGMR**G*KK***APAGFLGMR**G*KK***DSEEEENLDALLYYLLANRDPEDSVEDIRTV***KR***FQHD***KK***APSGFLGMR**G*KK***EAAWSDTELGTEGSNDLENEGKDHLITNSQ*

>*Thermobia domestica* (Zygentoma) TKRP [GenBank:GASN01005970.1]

***M***NIQMIILLLLASIALSVLAQSSSEDVN***KR***APSGFLGMR**G*KK***DPSDDYSSL***KR***VSNGFYGVR**G*KK***EDELEPFGDNSDYYLSYDD***KR***AAPSGFMGMR**G*KK***APSGFLGMR**G*KK***EPYDSGYSLSRDDGDAADLGLFDHDAYD***KR***APAGFFAMR**G*KK***VPSGFFGMR**G*KK***VPSGFFGMR**G*KK***APSGFLGMR**G*KK***DGQEEDVNLDALLYYLLNGSNEDYEIPSVDTFSQSQID***KR***AQ***KK***APSGFLGMR**G*KK***DSWSDSDSDNIEGGAPKDTNQLVAGNQ*

>*Atelura formicaria* (Zygentoma) TKRP [GenBank:GAYJ01314896.1]

***M***YNQMSPLSLLLVVVSLSVLAHGSSADVE***KR***APSGFLGMR**G*KK***DHDEEFSED***KR***IQSGFVGVR**G*KK***EFDSEPFNEDYYLPYDED***KR***AAPSGFFGMR**G*KK***APSGFLGMR**G*KK***DYLEGGDDNYPEEWGPMGYYGPDFWASD***KR***APLGLFGTR**G*KK***KVPSGFFGMR**G*KK***GQMGFLGMR**G*RR***VPAGFLGMR**G*KK***DSTEPEDEDLDALLYYLLGEDHGQVD***KR***GPAE***KK***APSGFLGMR**G*KK***DTSWAA…

>*Nicoletia phytophila* (Zygentoma) TKRP

***M***EHFSVVALLVCVSVLAHTSADDD***KR***APSGFLGMR**G*KK***DDFAED***KR***APSGFLGMR**G*KK***NPDTDDDYYLYEED***KR***APSGFLGMR**G*KK***APSGFLGMR**G*KK***EFADYMDETGPVDMYGADLWEDD***KR***AAPAGFFGMR**G*KK***VPQGFFGMR**G*KK***APSGFLGMR**G*RR***IPSGFLGMR**G*KK***DARDEDLDSLLYYLLGDNYDAIDDSPVD***KR***FAAN***KK***APSGFLGMR**G*KK***SVPWAEGSESSGNIVEVKDSSQLHAGSQ*

..............................................................................................

>*Xibalbanus tulumensis* (Remipedia) TKRP

***M***KTMRKHSALCLMPLALTIIGLAHSQDTD***KR***APSGFLGMR**G*KK***DAVDEYSGNVD***KR***APSGFLGMR**G*KK***DSDDVASSDDLEAD***KR***APSGFLGMR**G*KK***APSGFLGMR**G*KK***APSGFLGMR**G*KK***APSGFLGMR**G*KK***DNDMYPEYENGYDEMNEYE***KR***APSGFLGMR**G*KK***APSGFLGMR**G*KK***APSGFLGMR**G*KK***APSGFLGMR**G*KK***DDEEESNTQELDALLEYLLQEELGKED***KR***APSGFLGMR**G*KK***MTYEGADSFDED***KR***APSLGFSGVR**G*KK***DGMDED***KR***APSGFLGMR**G*KR***SIDDALLPAESDSLLDETAVNRAPRVSRFYAGR**G*KR***SPSENTDETKPDES*

>*Anaspides tasmaniae* (Malacostraca) TKRP

***M***TIRWLAAILVVGLTVLVTVTGADEDVAGEPKDGQRD***RR***TPSGFLGVR…

>*Lithobius forficatus* (Chilopoda) TKRP

…DNQPLSQILLHQRPFFDI***KR***LNNFMAMR**G*KK***DPFDATEMRKTQRLDSDIMSFLEA***KR***NRNIGFTGMR**G*KK***SQNEGE***RRRR***PESNYFAKFLGQR**G*KK***WLHGNRAETNMANTIQVHKNKNEGYQSNL*

>*Hanseniella* sp. (Symphyla) TKRP

…GFTDYS***KR***SKIPGFMGMR**G*KK***HASP***KK***EGKIGGFVGNR**G*KK***NMAMPWEAAQQNTVEII*

>*Eudigraphis takakuwai nigricans* (Diplopoda) TKRP

…KAETDDGVAENDQPFLELTQ***KR***GGGDGFYGMR**G*KK***ESRESEQQPDVLEDRFISSENADPTHDSLSSDLSIRK*

..............................................................................................

>*Drosophila melanogaster* TKRPa PA [FBpp0081962]

***M***RPLSGLIALALLLLLLLTAPSSAADTETESSGSPLTPGAEEPRRVV***KR***APTSSFIGMR**G*KK***DEEHDTSEGNWLGSGPDPLDYADEEADSSYAEN**G*RR***L***KK***APLAFVGLR**G*KK***FIPINNRLSDVLQSLEEERLRDSLLQDFFDRVAGRDGSAV**G*KR***APTGFTGMR**G*KR***PALLAGDDDAEADEATELQQ***KR***APVNSFVGMR**G*KK***DVSHQHY***KR***AALSDSYDLR**G*K***QQRFADFNSKFVAVR**G*KK***SDLEGNGVGIGDDHEQALVHPWLYLWGE***KR***APNGFLGMR**G*KR***PALFE*

>*Drosophila melanogaster* TKRP PB [FBpp0307416]

***M***RPLSGLIALALLLLLLLTAPSSAADTETESSGSPLTPGAEEPRRVV***KR***APTSSFIGMR**G*KK***DEEHDTSEGNWLGSGPDPLDYADEEADSSYAEN**G*RR***L***KK***APLAFVGLR**G*KK***FIPINNRLSDVLQSLEEERLRDSLLQDFFDRVAGRDGSAV**G*KR***APTGFTGMR**G*KR***PALLAGDDDAEADEATELQQ***KR***APVNSFVGMR**G*KK***DVSHQHY***KR***AALSDFWHTFFKKSYDLR***GK***QQRFADFNSKFVAVR**G*KK***SDLEGNGVGIGDDHEQALVHPWLYLWGE***KR***APNGFLGMR**G*KR***PALFE*

>*Daphnia pu*lex TKRP [[Dappu1_236286](http://genome.jgi.doe.gov/cgi-bin/dispGeneModel?db=Dappu1&id=236286), GenBank:[EFX86778.1](http://www.ncbi.nlm.nih.gov/protein/321475816?report=genbank&log$=protalign&blast_rank=1&RID=YES0MNP0015)]

***M***AVLMTVLAYCQPASAAATAVTDDDELMARQTRGLVLRSWRNAQQQTDHSADKTPSAKVAEPILPSQKEAMVFNGLPISMRLVLLQHLAGYD***KR***TPNSRAFLGMR**G*KK***SSPPGADALTMEDNQLDDASGWPQGDILPDTYYFGPAPQ***KK***KMHGEKFLGMR**G*KK***MMNGLADGTAFIPNWRERYIYQEPFEK***KR***APSSNSFMGMR**G*KR***SESTTPTPNDYQFFNDDIIVDEELPDVDSKVSPRRSQERTPI*

Trissin

>*Nipponentomon nippon* (Protura) trissin

***M***PSSTTIVLLLGLFMIVISQSEAERCNSCGRECVADCGGKNYRTCCMSFF***RRKK***SDQHFIRFLHELQKNDRGSDFDVPAKVALHCILSNDPECWKTASVIDSDFDFT***RR***EDSKQSPLLNLLLTDEKEEE*

>*Acerentomon* sp. (Protura) trissin [GenBank:GAXE01025862.1]

***M***QLILSRIFIVVSACCVLLAQSDPSCGTCGKECSHSCGTRSFRGCCMNYQ***RRKR***DGLLLEDEDSQPKLSLPRTAMADCSGVDAASCLEIIMEGKSQPMSELEYTRSKP***KR***PRDQQ…

>*Filientomon takanawanum* (Protura) trissin

***M***QAALLRILLVVSTCVIAISCAKYNSACGTCGDQCAYSCGTSNFRGCCMAFQ***RRKR***FSMFAAKQTETTPGCSDPEDPACLELLLDESSDSKGELDISRSKLS***RR***NEYSPFEGSVLGDLMFAEYVMRNNAD*

>*Anurida maritima* (Collembola) trissin [GenBank:GAUE01010527.1]

…AVLFPVLAVLNVRGSDDLVMSADGIQCNSCGAKCSLVCGSRQFKFCCLNFV***KR***SYPSIPDIL***RR***HIMSGGYEANGHAWIDPKYLSESQRHWNKPFKTETANAAEVLTEDSSPSNAFDDSFSIDRVDHHHSTSPLLIDLPVPLGNY*

>*Folsomia candida* (Collembola) trissin [GenBank:GASX01078951.1]

***M***HYPAFTLLFGLVFVTLGLAVTSSLALSDQELNLGIDGLRCDSCGMECSTVCGSRAFRTCCFNYI***KKKR***SGGSDEDRMRGSRGGGGKL***RR***GSGSNGYWVTAGEYFEPQMQVEGPWAWFYPQQPPMHQPPTSGGDDVEHGRNSRT…

>*Bourletiella hortensis* (Collembola) trissin1

***M***ASRIRFSLVMLVIGCLVTQLLIISVSSEEMGIEALNCNSCGRDCSAVCGTRQFRSCCFNYI***RKR***SSEPSSRSLLRSNELRSAGSSSSSSGGPSLVSVASLTDADSLLNNNNNNRMNNP…

>*Bourletiella hortensis* (Collembola) trissin2

…AFVGSEEYTAFNQEKV***RR***LDCTSCGEECAASCGTKLFRTCCLSFA***RKR***SGGFTGIDDIGNGSEEDWRGHISAEDSLTYNRLLQPPYFKTLQGQSHHP***KK***ESGSPKVVWSLMP*

>*Bourletiella hortensis* (Collembola) trissin3

…FVFLTLTNDVVNCEDMDFSLASGDMFSGIGNPGGPTCDDCGTRCKIMCRTRKFRLCCMQYM***RKR***SENLLANSGNNPDFDSFPTLPKHPITSCPSNYGN*

>*Bourletiella hortensis* (Collembola) trissin-like1

***M***EARVQAVAIRVLIVLLGVLALCSVGSAELELHGGSEAGLQCSTCGSECKILCAARQSRLFRSCCLNFM***RKR***ADPDWESFLMPTNRFL*

>*Bourletiella hortensis* (Collembola) trissin-like2

***M***KAIKTPGAALACLSITSNAVAETNVDPDYESYRSGGGGDEDPEDDVLIERIGGGGGGGG***R***YNRCNQCGAACKHKCGTRSFRSCCLRLM***RRKK***AQVGPDYLTD…

>*Bourletiella hortensis* (Collembola) trissin-like3

***M***KNLAVCLIILIGTMLEQTSGTQELESELAGGGSLSMMGMDGP***RR***APAACGNCHPGCLSKCDTRAYTFCCLSFL***RKR***APTDPLMDVLRWDGR…

>*Bourletiella hortensis* (Collembola) trissin-like4

***M***GHPSVYNVSLLLGLLCLLAIPCDSFGSHFEKFKLLSSGRFGLRAVQTTTPHPIAIAMNPICHVCHSACVSKCLTKDFKRCCYHFAKF***KR***GEADLMTID*

>*Pogonognathellus sp.* (Collembola) trissin [GenBank:GATD01086967.1]

…LKCFCISVGVFLAGAIMVNADHDLPMSVEGLRCDSCGRECSVVCGSRQFRTCCFNYI***KKR***GDPTLTGMVTGSETKFG***RR***INNEVDFTKDSIPSPAYVLAGPQVYISELLDRQPSFRQVVPS***KK***LPFDI*

>*Pogonognathellus sp.* (Collembola) trissin-like[GenBank:GATD01036714.1]

…FTIILSVMFIATLVWAATKEDCAACGKVCEPLCGKKKFRICCIQNM***RRKR***ESEADMVATSHSNLALAEEWFLPSVAS*

>*Sminthurus viridis* (Collembola) trissin1 [GenBank:GATZ01081393.1]

***M***GQGFTNFPIIFTVCILIVGQIVSVRPEEFSIENLGCDSCGRDCSAVCGSRQFRSCCFNYI***KKR***SSDSPA…

>*Sminthurus viridis* (Collembola) trissin2 ( GATZ01062781.1]

…VKASLAEAEDMLMGQDDDFEIYANNANYCDLCGNHCKFRCGTRRFRFCCHSFL***RKR***SVSRNNFK*

>*Sminthurus viridis* (Collembola) trissin3 [GenBank:GATZ01091084.1]

…TKTLECVLCGRYCNFKCGSRRFRSCCFRTL***RKR***GDIPKTWVKPTLPVMLELGVEAGGS***KR***GN*

>*Sminthurus viridis* (Collembola) trissin-like1 [GenBank:GATZ01030113.1]

***M***NSFPLVLALTLMLSGAVTSYSSDGDYMFAAEMQAG***R***GIDCRACGDECIVKCGTKMFRTCCFHFSN***KKKR***KGL…

>*Sminthurus viridis* (Collembola) trissin-like2 [GenBank:GATZ01093766.1]

***M***CRIQGIFLILIVGVVAIAAQSSAEMELHGGSGGLQCSMCGSECKILCTSRQSRLFRSCCSNFM***KKR***ADPDLDDFFTNTFT*

>*Sminthurus viridis* (Collembola) trissin-like3 [GenBank:GATZ01067123.1]

…ALLIVALLLSVTAPNLVTGLLEGELCANCGIECQQFCGSKYFRFCCVTFNKA***KR***SGWDDGLTWPAIDPFAPKIKNEWRIAG…

>*Sminthurus viridis* (Collembola) trissin-like4 [GenBank:GATZ01034242.1]

…TLEEVTSPSVSIIAME***C***FVCDQRCLPYCGSRRFKRCCRNFE***RKKR***AASVELFKHLSSME*

>*Sminthurus viridis* (Collembola) trissin-like5 [GenBank:GATZ01008296.1]

***M***GILALGVIMFALGTFVPDYAVGLGVGDGHPPDICESCGIPCRKFCGTDSFRRCCTNMQKP***KR***GWSG…

>*Jordanathrix leptothrix* (Collembola) trissin

***M***EARGRFRANYSTFLILFGILVAELLILPMRVRGNAEFSIDGLGCDSCGKDCSAVCGSRQFRSCCFNYI***KKR***SPGSPPQPPNSLNSNANNSPSSSNFEAELLDPSSFTSGSGGGGMNGRKSQASQIEIQNRVDGAQCGGQQEMEEEPMFSMSVPGGGGGSITIMISSSPITTLKNPGSSTNWNSHAFLVTETEIPNHLIR…

>*Jordanathrix leptothrix* (Collembola) trissin-like1

***M***SWKWYIVAIVLMNVVKFSLTTANSADKNEEGEVRYDGGCETCGPACKSSCNTYFFRFCCMGLFLKS***KR***GEASYDHDAGNLLEMRPEGSSKFENKPWRWRYM*

>*Jordanathrix leptothrix* (Collembola) trissin-like2

***M***ENNSTKTYFTIQLVLLIVFIGVLSSLQSEVSAETDFIAESGSLQCHMCGSECKQFCSTKQSRLFRSCCMNFM***RKR***SADTECPISYINENND*

>*Campodea augens* (Diplura) trissin [GenBank:GAYN01132259.1]

***M***KISAIFILGLLAMTVTWALNCDSCGRECTNACGTRTYRTCCFNYL***KKR***LTILPRAT***RR***DESWQRFNLQPSIHSTPYFIQALSPVDADWNILRDGNIHRSQSGDCKNVEQTSAPPDVETN*

>*Lepidocampa weberi* (Diplura) trissin

***M***MRVSAIFLLGILALSMSWALNCDSCGRECTNACGTRTYRTCCFNYL***KKR***LTILPRPS***RR***DEVWTPL***KK***LSLHTKPYLIRAIVPAEADWSMLKDDASPRSDYMPNDDCRNSEEPTPPPDMERN*

>*Occasjapyx japonicus* (Diplura) trissin [GenBank:GAXJ01029286.1]

***M***NPSPTKRPPCFLSSILSFRPGFVLSCAQSIAAFNCESCGRECTNACGTRTYRTCCFNYL***RKR***I*

>*Pedetontus okajimae* (Archaeognatha) trissin

***M***HGAILLSALLVLCMISRNVVAMAFTCDSCGRECKDACGTRSFRACCFNYV***RKR***GGKDTWEDGGGTLNGNEMENTIYGNWRMLPPT*

>*Machilis hrabei* (Archaeognatha) trissin [GenBank:GAUM01168561.1]

…AILLSALVVLCMVSRNAMAVTCDSCGRECKDACGTRNFRACCFNYL***RKR***GGRDAWEDGSAPEDDTAYINWRVVPPT*

>*Meinertellus cundinamarcensis* (Archaeognatha) trissin [GenBank:GAUG01234881.1]

***M***THGVIFLSALLVVCVVLKSTAAVSCDSCGRECSDACGTRNFRACCFNY***LRKR***SGDTTWEEGNPEDGSHPALMAWRLLPQS*

>*Machilontus* sp. (Archaeognatha) trissin

…VLSALVIVVCVMFRSSAAVSCDSCGRECSDACGTRNFRACCFNYL***RKR***SGASTSVASWDADAQDKPNRS…

>*Thermobia domestica* (Zygentoma) trissin [GenBank:GASN01364631.1]

***M***NGTTLLVSGILLWYACTWSVALSCDSCGRECTSSCGTRNFRTCCFNYL***RKR***GPPPPPPVEEQEPDIEFDTYFALPEKVAQGIAQLQQQ***RR***MMNPGDQRNIIARVHPNVVVDEDF*

>*Atelura formicaria* (Zygentoma) trissin [GenBank:GAYJ01302590.1]

***M***HGSTLLVSGILLWCAFTWSVAMSCDSCGRECTSACGTRSFRTCCFNYL***RKR***SFQMKEPEFHQESLVALPDLVAQGLAQLHE***KR***RTLSEDSPLEALHRKSLYQEYKDEDDF*

>*Nicoletia phytophila* (Zygentoma) trissin1

***M***MSSATLLVSGILLWCACTWSLALSCDSCGRECMSSCGTRSFRTCCFNYL***RKR***AEVPLHEPLVELDPVLALPDLVSRGFVRLHGQR***KR***PDNHDIYPTYSPYKEEEDF*

>*Nicoletia phytophila* (Zygentoma) trissin2

***M***IRQWSLLVAVGVILLASSLDWRFLPGAEAADCSICGPECTSACGTRLFRACCFNYN***RKR***SYEPPGKTMDGDDTDEDNGNEINDLDDVMLSQNLEPMVRHWARKEQEMESMQRRHQN***KR***RHWRRD*

..............................................................................................

>*Anaspides tasmaniae* (Malacostraca) trissin

…VYSCCNQYLGPYWTVWACVGGAWAWSSSEMSCSSCGTECQAACGTRNFRACCFNFQ***RRRR***GGGVTLVSKPEG*

>*Hanseniella* sp. (Symphyla) trissin

***M***TEQRRHFRVLPVTCMLLLLFHHWLSVNADPCNACGTECANACGTRVFRACCFNFN***RKR***RSFPPLLNAALH***KR***NRWTNEDIRNSEDPLVSGDYSEEADGENAGRNDVFGG…

>*Eudigraphis takakuwai nigricans* (Diplopoda) trissin

***M***LGSSKPITTFVVALSVVTFLLNVGCISVDACSACGPECASACGTPLFRACCFNYI***KKR***SDPTITRKAEALHDKQDLSLTSNQRNLTSTDALFAERSSNDLDSNAYTKFLTRNAPSRNHYAPSYNGDPSVAVISNEEDVSNAMDIMDFYNRFPDDGVDSVDSNRETNSAMLRMQKSRTASTRNADFLEDPIPDRIWVQQNGKPARLVKLYSYL*

..............................................................................................

>*Drosophila melanogaster* trissin [[FBpp0289411](http://flybase.org/reports/FBpp0289411.html)]

***M***TKTTMHWLAHFQIILLCIWLMCPPSSQAIKCDTCGKECASACGTKHFRTCCFNYL***RKR***SDPDALRQSSN***RR***LIDFILLQ**G*R***ALFTQELRE***RR***HNGTLMDLGLNTYYP*

no trissin known from *D. pulex*
