# Supplementary material for: Evolution of neuropeptides in non-pterygote hexapods
Source: BMC Evol Biol. 2016 Feb 29;16:51. doi: 10.1186/s12862-016-0621-4 (PMC4770511; doi:10.1186/s12862-016-0621-4)
Supplement: Additional file 2: — Sequence logos of single-copy peptides. The logos show the sequence variability in the single-copy peptides (<30 amino acids) of non-pterygote hexapod lineages determined using WebLogo version 2.8.2 (http://weblogo.berkeley.edu/). For species with two described genes containing different mature neuropeptides, the more conserved sequence was selected. CNMa was not found in Protura and Collembola, while elevenin was not found in Collembola. Pigment-dispersing factor (PDF) was not found in Protura and Diplura. For comparative purposes, the sequence conservation of CAPA-PVK paracopies was added as an example of multiple-copy peptides. A distinct core sequence is obvious in all sequence logos. The N-terminal sequence motifs are variable in a number of peptides, including SIFa, and this variability is similar to that of the N-termini in the different PVK paracopies. (DOC 460 kb) [file 12862_2016_621_MOESM2_ESM.doc]

**Additional file 2** (additional file 2.doc). *Sequence logos of single-copy peptides*. The logos show the sequence variability in the single-copy peptides (< 30 amino acids) of apterygote hexapod lineages determined using WebLogo version 2.8.2 (http://weblogo.berkeley.edu/). For species with two described genes containing different mature neuropeptides, the more conserved sequence was selected. CNMa was not found in Protura and Collembola, while elevenin was not found in Collembola. Pigment-dispersing factor (PDF) was not found in Protura and Diplura. For comparative purposes, the sequence conservation of CAPA-PVK paracopies was added as an example of multiple-copy peptides. A distinct core sequence is obvious in all sequence logos. The N-terminal sequence motifs are variable in a number of peptides, including SIFa, and this variability is similar to that of the N-termini in the different PVK paracopies.
